# Computational evaluation of metal pentazolate frameworks: inorganic analogues of azolate metal–organic frameworks†
†Electronic supplementary information (ESI) available: Details of database surveys, candidate structures, computational procedures, and topological analyses, in pdf format. Crystallographic data for theoretical Zn(**pnz**)_2_ and Cd(**pnz**)_2_ frameworks in CIF format. See DOI: 10.1039/c7sc05020h


**DOI:** 10.1039/c7sc05020h

**Published:** 2018-02-28

**Authors:** Mihails Arhangelskis, Athanassios D. Katsenis, Andrew J. Morris, Tomislav Friščić

**Affiliations:** a Department of Chemistry , McGill University , 801 Sherbrooke St. W. H3A 0B8 Montreal , Canada . Email: tomislav.friscic@mcgill.ca; b School of Metallurgy and Materials , University of Birmingham , Edgbaston , Birmingham B15 2TT , UK

## Abstract

Pentazolate is the ultimate all-nitrogen, inorganic member of the azolate series of aromatic 5-membered ring anions. As an azolate ligand, it has the potential to form open framework structures with metal ions, that would be inorganic analogues of azolate metal–organic frameworks formed by its congeners. However, while the low stability and elusive nature of the pentazolate ion have so far prevented the synthesis of such frameworks, computational studies have focused on pentazolate exclusively as a ligand that would form discrete metallocene structures. Encouraged by the recent first isolation and structural characterization of pentazolate salts and metal complexes stable at ambient conditions, we now explore the role of pentazolate as a framework-forming ligand. We report a computational periodic density-functional theory evaluation of the energetics and topological preferences of putative metal pentazolate frameworks, which also revealed a topologically novel framework structure.

## Abstract 2

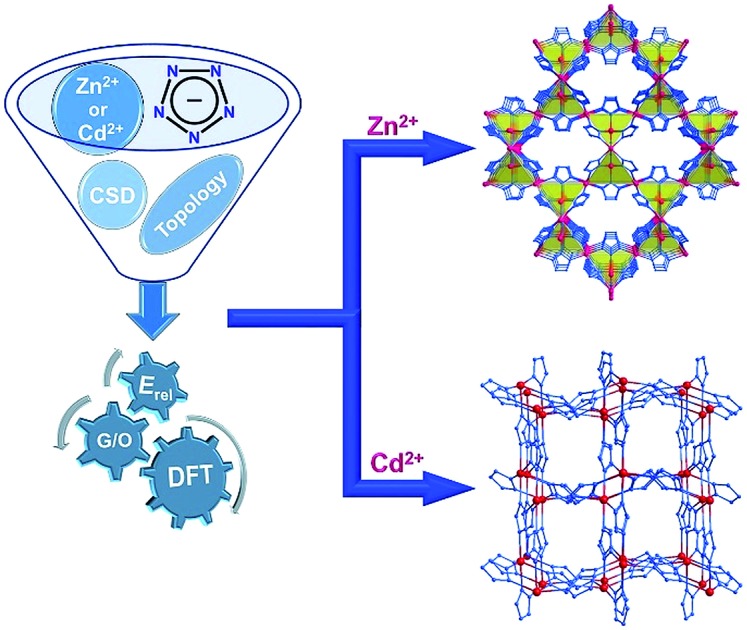
We report a periodic density-functional theory evaluation of putative frameworks, including a topologically novel arhangelskite (*arh*) structure, based on the pentazolate ion, the ultimate all-nitrogen, inorganic member of the azolate series of aromatic 5-membered ring anions.

## Introduction

**Fig. 1:**
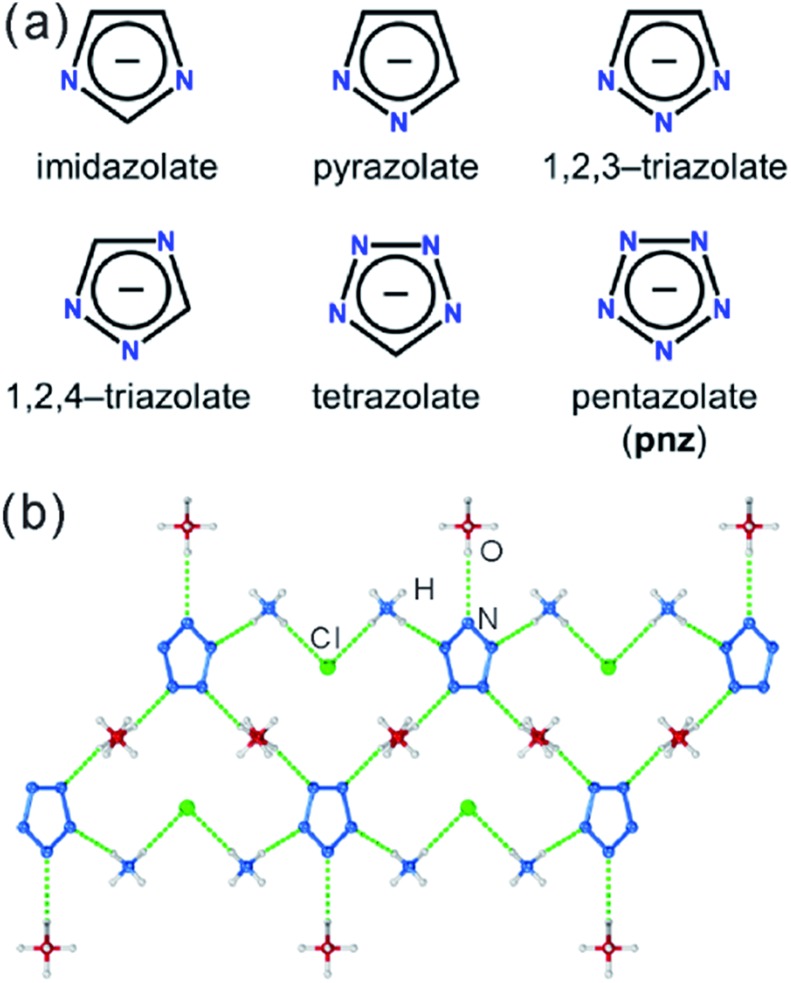
(a) Cyclic azolate anions; (b) fragment of the crystal structure of the first pentazolate compound stable under ambient conditions, reported by Zhang *et al.*^4^

Pentazolate, *cyclo*-N_5_^–^(**pnz^–^**), is the ultimate all-nitrogen member of the azolate class of aromatic 5-membered ring anions, whose compounds have until recently been all but impossible to synthesize.^1^ In 2017, computational structure predictions indicated stability of ammonium pentazolate at pressures above 30 GPa,^2^ which was followed by a high-pressure synthesis of cesium pentazolate from the corresponding azide.^3^ The first pentazolate compounds stable at ambient conditions were reported only very recently, as Zhang *et al.* described the mixed salt (H_3_O)_3_(NH_4_)_4_(**pnz**)_6_Cl composed of pentazolate and chloride anions forming a hydrogen-bonded net with hydronium and ammonium counterions (Fig. 1b).^4^ This was followed by isolation and structural characterization of a hydrated sodium pentazolate salt, and of simple hydrated metal complexes M(H_2_O)_4_(**pnz**)_2_·4H_2_O (M = Mn, Fe, Co, Zn), in which **pnz^–^** acts as a monodentate ligand.^5^–^7^ Remarkably, both the mixed ammonium salt and the transition metal complexes of **pnz^–^** do not decompose below 100 °C, holding promise for the synthesis of further compounds of **pnz^–^**. Finally, a series of sodium-pentazolate framework materials with zeolitic topologies were very recently reported, where the **pnz^–^** ligands were stabilized by coordinating to sodium.^8^ The demonstrated ability of **pnz** anion to form transition metal complexes and sodium-based frameworks suggest that **pnz^–^** should be capable of forming extended coordination frameworks with transition metals, analogous to metal azolate frameworks (MAFs),^9^,^10^ a popular class of metal–organic frameworks based on azolate ligands containing two (imidazolates,^11^,^12^ pyrazolates), three (triazolates)^13^ or four (tetrazolates)^13^,^14^ nitrogen atoms (Fig. 1a).^10^ Indeed, the structure of Na(H_2_O)**pnz**·2H_2_O^5^ shows **pnz^–^** ions bridging Na^+^ centers in a manner seen in metal pyrazolates.^15^ While such pentazolate frameworks might have applications as energetic materials,^16^ they would also be intrinsically interesting as direct inorganic structural analogues of azolate metal–organic frameworks, and as first examples of framework materials based on an aromatic inorganic linker.^17^

We now report a computational investigation of the topological landscape and enthalpic stability of putative pentazolate frameworks Zn(**pnz**)_2_ and Cd(**pnz**)_2_, including their potential as energetic materials.^16^ Compared to zeolites and related tetrahedral structures, for which computational structure prediction and modelling are well established,^18^–^27^ the use of theoretical calculations to evaluate or predict properties of metal–organic frameworks is very recent, with notable work focusing on thermodynamic stability,^28^–^30^ gas storage capacity,^31^,^32^ catalysis^33^ or organic linker flexibility.^34^,^35^ Our study was encouraged by the recent demonstration of the ability of periodic density-functional theory (DFT) calculations to provide a realistic assessment of topological preferences and enthalpic stability of imidazolate-based MAFs.^36^ Importantly, whereas earlier theoretical work has focused on **pnz^–^** as a ligand that should form discrete molecular complexes of the metallocene type,^37^,^38^ this study is the first to consider the formation of extended frameworks based on **pnz^–^**.

Due to the challenges of applying *ab initio* crystal structure prediction (CSP) techniques,^39^–^41^ to 3D-covalent structures that have so far prevented their application to coordination frameworks, we have based our study on an extensive survey of the Cambridge Structural Database (CSD) for framework structures composed of divalent metal ions bridged by azolate linkers in the respective stoichiometric ratio 1 : 2. The **pnz^–^** anion contains five nitrogen atoms potentially available for interacting with metal ions, indicating that metal-binding geometries accomplished through all types of azolates, *i.e.* imidazolates, pyrazolates, triazolates and tetrazolates, should be relevant to our study. We focused on zinc and cadmium as metal nodes, as they are highly popular in the synthesis of MAFs, and also exhibit the d^10^ electronic configuration which is particularly amenable to DFT calculations. Other divalent ions with incomplete d-shells, such as Co^2+^ or Fe^2+^, would require explicit treatment of magnetic states, which represents a particular challenge for standard local DFT functionals.^42^

## Computational methods

### Selection of structures and plane-wave DFT calculations

The CSD structure selection process made no restrictions on metal coordination number or geometry. Structures found in the database were manually screened for duplicates, resulting in a final set of 39 framework structures that were distinct in terms of three-dimensional (3-D) atomic arrangements, as seen upon structure overlay. These unique structures were then manipulated to generate putative polymorphs of Zn(**pnz**)_2_ or Cd(**pnz**)_2_, by removing all azolate ring substituents, guest molecules, and setting the type of all azolate ring atoms to nitrogen. Finally, the metal atom type was set to zinc or cadmium. The resulting structures were then geometry-optimized using the plane-wave DFT code CASTEP.^43^ Crystal structures were total-energy minimized with respect to unit cell parameters and atomic coordinates, subject to symmetry constraints of the corresponding space groups. The DFT calculations were made using PBE^44^ functional in combination with the plane-wave implementation^45^ of Grimme D2 (ref. 46) dispersion correction. Most of the commonly used DFT functionals underestimate the energy of van der Waals interactions and, when applied to porous metal–organic frameworks, this may lead to errors in calculated energies and lattice parameters.^34^,^47^–^50^ It is therefore important to compensate for the poor description of van der Waals interactions by utilizing functionals tailored for accurately reproducing long-range van der Waals interactions,^51^,^52^ symmetry-adapted perturbation theory (SAPT-DFT)^53^ approaches, or semi-empirical dispersion corrections (SEDC).^46^,^54^–^56^ Such dispersion-corrected DFT calculations were shown to improve energy rankings of ZIFs^28^,^57^,^58^ in comparison to plain DFT calculations.^59^

Throughout our study we have used PBE exchange-correlation functional combined with Grimme-D2 (ref. 46) dispersion correction. In this scheme the dispersion energy is evaluated as a sum of interatomic pairwise contributions, based on a pre-parameterized set of C_6_ coefficients for elements H–Xe. In order to evaluate the accuracy of PBE-D2 calculations, we have performed additional calculations with Tkatchenko–Scheffler (TS)^55^ and many body dispersion (MBD*)^56^,^60^,^61^ correction schemes. The TS approach derives pairwise C_6_ dispersion coefficients from the DFT-calculated electron density, as opposed to parameterization used in the D2 scheme. The MBD* adds many body terms to the TS pairwise dispersion energy. As the CASTEP implementation of MBD* method is currently in development, the full geometry optimization using the PBE + MBD* method was not possible. However, similarity of calculated geometries permitted single point calculations using the geometries obtained from PBE + D2 and PBE + TS calculations. In order to evaluate the importance of dispersion corrections on the final energy ranking of **pnz** frameworks, energies were also calculated for a subset of structures using uncorrected PBE^44^ and LDA^62^ functionals.

The plane-wave cut-off was set to 750 eV and the norm-conserving pseudopotentials^63^ were used. The Brillouin zone was sampled with a Monkhorst–Pack^64^*k*-point grid of 0.03 Å^–1^ spacing. The following convergence criteria were used: maximum energy change 10^–5^ eV per atom, maximum force on atom 0.03 eV Å^–1^, maximum atom displacement 0.001 Å and residual stress 0.05 GPa.

Energy minimization of putative crystal structures provided energy landscapes for topologically distinct Zn(**pnz**)_2_ and Cd(**pnz**)_2_ frameworks (Table 1). The structure optimization sometimes led to structures of identical topologies, but of different energies due to differences in symmetry. An example of this would be Zn(2-methylimidazolate)_2_ (CSD code OFERUN01, space group *P*2_1_/*c*)^65^ and Zn(5-methyltetrazolate)_2_ (CSD code HOKMUR, space group *Pc*),^66^ which share the same diamondoid (*dia*) topology, yet have significantly different unit cell parameters and different space group symmetries. These structural differences result in crystallographically distinct structures for both Zn(**pnz**)_2_ and Cd(**pnz**)_2_ frameworks. While such hypothetical structures, in principle, represent isoreticular polymorphs, their detailed description and comparison would not be justified considering the accuracy of the herein used approach. Consequently, in our analysis we focused on lowest energy calculated structure for each topology, with a complete set of minimized structures provided in the ESI.†

**Table 1:** Relative energies (*E*_rel_), topologies, coordination number (CN), packing coefficient (PC) and CSD codes of original structures for ten lowest-energy calculated structures of Zn(**pnz**)_2_

CSD code	*E*_rel_ (kJ mol^–1^)	Topology	CN	PC
WAQQUB	0.000	*crs*	6	0.454
LIHQUP	12.989	Interpenetrated *dia*	4	0.662
IMIDZB01	20.887	*zni*	4	0.555
IMIDZB07	23.044	*coi*	4	0.548
ONATUT	25.249	*sql*	4	0.615
GUPBOJ	30.472	*yqt1*	4	0.519
CUIMDZ03	37.404	*mog*	4	0.532
GUPBOJ01	37.730	*ict*	4	0.437
HIFWAV	39.188	*nog*	4	0.369
GITTEJ	40.612	*crb*	4	0.397

To evaluate the thermodynamic feasibility of Zn(**pnz**)_2_ and Cd(**pnz**)_2_ in comparison to the reported mixed pentazolate salt,^4^ as well as their energies of combustion, we have also calculated energies for elements in their standard states, namely zinc and cadmium metals, O_2_, N_2_, H_2_, Cl_2_, and H_2_O (all in gas phase), as well as energies of zinc and cadmium oxides, which are expected to be principal combustion products.

Finally, phonon and electronic density of states (DOS) calculations were performed for a selection of lowest-energy structures of Zn(**pnz**)_2_ and Cd(**pnz**)_2_. The DOS and projected density of states (PDOS) were evaluated using the program OptaDOS.^67^,^68^ The purpose of phonon calculations was to verify the absence of imaginary frequencies in the putative metal pentazolate framework structures, while the DOS calculations were used to investigate the optical properties of these materials. Details of these periodic DFT calculations are given in the ESI.†

### Topological analysis

Topological analysis of the optimized structures was performed using the program ToposPro.^69^ In order to ensure that any changes in framework connectivity resulting from the optimization procedure are significant, we performed extensive searches of the CSD (see ESI† for the description of this analysis), which indicated that Zn–N and Cd–N distances shorter that 2.20 Å and 2.50 Å, respectively, should be considered bonds in topological analysis. These cut-offs in the assignment of metal–nitrogen bonds were validated by Mulliken population analysis^70^,^71^ in the plane-wave implementation.^72^ Bond populations were found to correlate well with the corresponding interatomic distances (see ESI Fig. S4–S5†), confirming the physical significance of the chosen bond cut-offs. In addition, as a further criterion for bond assignment, it was found that nitrogen atoms coordinated to metal centers are more negatively charged than the uncoordinated nitrogen atoms (ESI Tables S2 and S3†).

## Results and discussion

**Fig. 2:**
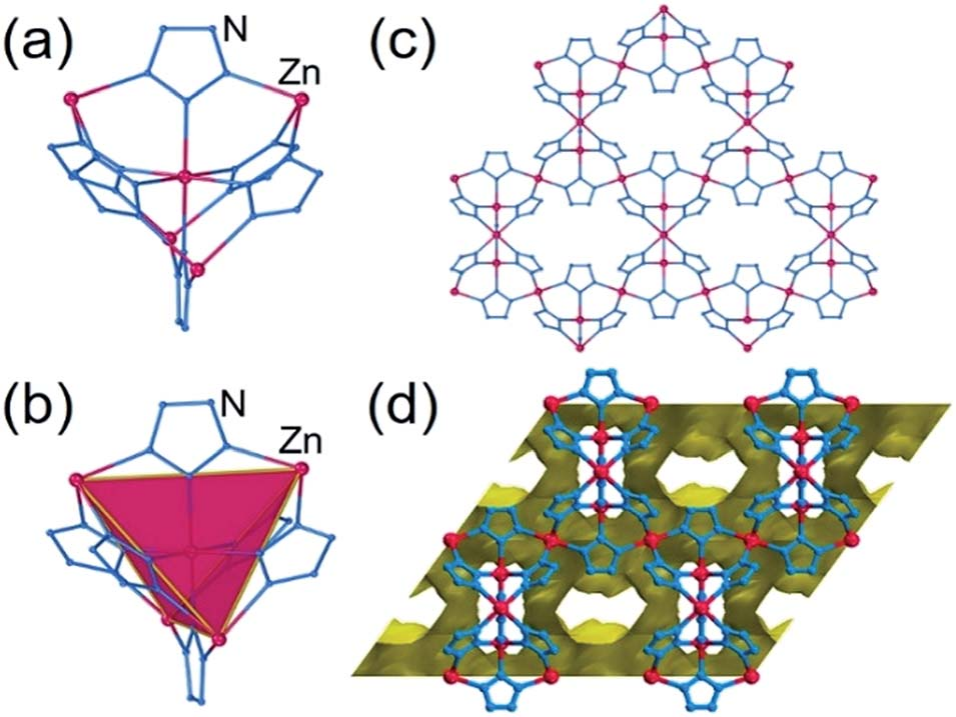
Structure of *crs*-Zn(**pnz**)_2_: (a) the pentanuclear Zn_5_(**pnz**)_6_ SBU with (b) the tetrahedral orientation of the peripheral Zn^2+^ ions highlighted; (c) view of the *crs*-Zn(**pnz**)_2_ framework along the crystallographic c-direction and (d) view of the *crs*-Zn(**pnz**)_2_ framework along the crystallographic *c*-direction, displaying the contact surface area calculated for a spherical probe of 1.2 Å radius.^74^

The hypothetical lowest-energy framework structure for Zn(**pnz**)_2_ adopts the *crs*-topology (Fig. 2), and results from optimizing either the zinc 5-methyltetrazolate framework with the CSD code WAQQUB, or the copper(ii) 1,2,3-triazolate structure with the CSD code CAYBAH.^73^ Hypothetical *crs*–Zn(**pnz**)_2_ contains two distinct octahedrally-coordinated Zn^2+^ ions. One type of zinc ion is surrounded by six nitrogen atoms from neighboring **pnz^–^** anions, producing a regular octahedron with Zn–N bonds of 2.14 Å. Each of these six **pnz^–^** ligands is further attached to two further, symmetrically equivalent Zn^2+^ ions to form a pentanuclear tetrahedral supramolecular building unit (SBU). Each SBU involves four zinc ions at its vertices, as well as an additional one at the center of the tetrahedron (Fig. 2a and b). The octahedral geometry of zinc ions at the vertices is completed by three **pnz^–^** ligands from a neighboring SBU, with Zn–N distances of 2.16 Å.

The structure is best described through vertex-sharing tetrahedra in the *crs*-topology (Fig. 2c), although it can also be described as a 3,6,6T1-net with each **pnz^–^** as a 3-c node and zinc atoms as 6-c nodes. Importantly, the structure of *crs*-Zn(**pnz**)_2_ is potentially microporous (Fig. 2d), with a low packing coefficient of 0.454.

**Fig. 3:**
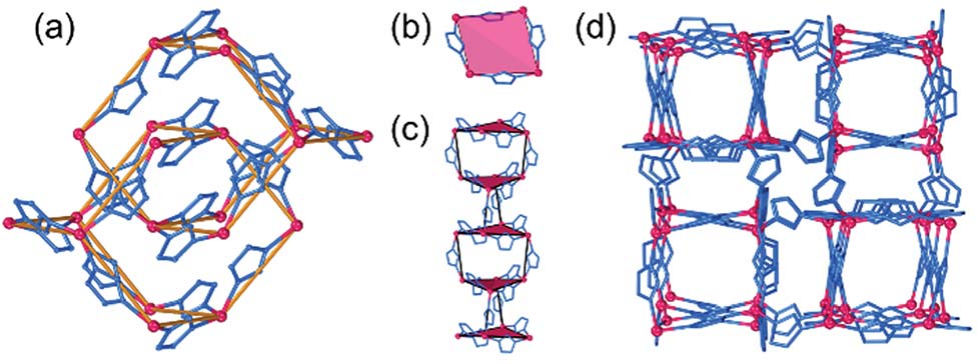
(a) Fragment of the interpenetrated *dia*-Zn(**pnz**)_2_ structure, viewed approximately along the crystallographic *a*-direction, with the two interpenetrating nets highlighted. Different views of the *zni*-Zn(**pnz**)_2_ framework: (b) 4-membered rings, (c) narsarsukite chains and (d) view of the framework displaying parallel orientation and connectivity of the narsarsukite chains.

The second lowest-energy framework topology for Zn(**pnz**)_2_ resulted from optimizing structures with CSD codes LIHQUP (zinc triazolate) and WAQRAI (zinc tetrazolate). Optimization led to almost identical singly-interpenetrated *dia*-topology structures that were 13.0 kJ mol^–1^ and 14.5 kJ mol^–1^ higher than *crs*-Zn(**pnz**)_2_, respectively. Each zinc atom in *dia*-Zn(**pnz**)_2_ adopts a tetrahedral geometry, with Zn–N distances in the range of 1.98–2.01 Å, and each **pnz^–^** links two metal nodes through 1,3-nitrogen atoms (Fig. 3a). The third lowest-energy Zn(**pnz**)_2_ framework adopts the *zni* topology, considerably higher (>20 kJ mol^–1^) in energy than *crs*-Zn(**pnz**)_2_. The ligands in *zni*-Zn(**pnz**)_2_, bridge zinc atoms to form 4-membered rings, bridged by further **pnz^–^** anions to form narsarsukite-type chains (Fig. 3a and b), further connected into a 3-dimensional (3-D) network by remaining **pnz^–^** anions (Fig. 3c).^75^

Relative energy (*E*_rel_) ordering of topologies for Zn(**pnz**)_2_ is very different compared to popular imidazolate MAFs (Table 1): the non-interpenetrated *dia*-, *qtz*- or *zni*-topologies, prominent as lowest-energy structures for imidazolates, are all 20 kJ mol^–1^ or more higher in energy than *crs*-Zn(**pnz**)_2_.

Notably, *crs*-Zn(**pnz**)_2_ is the lowest-energy structure in our screen despite exhibiting a potentially microporous structure, with a packing coefficient lower than a number of other herein considered structures. However, such higher density structures are based on four-coordinated zinc atoms, compared to the octahedral coordination found in *crs*-Zn(**pnz**)_2_. This indicates that forming additional Zn–N bonds, which is more likely for **pnz^–^** than for other azolates, can contribute more to stabilizing Zn(**pnz**)_2_ than creating non-covalent interactions in densely-packed structures. This again contrasts imidazolate frameworks, where the denser structures have so far been found to be the more stable ones, both experimentally and theoretically.^36^,^76^ Due to absence of substituents on **pnz^–^**, it is expected that non-covalent interactions should be less important for framework stability compared to other MAFs. Specifically, detailed analysis of Zn(**pnz**)_2_ structures using Olex2 77 revealed no π···π stacking interactions (ESI Table S26†).

The lowest-energy structure for hypothetical Cd(**pnz**)_2_ was obtained by optimization of the zinc tetrazolate structure with CSD code WAQRAI. The optimization led to a tri-nodal 3-D network with a novel topology with net point symbol {4.6^2^}_2_{4^2^.6^9^.8^4^}, herein named arhangelskite (*arh*) (Table 2). The novelty of the *arh* topology was confirmed with the aid of ToposPro software and a new entry has been added to the TOPOS Topological Database (TTD).^69^,^78^

**Table 2:** Relative energies (*E*_rel_), topologies, coordination number (CN), packing coefficient (PC) and CSD codes of original structures for ten lowest-energy calculated structures of Cd(**pnz**)_2_

CSD code	*E*_rel_ (kJ mol^–1^)	Topology	CN	PC
WAQRAI	0.000	*arh*	6	0.647
WAQQUB	9.905	*crs*	6	0.396
AXIVAF	16.654	*bcu*	6	0.587
LIHQUP	17.783	Interpenetrated *dia*	4	0.431
CUIMDZ02	30.317	4,4L37	4	0.692
ONATUT	30.739	*seh*-3,5-Pbca	5	0.54
BOJXAZ	31.159	*sql*	4	0.588
CUIMDZ03	39.198	{4.6^2^}_2_{4.6^9^}_2_{6^6^}	4, 5	0.547
IMIDZB01	39.921	*zni*	4	0.558
IMIDZB07	33.756	*coi*	5	0.506

**Fig. 4:**
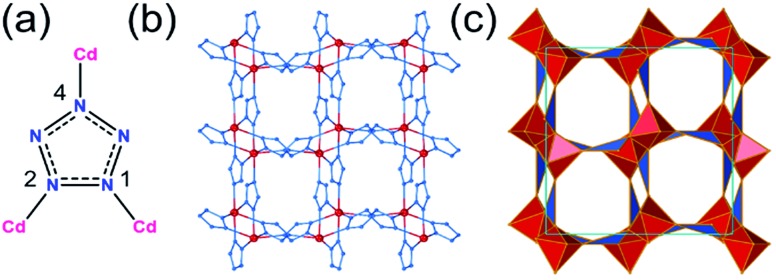
The *arh*-topology Cd(**pnz**)_2_: (a) coordination of **pnz^–^** in *arh*-Cd(**pnz**)_2_; (b) fragment of the *arh*-Cd(**pnz**)_2_ structure viewed along the crystallographic *b*-direction, and (c) corresponding view of the *arh*-framework in its augmented form, with each node represented by a vertex figure.

The *arh*-Cd(**pnz**)_2_ topology results from additional Cd–N bonds formed between components of two interpenetrated *dia*-nets in the original structure. This transforms the initially tetrahedral metal nodes to distorted octahedral ones, with Cd–N bonds ranging from 2.33 Å to 2.44 Å, with each **pnz^–^** ligand acting as a 3-c node with 1,2,4-coordination (Fig. 4). The *arh*-Cd(**pnz**)_2_ structure shows a high packing coefficient of 0.647, with narrow channels of *ca.* 2.4 Å diameter that propagate along the crystallographic *b*-axis. The next lowest-energy polymorph of Cd(**pnz**)_2_ adopts a *crs*-topology framework structure (low packing coefficient of 0.396) which has been described above for the zinc analogue. The *crs*-Cd(**pnz**)_2_ structure was generated from the structure of either zinc 5-methyltetrazolate (CSD code WAQQUB) or copper(ii) 1,2,3-triazolate (CSD code CAYBAH), leading to almost identical structures that are +9.91 kJ mol^–1^ and +9.97 kJ mol^–1^ higher in energy compared to *arh*-Cd(**pnz**)_2_, respectively.

**Fig. 5:**
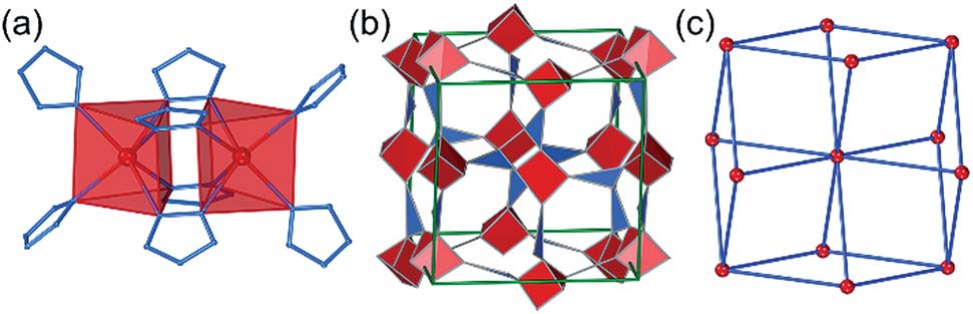
Structure of *bcu*-Cd(**pnz**)_2_: (a) dinuclear Cd_2_(**pnz**)_6_ units; (b) view of the structure with the Cd^2+^ ions and **pnz^–^** ligands replaced by their vertex figure and (c) depiction of the *bcu*-topology, where each node represents a Cd_2_(**pnz**)_6_ dinuclear unit.

The third lowest-energy framework is *bcu*-Cd(**pnz**)_2_, based on the structure with the CSD code AXIVAF (Fig. 5, for detailed description of the geometry optimization see ESI†). In this structure, **pnz^–^** ligands adopt the 1,2,4 coordination mode and form dinuclear units with the formula Cd_2_(**pnz**)_4_, where each Cd^2+^ ion adopts a trigonal prismatic coordination geometry (Fig. 5a). Each dimer is connected to further eight dimers through Cd–N bonds involving the nitrogen atom in position 4 of the **pnz^–^** ligand. Considering each dimer as an 8-coordinated node, the underlying framework topology is *bcu* (Fig. 5c).

**Fig. 6:**
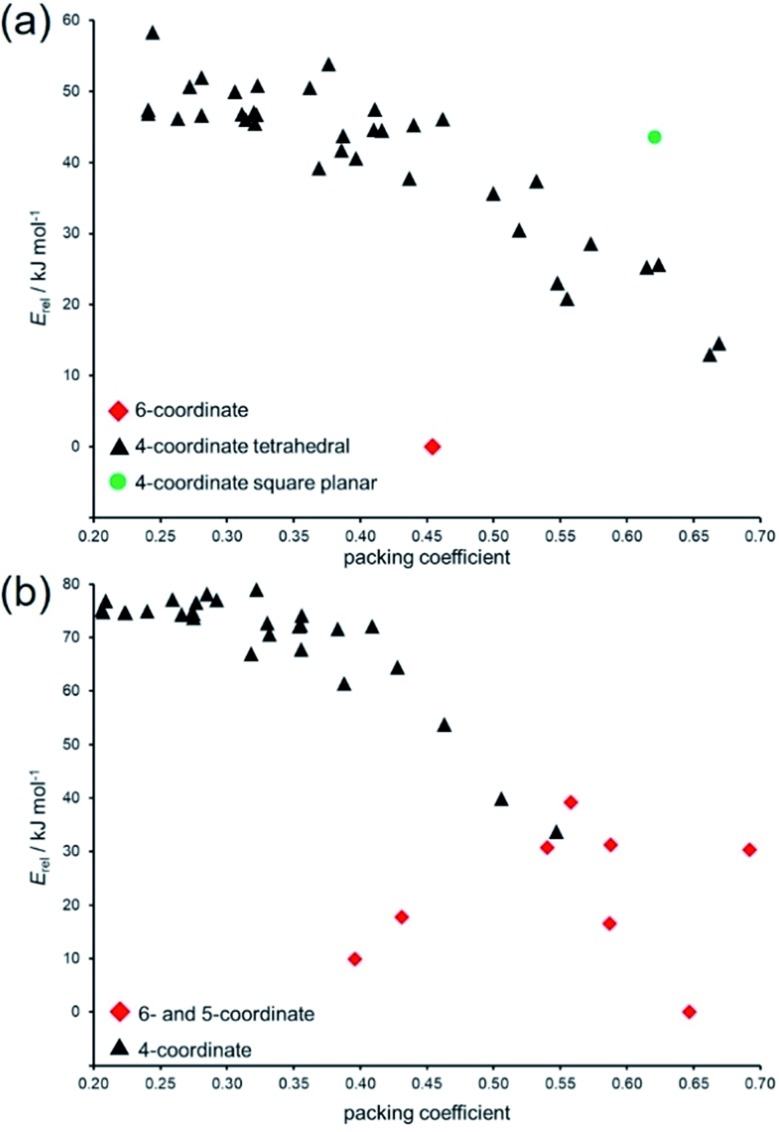
Comparison of relative lattice energies (*E*_rel_) and packing coefficients for putative: (a) Zn(**pnz**)_2_ and (b) Cd(**pnz**)_2_ framework structures.

Variation in packing coefficient of the lowest-energy hypothetical structures for Zn(**pnz**)_2_ and Cd(**pnz**)_2_ shows that changes in the coordination number of the metal ion are a significant factor in deciding the energies of pentazolate frameworks. The energy landscapes of Zn(**pnz**)_2_ and Cd(**pnz**)_2_ show different preferences for the metal coordination environment: the landscape for Zn(**pnz**)_2_ contains one 6-coordinate structure, the rest being based on 4-coordinate, almost exclusively tetrahedral metal nodes (Fig. 6a).

In contrast, Cd(**pnz**)_2_ shows a stronger preference for higher coordination number, explained by the larger size of the cadmium atom (Fig. 6b). In terms of intermolecular interactions, analysis using Olex2 (ref. 77) suggests that π···π stacking is not a significant factor in stabilizing Cd(**pnz**)_2_ structures: close π···π contacts of **pnz^–^** rings were only observed in higher energy structures with 4,4L37- and {4.6^2^}_2_{4.6^9^}_2_{6^6^}-topologies.

Phonon calculations were also conducted for selected putative low-energy structures of Zn(**pnz**)_2_ and Cd(**pnz**)_2_, confirming the absence of imaginary frequencies (see ESI, Tables S21–25†).

**Fig. 7:**
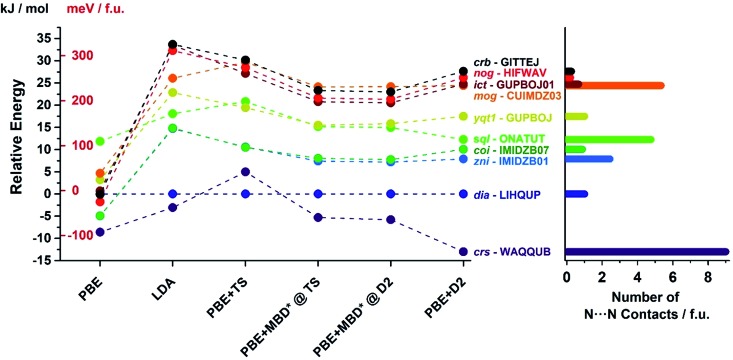
(Left) Energy ranking of selected Zn(**pnz**)_2_ structures using dispersion-corrected PBE functional as well as uncorrected PBE and LDA. For the D2 and TS corrections full geometry optimisation was performed. For the development version of MBD*, it was not possible to perform dispersion correction geometry optimization and, therefore, single point calculations were performed for structures optimized with PBE + D2 and PBE + TS methods. (Right) Number of short (3.0–3.1 Å) N···N contacts found in each structure using Olex2.^77^ All three dispersion corrections provide consistent energy rankings with the exception of *crs*-WAQQUB structure, which is found at the global minimum with D2 and MBD* methods, and is ranked second with TS dispersion correction. The anomalous behaviour of WAQQUB is correlated with the number of short N···N contacts present in the structure. The energy rankings produced by uncorrected PBE are entirely different from dispersion-corrected calculations, whereas the LDA-derived energy rankings are in good agreement with those obtained from dispersion-corrected PBE.

In order to confirm the accuracy of our predictions on metal pentazolate framework energies and topology predictions, as well as to verify that the observed trends are general and independent of the choice of computational method, we have performed additional calculation on a subset of topologically-distinct structures of Zn(**pnz**)_2_ and Cd(**pnz**)_2_ (Tables 1 and 2) using Tkatchenko–Scheffler (TS)^55^ and many body dispersion (MBD*) correction schemes. The results were also compared to those obtained using PBE and LDA functionals without dispersion corrections. The energy rankings of Zn(**pnz**)_2_ structures produced by three dispersion-corrected methods were generally consistent, the notable exception being the *crs*-Zn(**pnz**)_2_ structure (Fig. 7, also ESI Table S6†). Under the PBE-D2 method this structure is the global energy minimum, separated by 13.0 kJ mol^–1^ from the second lowest structure with interpenetrated *dia*-topology. The ranking of these two structures was reversed using the PBE-TS method, with interpenetrated *dia*-Zn(**pnz**)_2_ now becoming the energy minimum at a separation of 5.0 kJ mol^–1^ from *crs*-Zn(**pnz**)_2_. Finally, with PBE-MBD* method the *crs*-Zn(**pnz**)_2_ structure again becomes the global energy minimum, separated by 5.3 kJ mol^–1^ from the second-ranked *dia*-Zn(**pnz**)_2_ structure. Therefore, the many-body dispersion terms appear to stabilize the *crs*-structure more, relative to pairwise-only TS dispersion approach.

The *crs*-structure is the only Zn(**pnz**)_2_ structure containing an octahedrally coordinated metal ion, and also has the highest number of very short (3.0–3.1 Å) N···N contacts (Fig. 7). The behavior of *crs*-Zn(**pnz**)_2_ upon switching between D2, TS and MBD* dispersion correction schemes suggests that TS penalizes short-range interactions more heavily than D2 or MBD*. This observation is buttressed by the analogous behavior of higher energy *sql*- and *mog*-Zn(**pnz**)_2_ structures. These two structures also exhibit a significant number of short intermolecular N···N contacts and undergo similar, but less pronounced, variation in calculated energy between TS, D2 and MBD* dispersion correction schemes.

For Cd(**pnz**)_2_, all three dispersion correction approaches showed consistency in the lowest two energy structures with *arh*- and *crs*-topologies. The energy rankings of the third- and fourth-ranked structures of *bcu*-Cd(**pnz**)_2_ and *dia*-Cd(**pnz**)_2_ were reversed under PBE + TS and PBE + MBD* schemes. The result is not surprising, as the energy separation of these structures under the PBE-D2 method was only 1.1 kJ mol^–1^ under PBE-D2 method.

In general, all three dispersion correction schemes provide consistent energy rankings and unit cell volumes for Zn(**pnz**)_2_ and Cd(**pnz**)_2_. While some reranking is observed, it generally occurs for the structures separated by less than 5 kJ mol^–1^, the only exception being the *crs*-Zn(**pnz**)_2_ structure. In contrast, the uncorrected PBE functional produces energy ranking entirely different from dispersion-corrected PBE. As an example, a low-density structure *crb*-Zn(**pnz**)_2_ fell from rank 10 under PBE-D2 (+40.6 kJ mol^–1^) to rank 4 for uncorrected PBE (+8.5 kJ mol^–1^). Evidently, accounting for dispersion forces is crucial for the accurate evaluation of relative enthalpic stability of topologically distinct polymorphs of metal pentazolate frameworks.

We have also explored the ranking of the structures Zn(**pnz**)_2_ and Cd(**pnz**)_2_ structures using the LDA functional. In comparison with GGA functionals, LDA shows over-binding, resulting in more attractive supramolecular interactions. Indeed, the energy rankings of **pnz** frameworks produced by LDA were very similar to those generated by dispersion-corrected PBE, hinting at its use as a “poor man's energy dispersion correction”. The unit cell volumes produced by LDA calculations were all found to be *ca.* 5–10% lower than the volumes obtained by PBE-D2 calculations for the corresponding structures (Tables S8 and S9†), which is interpreted as a manifestation of stronger supramolecular forces in LDA structures.

In order to provide a preliminary assessment whether zinc- or cadmium-based pentazolate frameworks are experimentally accessible, we evaluated their enthalpies of formation with respect to the elements, by subtracting the energies of the elements in their standard states (crystalline zinc and cadmium, N_2_ gas) from the calculated energies of *crs*-Zn(**pnz**)_2_ and *arh*-Cd(**pnz**)_2_ structures. Analogous calculation was also done for the reported structure of the mixed salt (H_3_O)_3_(NH_4_)_4_(**pnz**)_6_Cl. The formation enthalpies for the lowest-energy structures of Zn(**pnz**)_2_ and Cd(**pnz**)_2_ were found to be +221.6 kJ mol^–1^ and +266.2 kJ mol^–1^ respectively. The formation of the mixed **pnz^–^** salt, on the other hand, was found to be exothermic with an enthalpy of –59.0 kJ mol^–1^ per **pnz** unit, consistent with its observed stability under ambient conditions.

While the positive enthalpies of formation suggest that metal pentazolate frameworks would be significantly less stable than (H_3_O)_3_(NH_4_)_4_(**pnz**)_6_Cl, this does not imply that Zn(**pnz**)_2_ and Cd(**pnz**) should not be feasible: enthalpies of formation for known solid energetic compounds readily exceed +240 kJ mol^–1^,^79^ as illustrated by hexahydro-1,3,5-trinitroso-1,3,5-triazine (TIT, +286 kJ mol^–1^),^80^ hexanitroazobenzene (HNAB, +284 kJ mol^–1^)^79^ or ε-hexanitrohexaazaisowurtzitane (CL-20, +377 kJ mol^–1^).^81^ Moreover, MAFs are typically obtained by solution crystallization, where reaction thermodynamics are strongly influenced by solvent–solute interactions, which might facilitate the formation of herein described structures. Indeed, complexation with Zn^2+^ ions was noted to stabilize **pnz^–^** in solution.^1^ The solvent might also have a significant effect on the accessibility of herein described structures: inclusion of solvent molecule guests can provide significant stabilization and guide the formation of some of the lower density Zn(**pnz**)_2_ and Cd(**pnz**)_2_ frameworks. Indeed, it may be envisaged that a program code such as ZEBEDDE could be utilized to identify suitable molecular templates for the synthesis of herein described Zn(**pnz**)_2_ and Cd(**pnz**)_2_ frameworks.^82^–^84^ Alternatively, a potential route to **pnz** frameworks may be *via* high pressure reactions of zinc^85^ or cadmium^86^ azides with N_2_ gas, analogous to high pressure syntheses of Na(**pnz**)^87^ or Cs(**pnz**) from corresponding azides.^3^ Positive formation enthalpies of *crs*-Zn(**pnz**)_2_ and *arh*-Cd(**pnz**)_2_ suggest their potential as energetic materials, which was evaluated by calculating the enthalpies of combustion for the reaction:

1 M(N_5_)_2_(s) + 1/2O_2_(g) → MO(s) + 5N_2_(g), (M = Zn, Cd)

Calculated enthalpies for reactants and products in their standard states were found to be –763 and –739 kJ mol^–1^ for *crs*-Zn(**pnz**)_2_ and *arh*-Cd(**pnz**)_2_, respectively, corresponding to energy densities of 3.71 and 2.93 kJ g^–1^ that are close to that of 1,3,5-trinitrotoluene (TNT, 4.6 kJ g^–1^).^88^

The enthalpies of formation (ESI Tables S12 and S13†) and combustion (ESI Tables S16 and S17†) were also calculated using the LDA functional. However, the LDA calculations revealed a large deviation from the enthalpies calculated using PBE. A tentative explanation of this difference might be in the known tendency of the LDA functional to overestimate bond dissociation energies,^89^ with GGA methods showing higher accuracy. We verified this by calculating bond dissociation energies for O_2_ and N_2_ molecules as well as atomization enthalpies for **pnz** frameworks (ESI Tables S18–S20†). While LDA calculations predicted higher dissociation energies compared to PBE in all cases, the comparison with experimentally determined dissociation energies for O_2_ and N_2_ molecules confirms the higher accuracy of the PBE functional (ESI Table S20†).^90^

Finally, we have also investigated the optical properties of pentazolate frameworks by calculating the electronic density of states (DOS) for the three lowest energy predicted structures of Zn(**pnz**)_2_ and Cd(**pnz**)_2_. The band gaps for all materials were in the range of 4.5–5.1 eV but, as the PBE functional generally underestimates the band gaps, the true values are likely to be even higher. These results suggest that these putative materials should be colorless. In addition, PDOS analysis confirmed that HOCO and LUCO bands in these structures are entirely localized on the **pnz** ligand orbitals, consistent with the d^10^ electronic configurations of Zn^2+^ and Cd^2+^ ions.

## Conclusions

In summary, we have utilized database mining and periodic DFT calculations to survey the structural landscapes of coordination frameworks based on the pentazolate ion, whose first stable compounds have just been reported.^1^,^4^,^5^ Whereas earlier work has considered the pentazolate anion as a ligand that would form discrete metallocene-like complexes,^37^,^38^ this computational screen is the first to investigate a different role: as a ligand in the formation of frameworks analogous to those of other azolates. The possibility of framework formation is expected not only by analogy to existing MAFs, but also based on the binding geometries of pentazolate ions in recently isolated complexes and salts,^1^,^4^,^5^ as well as by structures of two cobalt complexes of a related N_6_H_2_ ligand, which demonstrated a pentazole unit acting as a tetradentate ligand through four distinct nitrogen atoms (CSD codes UKEZIU, UKEZOA).^91^ The proposed metal pentazolate frameworks are of fundamental interest, as they structurally represent direct analogues of azolate metal–organic frameworks, while their formally inorganic nature would classify them together with other inorganic frameworks, such as zeolites. Considering the still highly exotic nature of the pentazolate ion, and the expected high energy content of these putative frameworks, this work illustrates how crystal structure modelling and database mining can be used to predict structures and properties of synthetically challenging new materials^40^,^92^–^98^ and paves the way for future high-throughput automated studies. The lowest-energy structure of zinc pentazolate is an open framework with a low packing coefficient, stabilized over alternative frameworks of greater density by the formation of additional Zn–N coordination bonds. For cadmium pentazolate, the lowest-energy structure is a framework of a novel topology, generated by cross-linking of two interpenetrated diamondoid nets. The calculated energy densities for lowest-energy zinc and cadmium pentazolate frameworks are comparable to those of known energetic compounds, and their calculated positive enthalpies of formation are smaller than those of several known explosives, indicating the frameworks should be chemically feasible.

## Conflicts of interest

There are no conflicts to declare.

## Supplementary Material

Crystal structure data

Supplementary information

Crystal structure data
