# Supplementary material for: Computational evaluation of metal pentazolate frameworks: inorganic analogues of azolate metal–organic frameworks
Source: Chem Sci. 2018 Feb 28;9(13):3367–75. doi: 10.1039/c7sc05020h (PMC5933226; doi:10.1039/c7sc05020h)
Supplement: Supplementary file 2 [file SC-009-C7SC05020H-s002.pdf]

Electronic Supplementary Material (ESI) for Chemical Science.
This journal is © The Royal Society of Chemistry 2018

Electronic Supplementary Information

Computational evaluation of metal pentazolate frameworks: inorganic analogues of azolate metal-organic frameworks

Mihails Arhangelskis,[a] Athanassios D. Katsenis,[a] Andrew J. Morris,[b] Tomislav Friščić*[a]

a) Department of Chemistry, McGill University, 801 Sherbrooke St. W. H3A 0B8 Montreal, Canada

a) School of Metallurgy and Materials, University of Birmingham, Edgbaston, Birmingham B15 2TT, UK

Contents

1. Methods 2

1.1 Crystal structure search 2

1.2 Periodic DFT calculations 3

1.2.1 Geometry optimization 3

1.2.2 Testing of alternative dispersion corrections and functionals 4

1.2.3 Density of states (DOS) analysis 4

1.2.4 Phonon calculations 4

1.3. Topological and geometrical analysis of calculated structures 4

2. Results 8

2.1 Calculated energies and topologies of pentazolate frameworks 8

2.2 Calculated reaction enthalpies 14

2.3 Calculated atomization enthalpies 20

2.4 Density of States (DOS) analysis 21

2.5 Phonon calculations 24

2.6 Analysis of π-π interactions in pentazolate framework structures 46

3. Crystal structures of pentazolate frameworks in CIF format 47

1. Methods

1.1 Crystal structure search

The set of crystal structures used in our study was compiled by searching the Cambridge Structural Database (CSD v. 1.18) for all MAF-type crystal structures containing azolate-type ligands (pyrazolate, imidazolate, 1,2,3-triazolate, 1,2,4-triazolate and tetrazolate) coordinated to transition metals. Following this search the structures were manually evaluated to remove all mononuclear complexes and one-dimensional (1D) chain structures, as well as structures containing metals with oxidation states other than +2. Finally, we have removed all duplicate entries as well as isomorphous structures containing different metal atoms (*e.g.* the ZIF-8 structure OFERUN03 and its cobalt analogue GITTOT). In the cases of isomorphous structures preference was given to a structure containing zinc as the metal node or, if a zinc-containing structure was not available, another 3*d*-element.

The search resulted in 39 distinct crystal structures of metal(II) MAFs (Table S1). In preparation for periodic DFT calculations the structures were converted to Zn(**pnz**)$_2$ form using the following procedure:

1. Any guest molecules/ions were removed from the voids, leaving only the framework structures.
2. All azolate side groups were removed, leaving only 5-membered azolate rings coordinated to metal centers.
3. The metal centers were replaced with Zn atoms, while all carbons in the azolate rings were replaced with nitrogens.
4. In the case of disordered structures, the minor disorder components were deleted and the major component was assigned full occupancy.
5. For the structures where disorder was defined with space group symmetry, structures were manipulated into the corresponding lower symmetry space groups.

Table S1. Details of CSD structures used for generating hypothetical **pnz** frameworks.

CSD CODE	Chemical formula	Systematic chemical name
AXIVAF	$(C_8H_{14}N_8Zn)_n$	catena-(bis(μ$_2$-5-isopropyltetrazolato)-zinc)
BISPAU	$(C_{24}H_{16}N_{12}Zn_2)_n$	catena(tetrakis(benzotriazolato)-di-zinc(II))
BOJXAZ	$(C_2HgN_{10}O_4)_n$	catena-(bis(μ$_2$-5-nitro-1,2,3,4-tetrathiazole-N1,N3)-mercury)
CAGLIF	$(C_8H_{10}FeN_4)_n$	bis(μ$_2$-2-methylimidazolato-N,N')-iron(II)
CAYBAH	$(C_{12}H_{12}Cu_3N_{18})_n$	catena-[hexakis(μ$_3$-1,2,3-triazolato)-tri-copper(II)]
CAYSEB	$(C_{12}H_8N_6Zn)_n$	catena-(bis(μ$_2$-benzotriazole)-zinc(II))
CUIMDZ02	$(C_{30}H_{30}Cu_5N_{20})_n$	catena-(decakis(μ$_2$-imidazolato-N,N')-penta-copper(II))
CUIMDZ03	$(C_{18}H_{18}Cu_3N_{12})_n$	catena-(hexakis(μ$_2$-imidazolato-N,N')-tri-copper(II))
EHETER02	$(C_{10}H_{14}N_4Zn)_n$	catena-(bis(μ$_2$-2-ethylimidazolato)-zinc)
EQOBUH	$(C_{12}H_{12}Co_2N_8)_n \cdot n\ C_5H_5N$	catena-(tetrakis(μ$_2$-imidazol-1,3-diyl)-di-cobalt(II) pyridine clathrate)
EQOCOC01	$(C_{12}H_{12}N_8Zn_2)_n \cdot n\ H_2O$	catena-(tetrakis(μ$_2$-imidazolato-N,N')-di-zinc(II) unknown clathrate monohydrate)
GITTAF	$(C_{14}H_{16}N_8Zn_2)_n$	catena-(bis(μ$_2$-imidazolato-N,N')-bis(μ$_2$-2-methylimidazolato-N,N')-di-zinc(II) unidentified solvent clathrate)
GITTEJ	$(C_{24}H_{24}N_{16}Zn_4)_n \cdot 2.24n\ C_3H_7NO$	catena-(octakis(μ$_2$-imidazolato-N,N')-tetra-zinc(II) dimethylformamide clathrate)
GUPBOJ	$(C_{24}H_{30}Cd_3N_{12})_n$	catena-(hexakis(μ$_2$-2-methylimidazolato-N,N')-tri-cadmium)
GUPBOJ01	$(C_{48}H_{60}Cd_6N_{24})_n$	catena-(dodecakis(μ$_2$-2-methylimidazolato)-hexa-cadmium)
GUPCAW	$(C_{32}H_{40}Cd_4N_{16})_n \cdot n\ H_2O$	catena-(octakis(μ$_2$-2-methylimidazolato-N,N')-tetra-cadmium monohydrate)
HIFWAV	$(C_{30}H_{30}N_{20}Zn_5)_n \cdot n\ C_5H_{11}NO$	catena-(decakis(μ$_2$-imidazolato-N,N')-penta-zinc(II) N,N-diethylformamide clathrate)
HOKMUR	$(C_{16}H_{24}N_{32}Zn_4)_n$	catena-[octakis(μ-5-methyltetrazolato)-tetra-zinc(II) solvate]
IMIDZB01	$(C_{12}H_{12}N_8Zn_2)_n$	catena-(tetrakis(μ$_2$-imidazolyl)-di-zinc(II))
IMIDZB07	$(C_{24}H_{24}N_{16}Zn_4)_n$	catena-[octakis(μ$_2$-imidazolato-N,N')-tetra-zinc(II)]
IMIDZB11	$(C_{12}H_{12}N_8Zn_2)_n$	catena-[tetrakis(μ-imidazolato)-di-zinc]
KEYSEO	$(C_8H_{12}N_6Zn)_n$	catena-(bis(μ$_2$-3-ethyl-1,2,4-triazolato)-zinc)
LIHQUP	$(C_4H_6N_6Zn)_n$	catena-(bis(μ$_2$-1,2,4-triazole)-zinc(II))
MECWOH	$(C_{20}H_{28}N_8Zn_2)_n \cdot 3n\ H_2O$	catena-(tetrakis(μ$_2$-2-ethylimidazolato)-zinc(II) trihydrate clathrate)
OFERUN01	$(C_{16}H_{20}N_8Zn_2)_n$	catena-[tetrakis(μ$_2$-2-methylimidazolato-N,N')-di-zinc(II)]
OFERUN03	$(C_{16}H_{20}N_8Zn_2)_n$	catena-(tetrakis(μ$_2$-2-methylimidazolato)-di-zinc)

OFERUN08	$(C_{64}H_{80}N_{32}Zn_8)_n$	catena-[hexadecakis(μ-2-methylimidazolato)-octa-zinc]
ONATUT	$(C_4H_4N_8Zn)_n$	catena-[(μ$_4$-1,2-bis(tetrazol-5-yl)ethane-N1,N'1,N2,N4,N'4)-zinc(II)]
PAJRUQ	$(C_{36}H_{36}N_{24}Zn_6)_n$	catena-(dodecakis(μ-imidazolato)-hexa-zinc unknown solvate)
SIVGEL	$(C_8H_{12}N_{16}Zn_2)_n \cdot 2n\ CH_4O$	catena-[tetrakis(μ$_2$-5-methyltetrazol-1-yl)-di-zinc methanol solvate]
SIVGOV	$(C_4H_6N_8Zn)_n \cdot n\ C_5H_9NO$	catena-[bis(μ$_2$-5-methyltetrazol-1-yl)-zinc 1-methylpyrrolidin-2-one]
TOHDEB	$(C_{22}H_{18}Cl_2N_8Zn_2)_n, \cdot 0.25n\ H_2O$	catena-[bis(μ$_2$-5-chlorobenzimidazolyl)-bis(μ2-2-methylimidazolyl)-di-zinc hydrate]
VEJYEP	$(C_{12}H_{12}N_8Zn_2)_n \cdot n\ C_4H_9NO$	catena-(tetrakis(μ$_2$-imidazolato-N,N')-di-zinc(II) N,N-dimethylacetamide clathrate)
VEJYIT	$(C_{12}H_{12}N_8Zn_2)_n \cdot 3n\ C_3H_7NO$	catena-(tetrakis(μ$_2$-imidazolato-N,N')-di-zinc(II) tris(N,N-dimethylformamide) clathrate)
VEJYOZ	$(C_{12}H_{12}N_8Zn_2)_n \cdot 6n\ H_2O$	catena-(tetrakis(μ$_2$-imidazolato-N,N')-di-zinc(II) clathrate hexahydrate)
VEJZIU	$(C_{12}H_{12}N_8Zn_2)_n \cdot 1.38n\ H_2O$	catena-(tetrakis(μ$_2$-imidazolato-N,N')-di-zinc(II) clathrate hydrate)
WAQQUB	$(C_{12}H_{18}N_{24}Zn_3)_n \cdot n\ H_2O$	catena-(hexakis(μ$_3$-5-methyltetrazolato-N,N',N'')-tri-zinc(II) monohydrate)
WAQRAI	$(C_2H_2N_8Zn)_n$	catena-(bis(μ$_2$-tetrazolato-N,N''')-zinc(II))
YOMBOS	$(C_{36}H_{24}N_{18}Zn_3)_n$	catena-[hexakis(μ$_2$-benzotriazolyl)-tri-zinc]

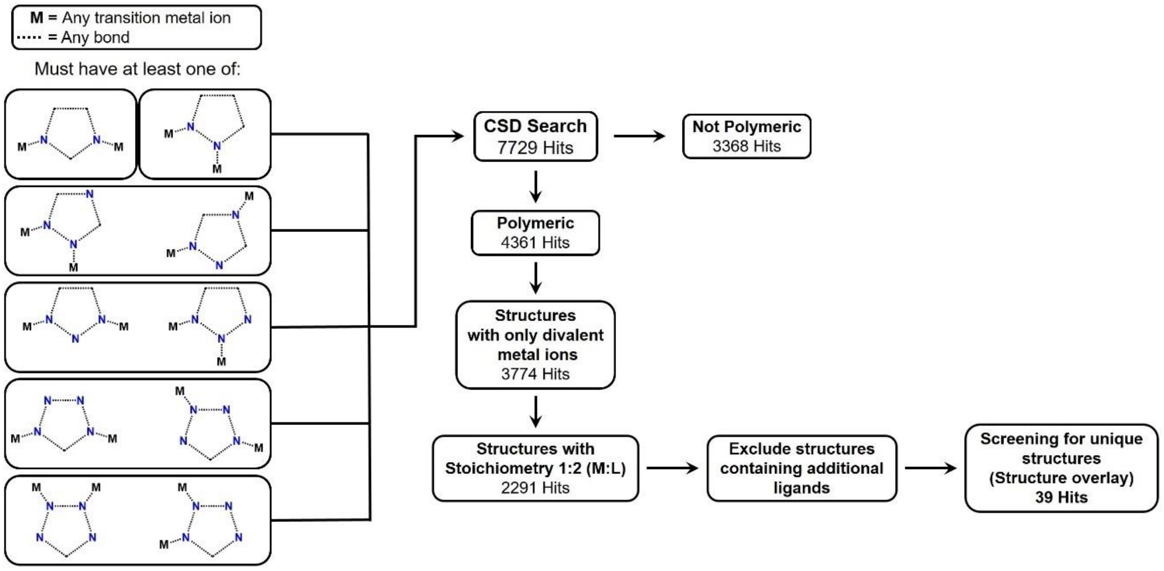

Figure S1. Diagram illustrating the CSD structure selection process.

1.2 Periodic DFT calculations

Periodic DFT calculations were performed using the plane-wave DFT program CASTEP 16.11. CASTEP input files were prepared with the aid of the program CIF2Cell. Structures with primitive cells were processed using their original space group symmetry, while structures having C-, I-, F- or R- symmetry were converted into the equivalent primitive setting for the purpose of reducing the volume of the simulation cell and speeding up the DFT calculation. The transformation into P-type lattice is mathematically exact, therefore the resulting structures are completely equivalent to the structures in conventional space group settings.

1.2.1 Geometry optimization

The geometry optimization procedure involved full relaxation of atom coordinates and unit cell parameters subject to the space group symmetry constraints. The calculations were performed with PBE GGA-type functional combined with Grimme D2 dispersion correction. The plane-wave cut-off was set to 750 eV and the norm-conserving pseudopotentials were used. The Brillouin zone was sampled with a 0.03 $Å^{-1}$ k-point grid. The following convergence criteria were applied for geometry optimization: maximum energy change 10^{-5} eV/atom, maximum force on atom 0.03 eV/Å, maximum atom displacement 0.001 Å and residual stress 0.05 GPa.

Putative Zn(**pnz**)$_2$ structures were optimized first. The optimized structures were then used as a starting point for Cd(**pnz**)$_2$, replacing the zinc nodes with cadmium. One structure that required special treatment compared to the standard procedure described above was the one with the CCDC code AXIVAF. This structure is a 2-dimensional (2D) layered framework with the formula Zn(5-isopropyltetrazolate)$_2$. Removal of the bulky *iso*-propyl groups resulted in a highly porous Zn(**pnz**)$_2$ structure with essentially non-interacting 2D layers (interlayer separation over 10 Å). The geometry optimization procedure did not

rectify this chemically unrealistic arrangement, resulting in a high energy structure. At this point it was deemed necessary to modify the starting AXIVAF structure by manually shrinking the unit cell in a way that would bring the layers to a more realistic separation distance. Following this modification geometry optimization was performed, resulting in a denser and lower energy structure. This was the only 2D layered structure with highly separated layers. Other frameworks were optimized starting from the atom coordinates found in CSD experimental structures.

Besides the **pnz** frameworks, we have calculated energies for the mixed **pnz** salt reported by Zhang *et al.*, as well as for the structures of Zn and Cd metals, crystalline ZnO and CdO, as well as gaseous O_2, N_2, H_2, Cl_2 and H_2O. Calculation of enthalpies for molecules in the gas phase was performed by placing the molecules in a cubic box of 30×30×30 $Å^3$ dimensions and performing geometry optimization while keeping the box size fixed. Convergence testing with respect to the box size was performed and it was found that this was a sufficient size to make intermolecular interactions between periodic images negligible. The calculated energies for the **pnz** frameworks, metals, metal oxides and compounds in the gas phase were used to compute reaction enthalpies for the formation and combustion reactions of the **pnz** frameworks.

1.2.2 Testing of alternative dispersion corrections and functionals

A subset of structures of Zn(**pnz**)$_2$ (WAQQUB, LIHQUP, IMIDZB01, IMIDZB07, ONATUT, GUPBOJ, CUIMDZ03, GUPBOJ01, HIFWAV and GITTEJ) and Cd(**pnz**)$_2$. (WAQRAI, WAQQUB, AXIVAF, LIHQUP, CUIMDZ02, ONATUT, BOJXAZ, CUIMDZ03, IMIDZB01 and IMIDZB07) were used for evaluating the effect of using different dispersion correction approaches and a different DFT functional on the energy ranking. The structures were geometry optimized using PBE+MBD*, PBE and LDA methods. The lattice energies and volumes obtained by these methods were compared with the PBE+D2 results.

1.2.3 Density of states (DOS) analysis

Density of states (DOS) calculations were performed for the three lowest energy topologically distinct structures of Zn(**pnz**)$_2$ (WAQQUB, LIHQUP, IMIDZB01) and Cd(**pnz**)$_2$ (WAQRAI, WAQQUB, AXIVAF). The optical calculations used PBE functional with 750 eV plane wave cutoff. The electronic k-point grid spacing was set to 0.03 $Å^{-1}$, while a denser grid of 0.015 $Å^{-1}$ was used to calculate DOS plots. Besides the full DOS plots, projection onto Zn/Cd and N atoms were calculated.

1.2.4 Phonon calculations

Phonon calculations were performed for selected lowest energy topologically distinct structures of Zn(**pnz**)$_2$ (WAQQUB, LIHQUP) and Cd(**pnz**)$_2$ (WAQRAI, WAQQUB, AXIVAF). In preparation for the phonon calculations the structures were optimized with a tighter force convergence criterion of 0.01 eV/Å. Phonons were calculated using linear response method, the standard grid scale and the fine grid scale were set to 2 and 3, respectively.

1.3. Topological and geometrical analysis of calculated structures

The optimized structures of **pnz** frameworks were used for topology analysis with the software ToposPro. Structures which originally had P-type symmetry were used directly as they were generated in the CASTEP output. The structures which originally possessed symmetry other than primitive and were transformed to non-conventional symmetry settings (see section 1.2) were represented in the *P*1 space group for the purpose of topological analysis.

Extensive CSD analysis was performed to determine the distribution of Zn-N and Cd-N bond lengths in metal-azolate frameworks and complexes. Based on these distributions (Figures S1 and S2) the bond length cut-offs were set to 2.20 Å and 2.50 Å for Zn-N and Cd-N bonds, respectively. These CSD-based cut-offs were used to classify the interatomic interactions for the purpose of topological analysis. In order to further validate the geometrical classification of Zn/Cd-N bonds we have determined the bond orders with the aid of Mulliken population analysis (Figures S3-S4).

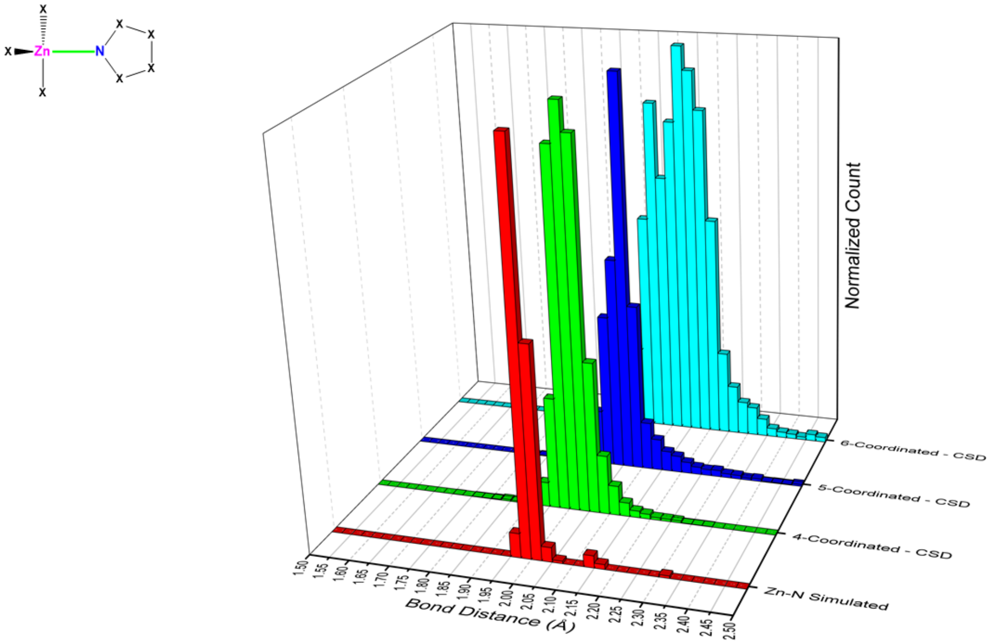

Figure S2. Histograms showing Zn-N bond length distributions in the CSD (turquoise: 6-coordinate zinc, blue: 5-coordinate zinc, green: 4-coordinate zinc). The red histogram is based on the simulated structures of Cd(**pnz**)$_2$ frameworks. The diagrams indicate that 2.20 Å is a suitable cut-off for Zn-N bond length distance.

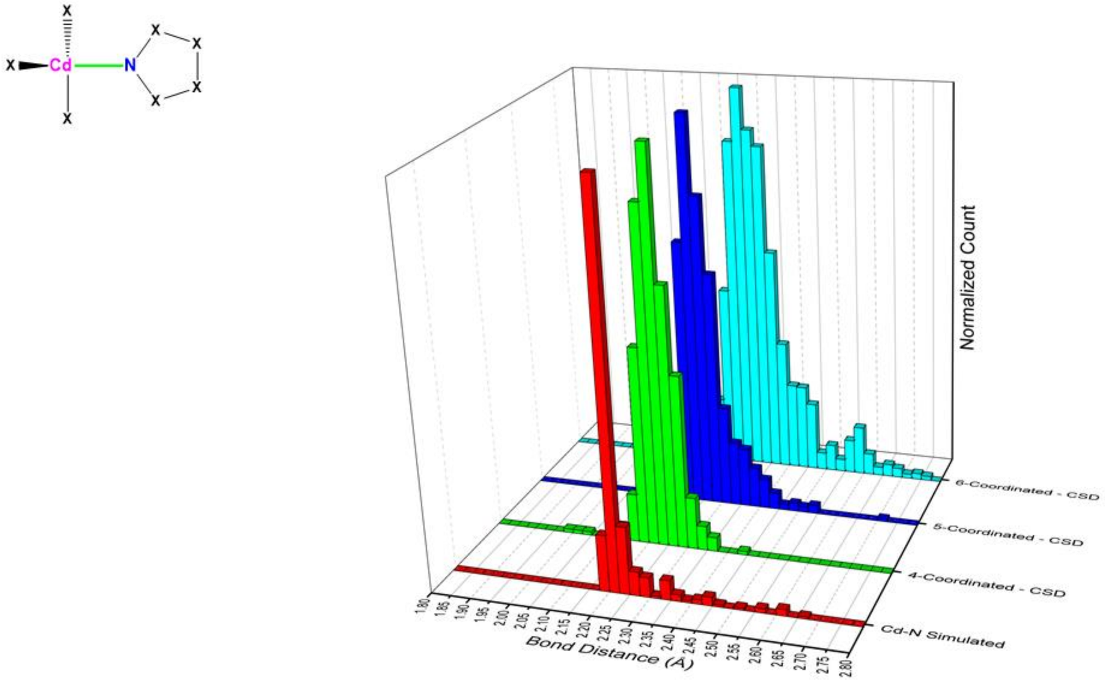

Figure S3. Histograms showing Cd-N bond length distributions in the CSD (turquoise: 6-coordinate cadmium, blue: 5-coordinate cadmium, green: 4-coordinate cadmium). The red histogram is based on the simulated structures of Cd(**pnz**)$_2$ frameworks. The diagrams indicate that 2.50 Å is a suitable cut-off for Zn-Cd bond length distances.

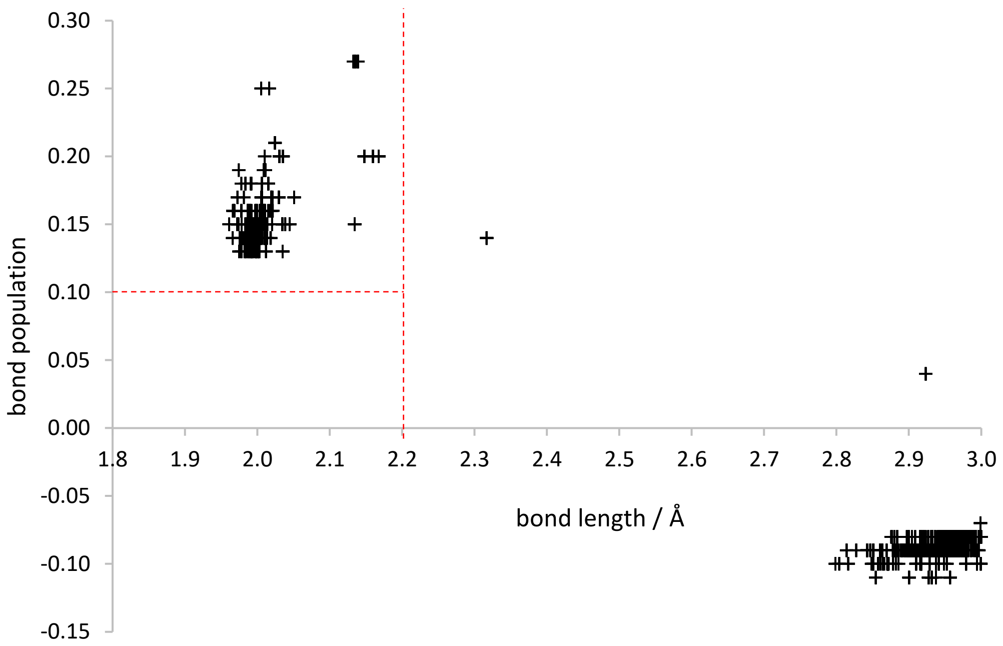

Figure S4. Distribution of Mulliken bond populations as a function of metal-ligand bond length distances for Zn(**pnz**)$_2$ structures. The majority of bonding contacts (population >0.1) fall within 2.2 Å cut-off, consistent with the bond length distributions established by a CSD search. Red lines outline the area in the interatomic distance *vs* bond population plot that defines chemical bonds.

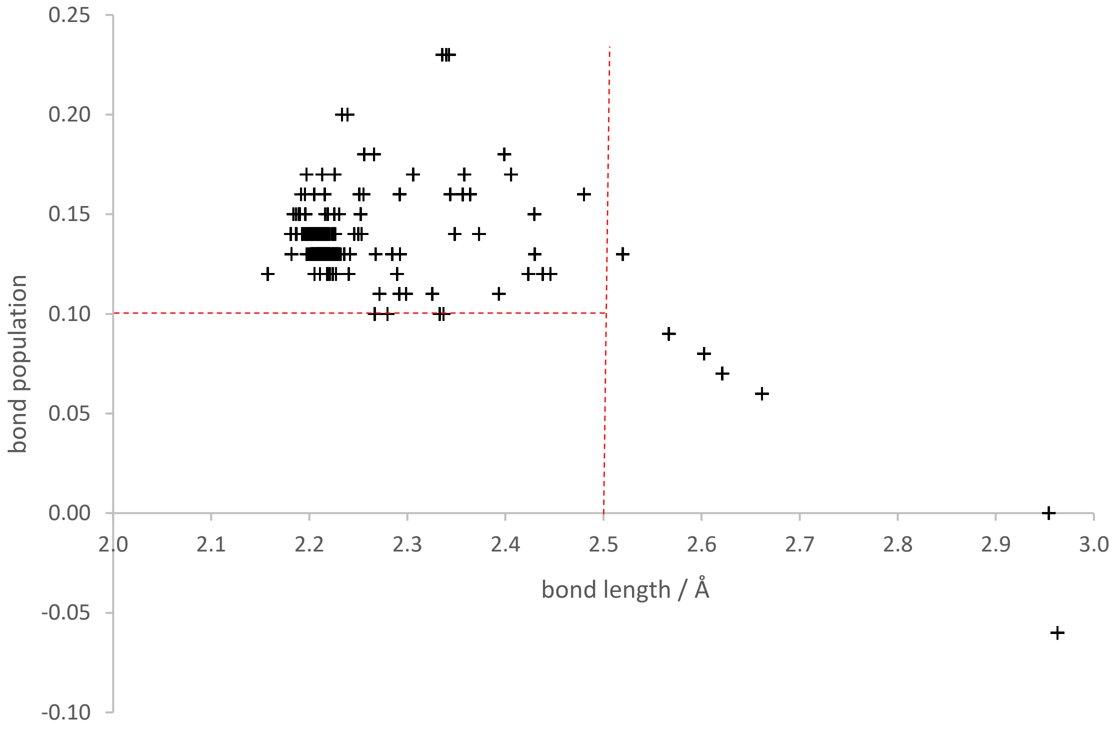

Figure S5. Distribution of Mulliken bond populations as a function of bond length distances for Cd(**pnz**)$_2$ structures. The majority of bonding contacts (population > 0.1) fall within 2.5 Å cut-off, consistent with the bond length distributions established by a CSD search. Red lines outline the area in the interatomic distance *vs* bond population plot that defines chemical bonds.

Table S2. Mulliken atom charges for selected structures of Zn(**pnz**)$_2$. The nitrogens coordinated to zinc are more negatively charged than the non-coordinated atoms.

CSD CODE	Mulliken charges		
	**Zn**	**N coordinated to Zn**	**non-coordinated N**
WAQQUB	1.33; 1.32	-0.17	-0.06
LIHQUP	1.43	-0.24 to -0.23	-0.10 to -0.06
IMIDZB01	1.40; 1.43	-0.25 to -0.22	-0.09 to -0.07
IMIDZB07	1.41; 1.43	-0.25 to -0.23	-0.09 to -0.07
ONATUT	1.38	-0.23 to -0.17	-0.14 to -0.05
GUPBOJ	1.39 to 1.43	-0.25 to -0.23	-0.09 to -0.06
CUIMDZ03	1.40; 1.44	-0.25 to -0.20	-0.09 to -0.07
GUPBOJ01	1.43 to 1.45	-0.26 to -0.24	-0.08 to -0.06
HIFWAV	1.44 to 1.45	-0.26 to -0.24	-0.08 to -0.06
GITTEJ	1.44 to 1.46	-0.26 to -0.25	-0.09 to -0.07

Table S3. Mulliken atom charges for selected structures of Cd(**pnz**)$_2$. The nitrogens coordinated to cadmium are more negatively charged than the non-coordinated atoms.

CSD CODE	Mulliken charges		
	**Cd**	**N coordinated to Cd**	**non coordinated N**
WAQRAI	1.36	-0.18 to -0.16	-0.09 to -0.08
WAQQUB	1.24; 1.30	-0.18 to -0.17	-0.06
AXIVAF	1.33	-0.18 to -0.15	-0.08 to -0.07
LIHQUP	1.38	-0.21 to -0.19	-0.14 to -0.08
CUIMDZ02	1.34; 1.38	-0.19 to -0.17	-0.15 to -0.05
ONATUT	1.38	-0.22 to -0.15	-0.10 to -0.06
BOJXAZ	1.36	-0.22 to -0.18	-0.15 to -0.06
CUIMDZ03	1.37	-0.22 to -0.16	-0.15 to -0.06
IMIDZB01	1.37; 1.39	-0.22 to -0.15	-0.12 to -0.06
IMIDZB07	1.31 to 1.39	-0.22 to -0.14	-0.13 to -0.06

2. Results

2.1 Calculated energies and topologies of pentazolate frameworks

Table S4. Topologies, relative lattice energies and packing coefficients for the Zn(**pnz**)$_2$ structures.

CSD CODE	topology	$E_{\text{formula unit}}$ / eV	E_{rel} / kJ mol^{-1}	packing coefficient
WAQQUB	*crs*	-4107.16	0.000	0.454
CAYBAH	*crs*	-4107.15	0.085	0.459
LIHQUP	*dia*-interpenetrated	-4107.02	12.989	0.662
WAQRAI	*dia*-interpenetrated	-4107.01	14.536	0.669
IMIDZB01	*zni*	-4106.94	20.887	0.555
IMIDZB07	*coi*	-4106.92	23.044	0.548
ONATUT	*sql*	-4106.89	25.249	0.615
BOJXAZ	*sql*	-4106.89	25.625	0.624
AXIVAF	*sql*	-4106.86	28.619	0.573
GUPBOJ	*yqt*	-4106.84	30.472	0.519
GITTAF	*zni*	-4106.79	35.637	0.500
CUIMDZ03	*mog*	-4106.77	37.404	0.532
GUPBOJ01	*ict*	-4106.76	37.730	0.437
HIFWAV	*nog*	-4106.75	39.188	0.369
GITTEJ	*crb*	-4106.73	40.612	0.397
VEJYEP	*crb*	-4106.72	41.674	0.386
CUIMDZ02	*4,4L37*	-4106.7	43.503	0.621
IMIDZB11	*cag*	-4106.7	43.782	0.387
OFERUN08	*kat*	-4106.69	44.532	0.416
EQOBUH	*neb*	-4106.69	44.591	0.41
OFERUN01	*dia*	-4106.69	45.298	0.44
SIVGEL	*lon*	-4106.68	45.539	0.321
OFERUN03	*SOD*	-4106.68	46.078	0.314
CAYSEB	*hcb*	-4106.68	46.152	0.462
PAJRUQ	*CAN*	-4106.68	46.207	0.263
VEJYOZ	*DFT*	-4106.67	46.609	0.281
HOKMUR	*dia*	-4106.67	46.735	0.322
YOMBOS	*BIK*	-4106.67	46.819	0.311
VEJZIU	*MER*	-4106.67	46.876	0.241
SIVGOV	*dia*	-4106.67	47.062	0.32
GUPCAW	*MER*	-4106.66	47.437	0.241
KEYSEO	*dia*	-4106.66	47.488	0.411
EQOCOC01	*GIS*	-4106.64	50.014	0.306
VEJYIT	*crb*	-4106.63	50.507	0.362
TOHDEB	*CHA*	-4106.63	50.670	0.272
BISPAU	*sra*	-4106.63	50.848	0.323
EHETER02	*ANA*	-4106.62	51.978	0.281
CAGLIF	*qtz*	-4106.6	53.880	0.376
MECWOH	*RHO*	-4106.55	58.342	0.244

Table S5. Topologies, relative lattice energies and packing coefficients for the Cd(**pnz**)$_2$ structures.

CSD CODE	topology or net point symbol	$E_{formula\ unit}$ / eV	E_{rel} / kJ mol^{-1}	packing coefficient
WAQRAI	*arh*	-3914.51	0.000	0.647
WAQQUB	*crs*	-3914.40	9.905	0.396
CAYBAH	*crs*	-3914.40	9.968	0.400
AXIVAF	*bcu*	-3914.34	16.564	0.587
LIHQUP	*dia*	-3914.32	17.783	0.431
CUIMDZ02	*4,4L37*	-3914.19	30.317	0.692
ONATUT	*seh-3,5-Pbca*	-3914.19	30.739	0.540
BOJXAZ	*sql*	-3914.18	31.159	0.588
IMIDZB07	$\{4.5.7^2.8^2\}\{4.5.7^4.8^4\}\{4^2.5.6.7.8\}\{4^2.5.6.7^2\}\{5.7^2\}$	-3914.16	33.756	0.547
CUIMDZ03	$\{4.6^2\}_2\{4.6^9\}_2\{6^6\}$	-3914.10	39.198	0.558
IMIDZB01	*zni*	-3914.09	39.921	0.506
GUPBOJ	*yqt*	-3913.95	53.823	0.463
GUPBOJ01	*ict*	-3913.87	61.356	0.388
GITTAF	*zni*	-3913.84	64.518	0.428
HIFWAV	*nog*	-3913.81	66.956	0.318
GITTEJ	*crb*	-3913.80	67.764	0.356
VEJYEP	*crb*	-3913.77	70.608	0.332
OFERUN01	*dia*	-3913.76	71.621	0.383
EQOBUH	*neb*	-3913.76	72.106	0.354
CAYSEB	*hcb*	-3913.76	72.159	0.409
OFERUN08	*kat*	-3913.76	72.299	0.355
IMIDZB11	*cag*	-3913.75	72.747	0.330
SIVGEL	*lon*	-3913.74	73.688	0.275
KEYSEO	*dia*	-3913.74	74.105	0.356
YOMBOS	*BIK*	-3913.74	74.301	0.266
SIVGOV	*dia*	-3913.74	74.363	0.273
HOKMUR	*dia*	-3913.74	74.392	0.275
OFERUN03	*SOD*	-3913.73	74.651	0.223
PAJRUQ	*CAN*	-3913.73	74.654	0.224
VEJZIU	*MER*	-3913.73	74.849	0.207
VEJYOZ	*DFT*	-3913.73	74.939	0.240
GUPCAW	*MER*	-3913.73	75.220	0.206
BISPAU	*sra*	-3913.71	76.564	0.277
EQOCOC01	*GIS*	-4106.63	76.900	0.209
TOHDEB	*CHA*	-4106.63	77.092	0.292
VEJYIT	*crb*	-4106.63	77.149	0.259
EHETER02	*ANA*	-4106.62	78.193	0.285
CAGLIF	*qtz*	-4106.6	79.008	0.322
MECWOH	*RHO*	-4106.55	82.551	0.176

Table S6. Comparison of relative lattice energies of selected Zn(**pnz**)$_2$ structures calculated with PBE+D2, PBE+TS PBE+MBD*, PBE and LDA methods. The energies are shown relative to LIHQUP structure (set to zero).

Structure	Relative energy per formula unit / kJ mol^{-1}					
	**PBE+D2**	**PBE+TS**	**PBE+MBD* @ PBE+D2 geometry**	**PBE+MBD* @ PBE+TS geometry**	**LDA**	**PBE**
WAQQUB	-12.989	4.972	-5.289	-5.804	-3.050	-8.603
LIHQUP	0.000	0.000	0.000	0.000	0.000	0.000
IMIDZB01	7.899	10.674	7.398	7.176	14.707	-4.955
IMIDZB07	10.056	10.490	8.027	7.750	14.866	-4.906
ONATUT	12.260	20.802	15.184	14.967	18.075	11.845
GUPBOJ	17.483	19.450	15.490	15.827	22.828	3.196
CUIMDZ03	24.415	29.647	24.141	24.155	26.055	4.665
GUPBOJ01	24.741	27.126	20.778	20.536	33.529	0.697
HIFWAV	26.199	28.468	21.625	21.324	32.297	-1.769
GITTEJ	27.623	30.143	23.327	22.971	33.671	-0.107

Table S7. Comparison of relative lattice energies of selected Cd(**pnz**)$_2$ structures calculated with PBE+D2, PBE+TS, PBE+MBD*, PBE and LDA methods. The energies are shown relative to LIHQUP structure (set to zero).

Structure	Relative energy per formula unit / kJ mol^{-1}					
	**PBE+D2**	**PBE+TS**	**PBE+MBD* @ PBE+D2 geometry**	**PBE+MBD* @ PBE+TS geometry**	**LDA**	**PBE**
WAQRAI	-17.783	-13.070	-15.124	-15.065	-23.451	-5.296
WAQQUB	-7.878	-2.917	-5.800	-5.693	-10.591	-10.284
AXIVAF	-1.219	6.705	3.045	2.922	-1.872	6.518
LIHQUP	0.000	0.000	0.000	0.000	0.000	0.000
CUIMDZ02	12.533	18.161	17.864	17.969	10.741	28.005
ONATUT	12.956	15.916	12.676	12.639	11.563	8.205
BOJXAZ	13.375	15.995	13.605	13.569	12.541	11.704
IMIDZB07	15.973	17.632	16.690	16.711	16.459	11.010
CUIMDZ03	21.414	25.497	23.908	24.364	21.281	11.033
IMIDZB01	22.137	21.481	21.042	21.495	22.354	10.931

Table S8. Comparison of volumes per formula unit for Zn(**pnz**)$_2$ structures calculated with PBE+D2, PBE+TS, PBE+MBD*, PBE and LDA methods.

Structure	Volume per formula unit /Å^3			
	**PBE+D2**	**PBE+TS**	**LDA**	**PBE**
WAQQUB	221.785	225.356	213.8259	226.893
LIHQUP	154.454	156.112	142.5473	162.230
IMIDZB01	183.488	184.298	172.8407	197.760
IMIDZB07	184.896	185.032	167.9354	195.749
ONATUT	163.876	164.920	155.8985	174.936
GUPBOJ	194.988	194.413	182.9708	204.084
CUIMDZ03	189.408	193.810	175.3036	258.153
GUPBOJ01	229.588	229.711	218.0873	235.874
HIFWAV	271.110	270.431	255.1656	276.144
GITTEJ	254.119	253.907	233.6717	260.512

Table S9. Comparison of volumes per formula unit for Cd(**pnz**)$_2$ structures calculated with PBE+D2, PBE+TS, PBE+MBD*, PBE and LDA methods.

Structure	Volume per formula unit /Å^3			
	PBE+D2	PBE+TS	LDA	PBE
WAQRAI	165.363	167.598	159.220	171.212
WAQQUB	263.446	270.189	256.214	275.999
AXIVAF	181.884	185.625	175.200	187.595
LIHQUP	169.805	172.509	164.130	185.469
CUIMDZ02	154.884	157.056	145.796	177.493
ONATUT	195.515	197.749	185.886	202.117
BOJXAZ	181.523	182.515	173.101	191.939
IMIDZB07	195.127	197.237	186.936	211.277
CUIMDZ03	189.947	195.277	179.492	262.494
IMIDZB01	211.394	211.700	199.122	219.440

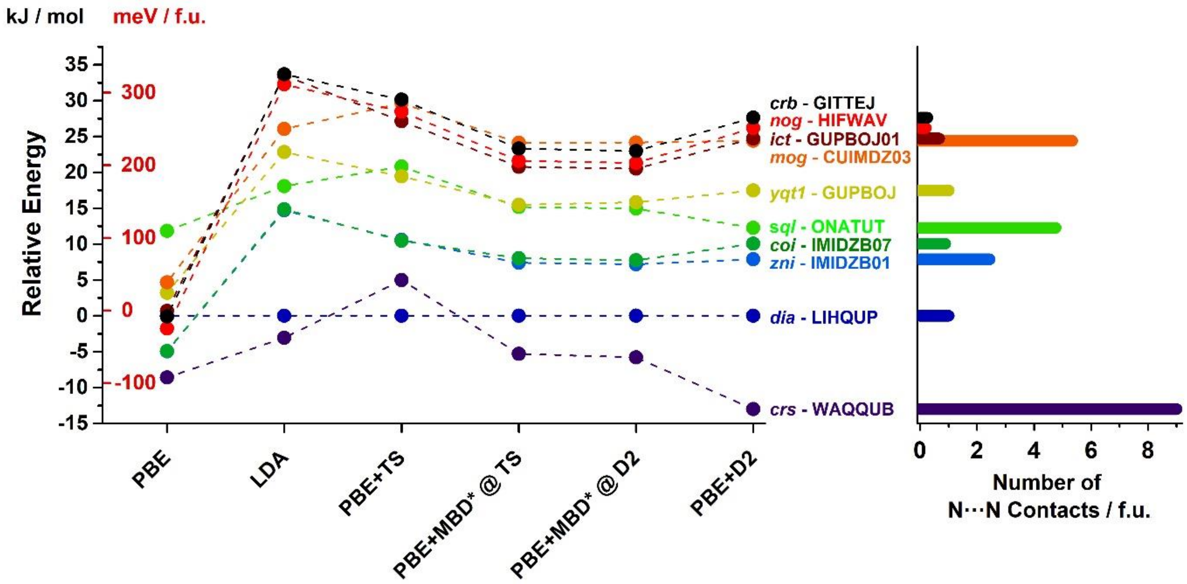

Figure S6. Data from Table S6. Comparison of relative lattice energies of selected Zn(**pnz**)$_2$ structures calculated with PBE+D2, PBE+MBD* (single point with PBE+TS and PBE+D2 geometries), PBE and LDA methods. The energies are shown relative to LIHQUP structure (set to zero). The bars on the right are showing the number of short (3.0-3.1 Å) N···N contacts present in each structure.

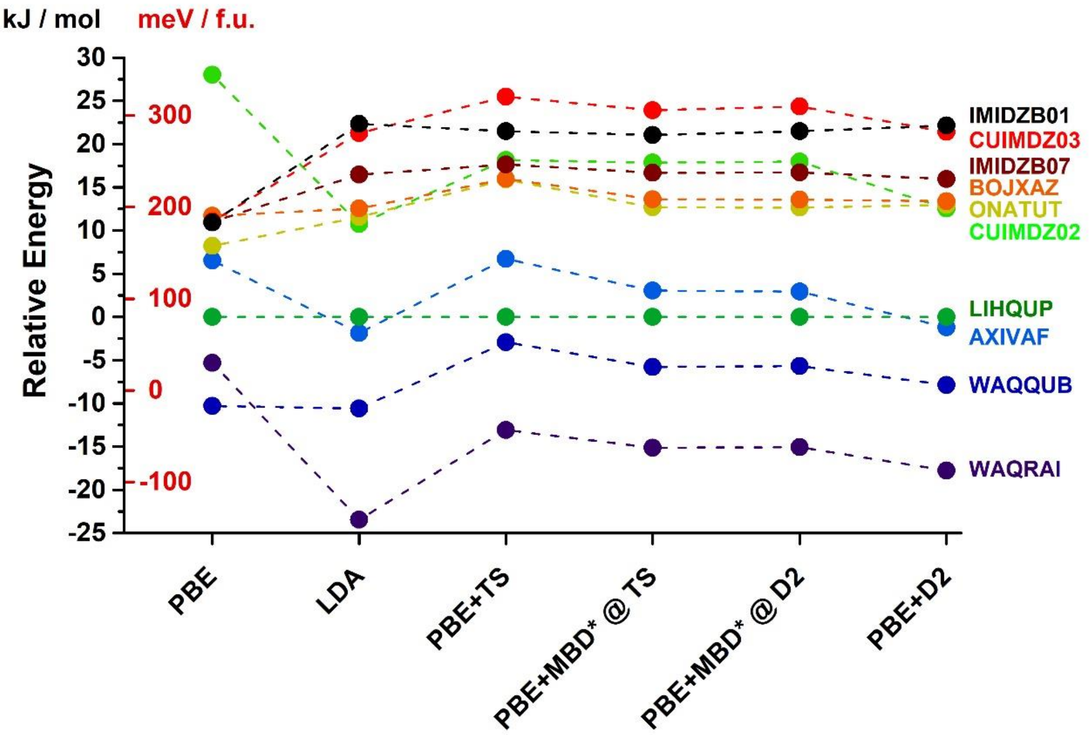

Figure S7. Data from Table S7. Comparison of relative lattice energies of selected Cd(**pnz**)$_2$ structures calculated with PBE+D2, PBE+MBD* (single point with PBE+TS and PBE+D2 geometries), PBE and LDA methods. The energies are shown relative to LIHQUP structure (set to zero).

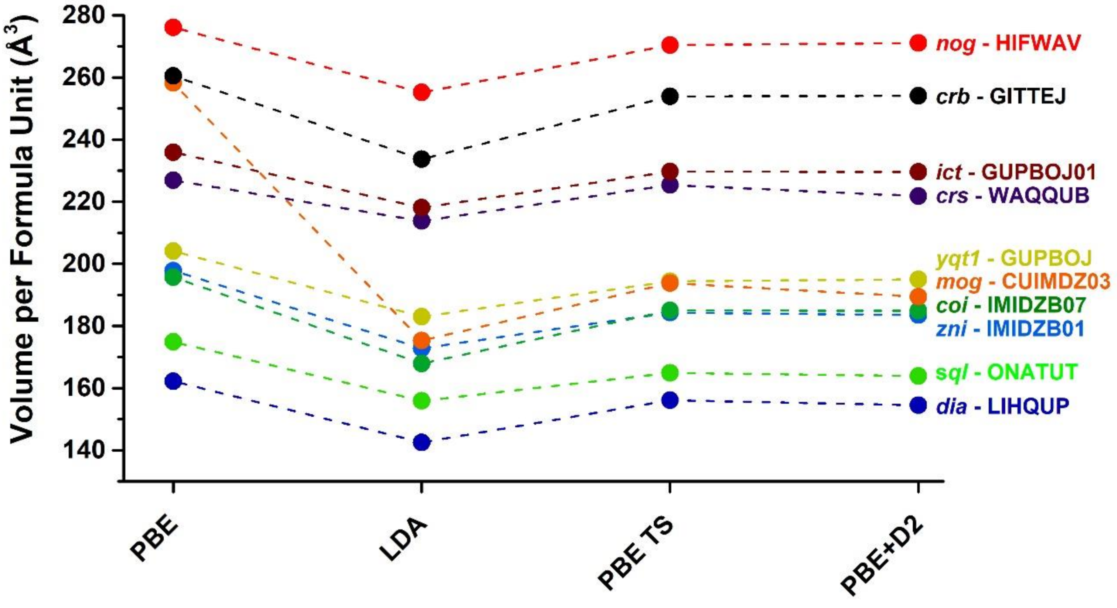

Figure S8. Data from Table S8. Comparison of volumes per formula unit for Zn(**pnz**)$_2$ structures calculated with PBE+D2, PBE+TS, PBE and LDA methods.

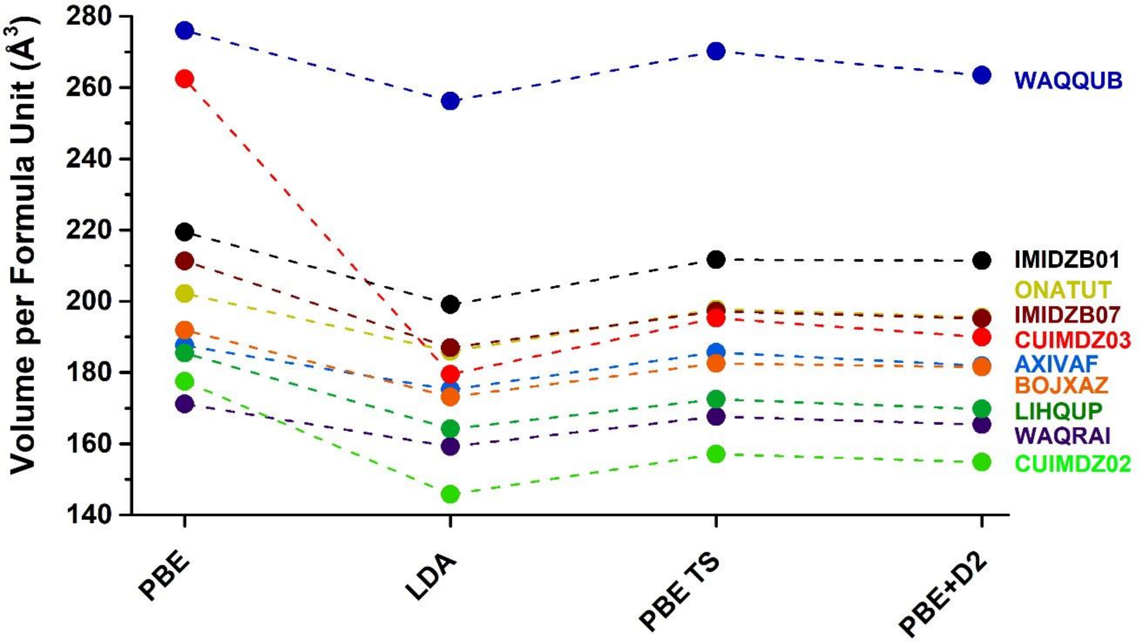

Figure S9. Data from Table S9. Comparison of volumes per formula unit for Cd(**pnz**)$_2$ structures calculated with PBE+D2, PBE+TS, PBE and LDA methods.

2.2 Calculated reaction enthalpies

Table S10. Calculated enthalpy of formation for Zn(**pnz**)$_2$.
$Zn(s) + 5\ N_2(g) \rightarrow Zn(\mathbf{pnz})_2(s)$

**CSD CODE**	**Δ*H*° / kJ mol^{-1} per pnz^{-} unit**
WAQQUB	221.607
CAYBAH	221.650
LIHQUP	228.102
WAQRAI	228.875
IMIDZB01	232.051
IMIDZB07	233.130
ONATUT	234.232
BOJXAZ	234.420
AXIVAF	235.917
GUPBOJ	236.843
GITTAF	239.426
CUIMDZ03	240.309
GUPBOJ01	240.472
HIFWAV	241.201
GITTEJ	241.913
VEJYEP	242.444
CUIMDZ02	243.359
IMIDZB11	243.498
OFERUN08	243.873
EQOBUH	243.903
OFERUN01	244.256
SIVGEL	244.377
OFERUN03	244.646
CAYSEB	244.683
PAJRUQ	244.711
VEJYOZ	244.912
HOKMUR	244.975
YOMBOS	245.017
VEJZIU	245.045
SIVGOV	245.138
GUPCAW	245.326
KEYSEO	245.351
EQOCOC01	246.614
VEJYIT	246.861
TOHDEB	246.942
BISPAU	247.032
EHETER02	247.569
CAGLIF	247.596
MECWOH	248.548

Table S11. Calculated enthalpy of formation for Cd(**pnz**)$_2$.

Cd(s) + 5 N_2(g) → Cd(**pnz**)$_2$(s)

**CSD CODE**	**ΔH° / kJ mol^{-1} per pnz unit**
WAQRAI	266.243
WAQQUB	271.196
CAYBAH	271.227
AXIVAF	274.525
LIHQUP	275.135
CUIMDZ02	281.401
ONATUT	281.613
BOJXAZ	281.822
IMIDZB07	283.121
CUIMDZ03	285.842
IMIDZB01	286.203
GUPBOJ	293.155
GUPBOJ01	296.921
GITTAF	298.502
HIFWAV	299.721
GITTEJ	300.125
VEJYEP	301.547
OFERUN01	302.054
EQOBUH	302.296
CAYSEB	302.322
OFERUN08	302.392
IMIDZB11	302.617
SIVGEL	303.087
KEYSEO	303.296
YOMBOS	303.393
SIVGOV	303.425
HOKMUR	303.439
OFERUN03	303.569
PAJRUQ	303.570
VEJZIU	303.668
VEJYOZ	303.712
GUPCAW	303.853
BISPAU	304.525
EQOCOC01	304.693
TOHDEB	304.789
VEJYIT	304.818
EHETER02	305.340
CAGLIF	305.747
MECWOH	307.519

Table S12. Comparison of formation enthalpies of Zn(**pnz)**$_2$ calculated with PBE+D2, PBE+TS, PBE and LDA methods. The data shows that dispersion correction has a relatively small effect on reaction enthalpy, whereas changing a functional from PBE to LDA makes a large difference.

CSD CODE	ΔH° / kJ mol^{-1} per pnz$^-$ unit			
	**PBE+D2**	**PBE+TS**	**PBE**	**LDA**
WAQQUB	221.608	239.640	246.612	23.827
LIHQUP	228.103	237.154	250.914	25.351
IMIDZB01	232.052	242.455	248.436	32.705
IMIDZB07	233.130	242.399	248.461	32.784
ONATUT	234.233	247.555	256.836	34.389
GUPBOJ	236.844	246.879	252.512	36.765
CUIMDZ03	240.310	251.977	253.246	38.379
GUPBOJ01	240.473	250.717	251.262	42.116
HIFWAV	241.202	251.388	250.029	41.500
GITTEJ	241.914	252.226	250.860	42.187

Table S13. Comparison of formation enthalpies of Cd(**pnz)**$_2$ calculated with PBE+D2, PBE+TS, PBE+MBD*, PBE and LDA methods. The data shows that dispersion correction has a relatively small effect on reaction enthalpy, whereas changing a functional from PBE to LDA makes a large difference.

CSD CODE	ΔH° / kJ mol^{-1} per pnz$^-$ unit			
	**PBE+D2**	**PBE+TS**	**PBE**	**LDA**
WAQRAI	263.043	263.586	280.032	54.850
WAQQUB	267.996	268.663	277.538	61.279
AXIVAF	271.325	273.474	285.938	65.639
LIHQUP	271.935	270.121	282.680	66.575
CUIMDZ02	278.202	279.202	296.682	71.945
ONATUT	278.413	278.080	286.782	72.356
BOJXAZ	278.623	278.119	288.532	72.845
IMIDZB07	279.922	278.937	288.185	74.804
CUIMDZ03	282.642	282.870	288.196	77.216
IMIDZB01	283.004	280.862	288.145	77.752

Table S14. Calculated combustion enthalpy and energy density of Zn(**pnz**)$_2$.

Zn(**pnz**)$_2$(s) + 0.5 O_2 (g) → ZnO(s) +N_2(g)

**CSD CODE**	**Δ*H*° / kJ mol^{-1}**	**Energy density / kJ g^{-1}**
WAQQUB	-763.125	3.714
CAYBAH	-763.210	3.715
LIHQUP	-776.114	3.778
WAQRAI	-777.661	3.785
IMIDZB01	-784.012	3.816
IMIDZB07	-786.169	3.827
ONATUT	-788.373	3.837
BOJXAZ	-788.749	3.839
AXIVAF	-791.744	3.854
GUPBOJ	-793.596	3.863
GITTAF	-798.762	3.888
CUIMDZ03	-800.529	3.897
GUPBOJ01	-800.854	3.898
HIFWAV	-802.313	3.905
GITTEJ	-803.737	3.912
VEJYEP	-804.799	3.917
CUIMDZ02	-806.628	3.926
IMIDZB11	-806.907	3.928
OFERUN08	-807.656	3.931
EQOBUH	-807.716	3.932
OFERUN01	-808.423	3.935
SIVGEL	-808.664	3.936
OFERUN03	-809.203	3.939
CAYSEB	-809.277	3.939
PAJRUQ	-809.332	3.939
VEJYOZ	-809.734	3.941
HOKMUR	-809.859	3.942
YOMBOS	-809.944	3.942
VEJZIU	-810.001	3.943
SIVGOV	-810.187	3.944
GUPCAW	-810.562	3.945
KEYSEO	-810.613	3.946
EQOCOC01	-813.138	3.958
VEJYIT	-813.632	3.960
TOHDEB	-813.795	3.961
BISPAU	-813.973	3.962
EHETER02	-815.103	3.967
CAGLIF	-817.005	3.977
MECWOH	-821.466	3.998

Table S15. Calculated combustion enthalpy and energy density of Cd(**pnz**)$_2$.

Cd(**pnz**)$_2$(s) + 0.5 O_2(g) → CdO(s) +N_2(g)

CSD CODE	Δ*H*° / kJ mol^{-1}	Energy density / kJ g^{-1}
WAQRAI	-738.959	2.927
WAQQUB	-748.864	2.966
CAYBAH	-748.927	2.966
AXIVAF	-755.523	2.992
LIHQUP	-756.743	2.997
CUIMDZ02	-769.276	3.047
ONATUT	-769.698	3.049
BOJXAZ	-770.118	3.050
IMIDZB07	-772.716	3.061
CUIMDZ03	-778.157	3.082
IMIDZB01	-778.880	3.085
GUPBOJ	-792.783	3.140
GUPBOJ01	-800.316	3.170
GITTAF	-803.478	3.182
HIFWAV	-805.916	3.192
GITTEJ	-806.724	3.195
VEJYEP	-809.567	3.206
OFERUN01	-810.580	3.210
EQOBUH	-811.065	3.212
CAYSEB	-811.118	3.213
OFERUN08	-811.258	3.213
IMIDZB11	-811.707	3.215
SIVGEL	-812.647	3.219
KEYSEO	-813.065	3.220
YOMBOS	-813.260	3.221
SIVGOV	-813.322	3.221
HOKMUR	-813.352	3.221
OFERUN03	-813.611	3.223
PAJRUQ	-813.614	3.223
VEJZIU	-813.809	3.223
VEJYOZ	-813.898	3.224
GUPCAW	-814.180	3.225
BISPAU	-815.524	3.230
EQOCOC01	-815.859	3.231
TOHDEB	-816.051	3.232
VEJYIT	-816.108	3.232
EHETER02	-817.153	3.237
CAGLIF	-817.967	3.240
MECWOH	-821.510	3.254

Table S16. Comparison of combustion enthalpies of Zn(**pnz)**$_2$ calculated with PBE+D2, PBE+TS, PBE and LDA methods. Data shows that dispersion correction has a relatively small effect on reaction enthalpy, whereas changing the functional from PBE to LDA makes a large difference.

**CSD CODE**	**Δ*H*° / kJ mol^{-1} per pnz$^-$ unit**			
	**PBE+D2**	**PBE+TS**	**PBE**	**LDA**
WAQQUB	-763.126	-772.145	-792.536	-381.249
LIHQUP	-776.115	-767.173	-801.139	-384.299
IMIDZB01	-784.013	-777.775	-796.185	-399.005
IMIDZB07	-786.170	-777.663	-796.234	-399.164
ONATUT	-788.374	-787.975	-812.984	-402.374
GUPBOJ	-793.598	-786.623	-804.335	-407.126
CUIMDZ03	-800.530	-796.820	-805.805	-410.353
GUPBOJ01	-800.856	-794.299	-801.836	-417.827
HIFWAV	-802.314	-795.641	-799.370	-416.595
GITTEJ	-803.738	-797.316	-801.032	-417.969

Table S17. Comparison of combustion enthalpies of Zn(**pnz)**$_2$ calculated with PBE+D2, PBE+TS, PBE and LDA methods. Data shows that dispersion correction has a relatively small effect on reaction enthalpy, whereas changing the functional from PBE to LDA makes a large difference.

**CSD CODE**	**Δ*H*° / kJ mol^{-1} per pnz$^-$ unit**			
	**PBE+D2**	**PBE+TS**	**PBE**	**LDA**
WAQRAI	-738.961	-725.766	-753.948	-348.132
WAQQUB	-748.866	-735.918	-748.960	-360.992
AXIVAF	-755.525	-745.540	-765.762	-369.711
LIHQUP	-756.744	-738.836	-759.244	-371.583
CUIMDZ02	-769.277	-756.996	-787.249	-382.323
ONATUT	-769.700	-754.752	-767.449	-383.145
BOJXAZ	-770.119	-754.830	-770.948	-384.123
IMIDZB07	-772.717	-756.468	-770.254	-388.041
CUIMDZ03	-778.159	-764.333	-770.277	-392.864
IMIDZB01	-778.881	-760.317	-770.174	-393.937

2.3 Calculated atomization enthalpies

Table S18. Comparison of atomization enthalpies of Zn(**pnz**)$_2$ calculated with PBE+D2, PBE+TS, PBE+MBD*, PBE and LDA methods. Dispersion correction has a relatively small effect on reaction enthalpy, whereas LDA gives 20% higher atomization enthalpy.

CSD CODE	$\Delta H°$ / eV per formula unit				
	**PBE+D2**	**PBE+TS**	**PBE+MBD***	**PBE**	**LDA**
WAQQUB	49.390	49.111	49.234	48.533	59.517
LIHQUP	49.255	49.163	49.240	48.444	59.485
IMIDZB01	49.174	49.052	49.167	48.495	59.333
IMIDZB07	49.151	49.054	49.156	48.495	59.331
ONATUT	49.128	48.947	49.083	48.321	59.298
GUPBOJ	49.074	48.961	49.077	48.411	59.249
CUIMDZ03	49.002	48.856	48.993	48.396	59.215
GUPBOJ01	48.999	48.882	49.026	48.437	59.138
HIFWAV	48.984	48.868	49.008	48.462	59.151
GITTEJ	48.969	48.851	48.993	48.445	59.136

Table S19. Comparison of atomization enthalpies of Cd(**pnz**)$_2$ calculated with PBE+D2, PBE+TS, PBE+MBD*, PBE and LDA methods. Dispersion correction has a relatively small effect on reaction enthalpy, whereas LDA gives 15% higher atomization enthalpy.

CSD CODE	$\Delta H°$ / eV per formula unit				
	**PBE+D2**	**PBE+TS**	**PBE+MBD***	**PBE**	**LDA**
WAQRAI	48.337	48.122	48.259	47.365	54.561
WAQQUB	48.234	48.017	48.115	47.416	54.428
AXIVAF	48.165	47.917	48.059	47.242	54.338
LIHQUP	48.153	47.987	48.109	47.310	54.318
CUIMDZ02	48.023	47.798	47.910	47.019	54.207
ONATUT	48.018	47.822	47.966	47.225	54.198
BOJXAZ	48.014	47.821	47.968	47.188	54.188
IMIDZB07	47.987	47.804	47.922	47.196	54.148
CUIMDZ03	47.931	47.722	47.849	47.195	54.098
IMIDZB01	47.923	47.762	47.874	47.196	54.087

Table S20. Comparison of calculated (PBE and LDA) and experimental bond dissociation energies of O_2 and N_2 molecules. While both functionals overestimate dissociation energies, particularly for O_2, the PBE values are in a better agreement with experiment.

	Bond dissociation energy / kJ mol^{-1}		
**Molecule**	**PBE**	**LDA**	**Experimental**
O_2	614.198	722.082	493.59
N_2	1011.715	1122.630	941.49

2.4 Density of States (DOS) analysis

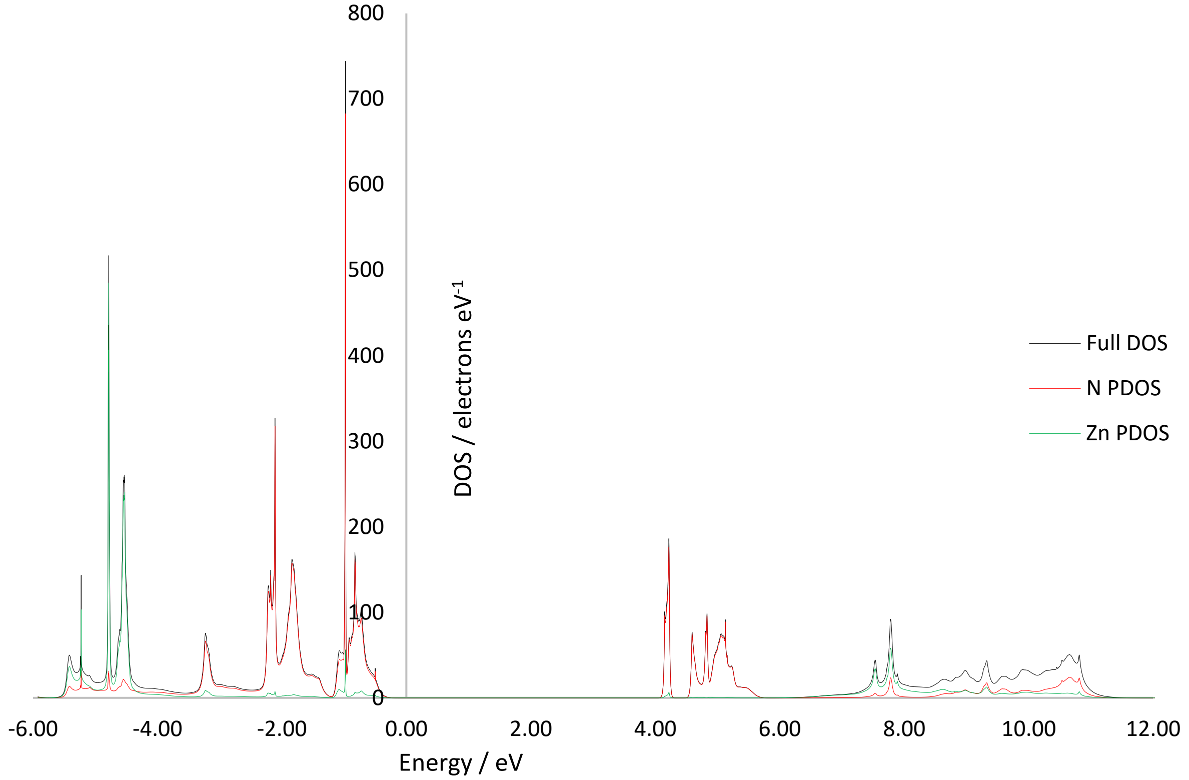

Figure S10. DOS plot for the *crs*-Zn(**pnz**)$_2$ structure. The Projected Density of States (PDOS) corresponding to zinc (green) and nitrogen (red) are also shown, indicating that HOCO and LUCO bands are entirely localized on **pnz**$^-$ ligands.

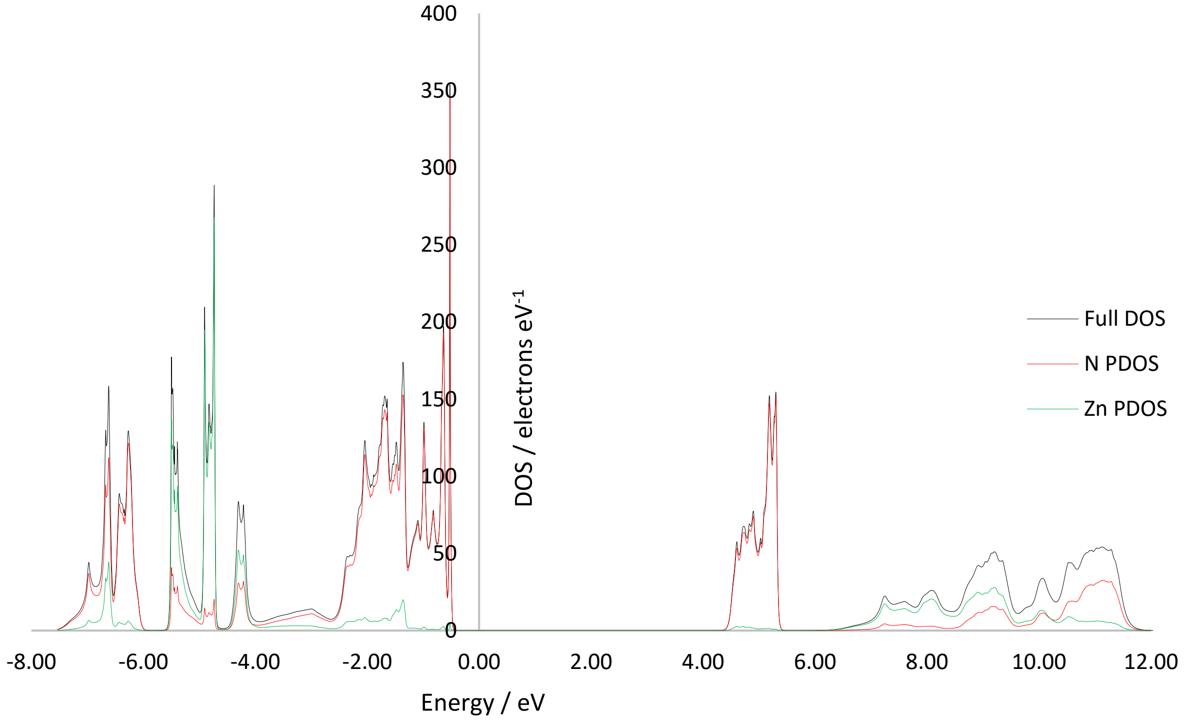

Figure S11. DOS plot for the interpenetrated *dia*-Zn(**pnz**)$_2$ structure. The Projected Density of States (PDOS) corresponding to zinc (green) and nitrogen (red) are also shown, indicating that HOCO and LUCO bands are entirely localized on **pnz**$^-$ ligands.

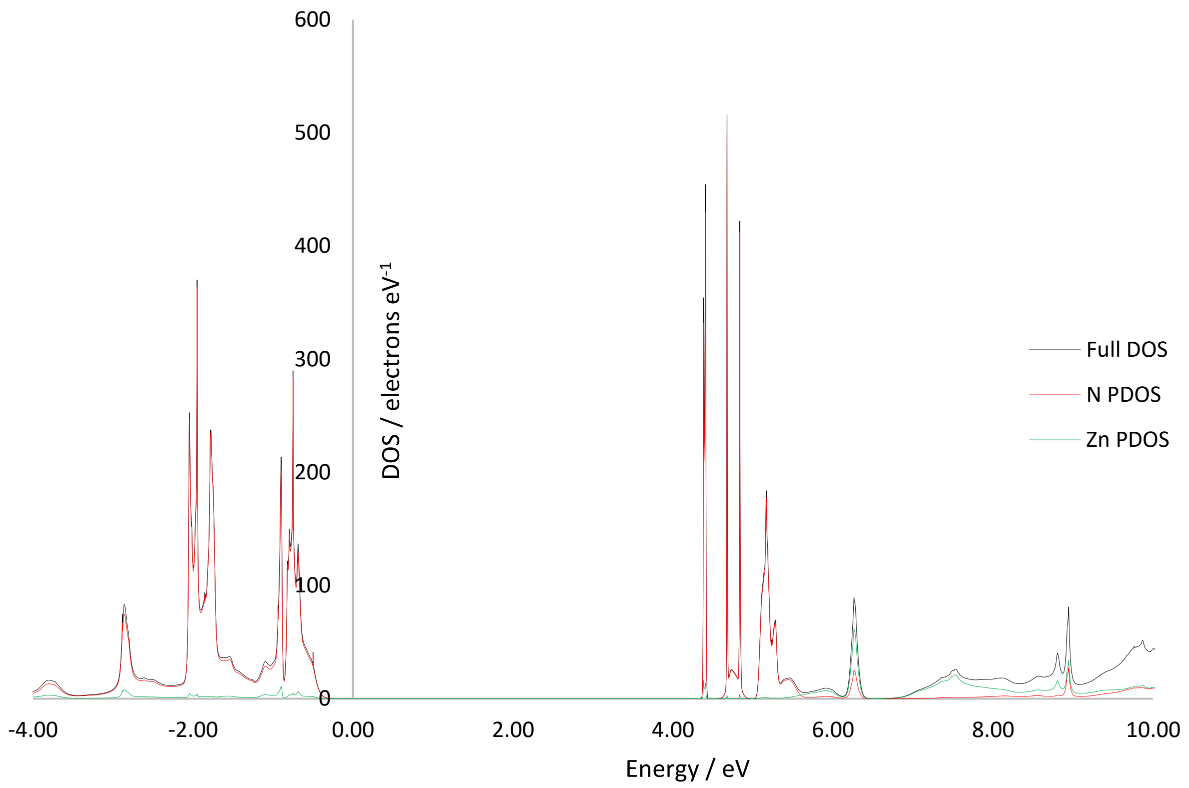

Figure S12. DOS plot for the *zni*-Zn(**pnz**)$_2$ structure. The Projected Density of States (PDOS) corresponding to zinc (green) and nitrogen (red) are also shown, indicating that HOCO and LUCO bands are entirely localized on **pnz**$^-$ ligands.

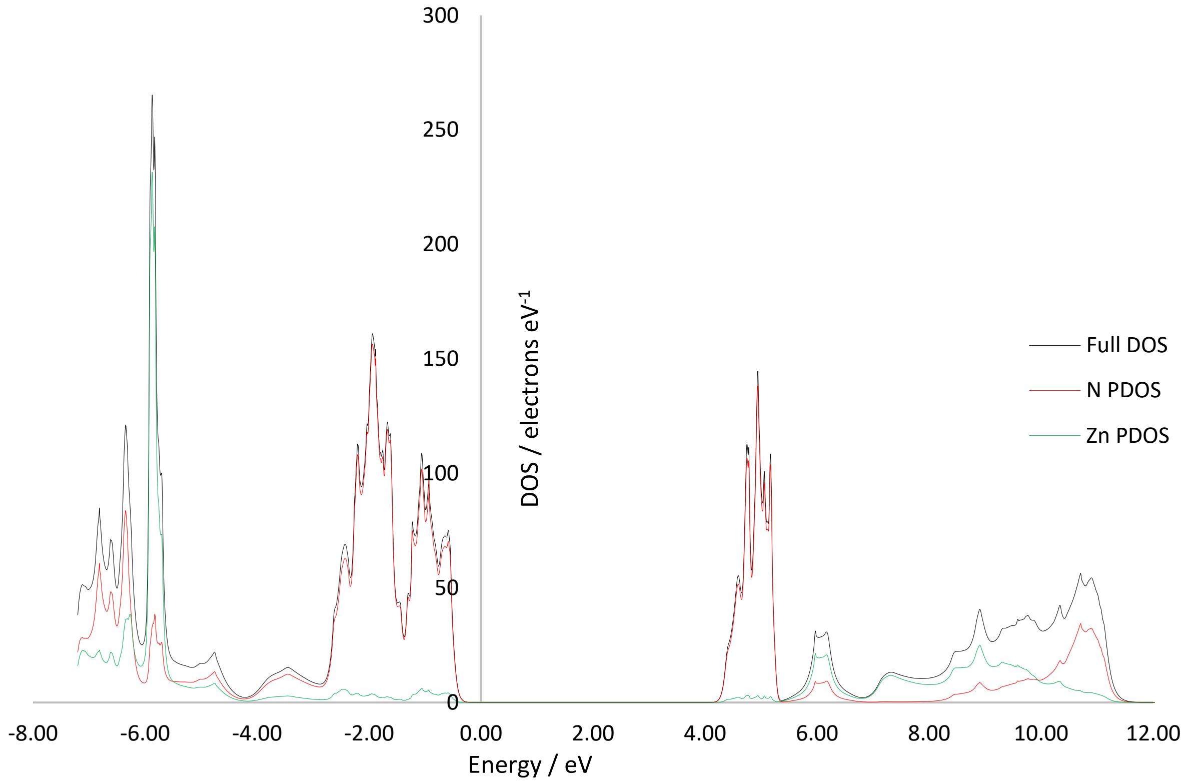

Figure S13. DOS plot for the *arh*-Cd(**pnz**)$_2$ structure. The Projected Density of States (PDOS) corresponding to cadmium (green) and nitrogen (red) are also shown, indicating that HOCO and LUCO bands are entirely localized on **pnz** ligands.

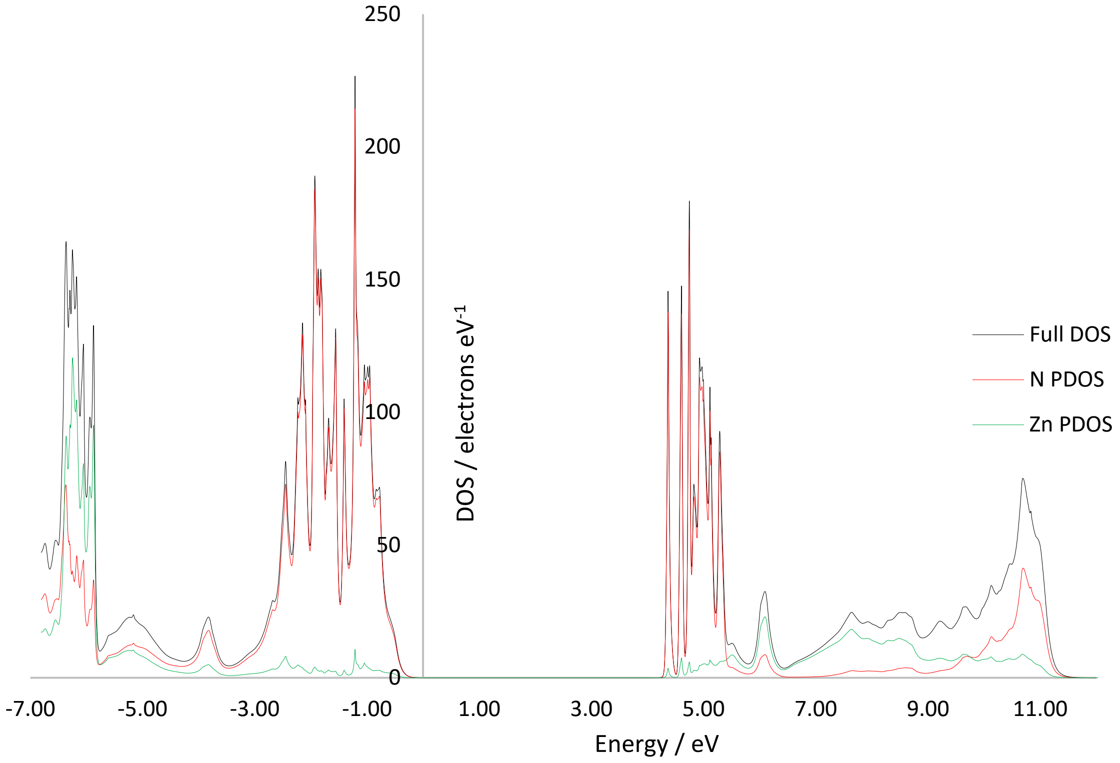

Figure S14. DOS plot for the *crs*-Cd(**pnz**)$_2$ structure. The Projected Density of States (PDOS) corresponding to cadmium (green) and nitrogen (red) are also shown, indicating that HOCO and LUCO bands are entirely localized on **pnz**$^-$ ligands.

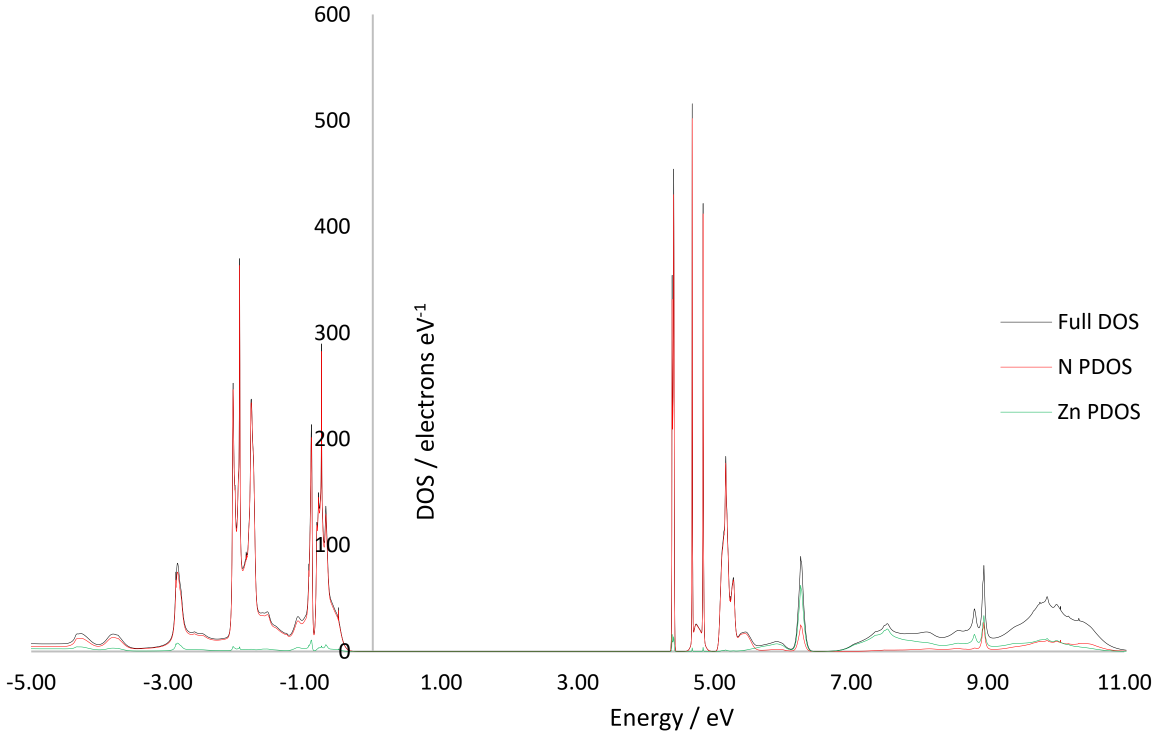

Figure S15. DOS plot for the *bcu*-Cd(**pnz**)$_2$ structure. The Projected Density of States (PDOS) corresponding to cadmium (green) and nitrogen (red) are also shown, indicating that HOCO and LUCO bands are entirely localized on **pnz**$^-$ ligands.

2.5 Phonon calculations

Table S21. Γ-point phonon frequencies for the Zn(**pnz**)$_2$ structure WAQQUB (O_h point group). The first three frequencies correspond to acoustic modes, all vibrational frequencies are real.

Normal mode	Frequency / cm^{-1}	Symmetry
1	-0.032481	Eu
2	-0.032481	Eu
3	0.982512	A2u
4	10.634939	Tu
5	10.634939	Tu
6	10.634939	Tu
7	51.283619	Tg
8	51.283619	Tg
9	51.283619	Tg
10	75.567306	Eu
11	75.567306	Eu
12	76.957512	A2u
13	82.734182	Tu
14	82.734182	Tu
15	82.734182	Tu
16	84.894632	Tg
17	84.894632	Tg
18	84.894632	Tg
19	93.55566	Tg
20	93.55566	Tg
21	93.55566	Tg
22	102.236068	Eu
23	102.236068	Eu
24	126.171805	Tg
25	126.171805	Tg
26	126.171805	Tg
27	135.695856	B1g
28	136.996363	Eu
29	136.996363	Eu
30	137.03198	A2u
31	138.695242	Tg
32	138.695242	Tg
33	138.695242	Tg
34	143.236327	Tu
35	143.236327	Tu
36	143.236327	Tu
37	144.844609	Eg
38	144.844609	Eg
39	154.427257	Eu
40	154.427257	Eu
41	156.829819	A2u
42	171.871708	A1u
43	180.541955	Tg
44	180.541955	Tg

45	180.541955	Tg
46	182.237046	Eu
47	182.237046	Eu
48	190.834906	Tg
49	190.834906	Tg
50	190.834906	Tg
51	192.041776	Tu
52	192.041776	Tu
53	192.041776	Tu
54	193.38257	B1u
55	201.234539	Tu
56	201.234539	Tu
57	201.234539	Tu
58	203.189876	Eu
59	203.189876	Eu
60	204.168995	A2u
61	206.395975	Eu
62	206.395975	Eu
63	210.076214	Tg
64	210.076214	Tg
65	210.076214	Tg
66	214.659882	Eg
67	214.659882	Eg
68	217.645805	A2u
69	224.035839	Eu
70	224.035839	Eu
71	228.686826	B1u
72	230.741649	Tg
73	230.741649	Tg
74	230.741649	Tg
75	231.429715	Tg
76	231.429715	Tg
77	231.429715	Tg
78	231.635857	A1g
79	240.664466	Eu
80	240.664466	Eu
81	252.571054	A2u
82	258.497449	Tu
83	258.497449	Tu
84	258.497449	Tu
85	274.496584	Eu
86	274.496584	Eu
87	278.743477	A2u
88	289.198486	Tg
89	289.198486	Tg
90	289.198486	Tg
91	759.574797	Tg
92	759.574797	Tg

93	759.574797	Tg
94	759.662225	B1g
95	761.345435	Tu
96	761.345435	Tu
97	761.345435	Tu
98	762.336886	A1u
99	762.799427	Eu
100	762.799427	Eu
101	763.05372	Tu
102	763.05372	Tu
103	763.05372	Tu
104	764.000262	Tg
105	764.000262	Tg
106	764.000262	Tg
107	764.347622	Eg
108	764.347622	Eg
109	767.484886	Eu
110	767.484886	Eu
111	767.485449	A2u
112	768.552819	Tg
113	768.552819	Tg
114	768.552819	Tg
115	985.34034	Eg
116	985.34034	Eg
117	985.437356	Eu
118	985.437356	Eu
119	989.640482	Eu
120	989.640482	Eu
121	989.641888	A2u
122	990.262191	Tg
123	990.262191	Tg
124	990.262191	Tg
125	999.591198	B1u
126	1000.896344	A1g
127	1055.740918	Tg
128	1055.740918	Tg
129	1055.740918	Tg
130	1058.433207	Tu
131	1058.433207	Tu
132	1058.433207	Tu
133	1060.162369	Eu
134	1060.162369	Eu
135	1060.165114	A2u
136	1063.131264	Tg
137	1063.131264	Tg
138	1063.131264	Tg
139	1104.370763	Tg
140	1104.370763	Tg

141	1104.370763	Tg
142	1105.12981	Tu
143	1105.12981	Tu
144	1105.12981	Tu
145	1107.783067	Tg
146	1107.783067	Tg
147	1107.783067	Tg
148	1108.274445	Eu
149	1108.274445	Eu
150	1108.317538	A2u
151	1146.862322	Eg
152	1146.862322	Eg
153	1149.002578	Eu
154	1149.002578	Eu
155	1150.986093	Eu
156	1150.986093	Eu
157	1151.00833	A2u
158	1151.254759	Tg
159	1151.254759	Tg
160	1151.254759	Tg
161	1154.388519	B1u
162	1159.968084	A1g
163	1195.770347	Eg
164	1195.770347	Eg
165	1198.880697	Eu
166	1198.880697	Eu
167	1199.477829	Tg
168	1199.477829	Tg
169	1199.477829	Tg
170	1199.88261	Eu
171	1199.88261	Eu
172	1200.106885	A2u
173	1202.752162	B1u
174	1206.853112	A1g
175	1232.689791	Tg
176	1232.689791	Tg
177	1232.689791	Tg
178	1235.874877	Tu
179	1235.874877	Tu
180	1235.874877	Tu
181	1242.364154	Eu
182	1242.364154	Eu
183	1243.226892	A2u
184	1247.984837	Tg
185	1247.984837	Tg
186	1247.984837	Tg
187	1251.871761	Eg
188	1251.871761	Eg

189	1256.626597	Eu
190	1256.626597	Eu
191	1259.617033	Tg
192	1259.617033	Tg
193	1259.617033	Tg
194	1260.479685	Eu
195	1260.479685	Eu
196	1261.818281	A2u
197	1266.384512	B1u
198	1275.40089	A1g

Table S22. Γ-point phonon frequencies for the Zn(**pnz**)$_2$ structure LIHQUP (D_{2h} point group). The first three frequencies correspond to acoustic modes, all vibrational frequencies are real.

Normal mode	Frequency / cm^{-1}	Symmetry
1	-0.03279	B2u
2	-0.023191	B3u
3	0.682197	B1u
4	27.295212	B2g
5	32.041529	Au
6	36.075296	Ag
7	37.040812	B1u
8	39.827106	B1g
9	40.199278	Au
10	42.049159	B3g
11	43.245978	B2g
12	47.223272	Au
13	47.864327	B2u
14	49.511691	Ag
15	53.650195	B3u
16	55.56605	B3g
17	60.881062	B1g
18	63.092262	B2g
19	67.38944	B3g
20	69.013083	B3u
21	70.162308	Ag
22	70.979313	B2u
23	71.083678	Ag
24	76.499813	B1u
25	78.775853	B1g
26	78.856407	B2g
27	82.332808	Au
28	84.431294	B3g
29	84.537329	B1u
30	93.888495	B2u
31	95.198557	B1g
32	95.866258	Au
33	97.405831	B3u
34	104.236415	B3u
35	107.499711	Ag
36	112.147061	B2u
37	113.363321	B2g
38	115.650009	B3g
39	117.11836	Au
40	117.251261	Ag
41	118.152928	B2u
42	120.46266	B1u

43	121.393474	B1g
44	123.082804	B3g
45	124.741578	Au
46	128.213339	B1g
47	128.258411	Ag
48	128.301977	B2g
49	135.761699	B3u
50	137.330889	B1u
51	142.635085	B3g
52	142.930827	B3u
53	143.31394	B2g
54	147.211269	B2u
55	147.676871	Au
56	148.446435	B1u
57	148.85547	Ag
58	153.686538	B1g
59	153.745273	B2u
60	156.327315	B3u
61	158.706541	B3g
62	169.583867	B2g
63	179.752729	B1g
64	181.819036	B1u
65	182.289276	Ag
66	184.815752	B2g
67	187.674244	B3u
68	188.743314	B1g
69	188.807331	Au
70	191.55215	B2u
71	195.191657	B3g
72	196.466372	B2u
73	196.839049	B2g
74	196.911795	B1u
75	197.09686	B1g
76	200.810912	B3u
77	201.544621	B1u
78	206.615184	B2g
79	207.950638	B3g
80	208.087406	B2u
81	211.037737	B1u
82	217.35616	B1g
83	221.170915	Ag
84	222.700581	Ag
85	222.751237	Au
86	225.825326	Au
87	226.935177	B3u
88	229.722914	B2g
89	231.412147	B3g
90	231.810064	B1u
91	233.677691	B3u
92	234.284402	B2u
93	236.986321	B1u
94	237.10514	B1g
95	239.474675	Au
96	242.006065	B3g
97	244.031716	Ag
98	244.789209	B3g
99	245.183927	B3u
100	245.289758	B2u

101	246.08249	B1g
102	247.373256	Au
103	260.861427	Ag
104	261.363113	B2g
105	264.780894	B1u
106	264.845119	B3g
107	266.397383	B3u
108	267.170691	B1g
109	279.60093	B2u
110	281.122644	Ag
111	281.202341	Au
112	288.073906	B2g
113	296.86225	B1g
114	297.973792	Au
115	302.001536	Ag
116	302.956458	B3g
117	304.055483	B2u
118	306.122407	B3u
119	309.652578	B2g
120	313.995446	B1u
121	755.169054	B2g
122	755.173259	Ag
123	756.345599	Au
124	756.390408	B2u
125	757.376879	B1u
126	757.509808	B3u
127	757.796496	B1g
128	757.815874	B3g
129	759.988432	Ag
130	760.079272	B3u
131	760.200546	Au
132	760.22974	B3g
133	761.116111	B1u
134	761.133801	B2u
135	761.406319	B1g
136	761.603387	B3g
137	761.633903	B3u
138	761.866885	Au
139	762.144183	B2g
140	762.199693	B1g
141	762.259787	B2u
142	762.306195	B2g
143	762.351962	B1u
144	762.40622	Ag
145	762.731485	B2g
146	762.882426	B1g
147	762.890127	B1u
148	763.045537	Au
149	763.117449	Ag
150	763.18395	B3g
151	763.212168	B2u
152	763.400493	B3u
153	963.885362	B2g
154	963.955938	B1u
155	964.207935	B1g
156	964.238098	B2u
157	966.646846	Ag
158	966.793107	B3u

159	967.15041	B3g
160	967.804028	Au
161	976.865373	B3g
162	977.217226	B1g
163	979.316786	B3u
164	979.393244	B1u
165	979.533748	B2u
166	980.155232	Au
167	981.789263	B2g
168	981.813832	Ag
169	1003.790276	B1g
170	1003.892505	B1u
171	1004.046026	B2g
172	1004.142007	B2u
173	1005.448417	B3u
174	1005.5371	Au
175	1005.58682	Ag
176	1005.795842	B3g
177	1021.022008	Ag
178	1021.058032	B2g
179	1021.201769	B1u
180	1021.34892	B3u
181	1021.530059	B1g
182	1021.61609	B3g
183	1023.149917	Au
184	1023.178543	B2u
185	1106.778327	B2u
186	1107.450755	Ag
187	1107.453076	Au
188	1107.463703	B1g
189	1107.53512	B3g
190	1107.696975	B2g
191	1107.839754	B1u
192	1108.01866	B3u
193	1112.447041	B2u
194	1112.732005	B1u
195	1112.732724	B2g
196	1112.774233	B3g
197	1112.962933	Au
198	1113.109974	B1g
199	1113.240979	Ag
200	1113.367648	B3u
201	1113.946449	Au
202	1113.981037	B3u
203	1114.129075	B2u
204	1114.207879	B1u
205	1115.21122	Ag
206	1115.309364	B2g
207	1115.746042	B1g
208	1116.500167	B3g
209	1119.871556	Au
210	1119.970301	B3g
211	1120.084109	B1g
212	1120.149303	B2u
213	1121.647145	B3u
214	1121.675502	B2g
215	1121.986362	Ag
216	1122.240795	B1u

217	1221.880529	B3u
218	1221.945377	Ag
219	1222.044422	B3g
220	1222.055012	Au
221	1226.095144	B1g
222	1226.849195	B2u
223	1227.095181	B2g
224	1227.142169	B1u
225	1234.250323	B2u
226	1234.553905	B2g
227	1234.771434	B1g
228	1234.90709	B1u
229	1235.693385	Au
230	1235.876378	Ag
231	1236.176782	B2g
232	1236.230908	B3u
233	1236.427156	B3g
234	1236.540803	B2u
235	1236.690377	Ag
236	1236.826871	Au
237	1239.155585	B1u
238	1239.322188	B3u
239	1240.457472	B3g
240	1240.510346	B1g
241	1242.735	B2u
242	1242.945097	Au
243	1243.059199	B3u
244	1243.475771	B1u
245	1243.558222	B3g
246	1243.907529	B1g
247	1244.22874	B2g
248	1244.662116	Ag
249	1277.54539	Ag
250	1277.550933	B3u
251	1277.924179	B3g
252	1278.092073	Au
253	1280.012117	B2u
254	1280.013429	B1g
255	1280.348279	B2g
256	1280.73588	B1u
257	1291.803604	B3u
258	1291.836028	B1u
259	1291.948513	Au
260	1292.086116	B2u
261	1292.524056	Ag
262	1292.583297	B2g
263	1293.68538	B1g
264	1294.037554	B3g

Table S23. Γ-point phonon frequencies for the Cd(**pnz**)$_2$ structure WAQRAI (D_{2h} point group). The first three frequencies correspond to acoustic modes, all vibrational frequencies are real.

Normal mode	Frequency / cm^{-1}	Symmetry
1	-0.029629	B3u
2	-0.020586	B2u
3	1.365044	B1u
4	3.423977	Au

5	25.440359	B2u
6	35.320304	Au
7	40.583198	B2g
8	43.90114	Ag
9	44.566104	B1u
10	48.776495	B2u
11	48.989982	B2g
12	52.283956	B3g
13	53.771512	Au
14	54.998131	Ag
15	56.644417	B3g
16	56.701999	B1u
17	58.651984	B3u
18	59.924715	B1g
19	59.92939	Ag
20	66.063912	Au
21	67.200036	B1g
22	71.518791	B3u
23	73.262228	Ag
24	76.601974	B2u
25	77.093033	B3g
26	77.243606	B1g
27	78.663836	B3g
28	79.522287	Ag
29	80.818301	B1u
30	81.628157	B2g
31	84.178159	B2u
32	88.496782	B3u
33	89.196222	B2g
34	92.096768	B1g
35	95.833164	Au
36	97.10032	B3g
37	98.930466	B2g
38	102.873679	B3u
39	103.961325	B1g
40	106.091549	Ag
41	106.26433	B3g
42	107.10875	B2u
43	107.111226	B2g
44	108.328875	B1u
45	110.301653	B1g
46	114.110437	B3u
47	114.980501	Au
48	116.825975	B2u
49	117.002907	B3g
50	118.027949	B1u
51	120.581531	Ag
52	125.535706	B3u
53	130.883343	Au
54	132.729697	Ag
55	133.198165	B2g
56	138.878324	B1g
57	138.955614	B2g
58	139.924571	B1u
59	141.454407	B3g
60	144.969	Au
61	145.291383	B1g
62	146.9983	B3g

63	147.077861	B3u
64	148.403824	B2g
65	150.278138	B1u
66	150.648584	B3u
67	151.789167	B1u
68	152.062397	Ag
69	152.569099	B2u
70	155.753478	B1g
71	159.681044	B3g
72	160.071352	B1u
73	160.072746	Au
74	162.120401	B2u
75	163.826545	B1g
76	165.38593	B2g
77	166.907368	Au
78	168.403833	B3u
79	169.902186	Ag
80	169.967367	B1g
81	175.115236	B2g
82	175.608424	Ag
83	179.202131	B2u
84	179.571613	B3u
85	183.511402	B1u
86	187.388549	B3g
87	187.631007	B1g
88	188.92183	Ag
89	190.318431	B3u
90	190.582369	B3g
91	191.464777	B2g
92	192.341937	Au
93	194.832836	Au
94	195.760422	B2u
95	197.357722	B2u
96	199.107356	B2g
97	200.168538	B1u
98	202.195082	Ag
99	202.515209	B3u
100	205.205537	B3u
101	206.191271	Au
102	206.477248	B2u
103	206.515275	B1u
104	207.575746	B1g
105	208.72625	Au
106	209.729091	B3g
107	209.8152	B2u
108	213.199395	B1g
109	216.064355	B3g
110	216.679523	Ag
111	217.886812	B2g
112	218.976058	B2u
113	218.982464	B3u
114	219.916641	Au
115	220.859834	B1u
116	224.185281	B3g
117	226.849124	B2g
118	226.939699	B1u
119	228.274159	Ag
120	229.224726	B1g

121	757.869138	Au
122	757.990164	Ag
123	758.16925	B2u
124	758.261571	B3u
125	758.42094	B2g
126	758.447612	B1u
127	758.488499	B3g
128	758.698872	B1g
129	761.511821	B2u
130	761.574945	B1u
131	761.687827	Au
132	761.740793	B3u
133	762.082719	B2g
134	762.329541	Ag
135	762.433473	B1g
136	762.615205	B3g
137	765.374258	B3u
138	765.510645	B2g
139	765.520172	B1u
140	765.60028	Ag
141	765.743824	Au
142	765.789659	B1g
143	765.905111	B2u
144	765.907216	B3g
145	767.707878	B1u
146	767.830524	B3u
147	768.184491	Ag
148	768.239175	B2g
149	769.301001	B3g
150	769.436901	B1g
151	769.909598	B2u
152	770.096721	Au
153	990.548785	B1u
154	990.64695	B2u
155	993.003884	B1g
156	993.098103	B2g
157	995.427586	Au
158	995.654281	B3u
159	996.994383	Ag
160	997.434381	B3g
161	1003.533852	B2u
162	1004.069585	Au
163	1005.772389	B3u
164	1006.24717	B3g
165	1006.397486	B1u
166	1006.659648	B1g
167	1007.741781	B2g
168	1009.212481	Ag
169	1019.759437	B1g
170	1019.876972	B2g
171	1021.163635	B1u
172	1021.556484	B2u
173	1021.614358	Ag
174	1022.112604	B3g
175	1022.333367	Au
176	1023.529462	B3u
177	1024.203074	B3g
178	1024.210516	B1g

179	1026.837023	Ag
180	1027.254593	B2g
181	1027.696188	B2u
182	1028.289083	Au
183	1029.228612	B3u
184	1029.58284	B1u
185	1107.85781	B2u
186	1108.298618	B1u
187	1109.274823	B2g
188	1109.27982	B1g
189	1110.692072	B3u
190	1110.831229	B3g
191	1110.913146	Au
192	1111.38992	B1u
193	1111.409931	B3u
194	1111.484021	Ag
195	1111.63795	Au
196	1111.940124	B2u
197	1112.13475	B2g
198	1112.481909	B1g
199	1112.48977	Ag
200	1113.722601	B3g
201	1123.404832	Ag
202	1123.418879	B3g
203	1124.39433	B1u
204	1124.420549	Au
205	1124.504203	B1g
206	1124.589909	B2u
207	1124.599438	B2g
208	1124.834062	B3u
209	1125.179834	B3g
210	1125.20582	Ag
211	1125.545474	B2g
212	1125.692842	B1g
213	1126.334389	B3u
214	1127.35421	Au
215	1128.440494	B2u
216	1128.572888	B1u
217	1205.65288	B2u
218	1205.666511	B1u
219	1207.326221	Au
220	1207.78603	B2u
221	1207.891046	B2g
222	1207.954031	B1g
223	1209.841293	Ag
224	1210.048664	B3u
225	1210.632288	Au
226	1211.115143	B3u
227	1211.120875	B3g
228	1211.194268	B1u
229	1211.46174	B1g
230	1211.800712	B3g
231	1213.035904	B2g
232	1215.868407	Ag
233	1223.06054	B3u
234	1223.520908	Au
235	1225.500011	Ag
236	1225.563781	B3g

237	1226.558271	B1g
238	1227.602467	B1u
239	1227.838461	B2u
240	1228.747754	B3u
241	1229.538747	B2g
242	1230.102284	B2u
243	1230.211455	Ag
244	1230.349932	Au
245	1230.744695	B1u
246	1231.987532	B2g
247	1233.339671	B3g
248	1234.947812	B1g
249	1253.327243	Ag
250	1254.193749	B2g
251	1254.551881	B3g
252	1255.34249	B1g
253	1255.345398	Ag
254	1255.662843	B3g
255	1257.770809	B2g
256	1257.866505	B1g
257	1258.081314	Au
258	1258.556492	B3u
259	1259.034817	B1u
260	1259.291123	B3u
261	1260.088914	B2u
262	1261.078213	Au
263	1262.060076	B1u
264	1262.194259	B2u

Table S24. Γ-point phonon frequencies for the Cd(**pnz**)$_2$ structure WAQQUB (O_h point group). The first three frequencies correspond to acoustic modes, all vibrational frequencies are real.

**Normal mode**	**Frequency / cm^{-1}**	**Symmetry**
1	-0.028966	Eu
2	-0.028966	Eu
3	0.817175	A2u
4	21.696259	Tu
5	21.696259	Tu
6	21.696259	Tu
7	46.306477	Tg
8	46.306477	Tg
9	46.306477	Tg
10	58.900002	Eu
11	58.900002	Eu
12	60.141731	Tg
13	60.141731	Tg
14	60.141731	Tg
15	60.547837	A2u
16	61.632156	Tu
17	61.632156	Tu
18	61.632156	Tu
19	68.14558	Tg
20	68.14558	Tg
21	68.14558	Tg
22	85.468658	Eu
23	85.468658	Eu

24	85.910173	Eu
25	85.910173	Eu
26	86.482308	A2u
27	99.254726	Tg
28	99.254726	Tg
29	99.254726	Tg
30	106.03279	Tu
31	106.03279	Tu
32	106.03279	Tu
33	115.614838	Eu
34	115.614838	Eu
35	116.489994	B1g
36	116.986775	A2u
37	126.205772	Tg
38	126.205772	Tg
39	126.205772	Tg
40	130.953446	Eg
41	130.953446	Eg
42	133.559866	A1u
43	139.349241	B1u
44	142.70061	Tg
45	142.70061	Tg
46	142.70061	Tg
47	144.998277	Eu
48	144.998277	Eu
49	145.807496	A2u
50	146.117888	Tu
51	146.117888	Tu
52	146.117888	Tu
53	149.43188	Tg
54	149.43188	Tg
55	149.43188	Tg
56	149.907926	Eu
57	149.907926	Eu
58	161.902899	Tu
59	161.902899	Tu
60	161.902899	Tu
61	166.237878	Eg
62	166.237878	Eg
63	166.843507	Tg
64	166.843507	Tg
65	166.843507	Tg
66	174.846119	Eu
67	174.846119	Eu
68	176.020397	Eu
69	176.020397	Eu
70	179.035124	Tg
71	179.035124	Tg
72	179.035124	Tg
73	182.015209	A2u
74	195.378462	Tg
75	195.378462	Tg
76	195.378462	Tg
77	196.01477	A1g
78	196.043125	B1u
79	199.671974	Eu
80	199.671974	Eu
81	210.212752	A2u

82	212.295764	Tu
83	212.295764	Tu
84	212.295764	Tu
85	215.868156	Eu
86	215.868156	Eu
87	221.216884	A2u
88	227.964196	Tg
89	227.964196	Tg
90	227.964196	Tg
91	755.686012	Tg
92	755.686012	Tg
93	755.686012	Tg
94	755.918184	Tu
95	755.918184	Tu
96	755.918184	Tu
97	758.282955	Eu
98	758.282955	Eu
99	758.283152	A2u
100	758.881839	Tg
101	758.881839	Tg
102	758.881839	Tg
103	760.624339	B1g
104	761.748477	A1u
105	761.850507	Tu
106	761.850507	Tu
107	761.850507	Tu
108	761.941693	Eu
109	761.941693	Eu
110	762.029898	Tg
111	762.029898	Tg
112	762.029898	Tg
113	762.745646	Eg
114	762.745646	Eg
115	969.530197	Eu
116	969.530197	Eu
117	969.658071	Eg
118	969.658071	Eg
119	972.807838	Eu
120	972.807838	Eu
121	972.836098	A2u
122	973.348217	Tg
123	973.348217	Tg
124	973.348217	Tg
125	981.114134	B1u
126	982.154051	A1g
127	1044.468935	Tg
128	1044.468935	Tg
129	1044.468935	Tg
130	1046.071081	Tu
131	1046.071081	Tu
132	1046.071081	Tu
133	1046.42915	Eu
134	1046.42915	Eu
135	1046.441679	A2u
136	1048.640037	Tg
137	1048.640037	Tg
138	1048.640037	Tg
139	1100.410726	Tg

140	1100.410726	Tg
141	1100.410726	Tg
142	1100.513881	Tu
143	1100.513881	Tu
144	1100.513881	Tu
145	1102.643801	Tg
146	1102.643801	Tg
147	1102.643801	Tg
148	1103.038986	Eu
149	1103.038986	Eu
150	1103.043325	A2u
151	1140.548016	Eg
152	1140.548016	Eg
153	1141.639608	Eu
154	1141.639608	Eu
155	1142.216656	Eu
156	1142.216656	Eu
157	1142.232248	A2u
158	1142.624574	Tg
159	1142.624574	Tg
160	1142.624574	Tg
161	1143.563012	B1u
162	1147.131733	A1g
163	1190.611608	Eg
164	1190.611608	Eg
165	1192.292043	Eu
166	1192.292043	Eu
167	1192.680639	Tg
168	1192.680639	Tg
169	1192.680639	Tg
170	1192.890729	Eu
171	1192.890729	Eu
172	1193.086994	A2u
173	1194.591076	B1u
174	1196.741752	A1g
175	1224.486865	Tg
176	1224.486865	Tg
177	1224.486865	Tg
178	1226.897159	Tu
179	1226.897159	Tu
180	1226.897159	Tu
181	1231.198101	Eu
182	1231.198101	Eu
183	1232.286747	A2u
184	1234.614194	Tg
185	1234.614194	Tg
186	1234.614194	Tg
187	1241.659247	Eg
188	1241.659247	Eg
189	1244.454766	Eu
190	1244.454766	Eu
191	1247.112624	Tg
192	1247.112624	Tg
193	1247.112624	Tg
194	1247.222787	Eu
195	1247.222787	Eu
196	1248.346051	A2u
197	1252.977732	B1u

198	1258.093114	A1g

Table S25. Γ-point phonon frequencies for the Cd(**pnz**)$_2$ structure AXIVAF (D_{2h} point group). The first three frequencies correspond to acoustic modes, all vibrational frequencies are real.

Normal mode	Frequency / cm^{-1}	Symmetry
1	-0.036284	B2u
2	-0.028961	B3u
3	0.989211	B1u
4	17.606269	Au
5	28.267596	B1u
6	35.802513	B1g
7	36.152312	Ag
8	40.285741	B2g
9	45.529953	B2u
10	48.376193	B1g
11	49.65454	Ag
12	49.896803	Au
13	55.915359	B3u
14	56.596954	Ag
15	58.219752	B3g
16	59.341107	B2g
17	70.942802	B1u
18	70.975183	B3g
19	71.740286	Au
20	77.510073	B3u
21	78.695816	B3g
22	80.405189	Au
23	81.555346	B1g
24	81.952448	Ag
25	83.471403	B2u
26	86.053438	B3u
27	87.135681	B2g
28	87.258357	B3g
29	89.987495	B2u
30	90.01668	B1u
31	92.341433	B2g
32	94.820141	Au
33	95.803636	B3u
34	96.868387	Ag
35	100.654275	B2u
36	102.123291	B1g
37	105.533351	Ag
38	107.023157	B3g
39	107.825953	B1g
40	110.021714	B2g
41	114.123236	B2u
42	114.933391	Ag
43	115.421602	B1u
44	116.963148	B1u
45	117.42201	B2g
46	117.577677	B3g
47	117.620389	Au
48	119.912887	B1g
49	120.809884	B1g
50	122.854394	B2g
51	123.980773	B3u
52	126.653722	B3g
53	126.693722	Ag

54	129.572674	B2u
55	130.172073	B2g
56	131.287476	B1u
57	132.020363	Au
58	133.628339	B1g
59	134.059834	B3u
60	136.407262	B3g
61	136.607326	B2g
62	143.806844	B3g
63	144.08407	B3u
64	145.126298	B2g
65	146.889602	Ag
66	147.523987	B1g
67	148.08521	B1u
68	149.01659	Au
69	150.634634	B1u
70	152.237739	B1g
71	152.790141	Au
72	153.895302	B2u
73	155.201479	B1u
74	155.295762	B3u
75	155.856601	B2u
76	158.019076	B3g
77	161.383392	B3u
78	162.597063	Ag
79	165.042568	B2u
80	165.501906	Au
81	173.024825	Ag
82	174.217994	B3u
83	174.219088	B2u
84	174.594858	B3g
85	175.855524	B1g
86	180.229417	B2g
87	181.561659	Au
88	184.610681	B3u
89	189.00388	B3g
90	189.989162	B2g
91	191.036858	B1u
92	191.192553	B2u
93	191.388234	B3g
94	192.197332	Au
95	192.937392	Ag
96	198.905338	B1g
97	199.687342	B1u
98	200.551818	B2g
99	202.097481	Ag
100	203.302103	B1g
101	205.57052	B2u
102	208.886509	Au
103	212.216795	B1u
104	217.432265	B3u
105	219.226101	B1g
106	220.71993	B2u
107	227.066342	Au
108	230.947119	B3u
109	234.792931	B2g
110	235.533033	Ag
111	236.733382	B3g

112	245.739385	B1u
113	249.412506	B3u
114	251.75849	Au
115	253.014664	B2u
116	256.799033	B1u
117	258.349261	B1g
118	260.919639	B2g
119	262.870276	Ag
120	263.297055	B3g
121	759.696678	B1g
122	759.826063	B2g
123	759.92627	Au
124	759.960249	Ag
125	760.046507	B2u
126	760.312008	B3u
127	760.39256	B1u
128	760.607481	Au
129	760.643621	B3g
130	760.802057	B3u
131	761.040683	B1u
132	761.051063	B2u
133	761.621	Ag
134	762.024062	B3g
135	762.080065	B2u
136	762.201217	B2g
137	762.403002	B1g
138	762.421339	B1u
139	762.49325	B3u
140	763.01551	Au
141	765.369715	B3g
142	765.560809	Ag
143	765.562598	B1g
144	765.836241	B2g
145	772.025687	B3g
146	772.050339	B2g
147	772.083356	B1g
148	772.343087	Ag
149	775.971449	B3u
150	776.228317	B1u
151	776.287125	Au
152	776.295574	B2u
153	992.319751	B3u
154	992.531793	B1u
155	993.126609	B1g
156	993.255512	B3g
157	993.860342	Au
158	994.440004	Ag
159	994.59019	B2u
160	995.054444	B2g
161	999.784173	Ag
162	1000.065373	B1u
163	1000.209047	B3g
164	1000.24198	B2u
165	1000.63471	B2g
166	1000.664268	B1g
167	1001.650241	B3u
168	1001.774362	Au
169	1007.986175	B3g

170	1008.141405	Au
171	1008.569817	B2g
172	1008.637459	B2u
173	1009.002477	Ag
174	1009.605057	B1u
175	1009.820716	B1g
176	1009.862563	B3u
177	1011.365566	B2g
178	1011.693418	B3g
179	1011.809687	B1g
180	1012.945244	B3u
181	1012.985026	B1u
182	1013.438222	Au
183	1014.660964	Ag
184	1015.32415	B2u
185	1102.958268	B3g
186	1103.199503	B1u
187	1103.212535	B3u
188	1103.561123	B1g
189	1104.071174	Ag
190	1104.109227	B2g
191	1104.316695	Au
192	1104.752843	B2u
193	1105.779045	B1u
194	1105.94758	B2u
195	1106.040842	B3u
196	1106.145836	B1g
197	1106.205085	Au
198	1106.717551	B2g
199	1106.740645	Ag
200	1106.882937	B3g
201	1120.374037	B1u
202	1120.774377	B3u
203	1121.281712	B2u
204	1121.494938	B3g
205	1121.703037	B1g
206	1121.736017	Au
207	1122.604826	B2g
208	1122.687805	Ag
209	1124.390037	B2g
210	1124.684747	B3g
211	1125.704608	Ag
212	1125.804429	B1g
213	1126.580249	Au
214	1126.718854	B1u
215	1126.772252	B3u
216	1127.617381	B2u
217	1208.641601	B2u
218	1209.574965	B3g
219	1209.840034	Ag
220	1210.696397	Au
221	1211.313418	B3u
222	1211.534641	B1u
223	1212.080217	B2g
224	1212.660018	B1g
225	1212.820583	Ag
226	1213.013357	B2g
227	1213.351221	B3g

228	1214.331817	B1g
229	1215.14003	Au
230	1216.475211	B2u
231	1216.999835	B1g
232	1217.165499	B3u
233	1217.656781	B1u
234	1217.789937	B2g
235	1218.160916	Ag
236	1218.809821	B1u
237	1218.901581	B3g
238	1219.297248	B3u
239	1220.433024	B2u
240	1220.758974	Au
241	1223.176348	B3u
242	1223.231366	Au
243	1223.804102	B2u
244	1224.793985	B1u
245	1227.300298	Ag
246	1227.395201	B1g
247	1227.903776	B2g
248	1229.261666	B3g
249	1263.486557	B2u
250	1263.980415	B1u
251	1264.019132	Au
252	1264.514518	B3u
253	1268.223228	Ag
254	1268.523688	B2g
255	1269.013004	B3g
256	1269.291426	B1g
257	1272.094419	B1g
258	1272.109927	Ag
259	1272.65632	B2g
260	1272.990419	B3g
261	1276.85298	B3u
262	1277.345611	B2u
263	1277.668118	Au
264	1278.195796	B1u

2.6 Analysis of π···π interactions in pentazolate framework structures

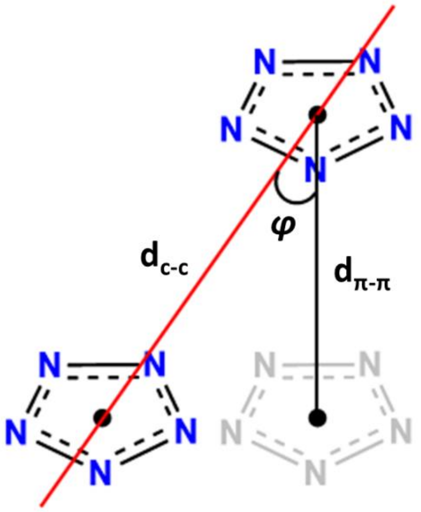

Table S26. Distances between centroids of **pnz**⁻ rings in selected Zn(**pnz**)₂ structures.

CSD Code	Topology	Symmetry op.	$d_{c\text{-}c}$ / Å	α / °	$d_{\pi\text{-}\pi}$ / Å	φ / °
WAQQUB	*crs*	x, y, z	4.6027	60	—	—
LIHQUP	*dia (interp.)*	-0.5+x, y, 1.5-z	4.2773	73.95	—	—
IMIDZB01	*zni*	x, y, z	4.3015	63.76	—	—
IMIDZB07	*coi*	x, y, z	4.3327	61.05	—	—
ONATUT	*sql*	x, 1.5-y, -0.5+z	3.9869	86.81	—	—
GUPBOJ	*yqt1*	x, -1+y, z	3.895	55.53	—	—
CUIMDZ03	*mog*	x, y, z	4.1756	79.95	—	—
GUPBOJ01	*ict*	x, 0.5-y, -0.5+z	4.241	89.64	—	—
HIFWAV	*nog*	1-x, 2-y, 1-z	4.4537	89.51	—	—
GITTEJ	*crb*	1.5-x, y, 0.5-z	4.3894	89.83	—	—

Table 27. Distances between centroids of **pnz**⁻ rings in selected Cd(**pnz**)₂ structures. The shortest contacts in CUIMDZ02 and CUIMDZ03 correspond to mutually parallel arrangement of **pnz**⁻ rings and may be regarded as π-π stacking interactions.

CSD Code	Topology	Symmetry op.	$d_{c\text{-}c}$ / Å	α / °	$d_{\pi\text{-}\pi}$ / Å	φ / °
WAQRAI	*arh*	x, 1.5-y, 0.5+z	4.0291	52.21	—	—
WAQQUB	*crs*	x, y, z	4.8935	60	—	—
AXIVAF	*bcu*	1.5-x, -0.5+y, z	3.8763	67.88	—	—
LIHQUP	*dia (interp.)*	x, y, z	4.2967	66.26	—	—
CUIMDZ02	*4,4L37*	1.5-x, 1-y, z	3.0811	15.37	3.03	10.2
ONATUT	*seh-3,5-Pbca*	0.5+x, y, 1.5-z	4.1507	89.43	—	—
BOJXAZ	*sql*	1-x, 1-y, 2-z	3.9523	86.01	—	—
CUIMDZ03	*$\{4.6^2\}_2\{4.6^9\}_2\{6^6\}$*	-1+x, -1+y, z	3.628	0	2.89	37.25
IMIDZB01	*zni*	x, 1+y, z	4.2247	53.75	—	—
IMIDZB07	*coi*	x, y, z	3.9604	42.4	—	—

3. Crystal structures of pentazolate frameworks in CIF format

Table S28. Crystallographic (CIF) data for structures of Zn(**pnz**)$_2$ (left) and Cd(**pnz**)$_2$ (right). Structures that were converted to non-standard space groups during CASTEP optimization (see section 1.2) are printed in *P1* space group.

```
data_1-crs_Zn
_audit_creation_method ToposPro
_Chemical_Name_Systematic WAQQUB
_cell_length_a 12.34618
_cell_length_b 12.34618
_cell_length_c 12.34618
_cell_angle_alpha 60
_cell_angle_beta 60
_cell_angle_gamma 60
_cell_volume 1330.708
_cell_formula_units_Z 6
_symmetry_space_group_name_H-M 'P 1'
_symmetry_Int_Tables_number 1
loop_
_symmetry_equiv_pos_site_id
_symmetry_equiv_pos_as_xyz
1 x,y,z
loop_
_atom_site_label
_atom_site_type_symbol
_atom_site_symmetry_multiplicity
_atom_site_fract_x
_atom_site_fract_y
_atom_site_fract_z
_atom_site_occupancy
N1 N 1 0.50270 0.50270 0.74730 1.0000
N2 N 1 0.50270 0.74730 0.50270 1.0000
N3 N 1 0.74730 0.50270 0.50270 1.0000
N4 N 1 0.25270 0.25270 0.49730 1.0000
N5 N 1 0.50270 0.74730 0.74730 1.0000
N6 N 1 0.74730 0.50270 0.74730 1.0000
N7 N 1 0.49730 0.49730 0.25270 1.0000
N8 N 1 0.74730 0.74730 0.50270 1.0000
N9 N 1 0.25270 0.49730 0.49730 1.0000
N10 N 1 0.49730 0.25270 0.25270 1.0000
N11 N 1 0.49730 0.25270 0.49730 1.0000
N12 N 1 0.25270 0.49730 0.25270 1.0000
N13 N 1 0.45843 0.45843 0.87683 1.0000
N14 N 1 0.45843 0.70631 0.45843 1.0000
N15 N 1 0.70631 0.45843 0.45843 1.0000
N16 N 1 0.29369 0.12317 0.54157 1.0000
N17 N 1 0.45843 0.70631 0.87683 1.0000
N18 N 1 0.87683 0.45843 0.70631 1.0000
N19 N 1 0.54157 0.54157 0.12317 1.0000
N20 N 1 0.12317 0.29369 0.54157 1.0000
N21 N 1 0.87683 0.70631 0.45843 1.0000
N22 N 1 0.29369 0.54157 0.54157 1.0000
N23 N 1 0.54157 0.29369 0.12317 1.0000
N24 N 1 0.54157 0.12317 0.54157 1.0000
N25 N 1 0.29369 0.54157 0.12317 1.0000
N26 N 1 0.45843 0.87683 0.45843 1.0000
N27 N 1 0.70631 0.87683 0.45843 1.0000
N28 N 1 0.45843 0.87683 0.70631 1.0000
N29 N 1 0.87683 0.45843 0.45843 1.0000
N30 N 1 0.45843 0.45843 0.70631 1.0000
N31 N 1 0.54157 0.54157 0.29369 1.0000
N32 N 1 0.70631 0.45843 0.87683 1.0000
N33 N 1 0.12317 0.54157 0.29369 1.0000
N34 N 1 0.12317 0.54157 0.54157 1.0000
N35 N 1 0.54157 0.29369 0.54157 1.0000
N36 N 1 0.54157 0.12317 0.29369 1.0000
N37 N 1 0.38672 0.38672 0.91620 1.0000
N38 N 1 0.38672 0.81036 0.38672 1.0000
N39 N 1 0.81036 0.38672 0.38672 1.0000
N40 N 1 0.18964 0.08380 0.61328 1.0000
N41 N 1 0.38672 0.81036 0.91620 1.0000
N42 N 1 0.91620 0.38672 0.81036 1.0000
N43 N 1 0.61328 0.61328 0.08380 1.0000
N44 N 1 0.08380 0.18964 0.61328 1.0000
N45 N 1 0.91620 0.81036 0.38672 1.0000
N46 N 1 0.18964 0.61328 0.61328 1.0000
N47 N 1 0.61328 0.18964 0.08380 1.0000
N48 N 1 0.61328 0.08380 0.61328 1.0000
N49 N 1 0.18964 0.61328 0.08380 1.0000
N50 N 1 0.38672 0.91620 0.38672 1.0000
N51 N 1 0.81036 0.91620 0.38672 1.0000
N52 N 1 0.38672 0.91620 0.81036 1.0000
N53 N 1 0.91620 0.38672 0.38672 1.0000
N54 N 1 0.38672 0.38672 0.81036 1.0000
N55 N 1 0.61328 0.61328 0.18964 1.0000
N56 N 1 0.81036 0.38672 0.91620 1.0000
N57 N 1 0.08380 0.61328 0.18964 1.0000
N58 N 1 0.08380 0.61328 0.61328 1.0000
N59 N 1 0.61328 0.18964 0.61328 1.0000
N60 N 1 0.61328 0.08380 0.18964 1.0000
Zn1 Zn 1 0.62500 0.62500 0.62500 1.0000
Zn2 Zn 1 0.37500 0.37500 0.37500 1.0000
Zn3 Zn 1 0.50000 0.50000 0.00000 1.0000
Zn4 Zn 1 0.50000 0.50000 0.50000 1.0000
Zn5 Zn 1 0.50000 0.00000 0.50000 1.0000
Zn6 Zn 1 0.00000 0.50000 0.50000 1.0000
#End
```

```
data_1-arh_Cd
_audit_creation_method ToposPro
_Chemical_Name_Systematic WAQRAI
_cell_length_a 12.28534
_cell_length_b 8.289567
_cell_length_c 12.99
_cell_angle_alpha 90
_cell_angle_beta 90
_cell_angle_gamma 90
_cell_volume 1322.904
_cell_formula_units_Z 8
_symmetry_space_group_name_H-M 'P b c a'
_symmetry_Int_Tables_number 61
loop_
_symmetry_equiv_pos_site_id
_symmetry_equiv_pos_as_xyz
1 x,y,z
2 1/2-x,-y,1/2+z
3 1/2+x,1/2-y,-z
4 -x,1/2+y,1/2-z
5 -x,-y,-z
6 1/2+x,y,1/2-z
7 1/2-x,1/2+y,z
8 x,1/2-y,1/2+z
loop_
_atom_site_label
_atom_site_type_symbol
_atom_site_symmetry_multiplicity
_atom_site_fract_x
_atom_site_fract_y
_atom_site_fract_z
_atom_site_occupancy
N1 N 8 0.94078 0.34277 0.25484 1.0000
N2 N 8 0.76034 0.18130 0.44347 1.0000
N3 N 8 0.69094 0.24672 0.50801 1.0000
N4 N 8 0.98262 0.48542 0.23713 1.0000
N5 N 8 0.91765 0.27197 0.16696 1.0000
N6 N 8 0.94622 0.37136 0.09456 1.0000
N7 N 8 0.98649 0.50350 0.13791 1.0000
N8 N 8 0.60917 0.14752 0.51478 1.0000
N9 N 8 0.62842 0.02165 0.45476 1.0000
N10 N 8 0.72221 0.04309 0.41056 1.0000
Cd1 Cd 8 0.94258 0.25109 0.42464 1.0000
#End

data_2-crs_Cd
_audit_creation_method ToposPro
_Chemical_Name_Systematic WAQQUB
_cell_length_a 13.07531
_cell_length_b 13.07531
_cell_length_c 13.07531
_cell_angle_alpha 60
_cell_angle_beta 60
_cell_angle_gamma 60
_cell_volume 1580.668
_cell_formula_units_Z 6
_symmetry_space_group_name_H-M 'P 1'
_symmetry_Int_Tables_number 1
loop_
_symmetry_equiv_pos_site_id
_symmetry_equiv_pos_as_xyz
1 x,y,z
loop_
_atom_site_label
_atom_site_type_symbol
_atom_site_symmetry_multiplicity
_atom_site_fract_x
_atom_site_fract_y
_atom_site_fract_z
_atom_site_occupancy
N1 N 1 0.49846 0.49846 0.75154 1.0000
N2 N 1 0.49846 0.75154 0.49846 1.0000
N3 N 1 0.75154 0.49846 0.49846 1.0000
N4 N 1 0.24846 0.24846 0.50154 1.0000
N5 N 1 0.49846 0.75154 0.75154 1.0000
N6 N 1 0.75154 0.49846 0.75154 1.0000
N7 N 1 0.50154 0.50154 0.24846 1.0000
N8 N 1 0.75154 0.75154 0.49846 1.0000
N9 N 1 0.24846 0.50154 0.50154 1.0000
N10 N 1 0.50154 0.24846 0.24846 1.0000
N11 N 1 0.50154 0.24846 0.50154 1.0000
N12 N 1 0.24846 0.50154 0.24846 1.0000
N13 N 1 0.45658 0.45658 0.87397 1.0000
N14 N 1 0.45658 0.71286 0.45658 1.0000
N15 N 1 0.71286 0.45658 0.45658 1.0000
N16 N 1 0.28714 0.12603 0.54342 1.0000
N17 N 1 0.45658 0.71286 0.87397 1.0000
N18 N 1 0.87397 0.45658 0.71286 1.0000
N19 N 1 0.54342 0.54342 0.12603 1.0000
N20 N 1 0.12603 0.28714 0.54342 1.0000
N21 N 1 0.87397 0.71286 0.45658 1.0000
N22 N 1 0.28714 0.54342 0.54342 1.0000
N23 N 1 0.54342 0.28714 0.12603 1.0000
```

N24 N 1 0.54342 0.12603 0.54342 1.0000
N25 N 1 0.28714 0.54342 0.12603 1.0000
N26 N 1 0.45658 0.87397 0.45658 1.0000
N27 N 1 0.71286 0.87397 0.45658 1.0000
N28 N 1 0.45658 0.87397 0.71286 1.0000
N29 N 1 0.87397 0.45658 0.45658 1.0000
N30 N 1 0.45658 0.45658 0.71286 1.0000
N31 N 1 0.54342 0.54342 0.28714 1.0000
N32 N 1 0.71286 0.45658 0.87397 1.0000
N33 N 1 0.12603 0.54342 0.28714 1.0000
N34 N 1 0.12603 0.54342 0.54342 1.0000
N35 N 1 0.54342 0.28714 0.54342 1.0000
N36 N 1 0.54342 0.12603 0.28714 1.0000
N37 N 1 0.38887 0.38887 0.91118 1.0000
N38 N 1 0.38887 0.81108 0.38887 1.0000
N39 N 1 0.81108 0.38887 0.38887 1.0000
N40 N 1 0.18892 0.08882 0.61113 1.0000
N41 N 1 0.38887 0.81108 0.91118 1.0000
N42 N 1 0.91118 0.38887 0.81108 1.0000
N43 N 1 0.61113 0.61113 0.08882 1.0000
N44 N 1 0.08882 0.18892 0.61113 1.0000
N45 N 1 0.91118 0.81108 0.38887 1.0000
N46 N 1 0.18892 0.61113 0.61113 1.0000
N47 N 1 0.61113 0.18892 0.08882 1.0000
N48 N 1 0.61113 0.08882 0.61113 1.0000
N49 N 1 0.18892 0.61113 0.08882 1.0000
N50 N 1 0.38887 0.91118 0.38887 1.0000
N51 N 1 0.81108 0.91118 0.38887 1.0000
N52 N 1 0.38887 0.91118 0.81108 1.0000
N53 N 1 0.91118 0.38887 0.38887 1.0000
N54 N 1 0.38887 0.38887 0.81108 1.0000
N55 N 1 0.61113 0.61113 0.18892 1.0000
N56 N 1 0.81108 0.38887 0.91118 1.0000
N57 N 1 0.08882 0.61113 0.18892 1.0000
N58 N 1 0.08882 0.61113 0.61113 1.0000
N59 N 1 0.61113 0.18892 0.61113 1.0000
N60 N 1 0.61113 0.08882 0.18892 1.0000
Cd1 Cd 1 0.62500 0.62500 0.62500 1.0000
Cd2 Cd 1 0.37500 0.37500 0.37500 1.0000
Cd3 Cd 1 0.50000 0.50000 0.00000 1.0000
Cd4 Cd 1 0.50000 0.50000 0.50000 1.0000
Cd5 Cd 1 0.50000 0.00000 0.50000 1.0000
Cd6 Cd 1 0.00000 0.50000 0.50000 1.0000
#End

data_2-crs2_Zn
_audit_creation_method TopospPro
_Chemical_Name_Systematic CAYBAH
_cell_length_a 12.31533
_cell_length_b 12.31533
_cell_length_c 12.36988
_cell_angle_alpha 60.14577
_cell_angle_beta 60.14577
_cell_angle_gamma 90
_cell_volume 1332.432
_cell_formula_units_Z 6
_symmetry_space_group_name_H-M 'P 1'
_symmetry_Int_Tables_number 1
loop_
_symmetry_equiv_pos_site_id
_symmetry_equiv_pos_as_xyz
1 x,y,z
loop_
_atom_site_label
_atom_site_type_symbol
_atom_site_symmetry_multiplicity
_atom_site_fract_x
_atom_site_fract_y
_atom_site_fract_z
_atom_site_occupancy
N1 N 1 0.75362 0.50362 0.49276 1.0000
N2 N 1 0.99638 0.74638 0.00724 1.0000
N3 N 1 0.00362 0.25362 0.99276 1.0000
N4 N 1 0.24638 0.49638 0.50724 1.0000
N5 N 1 0.75220 0.74780 0.25000 1.0000
N6 N 1 0.75220 0.50220 0.25000 1.0000
N7 N 1 0.00220 0.49780 0.75000 1.0000
N8 N 1 0.00220 0.25220 0.75000 1.0000
N9 N 1 0.24780 0.49780 0.75000 1.0000
N10 N 1 0.99780 0.74780 0.25000 1.0000
N11 N 1 0.99780 0.50220 0.25000 1.0000
N12 N 1 0.24780 0.25220 0.75000 1.0000
N13 N 1 0.62423 0.45963 0.58074 1.0000
N14 N 1 0.04037 0.70497 0.91926 1.0000
N15 N 1 0.95963 0.29503 0.08074 1.0000
N16 N 1 0.20497 0.54037 0.41926 1.0000
N17 N 1 0.37577 0.54037 0.41926 1.0000
N18 N 1 0.95963 0.12423 0.08074 1.0000
N19 N 1 0.04037 0.87577 0.91926 1.0000
N20 N 1 0.79503 0.45963 0.58074 1.0000
N21 N 1 0.62272 0.70703 0.33533 1.0000
N22 N 1 0.62272 0.45764 0.33533 1.0000
N23 N 1 0.79297 0.45805 0.16467 1.0000
N24 N 1 0.95764 0.54195 0.83533 1.0000
N25 N 1 0.95805 0.29297 0.66467 1.0000
N26 N 1 0.37728 0.54236 0.66467 1.0000
N27 N 1 0.95764 0.12272 0.83533 1.0000
N28 N 1 0.79297 0.87728 0.16467 1.0000
N29 N 1 0.04236 0.87728 0.16467 1.0000
N30 N 1 0.95805 0.54236 0.66467 1.0000
N31 N 1 0.04195 0.45764 0.33533 1.0000
N32 N 1 0.37728 0.29297 0.66467 1.0000
N33 N 1 0.20703 0.54195 0.83533 1.0000
N34 N 1 0.04195 0.70703 0.33533 1.0000
N35 N 1 0.04236 0.45805 0.16467 1.0000
N36 N 1 0.20703 0.12272 0.83533 1.0000
N37 N 1 0.58535 0.38838 0.72323 1.0000
N38 N 1 0.11162 0.80858 0.77677 1.0000
N39 N 1 0.88838 0.19142 0.22323 1.0000
N40 N 1 0.30858 0.61162 0.27677 1.0000
N41 N 1 0.41465 0.61162 0.27677 1.0000
N42 N 1 0.88838 0.08535 0.22323 1.0000
N43 N 1 0.11162 0.91465 0.77677 1.0000
N44 N 1 0.69142 0.38838 0.72323 1.0000
N45 N 1 0.58314 0.81123 0.30292 1.0000
N46 N 1 0.58314 0.38585 0.30292 1.0000
N47 N 1 0.68877 0.38606 0.19708 1.0000
N48 N 1 0.88585 0.61394 0.80292 1.0000
N49 N 1 0.88606 0.18877 0.69708 1.0000
N50 N 1 0.41686 0.61415 0.69708 1.0000
N51 N 1 0.88585 0.08314 0.80292 1.0000
N52 N 1 0.68877 0.91686 0.19708 1.0000
N53 N 1 0.11415 0.91686 0.19708 1.0000
N54 N 1 0.88606 0.61415 0.69708 1.0000
N55 N 1 0.11394 0.38585 0.30292 1.0000
N56 N 1 0.41686 0.18877 0.69708 1.0000
N57 N 1 0.31123 0.61394 0.80292 1.0000
N58 N 1 0.11394 0.81123 0.30292 1.0000
N59 N 1 0.11415 0.38606 0.19708 1.0000
N60 N 1 0.31123 0.08314 0.80292 1.0000
Zn1 Zn 1 0.87500 0.62500 0.25000 1.0000
Zn2 Zn 1 0.12500 0.37500 0.75000 1.0000
Zn3 Zn 1 0.50000 0.50000 0.50000 1.0000
Zn4 Zn 1 0.00000 0.50000 0.00000 1.0000
Zn5 Zn 1 0.00000 0.50000 0.50000 1.0000
Zn6 Zn 1 0.00000 0.00000 0.00000 1.0000
#End

data_3-crs2_Cd
_audit_creation_method TopospPro
_Chemical_Name_Systematic CAYBAH
_cell_length_a 13.04304
_cell_length_b 13.04304
_cell_length_c 13.0961
_cell_angle_alpha 60.13392
_cell_angle_beta 60.13392
_cell_angle_gamma 90
_cell_volume 1581.733
_cell_formula_units_Z 6
_symmetry_space_group_name_H-M 'P 1'
_symmetry_Int_Tables_number 1
loop_
_symmetry_equiv_pos_site_id
_symmetry_equiv_pos_as_xyz
1 x,y,z
loop_
_atom_site_label
_atom_site_type_symbol
_atom_site_symmetry_multiplicity
_atom_site_fract_x
_atom_site_fract_y
_atom_site_fract_z
_atom_site_occupancy
N1 N 1 0.74941 0.49941 0.50118 1.0000
N2 N 1 0.00059 0.75059 0.99882 1.0000
N3 N 1 0.99941 0.24941 0.00118 1.0000
N4 N 1 0.25059 0.50059 0.49882 1.0000
N5 N 1 0.74800 0.75200 0.25000 1.0000
N6 N 1 0.74800 0.49800 0.25000 1.0000
N7 N 1 0.99800 0.50200 0.75000 1.0000
N8 N 1 0.99800 0.24800 0.75000 1.0000
N9 N 1 0.25200 0.50200 0.75000 1.0000
N10 N 1 0.00200 0.75200 0.25000 1.0000
N11 N 1 0.00200 0.49800 0.25000 1.0000
N12 N 1 0.25200 0.24800 0.75000 1.0000
N13 N 1 0.62705 0.45775 0.58450 1.0000
N14 N 1 0.04225 0.71155 0.91550 1.0000
N15 N 1 0.95775 0.28845 0.08450 1.0000
N16 N 1 0.21155 0.54225 0.41550 1.0000
N17 N 1 0.37295 0.54225 0.41550 1.0000
N18 N 1 0.95775 0.12705 0.08450 1.0000
N19 N 1 0.04225 0.87295 0.91550 1.0000
N20 N 1 0.78845 0.45775 0.58450 1.0000
N21 N 1 0.62556 0.71352 0.33064 1.0000
N22 N 1 0.62556 0.45584 0.33064 1.0000
N23 N 1 0.78648 0.45620 0.16936 1.0000
N24 N 1 0.95584 0.54380 0.83064 1.0000
N25 N 1 0.95620 0.28648 0.66936 1.0000
N26 N 1 0.37444 0.54416 0.66936 1.0000
N27 N 1 0.95584 0.12556 0.83064 1.0000
N28 N 1 0.78648 0.87444 0.16936 1.0000
N29 N 1 0.04416 0.87444 0.16936 1.0000
N30 N 1 0.95620 0.54416 0.66936 1.0000
N31 N 1 0.04380 0.45584 0.33064 1.0000

data_3-dia_Inter1_Zn
_audit_creation_method TopospPro
_Chemical_Name_Systematic LIHQUP
_cell_length_a 9.571742
_cell_length_b 9.067689
_cell_length_c 14.23642
_cell_angle_alpha 90
_cell_angle_beta 90

_cell_angle_gamma 90
_cell_volume 1235.63
_cell_formula_units_Z 8
_symmetry_space_group_name_H-M 'P b c a'
_symmetry_Int_Tables_number 61
loop_
_symmetry_equiv_pos_site_id
_symmetry_equiv_pos_as_xyz
1 x,y,z
2 1/2-x,-y,1/2+z
3 1/2+x,1/2-y,-z
4 -x,1/2+y,1/2-z
5 -x,-y,-z
6 1/2+x,y,1/2-z
7 1/2-x,1/2+y,z
8 x,1/2-y,1/2+z
loop_
_atom_site_label
_atom_site_type_symbol
_atom_site_symmetry_multiplicity
_atom_site_fract_x
_atom_site_fract_y
_atom_site_fract_z
_atom_site_occupancy
N1 N 8 0.04711 0.05083 0.67765 1.0000
N2 N 8 0.15065 0.85291 0.71182 1.0000
N3 N 8 0.04669 0.87729 0.77221 1.0000
N4 N 8 0.12502 0.29704 0.51831 1.0000
N5 N 8 0.10728 0.39997 0.45270 1.0000
N6 N 8 0.30321 0.29700 0.43562 1.0000
N7 N 8 0.98250 0.99928 0.75131 1.0000
N8 N 8 0.15049 0.96070 0.65292 1.0000
N9 N 8 0.24607 0.23275 0.50755 1.0000
N10 N 8 0.21788 0.40005 0.40143 1.0000
Zn1 Zn 8 0.99236 0.24413 0.61991 1.0000
#End

data_4-dia_Inter2_Zn
_audit_creation_method TopsPro
_Chemical_Name_Systematic WAQRAI
_cell_length_a 9.783242
_cell_length_b 9.058628
_cell_length_c 13.72628
_cell_angle_alpha 90
_cell_angle_beta 90
_cell_angle_gamma 90
_cell_volume 1216.461
_cell_formula_units_Z 8
_symmetry_space_group_name_H-M 'P b c a'
_symmetry_Int_Tables_number 61
loop_
_symmetry_equiv_pos_site_id
_symmetry_equiv_pos_as_xyz
1 x,y,z
2 1/2-x,-y,1/2+z
3 1/2+x,1/2-y,-z
4 -x,1/2+y,1/2-z
5 -x,-y,-z
6 1/2+x,y,1/2-z
7 1/2-x,1/2+y,z
8 x,1/2-y,1/2+z
loop_
_atom_site_label
_atom_site_type_symbol
_atom_site_symmetry_multiplicity
_atom_site_fract_x
_atom_site_fract_y
_atom_site_fract_z
_atom_site_occupancy
N1 N 8 0.95157 0.35132 0.26969 1.0000
N2 N 8 0.76613 0.20124 0.43094 1.0000
N3 N 8 0.71478 0.27245 0.50457 1.0000
N4 N 8 0.03195 0.46150 0.24912 1.0000
N5 N 8 0.85176 0.34140 0.20407 1.0000
N6 N 8 0.87012 0.44624 0.14220 1.0000
N7 N 8 0.98113 0.52028 0.17027 1.0000
N8 N 8 0.58844 0.22716 0.51123 1.0000
N9 N 8 0.56187 0.12887 0.44159 1.0000
N10 N 8 0.67223 0.11265 0.39169 1.0000
Zn1 Zn 8 0.95819 0.21515 0.38403 1.0000
#End

data_5-zni_Zn
_audit_creation_method TopsPro
_Chemical_Name_Systematic IMIDZB01
_cell_length_a 21.53028
_cell_length_b 21.53028
_cell_length_c 16.48898
_cell_angle_alpha 49.24176
_cell_angle_beta 49.24176
_cell_angle_gamma 90
_cell_volume 2935.795
_cell_formula_units_Z 16
_symmetry_space_group_name_H-M 'P 1'
_symmetry_Int_Tables_number 1
loop_
_symmetry_equiv_pos_site_id
_symmetry_equiv_pos_as_xyz
1 x,y,z
loop_
_atom_site_label

N32 N 1 0.37444 0.28648 0.66936 1.0000
N33 N 1 0.21352 0.54380 0.83064 1.0000
N34 N 1 0.04380 0.71352 0.33064 1.0000
N35 N 1 0.04416 0.45620 0.16936 1.0000
N36 N 1 0.21352 0.12556 0.83064 1.0000
N37 N 1 0.59027 0.39042 0.71916 1.0000
N38 N 1 0.10958 0.80943 0.78084 1.0000
N39 N 1 0.89042 0.19057 0.21916 1.0000
N40 N 1 0.30943 0.60958 0.28084 1.0000
N41 N 1 0.40973 0.60958 0.28084 1.0000
N42 N 1 0.89042 0.09027 0.21916 1.0000
N43 N 1 0.10958 0.90973 0.78084 1.0000
N44 N 1 0.69057 0.39042 0.71916 1.0000
N45 N 1 0.58816 0.81188 0.30009 1.0000
N46 N 1 0.58816 0.38802 0.30009 1.0000
N47 N 1 0.68812 0.38825 0.19991 1.0000
N48 N 1 0.88802 0.61175 0.80009 1.0000
N49 N 1 0.88825 0.18812 0.69991 1.0000
N50 N 1 0.41184 0.61198 0.69991 1.0000
N51 N 1 0.88802 0.08816 0.80009 1.0000
N52 N 1 0.68812 0.91184 0.19991 1.0000
N53 N 1 0.11198 0.91184 0.19991 1.0000
N54 N 1 0.88825 0.61198 0.69991 1.0000
N55 N 1 0.11175 0.38802 0.30009 1.0000
N56 N 1 0.41184 0.18812 0.69991 1.0000
N57 N 1 0.31188 0.61175 0.80009 1.0000
N58 N 1 0.11175 0.81188 0.30009 1.0000
N59 N 1 0.11198 0.38825 0.19991 1.0000
N60 N 1 0.31188 0.08816 0.80009 1.0000
Cd1 Cd 1 0.87500 0.62500 0.25000 1.0000
Cd2 Cd 1 0.12500 0.37500 0.75000 1.0000
Cd3 Cd 1 0.50000 0.50000 0.50000 1.0000
Cd4 Cd 1 0.00000 0.50000 0.00000 1.0000
Cd5 Cd 1 0.00000 0.50000 0.50000 1.0000
Cd6 Cd 1 0.00000 0.00000 0.00000 1.0000
#End

data_4-bcu_Cd
_audit_creation_method TopsPro
_Chemical_Name_Systematic AXIVAF
_cell_length_a 9.479085
_cell_length_b 12.44622
_cell_length_c 12.33336
_cell_angle_alpha 90
_cell_angle_beta 90
_cell_angle_gamma 90
_cell_volume 1455.074
_cell_formula_units_Z 8
_symmetry_space_group_name_H-M 'P b c a'
_symmetry_Int_Tables_number 61
loop_
_symmetry_equiv_pos_site_id
_symmetry_equiv_pos_as_xyz
1 x,y,z
2 1/2-x,-y,1/2+z
3 1/2+x,1/2-y,-z
4 -x,1/2+y,1/2-z
5 -x,-y,-z
6 1/2+x,y,1/2-z
7 1/2-x,1/2+y,z
8 x,1/2-y,1/2+z
loop_
_atom_site_label
_atom_site_type_symbol
_atom_site_symmetry_multiplicity
_atom_site_fract_x
_atom_site_fract_y
_atom_site_fract_z
_atom_site_occupancy
N1 N 8 0.04304 0.68032 0.00953 1.0000
N2 N 8 0.08097 0.78029 0.00366 1.0000
N3 N 8 0.20809 0.78560 0.04640 1.0000
N4 N 8 0.14657 0.62488 0.05546 1.0000
N5 N 8 0.24906 0.68983 0.07865 1.0000
N6 N 8 0.83918 0.52518 0.12553 1.0000
N7 N 8 0.94536 0.46903 0.16682 1.0000
N8 N 8 0.73598 0.52459 0.19471 1.0000
N9 N 8 0.90850 0.43305 0.26121 1.0000
N10 N 8 0.77931 0.46754 0.27805 1.0000
Cd1 Cd 8 0.15000 0.43238 0.06458 1.0000
#End

data_5-dia_Inter_Cd
_audit_creation_method TopsPro
_Chemical_Name_Systematic LIHQUP
_cell_length_a 9.699145
_cell_length_b 8.864894
_cell_length_c 15.7991
_cell_angle_alpha 90
_cell_angle_beta 90
_cell_angle_gamma 90
_cell_volume 1358.437
_cell_formula_units_Z 8
_symmetry_space_group_name_H-M 'P b c a'
_symmetry_Int_Tables_number 61
loop_
_symmetry_equiv_pos_site_id
_symmetry_equiv_pos_as_xyz
1 x,y,z
2 1/2-x,-y,1/2+z
3 1/2+x,1/2-y,-z

_atom_site_type_symbol
_atom_site_symmetry_multiplicity
_atom_site_fract_x
_atom_site_fract_y
_atom_site_fract_z
_atom_site_occupancy
N1 N 1 0.32654 0.01575 0.04943 1.0000
N2 N 1 0.76575 0.87403 0.54943 1.0000
N3 N 1 0.18482 0.37403 0.54943 1.0000
N4 N 1 0.82654 0.43482 0.04943 1.0000
N5 N 1 0.12403 0.51575 0.04943 1.0000
N6 N 1 0.62403 0.93482 0.04943 1.0000
N7 N 1 0.68482 0.57654 0.54943 1.0000
N8 N 1 0.26575 0.07654 0.54943 1.0000
N9 N 1 0.36910 0.94072 0.01454 1.0000
N10 N 1 0.69072 0.86635 0.51454 1.0000
N11 N 1 0.29473 0.36635 0.51454 1.0000
N12 N 1 0.86910 0.54473 0.01454 1.0000
N13 N 1 0.11635 0.44072 0.01454 1.0000
N14 N 1 0.61635 0.04473 0.01454 1.0000
N15 N 1 0.79473 0.61910 0.51454 1.0000
N16 N 1 0.19072 0.11910 0.51454 1.0000
N17 N 1 0.41161 0.04478 0.90835 1.0000
N18 N 1 0.79478 0.93003 0.40835 1.0000
N19 N 1 0.29687 0.43003 0.40835 1.0000
N20 N 1 0.91161 0.54687 0.90835 1.0000
N21 N 1 0.18003 0.54478 0.90835 1.0000
N22 N 1 0.68003 0.04687 0.90835 1.0000
N23 N 1 0.79687 0.66161 0.40835 1.0000
N24 N 1 0.29478 0.16161 0.40835 1.0000
N25 N 1 0.25828 0.84785 0.22047 1.0000
N26 N 1 0.59785 0.77125 0.72047 1.0000
N27 N 1 0.18169 0.27125 0.72047 1.0000
N28 N 1 0.75828 0.43169 0.22047 1.0000
N29 N 1 0.02125 0.34785 0.22047 1.0000
N30 N 1 0.52125 0.93169 0.22047 1.0000
N31 N 1 0.68169 0.50828 0.72047 1.0000
N32 N 1 0.09785 0.00828 0.72047 1.0000
N33 N 1 0.23191 0.89445 0.24201 1.0000
N34 N 1 0.64445 0.77608 0.74201 1.0000
N35 N 1 0.11354 0.27608 0.74201 1.0000
N36 N 1 0.73191 0.36354 0.24201 1.0000
N37 N 1 0.02608 0.39445 0.24201 1.0000
N38 N 1 0.52608 0.86354 0.24201 1.0000
N39 N 1 0.61354 0.48191 0.74201 1.0000
N40 N 1 0.14445 0.98191 0.74201 1.0000
N41 N 1 0.17237 0.99957 0.30998 1.0000
N42 N 1 0.74957 0.76765 0.80998 1.0000
N43 N 1 0.94045 0.26765 0.80998 1.0000
N44 N 1 0.67237 0.19045 0.30998 1.0000
N45 N 1 0.01765 0.49957 0.30998 1.0000
N46 N 1 0.51765 0.69045 0.30998 1.0000
N47 N 1 0.44045 0.42237 0.80998 1.0000
N48 N 1 0.24957 0.92237 0.80998 1.0000
N49 N 1 0.03540 0.83561 0.62803 1.0000
N50 N 1 0.58561 0.58657 0.12803 1.0000
N51 N 1 0.78636 0.08657 0.12803 1.0000
N52 N 1 0.53540 0.03636 0.62803 1.0000
N53 N 1 0.83657 0.33561 0.62803 1.0000
N54 N 1 0.33657 0.53636 0.62803 1.0000
N55 N 1 0.28636 0.28540 0.12803 1.0000
N56 N 1 0.08561 0.78540 0.12803 1.0000
N57 N 1 0.15386 0.92936 0.44004 1.0000
N58 N 1 0.67936 0.65610 0.94004 1.0000
N59 N 1 0.88060 0.15610 0.94004 1.0000
N60 N 1 0.65386 0.13060 0.44004 1.0000
N61 N 1 0.90610 0.42936 0.44004 1.0000
N62 N 1 0.40610 0.63060 0.44004 1.0000
N63 N 1 0.38060 0.40386 0.94004 1.0000
N64 N 1 0.17936 0.90386 0.94004 1.0000
N65 N 1 0.98064 0.84741 0.61488 1.0000
N66 N 1 0.59741 0.65448 0.11488 1.0000
N67 N 1 0.78771 0.15448 0.11488 1.0000
N68 N 1 0.48064 0.03771 0.61488 1.0000
N69 N 1 0.90448 0.34741 0.61488 1.0000
N70 N 1 0.40448 0.53771 0.61488 1.0000
N71 N 1 0.28771 0.23064 0.11488 1.0000
N72 N 1 0.09741 0.73064 0.11488 1.0000
N73 N 1 0.34313 0.23164 0.83844 1.0000
N74 N 1 0.98164 0.06843 0.33844 1.0000
N75 N 1 0.17992 0.56843 0.33844 1.0000
N76 N 1 0.84313 0.42992 0.83844 1.0000
N77 N 1 0.31843 0.73164 0.83844 1.0000
N78 N 1 0.81843 0.92992 0.83844 1.0000
N79 N 1 0.67992 0.59313 0.33844 1.0000
N80 N 1 0.48164 0.09313 0.33844 1.0000
N81 N 1 0.38065 0.32973 0.62135 1.0000
N82 N 1 0.07973 0.24800 0.12135 1.0000
N83 N 1 0.29892 0.74800 0.12135 1.0000
N84 N 1 0.88065 0.54892 0.62135 1.0000
N85 N 1 0.49800 0.82973 0.62135 1.0000
N86 N 1 0.99800 0.04892 0.62135 1.0000
N87 N 1 0.79892 0.63065 0.12135 1.0000
N88 N 1 0.57973 0.13065 0.12135 1.0000
N89 N 1 0.06571 0.94919 0.41732 1.0000
N90 N 1 0.69919 0.76697 0.91732 1.0000
N91 N 1 0.88348 0.26697 0.91732 1.0000
N92 N 1 0.56571 0.13348 0.41732 1.0000
N93 N 1 0.01697 0.44919 0.41732 1.0000
N94 N 1 0.51697 0.63348 0.41732 1.0000
N95 N 1 0.38348 0.31571 0.91732 1.0000

4 -x,1/2+y,1/2-z
5 -x,-y,-z
6 1/2+x,y,1/2-z
7 1/2-x,1/2+y,z
8 x,1/2-y,1/2+z
loop_
_atom_site_label
_atom_site_type_symbol
_atom_site_symmetry_multiplicity
_atom_site_fract_x
_atom_site_fract_y
_atom_site_fract_z
_atom_site_occupancy
N1 N 8 0.07551 0.09624 0.67138 1.0000
N2 N 8 0.16741 0.88963 0.70758 1.0000
N3 N 8 0.06753 0.92502 0.76156 1.0000
N4 N 8 0.13023 0.35018 0.50582 1.0000
N5 N 8 0.13514 0.45264 0.44493 1.0000
N6 N 8 0.30293 0.30492 0.43178 1.0000
N7 N 8 0.01088 0.05239 0.73947 1.0000
N8 N 8 0.17191 0.99603 0.65131 1.0000
N9 N 8 0.23380 0.25830 0.49764 1.0000
N10 N 8 0.24295 0.42469 0.39901 1.0000
Cd1 Cd 8 0.99212 0.31869 0.61691 1.0000
#End

data_6-4,4L37_Cd
_audit_creation_method ToposPro
_Chemical_Name_Systematic CUIMDZ02
_cell_length_a 15.16488
_cell_length_b 20.13414
_cell_length_c 10.1453
_cell_angle_alpha 90
_cell_angle_beta 90
_cell_angle_gamma 90
_cell_volume 3097.683
_cell_formula_units_Z 20
_symmetry_space_group_name_H-M 'C c c a'
_symmetry_Int_Tables_number 68
loop_
_symmetry_equiv_pos_site_id
_symmetry_equiv_pos_as_xyz
1 x,y,z
2 1/2-x,-y,z
3 1/2+x,-y,1/2-z
4 -x,y,1/2-z
5 -x,-y,-z
6 1/2+x,y,-z
7 1/2-x,y,1/2+z
8 x,-y,1/2+z
9 1/2+x,1/2+y,z
10 -x,1/2-y,z
11 x,1/2-y,1/2-z
12 1/2-x,1/2+y,1/2-z
13 1/2-x,1/2-y,-z
14 x,1/2+y,-z
15 -x,1/2+y,1/2+z
16 1/2+x,1/2-y,1/2+z
loop_
_atom_site_label
_atom_site_type_symbol
_atom_site_symmetry_multiplicity
_atom_site_fract_x
_atom_site_fract_y
_atom_site_fract_z
_atom_site_occupancy
N1 N 16 0.90801 0.34306 0.98418 1.0000
N2 N 16 0.97806 0.32147 0.92179 1.0000
N3 N 16 0.93636 0.36699 0.09538 1.0000
N4 N 16 0.04889 0.33149 0.99412 1.0000
N5 N 16 0.02287 0.35987 0.10187 1.0000
N6 N 16 0.88152 0.50731 0.02874 1.0000
N7 N 16 0.86219 0.51032 0.15408 1.0000
N8 N 16 0.83447 0.55323 0.97046 1.0000
N9 N 16 0.80418 0.55903 0.17223 1.0000
N10 N 16 0.78736 0.58556 0.05850 1.0000
N11 N 16 0.78344 0.30176 0.25842 1.0000
N12 N 8 0.83493 0.25000 0.25000 1.0000
N13 N 16 0.70014 0.28212 0.25495 1.0000
Cd1 Cd 4 0.00000 0.25000 0.75000 1.0000
Cd2 Cd 16 0.84919 0.40419 0.26637 1.0000
#End

data_7-seh_Cd
_audit_creation_method ToposPro
_Chemical_Name_Systematic ONATUT
_cell_length_a 8.695808
_cell_length_b 11.37483
_cell_length_c 15.81304
_cell_angle_alpha 90
_cell_angle_beta 90
_cell_angle_gamma 90
_cell_volume 1564.12
_cell_formula_units_Z 8
_symmetry_space_group_name_H-M 'P b c a'
_symmetry_Int_Tables_number 61
loop_
_symmetry_equiv_pos_site_id
_symmetry_equiv_pos_as_xyz
1 x,y,z
2 1/2-x,-y,1/2+z

N96 N 1 0.19919 0.81571 0.91732 1.0000
N97 N 1 0.39870 0.27106 0.68766 1.0000
N98 N 1 0.02106 0.16364 0.18766 1.0000
N99 N 1 0.29128 0.66364 0.18766 1.0000
N100 N 1 0.89870 0.54128 0.68766 1.0000
N101 N 1 0.41364 0.77106 0.68766 1.0000
N102 N 1 0.91364 0.04128 0.68766 1.0000
N103 N 1 0.79128 0.64870 0.18766 1.0000
N104 N 1 0.52106 0.14870 0.18766 1.0000
N105 N 1 0.29051 0.26527 0.86606 1.0000
N106 N 1 0.01527 0.09343 0.36606 1.0000
N107 N 1 0.11867 0.59343 0.36606 1.0000
N108 N 1 0.79051 0.36867 0.86606 1.0000
N109 N 1 0.34343 0.76527 0.86606 1.0000
N110 N 1 0.84343 0.86867 0.86606 1.0000
N111 N 1 0.61867 0.54051 0.36606 1.0000
N112 N 1 0.51527 0.04051 0.36606 1.0000
N113 N 1 0.59521 0.23183 0.84252 1.0000
N114 N 1 0.98183 0.81227 0.34252 1.0000
N115 N 1 0.17564 0.31227 0.34252 1.0000
N116 N 1 0.09521 0.42564 0.84252 1.0000
N117 N 1 0.06227 0.73183 0.84252 1.0000
N118 N 1 0.56227 0.92564 0.84252 1.0000
N119 N 1 0.67564 0.84521 0.34252 1.0000
N120 N 1 0.48183 0.34521 0.34252 1.0000
N121 N 1 0.48251 0.21572 0.87613 1.0000
N122 N 1 0.96572 0.89137 0.37613 1.0000
N123 N 1 0.15815 0.39137 0.37613 1.0000
N124 N 1 0.98251 0.40815 0.87613 1.0000
N125 N 1 0.14137 0.71572 0.87613 1.0000
N126 N 1 0.64137 0.90815 0.87613 1.0000
N127 N 1 0.65815 0.73251 0.37613 1.0000
N128 N 1 0.46572 0.23251 0.37613 1.0000
N129 N 1 0.31409 0.32651 0.73074 1.0000
N130 N 1 0.07651 0.20517 0.23074 1.0000
N131 N 1 0.19275 0.70517 0.23074 1.0000
N132 N 1 0.81409 0.44275 0.73074 1.0000
N133 N 1 0.45517 0.82651 0.73074 1.0000
N134 N 1 0.95517 0.94275 0.73074 1.0000
N135 N 1 0.69275 0.56409 0.23074 1.0000
N136 N 1 0.57651 0.06409 0.23074 1.0000
N137 N 1 0.49228 0.15226 0.95811 1.0000
N138 N 1 0.90226 0.79962 0.45811 1.0000
N139 N 1 0.13963 0.29962 0.45811 1.0000
N140 N 1 0.99228 0.38963 0.95811 1.0000
N141 N 1 0.04962 0.65226 0.95811 1.0000
N142 N 1 0.54962 0.88963 0.95811 1.0000
N143 N 1 0.63963 0.74228 0.45811 1.0000
N144 N 1 0.40226 0.24228 0.45811 1.0000
N145 N 1 0.57940 0.33429 0.70960 1.0000
N146 N 1 0.08429 0.96100 0.20960 1.0000
N147 N 1 0.20611 0.46100 0.20960 1.0000
N148 N 1 0.07940 0.45611 0.70960 1.0000
N149 N 1 0.21100 0.83429 0.70960 1.0000
N150 N 1 0.71100 0.95611 0.70960 1.0000
N151 N 1 0.70611 0.82940 0.20960 1.0000
N152 N 1 0.58429 0.32940 0.20960 1.0000
N153 N 1 0.64931 0.34435 0.68883 1.0000
N154 N 1 0.09435 0.91186 0.18883 1.0000
N155 N 1 0.21681 0.41186 0.18883 1.0000
N156 N 1 0.14931 0.46681 0.68883 1.0000
N157 N 1 0.16186 0.84435 0.68883 1.0000
N158 N 1 0.66186 0.96681 0.68883 1.0000
N159 N 1 0.71681 0.89931 0.18883 1.0000
N160 N 1 0.59435 0.39931 0.18883 1.0000
Zn1 Zn 1 0.33584 0.13157 0.00386 1.0000
Zn2 Zn 1 0.88157 0.91030 0.50386 1.0000
Zn3 Zn 1 0.11457 0.41030 0.50386 1.0000
Zn4 Zn 1 0.83584 0.36457 0.00386 1.0000
Zn5 Zn 1 0.16030 0.63157 0.00386 1.0000
Zn6 Zn 1 0.66030 0.86457 0.00386 1.0000
Zn7 Zn 1 0.61457 0.58584 0.50386 1.0000
Zn8 Zn 1 0.38157 0.08584 0.50386 1.0000
Zn9 Zn 1 0.44521 0.91477 0.89758 1.0000
Zn10 Zn 1 0.66477 0.90721 0.39758 1.0000
Zn11 Zn 1 0.43765 0.40721 0.39758 1.0000
Zn12 Zn 1 0.94521 0.68765 0.89758 1.0000
Zn13 Zn 1 0.15721 0.41477 0.89758 1.0000
Zn14 Zn 1 0.65721 0.18765 0.89758 1.0000
Zn15 Zn 1 0.93765 0.69521 0.39758 1.0000
Zn16 Zn 1 0.16477 0.19521 0.39758 1.0000
#End

data_6-coi_Zn
_audit_creation_method TososPro
_Chemical_Name_Systematic IMIDZB07
_cell_length_a 21.17089
_cell_length_b 21.17089
_cell_length_c 16.36057
_cell_angle_alpha 49.6835
_cell_angle_beta 49.6835
_cell_angle_gamma 90
_cell_volume 2958.337
_cell_formula_units_Z 16
_symmetry_space_group_name_H-M 'P 1'
_symmetry_Int_Tables_number 1
loop_
_symmetry_equiv_pos_site_id
_symmetry_equiv_pos_as_xyz
1 x,y,z
loop_
3 1/2+x,1/2-y,-z
4 -x,1/2+y,1/2-z
5 -x,-y,-z
6 1/2+x,y,1/2-z
7 1/2-x,1/2+y,z
8 x,1/2-y,1/2+z
loop_
_atom_site_label
_atom_site_type_symbol
_atom_site_symmetry_multiplicity
_atom_site_fract_x
_atom_site_fract_y
_atom_site_fract_z
_atom_site_occupancy
N1 N 8 0.73222 0.75156 0.93160 1.0000
N2 N 8 0.64330 0.58162 0.93784 1.0000
N3 N 8 0.54819 0.65293 0.97898 1.0000
N4 N 8 0.75718 0.64276 0.90806 1.0000
N5 N 8 0.60380 0.75856 0.97516 1.0000
N6 N 8 0.83817 0.42871 0.74015 1.0000
N7 N 8 0.86648 0.38314 0.66596 1.0000
N8 N 8 0.70547 0.38631 0.76313 1.0000
N9 N 8 0.75194 0.31261 0.64328 1.0000
N10 N 8 0.65183 0.31432 0.70378 1.0000
Cd1 Cd 8 0.90070 0.89517 0.89567 1.0000
#End

data_8-sql_Cd
_audit_creation_method ToposPro
_Chemical_Name_Systematic BOJXAZ
_cell_length_a 9.952336
_cell_length_b 12.5841
_cell_length_c 11.5951
_cell_angle_alpha 90
_cell_angle_beta 90
_cell_angle_gamma 90
_cell_volume 1452.184
_cell_formula_units_Z 8
_symmetry_space_group_name_H-M 'P b c a'
_symmetry_Int_Tables_number 61
loop_
_symmetry_equiv_pos_site_id
_symmetry_equiv_pos_as_xyz
1 x,y,z
2 1/2-x,-y,1/2+z
3 1/2+x,1/2-y,-z
4 -x,1/2+y,1/2-z
5 -x,-y,-z
6 1/2+x,y,1/2-z
7 1/2-x,1/2+y,z
8 x,1/2-y,1/2+z
loop_
_atom_site_label
_atom_site_type_symbol
_atom_site_symmetry_multiplicity
_atom_site_fract_x
_atom_site_fract_y
_atom_site_fract_z
_atom_site_occupancy
N1 N 8 0.27111 0.19595 0.01839 1.0000
N2 N 8 0.22522 0.28196 0.06808 1.0000
N3 N 8 0.27295 0.36079 0.00769 1.0000
N4 N 8 0.34811 0.32365 0.92180 1.0000
N5 N 8 0.34705 0.22132 0.92834 1.0000
N6 N 8 0.02754 0.00453 0.16852 1.0000
N7 N 8 0.92389 0.95540 0.12038 1.0000
N8 N 8 0.82661 0.95545 0.19463 1.0000
N9 N 8 0.87068 0.00445 0.28740 1.0000
N10 N 8 0.99534 0.03477 0.27205 1.0000
Cd1 Cd 8 0.21812 0.03204 0.06760 1.0000
#End

data_9-New_Cd
_audit_creation_method ToposPro
_Chemical_Name_Systematic CUIMDZ03
_cell_length_a 12.57358
_cell_length_b 12.57358
_cell_length_c 10.28828
_cell_angle_alpha 96.72903
_cell_angle_beta 96.72903
_cell_angle_gamma 132.8723
_cell_volume 1139.681
_cell_formula_units_Z 6
_symmetry_space_group_name_H-M 'P 1'
_symmetry_Int_Tables_number 1
loop_
_symmetry_equiv_pos_site_id
_symmetry_equiv_pos_as_xyz
1 x,y,z
loop_
_atom_site_label
_atom_site_type_symbol
_atom_site_symmetry_multiplicity
_atom_site_fract_x
_atom_site_fract_y
_atom_site_fract_z
_atom_site_occupancy
N1 N 1 0.08794 0.58179 0.22608 1.0000
N2 N 1 0.41821 0.91206 0.27392 1.0000
N3 N 1 0.91206 0.41821 0.77392 1.0000
N4 N 1 0.58179 0.08794 0.72608 1.0000

_atom_site_label
_atom_site_type_symbol
_atom_site_symmetry_multiplicity
_atom_site_fract_x
_atom_site_fract_y
_atom_site_fract_z
_atom_site_occupancy
N1 N 1 0.70359 0.10388 0.53409 1.0000
N2 N 1 0.85388 0.01232 0.03409 1.0000
N3 N 1 0.76232 0.36203 0.53409 1.0000
N4 N 1 0.11203 0.95359 0.03409 1.0000
N5 N 1 0.36491 0.59967 0.80812 1.0000
N6 N 1 0.34967 0.07696 0.30812 1.0000
N7 N 1 0.82696 0.59220 0.80812 1.0000
N8 N 1 0.34220 0.61491 0.30812 1.0000
N9 N 1 0.85823 0.22801 0.22264 1.0000
N10 N 1 0.97800 0.16912 0.72264 1.0000
N11 N 1 0.91912 0.54935 0.22264 1.0000
N12 N 1 0.29935 0.10823 0.72264 1.0000
N13 N 1 0.43560 0.54277 0.85804 1.0000
N14 N 1 0.29277 0.95636 0.35804 1.0000
N15 N 1 0.70636 0.59919 0.85804 1.0000
N16 N 1 0.34919 0.68560 0.35804 1.0000
N17 N 1 0.77506 0.21384 0.27396 1.0000
N18 N 1 0.96384 0.20099 0.77396 1.0000
N19 N 1 0.95099 0.51220 0.27396 1.0000
N20 N 1 0.26220 0.02506 0.77396 1.0000
N21 N 1 0.44792 0.53990 0.77264 1.0000
N22 N 1 0.28990 0.02945 0.27264 1.0000
N23 N 1 0.77945 0.68747 0.77264 1.0000
N24 N 1 0.43747 0.69792 0.27264 1.0000
N25 N 1 0.89315 0.23935 0.47805 1.0000
N26 N 1 0.98935 0.87880 0.97805 1.0000
N27 N 1 0.62880 0.28259 0.47805 1.0000
N28 N 1 0.03259 0.14315 0.97805 1.0000
N29 N 1 0.39990 0.77475 0.49476 1.0000
N30 N 1 0.52475 0.35534 0.99476 1.0000
N31 N 1 0.10534 0.73049 0.49476 1.0000
N32 N 1 0.48049 0.64990 0.99476 1.0000
N33 N 1 0.80018 0.05571 0.72075 1.0000
N34 N 1 0.80571 0.72907 0.22075 1.0000
N35 N 1 0.47907 0.22353 0.72075 1.0000
N36 N 1 0.97353 0.05018 0.22075 1.0000
N37 N 1 0.21462 0.67822 0.72304 1.0000
N38 N 1 0.42822 0.31234 0.22304 1.0000
N39 N 1 0.06234 0.59874 0.72304 1.0000
N40 N 1 0.34874 0.46462 0.22304 1.0000
N41 N 1 0.79037 0.08641 0.77229 1.0000
N42 N 1 0.83641 0.68734 0.27229 1.0000
N43 N 1 0.43734 0.14130 0.77229 1.0000
N44 N 1 0.89130 0.04037 0.27229 1.0000
N45 N 1 0.22196 0.60857 0.74846 1.0000
N46 N 1 0.35857 0.27958 0.24846 1.0000
N47 N 1 0.02958 0.64296 0.74846 1.0000
N48 N 1 0.39296 0.47196 0.24846 1.0000
N49 N 1 0.15677 0.36337 0.00942 1.0000
N50 N 1 0.11337 0.08381 0.50942 1.0000
N51 N 1 0.83381 0.62721 0.00942 1.0000
N52 N 1 0.37721 0.40677 0.50942 1.0000
N53 N 1 0.35253 0.84099 0.03776 1.0000
N54 N 1 0.59099 0.85971 0.53776 1.0000
N55 N 1 0.60971 0.12125 0.03776 1.0000
N56 N 1 0.87125 0.60253 0.53776 1.0000
N57 N 1 0.11259 0.28883 0.98739 1.0000
N58 N 1 0.03883 0.15002 0.48739 1.0000
N59 N 1 0.90002 0.72378 0.98739 1.0000
N60 N 1 0.47378 0.36259 0.48739 1.0000
N61 N 1 0.18580 0.75576 0.19202 1.0000
N62 N 1 0.50576 0.87218 0.69202 1.0000
N63 N 1 0.62218 0.05222 0.19202 1.0000
N64 N 1 0.80222 0.43580 0.69202 1.0000
N65 N 1 0.26802 0.86120 0.00404 1.0000
N66 N 1 0.61120 0.97794 0.50404 1.0000
N67 N 1 0.72794 0.13476 0.00404 1.0000
N68 N 1 0.88476 0.51802 0.50404 1.0000
N69 N 1 0.22445 0.38789 0.81088 1.0000
N70 N 1 0.13789 0.21466 0.31088 1.0000
N71 N 1 0.96466 0.80122 0.81088 1.0000
N72 N 1 0.55122 0.47445 0.31088 1.0000
N73 N 1 0.72514 0.89291 0.74140 1.0000
N74 N 1 0.64291 0.78346 0.24140 1.0000
N75 N 1 0.53346 0.36569 0.74140 1.0000
N76 N 1 0.11569 0.97514 0.24140 1.0000
N77 N 1 0.60549 0.25364 0.28545 1.0000
N78 N 1 0.00364 0.35907 0.78545 1.0000
N79 N 1 0.10907 0.46091 0.28545 1.0000
N80 N 1 0.21091 0.85549 0.78545 1.0000
N81 N 1 0.89135 0.95525 0.58502 1.0000
N82 N 1 0.70525 0.77363 0.08502 1.0000
N83 N 1 0.52363 0.45973 0.58502 1.0000
N84 N 1 0.20973 0.14135 0.08502 1.0000
N85 N 1 0.53729 0.15616 0.31020 1.0000
N86 N 1 0.90616 0.40251 0.81020 1.0000
N87 N 1 0.15251 0.53364 0.31020 1.0000
N88 N 1 0.28364 0.78729 0.81020 1.0000
N89 N 1 0.50012 0.08137 0.46140 1.0000
N90 N 1 0.83137 0.28848 0.96140 1.0000
N91 N 1 0.03848 0.45723 0.46140 1.0000
N92 N 1 0.20723 0.75012 0.96140 1.0000
N93 N 1 0.81001 0.84036 0.75269 1.0000
N94 N 1 0.59036 0.68730 0.25269 1.0000
N5 N 1 0.93257 0.59249 0.24440 1.0000
N6 N 1 0.40751 0.06743 0.25560 1.0000
N7 N 1 0.06743 0.40751 0.75560 1.0000
N8 N 1 0.59249 0.93257 0.74440 1.0000
N9 N 1 0.07214 0.66359 0.29276 1.0000
N10 N 1 0.33641 0.92786 0.20724 1.0000
N11 N 1 0.92786 0.33641 0.70724 1.0000
N12 N 1 0.66359 0.07214 0.79276 1.0000
N13 N 1 0.95863 0.46058 0.13708 1.0000
N14 N 1 0.53942 0.04137 0.36292 1.0000
N15 N 1 0.04137 0.53942 0.86292 1.0000
N16 N 1 0.46058 0.95863 0.63708 1.0000
N17 N 1 0.86160 0.46680 0.14830 1.0000
N18 N 1 0.53320 0.13840 0.35170 1.0000
N19 N 1 0.13840 0.53320 0.85170 1.0000
N20 N 1 0.46680 0.86160 0.64830 1.0000
N21 N 1 0.81236 0.79850 0.44888 1.0000
N22 N 1 0.20150 0.18764 0.05112 1.0000
N23 N 1 0.18764 0.20150 0.55112 1.0000
N24 N 1 0.79850 0.81236 0.94888 1.0000
N25 N 1 0.79971 0.87241 0.63267 1.0000
N26 N 1 0.12759 0.20029 0.86733 1.0000
N27 N 1 0.20029 0.12759 0.36733 1.0000
N28 N 1 0.87241 0.79971 0.13267 1.0000
N29 N 1 0.84466 0.92267 0.44579 1.0000
N30 N 1 0.07733 0.15534 0.05421 1.0000
N31 N 1 0.15534 0.07733 0.55421 1.0000
N32 N 1 0.92267 0.84466 0.94579 1.0000
N33 N 1 0.78416 0.76684 0.56401 1.0000
N34 N 1 0.23316 0.21584 0.93599 1.0000
N35 N 1 0.21584 0.23316 0.43599 1.0000
N36 N 1 0.76684 0.78416 0.06401 1.0000
N37 N 1 0.83689 0.96885 0.56021 1.0000
N38 N 1 0.03115 0.16311 0.93979 1.0000
N39 N 1 0.16311 0.03115 0.43979 1.0000
N40 N 1 0.96885 0.83689 0.06021 1.0000
N41 N 1 0.34608 0.59498 0.41411 1.0000
N42 N 1 0.40502 0.65392 0.08589 1.0000
N43 N 1 0.65392 0.40502 0.58589 1.0000
N44 N 1 0.59498 0.34608 0.91411 1.0000
N45 N 1 0.31541 0.52131 0.58982 1.0000
N46 N 1 0.47869 0.68459 0.91018 1.0000
N47 N 1 0.68459 0.47869 0.41018 1.0000
N48 N 1 0.52131 0.31541 0.08982 1.0000
N49 N 1 0.24422 0.51002 0.47882 1.0000
N50 N 1 0.48998 0.75578 0.02118 1.0000
N51 N 1 0.75578 0.48998 0.52118 1.0000
N52 N 1 0.51002 0.24422 0.97882 1.0000
N53 N 1 0.47883 0.65789 0.48411 1.0000
N54 N 1 0.34211 0.52117 0.01589 1.0000
N55 N 1 0.52117 0.34211 0.51589 1.0000
N56 N 1 0.65789 0.47883 0.98411 1.0000
N57 N 1 0.46003 0.61216 0.59368 1.0000
N58 N 1 0.38784 0.53997 0.90632 1.0000
N59 N 1 0.53997 0.38784 0.40632 1.0000
N60 N 1 0.61216 0.46003 0.09368 1.0000
Cd1 Cd 1 0.31955 0.68045 0.25000 1.0000
Cd2 Cd 1 0.68045 0.31955 0.75000 1.0000
Cd3 Cd 1 0.81929 0.66786 0.29073 1.0000
Cd4 Cd 1 0.33214 0.18071 0.20927 1.0000
Cd5 Cd 1 0.18071 0.33214 0.70927 1.0000
Cd6 Cd 1 0.66786 0.81929 0.79073 1.0000
#End

data_10-zni_Cd
_audit_creation_method TopósPro
_Chemical_Name_Systematic IMIDZB01
_cell_length_a 22.21789
_cell_length_b 22.21789
_cell_length_c 17.13957
_cell_angle_alpha 49.59803
_cell_angle_beta 49.59803
_cell_angle_gamma 90
_cell_volume 3382.301
_cell_formula_units_Z 16
_symmetry_space_group_name_H-M 'P 1'
_symmetry_Int_Tables_number 1
loop_
_symmetry_equiv_pos_site_id
_symmetry_equiv_pos_as_xyz
1 x,y,z
loop_
_atom_site_label
_atom_site_type_symbol
_atom_site_symmetry_multiplicity
_atom_site_fract_x
_atom_site_fract_y
_atom_site_fract_z
_atom_site_occupancy
N1 N 1 0.31863 0.02560 0.05110 1.0000
N2 N 1 0.77560 0.88026 0.55110 1.0000
N3 N 1 0.17329 0.38026 0.55110 1.0000
N4 N 1 0.81863 0.42329 0.05110 1.0000
N5 N 1 0.13026 0.52560 0.05110 1.0000
N6 N 1 0.63026 0.92329 0.05110 1.0000
N7 N 1 0.67329 0.56863 0.55110 1.0000
N8 N 1 0.27560 0.06863 0.55110 1.0000
N9 N 1 0.36788 0.95805 0.01006 1.0000
N10 N 1 0.70805 0.87205 0.51006 1.0000
N11 N 1 0.28189 0.37205 0.51006 1.0000
N12 N 1 0.86788 0.53189 0.01006 1.0000

N95 N 1 0.43730 0.40694 0.75269 1.0000
N96 N 1 0.15694 0.06001 0.25269 1.0000
N97 N 1 0.81386 0.16001 0.38342 1.0000
N98 N 1 0.91001 0.05271 0.88342 1.0000
N99 N 1 0.80271 0.45657 0.38342 1.0000
N100 N 1 0.20657 0.06386 0.88342 1.0000
N101 N 1 0.38418 0.57949 0.87983 1.0000
N102 N 1 0.32949 0.98599 0.37983 1.0000
N103 N 1 0.73599 0.54067 0.87983 1.0000
N104 N 1 0.29067 0.63418 0.37983 1.0000
N105 N 1 0.67992 0.13707 0.46604 1.0000
N106 N 1 0.88707 0.10404 0.96604 1.0000
N107 N 1 0.85404 0.39689 0.46604 1.0000
N108 N 1 0.14689 0.92992 0.96604 1.0000
N109 N 1 0.40430 0.57499 0.74213 1.0000
N110 N 1 0.32499 0.10356 0.24213 1.0000
N111 N 1 0.85356 0.68288 0.74213 1.0000
N112 N 1 0.43288 0.65430 0.24213 1.0000
N113 N 1 0.86382 0.15035 0.53908 1.0000
N114 N 1 0.90035 0.84709 0.03908 1.0000
N115 N 1 0.59709 0.31057 0.53908 1.0000
N116 N 1 0.06057 0.11382 0.03908 1.0000
N117 N 1 0.32447 0.78045 0.56653 1.0000
N118 N 1 0.53045 0.35900 0.06653 1.0000
N119 N 1 0.10900 0.65301 0.56653 1.0000
N120 N 1 0.40301 0.57447 0.06653 1.0000
N121 N 1 0.84781 0.19966 0.62236 1.0000
N122 N 1 0.94966 0.77983 0.12236 1.0000
N123 N 1 0.52983 0.17798 0.62236 1.0000
N124 N 1 0.92798 0.09781 0.12236 1.0000
N125 N 1 0.33627 0.66853 0.60735 1.0000
N126 N 1 0.41853 0.30639 0.10735 1.0000
N127 N 1 0.05639 0.72413 0.60735 1.0000
N128 N 1 0.47413 0.58627 0.10735 1.0000
N129 N 1 0.07143 0.27417 0.10922 1.0000
N130 N 1 0.02417 0.06934 0.60922 1.0000
N131 N 1 0.81934 0.61661 0.10922 1.0000
N132 N 1 0.36661 0.32143 0.60922 1.0000
N133 N 1 0.23816 0.74359 0.21211 1.0000
N134 N 1 0.49359 0.79972 0.71211 1.0000
N135 N 1 0.54972 0.04429 0.21211 1.0000
N136 N 1 0.79429 0.48816 0.71211 1.0000
N137 N 1 0.37072 0.91347 0.90932 1.0000
N138 N 1 0.66347 0.96996 0.40932 1.0000
N139 N 1 0.71996 0.17722 0.90932 1.0000
N140 N 1 0.92722 0.62072 0.40932 1.0000
N141 N 1 0.25101 0.43311 0.82567 1.0000
N142 N 1 0.18311 0.17332 0.32567 1.0000
N143 N 1 0.92332 0.74122 0.82567 1.0000
N144 N 1 0.49122 0.50101 0.32567 1.0000
N145 N 1 0.83852 0.98699 0.57875 1.0000
N146 N 1 0.73699 0.83274 0.07875 1.0000
N147 N 1 0.58274 0.43426 0.57875 1.0000
N148 N 1 0.18426 0.08852 0.07875 1.0000
N149 N 1 0.60239 0.26228 0.20201 1.0000
N150 N 1 0.01228 0.44560 0.70201 1.0000
N151 N 1 0.19560 0.53571 0.20201 1.0000
N152 N 1 0.28571 0.85239 0.70201 1.0000
N153 N 1 0.54221 0.14181 0.44557 1.0000
N154 N 1 0.89181 0.26222 0.94557 1.0000
N155 N 1 0.01222 0.41262 0.44557 1.0000
N156 N 1 0.16262 0.79221 0.94557 1.0000
N157 N 1 0.70773 0.80239 0.84855 1.0000
N158 N 1 0.55239 0.69372 0.34855 1.0000
N159 N 1 0.44372 0.34906 0.84855 1.0000
N160 N 1 0.09906 0.95773 0.34855 1.0000
Zn1 Zn 1 0.90368 0.14826 0.39168 1.0000
Zn2 Zn 1 0.89826 0.95464 0.89168 1.0000
Zn3 Zn 1 0.70464 0.46006 0.39168 1.0000
Zn4 Zn 1 0.21006 0.15368 0.89168 1.0000
Zn5 Zn 1 0.36218 0.62109 0.96980 1.0000
Zn6 Zn 1 0.37109 0.91802 0.46980 1.0000
Zn7 Zn 1 0.66802 0.40912 0.96980 1.0000
Zn8 Zn 1 0.15912 0.61218 0.46980 1.0000
Zn9 Zn 1 0.52504 0.08352 0.62076 1.0000
Zn10 Zn 1 0.83352 0.10420 0.12076 1.0000
Zn11 Zn 1 0.85420 0.29572 0.62076 1.0000
Zn12 Zn 1 0.04572 0.77504 0.12076 1.0000
Zn13 Zn 1 0.39381 0.59202 0.61574 1.0000
Zn14 Zn 1 0.34202 0.24045 0.11574 1.0000
Zn15 Zn 1 0.99045 0.79224 0.61574 1.0000
Zn16 Zn 1 0.54224 0.64381 0.11574 1.0000
#End

data_7-sql_Zn
_audit_creation_method ToposPro
_Chemical_Name_Systematic ONATUT
_cell_length_a 8.199304
_cell_length_b 10.6073
_cell_length_c 15.07382
_cell_angle_alpha 90
_cell_angle_beta 90
_cell_angle_gamma 90
_cell_volume 1311.007
_cell_formula_units_Z 8
_symmetry_space_group_name_H-M 'P b c a'
_symmetry_Int_Tables_number 61
loop_
_symmetry_equiv_pos_site_id
_symmetry_equiv_pos_as_xyz
1 x,y,z

N13 N 1 0.12205 0.45805 0.01006 1.0000
N14 N 1 0.62205 0.03189 0.01006 1.0000
N15 N 1 0.78189 0.61788 0.51006 1.0000
N16 N 1 0.20805 0.11788 0.51006 1.0000
N17 N 1 0.40244 0.05459 0.91701 1.0000
N18 N 1 0.80459 0.93056 0.41701 1.0000
N19 N 1 0.27840 0.43056 0.41701 1.0000
N20 N 1 0.90244 0.52840 0.91701 1.0000
N21 N 1 0.18056 0.55459 0.91701 1.0000
N22 N 1 0.68056 0.02840 0.91701 1.0000
N23 N 1 0.77840 0.65244 0.41701 1.0000
N24 N 1 0.30459 0.15244 0.41701 1.0000
N25 N 1 0.26336 0.87001 0.20050 1.0000
N26 N 1 0.62001 0.78615 0.70050 1.0000
N27 N 1 0.17949 0.28615 0.70050 1.0000
N28 N 1 0.76336 0.42949 0.20050 1.0000
N29 N 1 0.03615 0.37001 0.20050 1.0000
N30 N 1 0.53615 0.92949 0.20050 1.0000
N31 N 1 0.67949 0.51336 0.70050 1.0000
N32 N 1 0.12001 0.01336 0.70050 1.0000
N33 N 1 0.23295 0.91218 0.22574 1.0000
N34 N 1 0.66218 0.79131 0.72574 1.0000
N35 N 1 0.11208 0.29131 0.72574 1.0000
N36 N 1 0.73295 0.36208 0.22574 1.0000
N37 N 1 0.04131 0.41218 0.22574 1.0000
N38 N 1 0.54131 0.86208 0.22574 1.0000
N39 N 1 0.61208 0.48295 0.72574 1.0000
N40 N 1 0.16218 0.98295 0.72574 1.0000
N41 N 1 0.15047 0.99578 0.33461 1.0000
N42 N 1 0.74578 0.76491 0.83461 1.0000
N43 N 1 0.91961 0.26491 0.83461 1.0000
N44 N 1 0.65047 0.16961 0.33461 1.0000
N45 N 1 0.01491 0.49578 0.33461 1.0000
N46 N 1 0.51491 0.66961 0.33461 1.0000
N47 N 1 0.41961 0.40047 0.83461 1.0000
N48 N 1 0.24578 0.90047 0.83461 1.0000
N49 N 1 0.03718 0.83688 0.61843 1.0000
N50 N 1 0.58688 0.59439 0.11843 1.0000
N51 N 1 0.79469 0.09439 0.11843 1.0000
N52 N 1 0.53718 0.04469 0.61843 1.0000
N53 N 1 0.84439 0.33688 0.61843 1.0000
N54 N 1 0.34439 0.54469 0.61843 1.0000
N55 N 1 0.29469 0.28718 0.11843 1.0000
N56 N 1 0.08688 0.78718 0.11843 1.0000
N57 N 1 0.14579 0.93229 0.43922 1.0000
N58 N 1 0.68229 0.66499 0.93922 1.0000
N59 N 1 0.87849 0.16499 0.93922 1.0000
N60 N 1 0.64579 0.12849 0.43922 1.0000
N61 N 1 0.91499 0.43229 0.43922 1.0000
N62 N 1 0.41499 0.62849 0.43922 1.0000
N63 N 1 0.37849 0.39579 0.93922 1.0000
N64 N 1 0.18229 0.89579 0.93922 1.0000
N65 N 1 0.97468 0.84070 0.62572 1.0000
N66 N 1 0.59070 0.64959 0.12572 1.0000
N67 N 1 0.78357 0.14959 0.12572 1.0000
N68 N 1 0.47468 0.03357 0.62572 1.0000
N69 N 1 0.89959 0.34070 0.62572 1.0000
N70 N 1 0.39959 0.53357 0.62572 1.0000
N71 N 1 0.28357 0.22468 0.12572 1.0000
N72 N 1 0.09070 0.72468 0.12572 1.0000
N73 N 1 0.32870 0.24813 0.83214 1.0000
N74 N 1 0.99813 0.08916 0.33214 1.0000
N75 N 1 0.16973 0.58916 0.33214 1.0000
N76 N 1 0.82870 0.41973 0.83214 1.0000
N77 N 1 0.33916 0.74813 0.83214 1.0000
N78 N 1 0.83916 0.91973 0.83214 1.0000
N79 N 1 0.66973 0.57870 0.33214 1.0000
N80 N 1 0.49813 0.07870 0.33214 1.0000
N81 N 1 0.36956 0.33954 0.62037 1.0000
N82 N 1 0.08954 0.26007 0.12037 1.0000
N83 N 1 0.29008 0.76007 0.12037 1.0000
N84 N 1 0.86956 0.54008 0.62037 1.0000
N85 N 1 0.51007 0.83954 0.62037 1.0000
N86 N 1 0.01007 0.04008 0.62037 1.0000
N87 N 1 0.79008 0.61956 0.12037 1.0000
N88 N 1 0.58954 0.11956 0.12037 1.0000
N89 N 1 0.04490 0.93936 0.44963 1.0000
N90 N 1 0.68936 0.75547 0.94963 1.0000
N91 N 1 0.86101 0.25547 0.94963 1.0000
N92 N 1 0.54490 0.11101 0.44963 1.0000
N93 N 1 0.00547 0.43936 0.44963 1.0000
N94 N 1 0.50547 0.61101 0.44963 1.0000
N95 N 1 0.36101 0.29490 0.94963 1.0000
N96 N 1 0.18936 0.79490 0.94963 1.0000
N97 N 1 0.37490 0.27362 0.70314 1.0000
N98 N 1 0.02362 0.17197 0.20314 1.0000
N99 N 1 0.27325 0.67197 0.20314 1.0000
N100 N 1 0.87490 0.52325 0.70314 1.0000
N101 N 1 0.42197 0.77362 0.70314 1.0000
N102 N 1 0.92197 0.02325 0.70314 1.0000
N103 N 1 0.77325 0.62490 0.20314 1.0000
N104 N 1 0.52362 0.12490 0.20314 1.0000
N105 N 1 0.29484 0.29777 0.82958 1.0000
N106 N 1 0.04777 0.12558 0.32958 1.0000
N107 N 1 0.12265 0.62558 0.32958 1.0000
N108 N 1 0.79484 0.37265 0.82958 1.0000
N109 N 1 0.37558 0.79777 0.82958 1.0000
N110 N 1 0.87558 0.87265 0.82958 1.0000
N111 N 1 0.62265 0.54484 0.32958 1.0000
N112 N 1 0.54777 0.04484 0.32958 1.0000
N113 N 1 0.58998 0.23338 0.84128 1.0000

2 1/2-x,-y,1/2+z
3 1/2+x,1/2-y,-z
4 -x,1/2+y,1/2-z
5 -x,-y,-z
6 1/2+x,y,1/2-z
7 1/2-x,1/2+y,z
8 x,1/2-y,1/2+z
loop_
_atom_site_label
_atom_site_type_symbol
_atom_site_symmetry_multiplicity
_atom_site_fract_x
_atom_site_fract_y
_atom_site_fract_z
_atom_site_occupancy
N1 N 8 0.74351 0.75477 0.92883 1.0000
N2 N 8 0.64703 0.57378 0.93501 1.0000
N3 N 8 0.54554 0.65120 0.97736 1.0000
N4 N 8 0.76960 0.63809 0.90471 1.0000
N5 N 8 0.60556 0.76369 0.97313 1.0000
N6 N 8 0.84455 0.43917 0.73940 1.0000
N7 N 8 0.87636 0.38400 0.66365 1.0000
N8 N 8 0.70623 0.39302 0.76574 1.0000
N9 N 8 0.75750 0.30391 0.64357 1.0000
N10 N 8 0.65208 0.30901 0.70679 1.0000
Zn1 Zn 8 0.90045 0.89538 0.89490 1.0000
#End

data_8-sql2_Zn

_audit_creation_method TopospPro
_Chemical_Name_Systematic BOJXAZ
_cell_length_a 9.612414
_cell_length_b 11.39182
_cell_length_c 11.91699
_cell_angle_alpha 90
_cell_angle_beta 90
_cell_angle_gamma 90
_cell_volume 1304.946
_cell_formula_units_Z 8
_symmetry_space_group_name_H-M 'P b c a'
_symmetry_Int_Tables_number 61
loop_
_symmetry_equiv_pos_site_id
_symmetry_equiv_pos_as_xyz
1 x,y,z
2 1/2-x,-y,1/2+z
3 1/2+x,1/2-y,-z
4 -x,1/2+y,1/2-z
5 -x,-y,-z
6 1/2+x,y,1/2-z
7 1/2-x,1/2+y,z
8 x,1/2-y,1/2+z
loop_
_atom_site_label
_atom_site_type_symbol
_atom_site_symmetry_multiplicity
_atom_site_fract_x
_atom_site_fract_y
_atom_site_fract_z
_atom_site_occupancy
N1 N 8 0.25802 0.19375 0.03557 1.0000
N2 N 8 0.20762 0.28726 0.08387 1.0000
N3 N 8 0.25542 0.37561 0.02591 1.0000
N4 N 8 0.33499 0.33621 0.94241 1.0000
N5 N 8 0.33704 0.22361 0.94848 1.0000
N6 N 8 0.04534 0.01232 0.17263 1.0000
N7 N 8 0.95281 0.93783 0.12990 1.0000
N8 N 8 0.84555 0.93987 0.19446 1.0000
N9 N 8 0.87234 0.01528 0.27633 1.0000
N10 N 8 0.99622 0.06039 0.26341 1.0000
Zn1 Zn 8 0.23163 0.03610 0.09930 1.0000
#End

data_9-sql3_Zn
_audit_creation_method TopospPro
_Chemical_Name_Systematic AXIVAF
_cell_length_a 9.298202
_cell_length_b 11.04785
_cell_length_c 13.83197
_cell_angle_alpha 90
_cell_angle_beta 90
_cell_angle_gamma 90
_cell_volume 1420.891
_cell_formula_units_Z 8
_symmetry_space_group_name_H-M 'P b c a'
_symmetry_Int_Tables_number 61
loop_
_symmetry_equiv_pos_site_id
_symmetry_equiv_pos_as_xyz
1 x,y,z
2 1/2-x,-y,1/2+z
3 1/2+x,1/2-y,-z
4 -x,1/2+y,1/2-z
5 -x,-y,-z
6 1/2+x,y,1/2-z
7 1/2-x,1/2+y,z
8 x,1/2-y,1/2+z
loop_
_atom_site_label
_atom_site_type_symbol

N114 N 1 0.98338 0.81874 0.34128 1.0000
N115 N 1 0.17535 0.31874 0.34128 1.0000
N116 N 1 0.08998 0.42535 0.84128 1.0000
N117 N 1 0.06874 0.73338 0.84128 1.0000
N118 N 1 0.56874 0.92535 0.84128 1.0000
N119 N 1 0.67535 0.83998 0.34128 1.0000
N120 N 1 0.48338 0.33998 0.34128 1.0000
N121 N 1 0.47839 0.22304 0.87993 1.0000
N122 N 1 0.97304 0.89168 0.37993 1.0000
N123 N 1 0.14703 0.39168 0.37993 1.0000
N124 N 1 0.97839 0.39703 0.87993 1.0000
N125 N 1 0.14168 0.72304 0.87993 1.0000
N126 N 1 0.64168 0.89703 0.87993 1.0000
N127 N 1 0.64703 0.72839 0.37993 1.0000
N128 N 1 0.47304 0.22839 0.37993 1.0000
N129 N 1 0.32034 0.35478 0.69747 1.0000
N130 N 1 0.10478 0.23219 0.19747 1.0000
N131 N 1 0.19775 0.73219 0.19747 1.0000
N132 N 1 0.82034 0.44775 0.69747 1.0000
N133 N 1 0.48219 0.85478 0.69747 1.0000
N134 N 1 0.98219 0.94775 0.69747 1.0000
N135 N 1 0.69775 0.57034 0.19747 1.0000
N136 N 1 0.60478 0.07034 0.19747 1.0000
N137 N 1 0.49217 0.16159 0.94648 1.0000
N138 N 1 0.91159 0.81136 0.44648 1.0000
N139 N 1 0.14193 0.31136 0.44648 1.0000
N140 N 1 0.99217 0.39193 0.94648 1.0000
N141 N 1 0.06136 0.66159 0.94648 1.0000
N142 N 1 0.56136 0.89193 0.94648 1.0000
N143 N 1 0.64193 0.74217 0.44648 1.0000
N144 N 1 0.41159 0.24217 0.44648 1.0000
N145 N 1 0.56696 0.33216 0.73443 1.0000
N146 N 1 0.08216 0.94861 0.23443 1.0000
N147 N 1 0.18341 0.44861 0.23443 1.0000
N148 N 1 0.06696 0.43341 0.73443 1.0000
N149 N 1 0.19861 0.83216 0.73443 1.0000
N150 N 1 0.69861 0.93341 0.73443 1.0000
N151 N 1 0.68341 0.81696 0.23443 1.0000
N152 N 1 0.58216 0.31696 0.23443 1.0000
N153 N 1 0.63651 0.33874 0.71015 1.0000
N154 N 1 0.08874 0.90333 0.21015 1.0000
N155 N 1 0.20111 0.40333 0.21015 1.0000
N156 N 1 0.13651 0.45111 0.71015 1.0000
N157 N 1 0.15333 0.83874 0.71015 1.0000
N158 N 1 0.65333 0.95111 0.71015 1.0000
N159 N 1 0.70111 0.88651 0.21015 1.0000
N160 N 1 0.58874 0.38651 0.21015 1.0000
Cd1 Cd 1 0.32148 0.14730 0.00886 1.0000
Cd2 Cd 1 0.89730 0.91966 0.50886 1.0000
Cd3 Cd 1 0.09384 0.41966 0.50886 1.0000
Cd4 Cd 1 0.82148 0.34384 0.00886 1.0000
Cd5 Cd 1 0.16966 0.64730 0.00886 1.0000
Cd6 Cd 1 0.66966 0.84384 0.00886 1.0000
Cd7 Cd 1 0.59384 0.57148 0.50886 1.0000
Cd8 Cd 1 0.39730 0.07148 0.50886 1.0000
Cd9 Cd 1 0.45071 0.92883 0.89007 1.0000
Cd10 Cd 1 0.67883 0.90922 0.39007 1.0000
Cd11 Cd 1 0.43110 0.40922 0.39007 1.0000
Cd12 Cd 1 0.95071 0.68110 0.89007 1.0000
Cd13 Cd 1 0.15922 0.42883 0.89007 1.0000
Cd14 Cd 1 0.65922 0.18110 0.89007 1.0000
Cd15 Cd 1 0.93110 0.70071 0.39007 1.0000
Cd16 Cd 1 0.17883 0.20071 0.39007 1.0000
#End

data_11-new2_Cd
_audit_creation_method TopospPro
_Chemical_Name_Systematic IMIDZB07
_cell_length_a 20.78823
_cell_length_b 20.78823
_cell_length_c 16.37886
_cell_angle_alpha 50.60928
_cell_angle_beta 50.60928
_cell_angle_gamma 90
_cell_volume 3122.028
_cell_formula_units_Z 16
_symmetry_space_group_name_H-M 'P 1'
_symmetry_Int_Tables_number 1
loop_
_symmetry_equiv_pos_site_id
_symmetry_equiv_pos_as_xyz
1 x,y,z
loop_
_atom_site_label
_atom_site_type_symbol
_atom_site_symmetry_multiplicity
_atom_site_fract_x
_atom_site_fract_y
_atom_site_fract_z
_atom_site_occupancy
N1 N 1 0.69242 0.11873 0.51255 1.0000
N2 N 1 0.86873 0.04503 0.01255 1.0000
N3 N 1 0.79503 0.36872 0.51255 1.0000
N4 N 1 0.11872 0.94242 0.01255 1.0000
N5 N 1 0.41756 0.59899 0.74655 1.0000
N6 N 1 0.34899 0.08590 0.24655 1.0000
N7 N 1 0.83590 0.65446 0.74655 1.0000
N8 N 1 0.40446 0.66756 0.24655 1.0000
N9 N 1 0.83478 0.19896 0.25272 1.0000
N10 N 1 0.94896 0.16251 0.75272 1.0000
N11 N 1 0.91251 0.54832 0.25272 1.0000

_atom_site_symmetry_multiplicity
_atom_site_fract_x
_atom_site_fract_y
_atom_site_fract_z
_atom_site_occupancy
N1 N 8 0.11054 0.68659 0.00612 1.0000
N2 N 8 0.14833 0.79868 0.00765 1.0000
N3 N 8 0.25772 0.80784 0.06918 1.0000
N4 N 8 0.19680 0.62723 0.06677 1.0000
N5 N 8 0.28764 0.70231 0.10629 1.0000
N6 N 8 0.85789 0.53740 0.12209 1.0000
N7 N 8 0.96019 0.47409 0.16650 1.0000
N8 N 8 0.74672 0.53693 0.17788 1.0000
N9 N 8 0.91339 0.43423 0.24927 1.0000
N10 N 8 0.78130 0.47317 0.25588 1.0000
Zn1 Zn 8 0.16259 0.46082 0.12061 1.0000
#End

data_10-yqt1_Zn
_audit_creation_method ToposPro
_Chemical_Name_Systematic GUPBOJ
_cell_length_a 10.31429
_cell_length_b 10.31429
_cell_length_c 12.41281
_cell_angle_alpha 107.678
_cell_angle_beta 107.678
_cell_angle_gamma 96.06631
_cell_volume 1169.93
_cell_formula_units_Z 6
_symmetry_space_group_name_H-M 'P 1'
_symmetry_Int_Tables_number 1
loop_
_symmetry_equiv_pos_site_id
_symmetry_equiv_pos_as_xyz
1 x,y,z
loop_
_atom_site_label
_atom_site_type_symbol
_atom_site_symmetry_multiplicity
_atom_site_fract_x
_atom_site_fract_y
_atom_site_fract_z
_atom_site_occupancy
N1 N 1 0.09831 0.11160 0.37564 1.0000
N2 N 1 0.88840 0.90169 0.12436 1.0000
N3 N 1 0.89875 0.88768 0.62237 1.0000
N4 N 1 0.11232 0.10125 0.87763 1.0000
N5 N 1 0.12270 0.32532 0.45325 1.0000
N6 N 1 0.67468 0.87730 0.04675 1.0000
N7 N 1 0.85940 0.67289 0.54256 1.0000
N8 N 1 0.32711 0.14060 0.95744 1.0000
N9 N 1 0.12056 0.60782 0.37381 1.0000
N10 N 1 0.39218 0.87944 0.12619 1.0000
N11 N 1 0.89770 0.39218 0.62868 1.0000
N12 N 1 0.60782 0.10230 0.87132 1.0000
N13 N 1 0.06519 0.75391 0.29471 1.0000
N14 N 1 0.24609 0.93481 0.20529 1.0000
N15 N 1 0.94449 0.24579 0.70909 1.0000
N16 N 1 0.75421 0.05551 0.79091 1.0000
N17 N 1 0.24598 0.65574 0.65737 1.0000
N18 N 1 0.34426 0.75402 0.84263 1.0000
N19 N 1 0.75191 0.34830 0.34250 1.0000
N20 N 1 0.65170 0.24809 0.15750 1.0000
N21 N 1 0.40087 0.47313 0.50823 1.0000
N22 N 1 0.52687 0.59913 0.99177 1.0000
N23 N 1 0.60330 0.45603 0.52408 1.0000
N24 N 1 0.54397 0.39670 0.97592 1.0000
N25 N 1 0.11926 0.22787 0.35606 1.0000
N26 N 1 0.77213 0.88074 0.14394 1.0000
N27 N 1 0.86295 0.77205 0.63862 1.0000
N28 N 1 0.22795 0.13705 0.86138 1.0000
N29 N 1 0.08934 0.13680 0.48451 1.0000
N30 N 1 0.86320 0.91066 0.01549 1.0000
N31 N 1 0.91714 0.86038 0.51685 1.0000
N32 N 1 0.13962 0.08286 0.98315 1.0000
N33 N 1 0.10522 0.26959 0.53272 1.0000
N34 N 1 0.73041 0.89478 0.96728 1.0000
N35 N 1 0.89255 0.72686 0.46713 1.0000
N36 N 1 0.27314 0.10745 0.03287 1.0000
N37 N 1 0.15937 0.73679 0.38364 1.0000
N38 N 1 0.26321 0.84063 0.11636 1.0000
N39 N 1 0.85657 0.26311 0.61690 1.0000
N40 N 1 0.73689 0.14343 0.88310 1.0000
N41 N 1 0.96853 0.63613 0.23002 1.0000
N42 N 1 0.36387 0.03147 0.26998 1.0000
N43 N 1 0.03966 0.36373 0.77799 1.0000
N44 N 1 0.63627 0.96034 0.72201 1.0000
N45 N 1 0.00275 0.54554 0.27915 1.0000
N46 N 1 0.45446 0.99725 0.22085 1.0000
N47 N 1 0.01055 0.45457 0.72785 1.0000
N48 N 1 0.54543 0.98945 0.77215 1.0000
N49 N 1 0.34650 0.65350 0.75000 1.0000
N50 N 1 0.65018 0.34982 0.25000 1.0000
N51 N 1 0.18170 0.75729 0.69239 1.0000
N52 N 1 0.24271 0.81830 0.80761 1.0000
N53 N 1 0.81536 0.24627 0.30744 1.0000
N54 N 1 0.75373 0.18464 0.19256 1.0000
N55 N 1 0.51158 0.52929 0.49547 1.0000
N56 N 1 0.47071 0.48842 0.00453 1.0000
N57 N 1 0.42392 0.36554 0.54452 1.0000
N58 N 1 0.63446 0.57608 0.95548 1.0000
N12 N 1 0.29832 0.08478 0.75272 1.0000
N13 N 1 0.33194 0.44219 0.00553 1.0000
N14 N 1 0.19219 0.91253 0.50553 1.0000
N15 N 1 0.66253 0.55228 0.00553 1.0000
N16 N 1 0.30228 0.58194 0.50553 1.0000
N17 N 1 0.74871 0.16216 0.30788 1.0000
N18 N 1 0.91216 0.19340 0.80788 1.0000
N19 N 1 0.94340 0.52995 0.30788 1.0000
N20 N 1 0.27995 0.99871 0.80788 1.0000
N21 N 1 0.33460 0.42568 0.93978 1.0000
N22 N 1 0.17568 0.97562 0.43978 1.0000
N23 N 1 0.72562 0.63454 0.93978 1.0000
N24 N 1 0.38454 0.58460 0.43978 1.0000
N25 N 1 0.90499 0.26219 0.49369 1.0000
N26 N 1 0.01219 0.85132 0.99369 1.0000
N27 N 1 0.60132 0.24412 0.49369 1.0000
N28 N 1 0.99412 0.15499 0.99369 1.0000
N29 N 1 0.41698 0.75971 0.44866 1.0000
N30 N 1 0.50971 0.38437 0.94866 1.0000
N31 N 1 0.13437 0.79163 0.44866 1.0000
N32 N 1 0.54163 0.66698 0.94866 1.0000
N33 N 1 0.77404 0.08086 0.73546 1.0000
N34 N 1 0.83086 0.74050 0.23546 1.0000
N35 N 1 0.49050 0.18368 0.73546 1.0000
N36 N 1 0.93368 0.02404 0.23546 1.0000
N37 N 1 0.24099 0.66966 0.68359 1.0000
N38 N 1 0.41966 0.32542 0.18359 1.0000
N39 N 1 0.07542 0.64674 0.68359 1.0000
N40 N 1 0.39674 0.49099 0.18359 1.0000
N41 N 1 0.77475 0.10808 0.78738 1.0000
N42 N 1 0.85808 0.68787 0.28738 1.0000
N43 N 1 0.43787 0.10454 0.78738 1.0000
N44 N 1 0.85454 0.02475 0.28738 1.0000
N45 N 1 0.24900 0.59587 0.72507 1.0000
N46 N 1 0.34587 0.27594 0.22507 1.0000
N47 N 1 0.02594 0.67907 0.72507 1.0000
N48 N 1 0.42907 0.49900 0.22507 1.0000
N49 N 1 0.16037 0.35262 0.99867 1.0000
N50 N 1 0.10262 0.09095 0.49867 1.0000
N51 N 1 0.84095 0.64871 0.99867 1.0000
N52 N 1 0.39871 0.41037 0.49867 1.0000
N53 N 1 0.35638 0.84764 0.01075 1.0000
N54 N 1 0.59764 0.88287 0.51075 1.0000
N55 N 1 0.63287 0.14161 0.01075 1.0000
N56 N 1 0.89161 0.60638 0.51075 1.0000
N57 N 1 0.06611 0.24130 0.06193 1.0000
N58 N 1 0.99130 0.12196 0.56193 1.0000
N59 N 1 0.87196 0.69676 0.06193 1.0000
N60 N 1 0.44676 0.31611 0.56193 1.0000
N61 N 1 0.18390 0.71477 0.21962 1.0000
N62 N 1 0.46477 0.84648 0.71962 1.0000
N63 N 1 0.59648 0.06560 0.21962 1.0000
N64 N 1 0.81560 0.43390 0.71962 1.0000
N65 N 1 0.23690 0.78604 0.06019 1.0000
N66 N 1 0.53604 0.95291 0.56019 1.0000
N67 N 1 0.70291 0.15377 0.06019 1.0000
N68 N 1 0.90377 0.48690 0.56019 1.0000
N69 N 1 0.17151 0.31173 0.90031 1.0000
N70 N 1 0.06173 0.17818 0.40031 1.0000
N71 N 1 0.92818 0.78796 0.90031 1.0000
N72 N 1 0.53796 0.42151 0.40031 1.0000
N73 N 1 0.75101 0.91177 0.73701 1.0000
N74 N 1 0.66177 0.76198 0.23701 1.0000
N75 N 1 0.51198 0.35122 0.73701 1.0000
N76 N 1 0.10122 0.00101 0.23701 1.0000
N77 N 1 0.58085 0.25015 0.27257 1.0000
N78 N 1 0.00015 0.39658 0.77257 1.0000
N79 N 1 0.14658 0.47728 0.27257 1.0000
N80 N 1 0.22728 0.83085 0.77257 1.0000
N81 N 1 0.92742 0.00261 0.51148 1.0000
N82 N 1 0.75261 0.81110 0.01148 1.0000
N83 N 1 0.56110 0.48591 0.51148 1.0000
N84 N 1 0.23591 0.17742 0.01148 1.0000
N85 N 1 0.50984 0.18536 0.26177 1.0000
N86 N 1 0.93536 0.47840 0.76177 1.0000
N87 N 1 0.22840 0.55287 0.26177 1.0000
N88 N 1 0.30287 0.75984 0.76177 1.0000
N89 N 1 0.47134 0.10037 0.40707 1.0000
N90 N 1 0.85037 0.37159 0.90707 1.0000
N91 N 1 0.12159 0.49255 0.40707 1.0000
N92 N 1 0.24255 0.72134 0.90707 1.0000
N93 N 1 0.87432 0.89809 0.65322 1.0000
N94 N 1 0.64809 0.72246 0.15322 1.0000
N95 N 1 0.47246 0.44869 0.65322 1.0000
N96 N 1 0.19869 0.12432 0.15322 1.0000
N97 N 1 0.79980 0.17193 0.37928 1.0000
N98 N 1 0.92193 0.07092 0.87928 1.0000
N99 N 1 0.82092 0.44879 0.37928 1.0000
N100 N 1 0.19879 0.04980 0.87928 1.0000
N101 N 1 0.38336 0.54899 0.88611 1.0000
N102 N 1 0.29899 0.98053 0.38611 1.0000
N103 N 1 0.73053 0.56490 0.88611 1.0000
N104 N 1 0.31490 0.63336 0.38611 1.0000
N105 N 1 0.66130 0.11291 0.46772 1.0000
N106 N 1 0.86291 0.12098 0.96772 1.0000
N107 N 1 0.87098 0.41937 0.46772 1.0000
N108 N 1 0.16937 0.91130 0.96772 1.0000
N109 N 1 0.38734 0.52233 0.78030 1.0000
N110 N 1 0.27233 0.08236 0.28030 1.0000
N111 N 1 0.83236 0.69737 0.78030 1.0000
N112 N 1 0.44737 0.63734 0.28030 1.0000

N59 N 1 0.54961 0.35515 0.55427 1.0000
N60 N 1 0.64485 0.45039 0.94573 1.0000
Zn1 Zn 1 0.21501 0.52372 0.49170 1.0000
Zn2 Zn 1 0.47628 0.78499 0.00830 1.0000
Zn3 Zn 1 0.79358 0.47419 0.51334 1.0000
Zn4 Zn 1 0.52581 0.20642 0.98666 1.0000
Zn5 Zn 1 0.07369 0.92631 0.25000 1.0000
Zn6 Zn 1 0.92747 0.07253 0.75000 1.0000
#End

data_11-zni2_Zn
_audit_creation_method ToposPro
_Chemical_Name_Systematic GITTAF
_cell_length_a 22.88506
_cell_length_b 22.88506
_cell_length_c 17.32761
_cell_angle_alpha 48.67238
_cell_angle_beta 48.67238
_cell_angle_gamma 90
_cell_volume 3244.704
_cell_formula_units_Z 16
_symmetry_space_group_name_H-M 'P 1'
_symmetry_Int_Tables_number 1
loop_
_symmetry_equiv_pos_site_id
_symmetry_equiv_pos_as_xyz
1 x,y,z
loop_
_atom_site_label
_atom_site_type_symbol
_atom_site_symmetry_multiplicity
_atom_site_fract_x
_atom_site_fract_y
_atom_site_fract_z
_atom_site_occupancy
N1 N 1 0.16305 0.75000 0.50000 1.0000
N2 N 1 0.25000 0.83695 0.00000 1.0000
N3 N 1 0.83695 0.25000 0.50000 1.0000
N4 N 1 0.33695 0.25000 0.50000 1.0000
N5 N 1 0.75000 0.16305 0.00000 1.0000
N6 N 1 0.25000 0.33695 0.00000 1.0000
N7 N 1 0.66305 0.75000 0.50000 1.0000
N8 N 1 0.75000 0.66305 0.00000 1.0000
N9 N 1 0.52574 0.09047 0.97672 1.0000
N10 N 1 0.90953 0.47426 0.52328 1.0000
N11 N 1 0.99755 0.59047 0.97672 1.0000
N12 N 1 0.49755 0.43281 0.97672 1.0000
N13 N 1 0.97426 0.06719 0.02328 1.0000
N14 N 1 0.09047 0.52574 0.47672 1.0000
N15 N 1 0.59047 0.99755 0.47672 1.0000
N16 N 1 0.06719 0.97426 0.52328 1.0000
N17 N 1 0.43281 0.49755 0.47672 1.0000
N18 N 1 0.00245 0.40953 0.02328 1.0000
N19 N 1 0.50245 0.56719 0.02328 1.0000
N20 N 1 0.02574 0.93281 0.97672 1.0000
N21 N 1 0.47426 0.90953 0.02328 1.0000
N22 N 1 0.40953 0.00245 0.52328 1.0000
N23 N 1 0.93281 0.02574 0.47672 1.0000
N24 N 1 0.56719 0.50245 0.52328 1.0000
N25 N 1 0.50378 0.25378 0.75000 1.0000
N26 N 1 0.74622 0.49622 0.75000 1.0000
N27 N 1 0.24622 0.75378 0.75000 1.0000
N28 N 1 0.99622 0.00378 0.25000 1.0000
N29 N 1 0.25378 0.50378 0.25000 1.0000
N30 N 1 0.75378 0.24622 0.25000 1.0000
N31 N 1 0.00378 0.99622 0.75000 1.0000
N32 N 1 0.49622 0.74622 0.25000 1.0000
N33 N 1 0.39607 0.13438 0.84934 1.0000
N34 N 1 0.86562 0.60393 0.65066 1.0000
N35 N 1 0.25459 0.63438 0.84934 1.0000
N36 N 1 0.75459 0.51628 0.84934 1.0000
N37 N 1 0.10393 0.98372 0.15066 1.0000
N38 N 1 0.13438 0.39607 0.34934 1.0000
N39 N 1 0.63438 0.25459 0.34934 1.0000
N40 N 1 0.98372 0.10393 0.65066 1.0000
N41 N 1 0.51628 0.75459 0.34934 1.0000
N42 N 1 0.74541 0.36562 0.15066 1.0000
N43 N 1 0.24541 0.48372 0.15066 1.0000
N44 N 1 0.89607 0.01628 0.84934 1.0000
N45 N 1 0.60393 0.86562 0.15066 1.0000
N46 N 1 0.36562 0.74541 0.65066 1.0000
N47 N 1 0.01628 0.89607 0.34934 1.0000
N48 N 1 0.48372 0.24541 0.65066 1.0000
N49 N 1 0.39175 0.99766 0.26588 1.0000
N50 N 1 0.00234 0.60825 0.23412 1.0000
N51 N 1 0.84237 0.49766 0.26588 1.0000
N52 N 1 0.34237 0.23647 0.26588 1.0000
N53 N 1 0.10825 0.26353 0.73412 1.0000
N54 N 1 0.99766 0.39175 0.76588 1.0000
N55 N 1 0.49766 0.84237 0.76588 1.0000
N56 N 1 0.26353 0.10825 0.23412 1.0000
N57 N 1 0.23647 0.34237 0.76588 1.0000
N58 N 1 0.15763 0.50234 0.73412 1.0000
N59 N 1 0.65763 0.76353 0.73412 1.0000
N60 N 1 0.89175 0.73647 0.26588 1.0000
N61 N 1 0.60825 0.00234 0.73412 1.0000
N62 N 1 0.50234 0.15763 0.23412 1.0000
N63 N 1 0.73647 0.89175 0.76588 1.0000
N64 N 1 0.76353 0.65763 0.23412 1.0000
N65 N 1 0.57172 0.09382 0.99438 1.0000
N66 N 1 0.90618 0.42828 0.50562 1.0000
N113 N 1 0.85445 0.17633 0.55424 1.0000
N114 N 1 0.92633 0.84131 0.05424 1.0000
N115 N 1 0.59131 0.26944 0.55424 1.0000
N116 N 1 0.01944 0.10445 0.05424 1.0000
N117 N 1 0.34449 0.77071 0.51284 1.0000
N118 N 1 0.52071 0.39267 0.01284 1.0000
N119 N 1 0.14267 0.71645 0.51284 1.0000
N120 N 1 0.46645 0.59449 0.01284 1.0000
N121 N 1 0.85538 0.21948 0.63835 1.0000
N122 N 1 0.96948 0.75627 0.13835 1.0000
N123 N 1 0.50627 0.14218 0.63835 1.0000
N124 N 1 0.89218 0.10538 0.13835 1.0000
N125 N 1 0.35732 0.65154 0.58037 1.0000
N126 N 1 0.40154 0.31231 0.08037 1.0000
N127 N 1 0.06231 0.76808 0.58037 1.0000
N128 N 1 0.51808 0.60732 0.08037 1.0000
N129 N 1 0.05991 0.26716 0.12160 1.0000
N130 N 1 0.01716 0.06849 0.62160 1.0000
N131 N 1 0.81849 0.61124 0.12160 1.0000
N132 N 1 0.36124 0.30991 0.62160 1.0000
N133 N 1 0.25764 0.75280 0.18863 1.0000
N134 N 1 0.50280 0.80372 0.68863 1.0000
N135 N 1 0.55372 0.05857 0.18863 1.0000
N136 N 1 0.80857 0.50764 0.68863 1.0000
N137 N 1 0.34342 0.86832 0.93115 1.0000
N138 N 1 0.61832 0.97544 0.43115 1.0000
N139 N 1 0.72544 0.20054 0.93115 1.0000
N140 N 1 0.95054 0.59342 0.43115 1.0000
N141 N 1 0.22924 0.38023 0.86186 1.0000
N142 N 1 0.13023 0.15890 0.36186 1.0000
N143 N 1 0.90890 0.75791 0.86186 1.0000
N144 N 1 0.50791 0.47924 0.36186 1.0000
N145 N 1 0.85119 0.01054 0.56385 1.0000
N146 N 1 0.76054 0.83496 0.06385 1.0000
N147 N 1 0.58496 0.42561 0.56385 1.0000
N148 N 1 0.17561 0.10119 0.06385 1.0000
N149 N 1 0.57721 0.27717 0.17974 1.0000
N150 N 1 0.02717 0.49305 0.67974 1.0000
N151 N 1 0.24305 0.54309 0.17974 1.0000
N152 N 1 0.29309 0.82721 0.67974 1.0000
N153 N 1 0.51542 0.14082 0.41269 1.0000
N154 N 1 0.89082 0.32189 0.91269 1.0000
N155 N 1 0.07189 0.44649 0.41269 1.0000
N156 N 1 0.19649 0.76542 0.91269 1.0000
N157 N 1 0.76525 0.84199 0.79245 1.0000
N158 N 1 0.59199 0.69230 0.29245 1.0000
N159 N 1 0.44230 0.36556 0.79245 1.0000
N160 N 1 0.11556 0.01525 0.29245 1.0000
Cd1 Cd 1 0.90276 0.18004 0.38717 1.0000
Cd2 Cd 1 0.93004 0.96006 0.88717 1.0000
Cd3 Cd 1 0.71006 0.43278 0.38717 1.0000
Cd4 Cd 1 0.18278 0.15276 0.88717 1.0000
Cd5 Cd 1 0.40840 0.62304 0.92697 1.0000
Cd6 Cd 1 0.37304 0.91463 0.42697 1.0000
Cd7 Cd 1 0.66463 0.44999 0.92697 1.0000
Cd8 Cd 1 0.19999 0.65839 0.42697 1.0000
Cd9 Cd 1 0.48460 0.04234 0.62548 1.0000
Cd10 Cd 1 0.79234 0.13992 0.12548 1.0000
Cd11 Cd 1 0.88992 0.33218 0.62548 1.0000
Cd12 Cd 1 0.08218 0.73460 0.12548 1.0000
Cd13 Cd 1 0.40807 0.54832 0.59617 1.0000
Cd14 Cd 1 0.29832 0.24577 0.09617 1.0000
Cd15 Cd 1 0.99577 0.85551 0.59617 1.0000
Cd16 Cd 1 0.60551 0.65807 0.09617 1.0000
#End

data_12-yqt1_Cd
_audit_creation_method ToposPro
_Chemical_Name_Systematic GUPBOJ
_cell_length_a 10.9153
_cell_length_b 10.9153
_cell_length_c 12.76498
_cell_angle_alpha 105.5104
_cell_angle_beta 105.5104
_cell_angle_gamma 98.0433
_cell_volume 1375.011
_cell_formula_units_Z 6
_symmetry_space_group_name_H-M 'P 1'
_symmetry_Int_Tables_number 1
loop_
_symmetry_equiv_pos_site_id
_symmetry_equiv_pos_as_xyz
1 x,y,z
loop_
_atom_site_label
_atom_site_type_symbol
_atom_site_symmetry_multiplicity
_atom_site_fract_x
_atom_site_fract_y
_atom_site_fract_z
_atom_site_occupancy
N1 N 1 0.08764 0.11734 0.38370 1.0000
N2 N 1 0.88266 0.91236 0.11630 1.0000
N3 N 1 0.91115 0.88109 0.61577 1.0000
N4 N 1 0.11891 0.08885 0.88423 1.0000
N5 N 1 0.11784 0.31748 0.45700 1.0000
N6 N 1 0.68252 0.88216 0.04300 1.0000
N7 N 1 0.86891 0.68024 0.53995 1.0000
N8 N 1 0.31976 0.13109 0.96005 1.0000
N9 N 1 0.10767 0.60595 0.36373 1.0000
N10 N 1 0.39405 0.89233 0.13627 1.0000

N67 N 1 0.93390 0.59382 0.99438 1.0000
N68 N 1 0.43390 0.41180 0.99438 1.0000
N69 N 1 0.92828 0.08820 0.00562 1.0000
N70 N 1 0.09382 0.57172 0.49438 1.0000
N71 N 1 0.59382 0.93390 0.49438 1.0000
N72 N 1 0.08820 0.92828 0.50562 1.0000
N73 N 1 0.41180 0.43390 0.49438 1.0000
N74 N 1 0.06610 0.40618 0.00562 1.0000
N75 N 1 0.56610 0.58820 0.00562 1.0000
N76 N 1 0.07172 0.91180 0.99438 1.0000
N77 N 1 0.42828 0.90618 0.00562 1.0000
N78 N 1 0.40618 0.06610 0.50562 1.0000
N79 N 1 0.91180 0.07172 0.49438 1.0000
N80 N 1 0.58820 0.56610 0.50562 1.0000
N81 N 1 0.18169 0.83804 0.39327 1.0000
N82 N 1 0.16196 0.81831 0.10673 1.0000
N83 N 1 0.92505 0.33804 0.39327 1.0000
N84 N 1 0.42505 0.26869 0.39327 1.0000
N85 N 1 0.31831 0.23131 0.60673 1.0000
N86 N 1 0.83804 0.18169 0.89327 1.0000
N87 N 1 0.33804 0.92505 0.89327 1.0000
N88 N 1 0.23131 0.31831 0.10673 1.0000
N89 N 1 0.26869 0.42505 0.89327 1.0000
N90 N 1 0.07495 0.66196 0.60673 1.0000
N91 N 1 0.57495 0.73131 0.60673 1.0000
N92 N 1 0.68169 0.76869 0.39327 1.0000
N93 N 1 0.81831 0.16196 0.60673 1.0000
N94 N 1 0.66196 0.07495 0.10673 1.0000
N95 N 1 0.76869 0.68169 0.89327 1.0000
N96 N 1 0.73131 0.57495 0.10673 1.0000
N97 N 1 0.41486 0.03111 0.14435 1.0000
N98 N 1 0.96889 0.58514 0.35565 1.0000
N99 N 1 0.94078 0.53111 0.14435 1.0000
N100 N 1 0.44078 0.32453 0.14435 1.0000
N101 N 1 0.08514 0.17547 0.85565 1.0000
N102 N 1 0.03111 0.41486 0.64435 1.0000
N103 N 1 0.53111 0.94078 0.64435 1.0000
N104 N 1 0.17547 0.08514 0.35565 1.0000
N105 N 1 0.32453 0.44078 0.64435 1.0000
N106 N 1 0.05922 0.46889 0.85565 1.0000
N107 N 1 0.55922 0.67547 0.85565 1.0000
N108 N 1 0.91486 0.82453 0.14435 1.0000
N109 N 1 0.58514 0.96889 0.85565 1.0000
N110 N 1 0.46889 0.05922 0.35565 1.0000
N111 N 1 0.82453 0.91486 0.64435 1.0000
N112 N 1 0.67547 0.55922 0.35565 1.0000
N113 N 1 0.40898 0.14003 0.90954 1.0000
N114 N 1 0.85997 0.59102 0.59046 1.0000
N115 N 1 0.18149 0.64003 0.90954 1.0000
N116 N 1 0.68149 0.45043 0.90954 1.0000
N117 N 1 0.09102 0.04957 0.09046 1.0000
N118 N 1 0.14003 0.40898 0.40954 1.0000
N119 N 1 0.64003 0.18149 0.40954 1.0000
N120 N 1 0.04957 0.09102 0.59046 1.0000
N121 N 1 0.45043 0.68149 0.40954 1.0000
N122 N 1 0.81851 0.35997 0.09046 1.0000
N123 N 1 0.31851 0.54957 0.09046 1.0000
N124 N 1 0.90898 0.95043 0.90954 1.0000
N125 N 1 0.59102 0.85997 0.09046 1.0000
N126 N 1 0.35997 0.81851 0.59046 1.0000
N127 N 1 0.95043 0.90898 0.40954 1.0000
N128 N 1 0.54957 0.31851 0.59046 1.0000
N129 N 1 0.48886 0.03658 0.17275 1.0000
N130 N 1 0.96342 0.51114 0.32725 1.0000
N131 N 1 0.83839 0.53658 0.17275 1.0000
N132 N 1 0.33839 0.29067 0.17275 1.0000
N133 N 1 0.01114 0.20933 0.82725 1.0000
N134 N 1 0.03658 0.48886 0.67275 1.0000
N135 N 1 0.53658 0.83839 0.67275 1.0000
N136 N 1 0.20933 0.01114 0.32725 1.0000
N137 N 1 0.29067 0.33839 0.67275 1.0000
N138 N 1 0.16161 0.46342 0.82725 1.0000
N139 N 1 0.66161 0.70933 0.82725 1.0000
N140 N 1 0.98886 0.79067 0.17275 1.0000
N141 N 1 0.51114 0.96342 0.82725 1.0000
N142 N 1 0.46342 0.16161 0.32725 1.0000
N143 N 1 0.79067 0.98886 0.67275 1.0000
N144 N 1 0.70933 0.66161 0.32725 1.0000
N145 N 1 0.10576 0.80473 0.43366 1.0000
N146 N 1 0.19527 0.89424 0.06634 1.0000
N147 N 1 0.96059 0.30473 0.43366 1.0000
N148 N 1 0.46059 0.26161 0.43366 1.0000
N149 N 1 0.39424 0.23839 0.56634 1.0000
N150 N 1 0.80473 0.10576 0.93366 1.0000
N151 N 1 0.30473 0.96059 0.93366 1.0000
N152 N 1 0.23839 0.39424 0.06634 1.0000
N153 N 1 0.26161 0.46059 0.93366 1.0000
N154 N 1 0.03941 0.69527 0.56634 1.0000
N155 N 1 0.53941 0.73839 0.56634 1.0000
N156 N 1 0.60576 0.76161 0.43366 1.0000
N157 N 1 0.89424 0.19527 0.56634 1.0000
N158 N 1 0.69527 0.03941 0.06634 1.0000
N159 N 1 0.76161 0.60576 0.93366 1.0000
N160 N 1 0.73839 0.53941 0.06634 1.0000
Zn1 Zn 1 0.30144 0.99955 0.19912 1.0000
Zn2 Zn 1 0.00045 0.69856 0.30088 1.0000
Zn3 Zn 1 0.99944 0.49955 0.19912 1.0000
Zn4 Zn 1 0.49944 0.30133 0.19912 1.0000
Zn5 Zn 1 0.19856 0.19867 0.80088 1.0000
Zn6 Zn 1 0.99955 0.30144 0.69912 1.0000
Zn7 Zn 1 0.49955 0.99944 0.69912 1.0000
N11 N 1 0.90631 0.39449 0.63765 1.0000
N12 N 1 0.60551 0.09369 0.86235 1.0000
N13 N 1 0.05375 0.74456 0.28781 1.0000
N14 N 1 0.25544 0.94625 0.21219 1.0000
N15 N 1 0.95383 0.25583 0.71552 1.0000
N16 N 1 0.74417 0.04617 0.78448 1.0000
N17 N 1 0.24196 0.66389 0.66302 1.0000
N18 N 1 0.33611 0.75804 0.83698 1.0000
N19 N 1 0.75902 0.33656 0.33678 1.0000
N20 N 1 0.66344 0.24098 0.16322 1.0000
N21 N 1 0.40386 0.47041 0.51147 1.0000
N22 N 1 0.52959 0.59614 0.98853 1.0000
N23 N 1 0.59332 0.45219 0.52367 1.0000
N24 N 1 0.54781 0.40668 0.97633 1.0000
N25 N 1 0.11803 0.22822 0.36595 1.0000
N26 N 1 0.77178 0.88197 0.13405 1.0000
N27 N 1 0.86783 0.77096 0.62973 1.0000
N28 N 1 0.22904 0.13217 0.87027 1.0000
N29 N 1 0.06906 0.13773 0.48507 1.0000
N30 N 1 0.86227 0.93094 0.01493 1.0000
N31 N 1 0.93895 0.85888 0.51815 1.0000
N32 N 1 0.14112 0.06105 0.98185 1.0000
N33 N 1 0.08850 0.26240 0.53069 1.0000
N34 N 1 0.73760 0.91150 0.96931 1.0000
N35 N 1 0.91255 0.73386 0.47100 1.0000
N36 N 1 0.26614 0.08745 0.02900 1.0000
N37 N 1 0.14461 0.72693 0.36868 1.0000
N38 N 1 0.27307 0.85539 0.13132 1.0000
N39 N 1 0.86707 0.27378 0.63199 1.0000
N40 N 1 0.72622 0.13293 0.86801 1.0000
N41 N 1 0.96116 0.63545 0.23307 1.0000
N42 N 1 0.36455 0.03884 0.26693 1.0000
N43 N 1 0.04623 0.36461 0.77282 1.0000
N44 N 1 0.63539 0.95377 0.72718 1.0000
N45 N 1 0.99448 0.54911 0.28037 1.0000
N46 N 1 0.45089 0.00552 0.21963 1.0000
N47 N 1 0.01676 0.45103 0.72420 1.0000
N48 N 1 0.54897 0.98324 0.77580 1.0000
N49 N 1 0.33690 0.66310 0.75000 1.0000
N50 N 1 0.66330 0.33670 0.25000 1.0000
N51 N 1 0.18262 0.75874 0.69577 1.0000
N52 N 1 0.24126 0.81738 0.80423 1.0000
N53 N 1 0.81787 0.24139 0.30410 1.0000
N54 N 1 0.75861 0.18213 0.19590 1.0000
N55 N 1 0.50901 0.52062 0.49375 1.0000
N56 N 1 0.47938 0.49099 0.00625 1.0000
N57 N 1 0.42281 0.37156 0.55193 1.0000
N58 N 1 0.62844 0.57719 0.94807 1.0000
N59 N 1 0.54064 0.36029 0.55947 1.0000
N60 N 1 0.63971 0.45936 0.94053 1.0000
Cd1 Cd 1 0.21099 0.52395 0.49126 1.0000
Cd2 Cd 1 0.47605 0.78901 0.00874 1.0000
Cd3 Cd 1 0.79333 0.47242 0.51102 1.0000
Cd4 Cd 1 0.52758 0.20667 0.98898 1.0000
Cd5 Cd 1 0.06725 0.93275 0.25000 1.0000
Cd6 Cd 1 0.93322 0.06678 0.75000 1.0000
#End

data_13-ict_Cd
_audit_creation_method ToposPro
_Chemical_Name_Systematic GUPBOJ01
_cell_length_a 17.22157
_cell_length_b 27.25072
_cell_length_c 14.22583
_cell_angle_alpha 90
_cell_angle_beta 102.6176
_cell_angle_gamma 90
_cell_volume 6514.951
_cell_formula_units_Z 24
_symmetry_space_group_name_H-M 'P 21/c'
_symmetry_Int_Tables_number 14
loop_
_symmetry_equiv_pos_site_id
_symmetry_equiv_pos_as_xyz
1 x,y,z
2 -x,1/2+y,1/2-z
3 -x,-y,-z
4 x,1/2-y,1/2+z
loop_
_atom_site_label
_atom_site_type_symbol
_atom_site_symmetry_multiplicity
_atom_site_fract_x
_atom_site_fract_y
_atom_site_fract_z
_atom_site_occupancy
N1 N 4 0.64963 0.10915 0.19321 1.0000
N2 N 4 0.54352 0.14807 0.16491 1.0000
N3 N 4 0.41639 0.13959 0.32007 1.0000
N4 N 4 0.44078 0.11408 0.46076 1.0000
N5 N 4 0.45416 0.25858 0.21626 1.0000
N6 N 4 0.47172 0.33238 0.19133 1.0000
N7 N 4 0.42191 0.46677 0.12619 1.0000
N8 N 4 0.33776 0.51347 0.16181 1.0000
N9 N 4 0.76386 0.01166 0.12994 1.0000
N10 N 4 0.78074 0.99286 0.99398 1.0000
N11 N 4 0.72388 0.44595 0.25307 1.0000
N12 N 4 0.62035 0.41568 0.17152 1.0000
N13 N 4 0.82500 0.93566 0.78298 1.0000
N14 N 4 0.85958 0.88716 0.68755 1.0000
N15 N 4 0.04388 0.28460 0.87997 1.0000

Zn8 Zn 1 0.19867 0.19856 0.30088 1.0000
Zn9 Zn 1 0.30133 0.49944 0.69912 1.0000
Zn10 Zn 1 0.00056 0.50045 0.80088 1.0000
Zn11 Zn 1 0.50056 0.69867 0.80088 1.0000
Zn12 Zn 1 0.80144 0.80133 0.19912 1.0000
Zn13 Zn 1 0.69856 0.00045 0.80088 1.0000
Zn14 Zn 1 0.50045 0.00056 0.30088 1.0000
Zn15 Zn 1 0.80133 0.80144 0.69912 1.0000
Zn16 Zn 1 0.69867 0.50056 0.30088 1.0000
#End

data_12-mog_Zn
_audit_creation_method ToposPro
_Chemical_Name_Systematic CUIMDZ03
_cell_length_a 12.1276
_cell_length_b 12.1276
_cell_length_c 9.507081
_cell_angle_alpha 94.94559
_cell_angle_beta 94.94559
_cell_angle_gamma 124.2176
_cell_volume 1136.451
_cell_formula_units_Z 6
_symmetry_space_group_name_H-M 'P 1'
_symmetry_Int_Tables_number 1
loop_
_symmetry_equiv_pos_site_id
_symmetry_equiv_pos_as_xyz
1 x,y,z
loop_
_atom_site_label
_atom_site_type_symbol
_atom_site_symmetry_multiplicity
_atom_site_fract_x
_atom_site_fract_y
_atom_site_fract_z
_atom_site_occupancy
N1 N 1 0.10932 0.61480 0.24285 1.0000
N2 N 1 0.38520 0.89068 0.25715 1.0000
N3 N 1 0.89068 0.38520 0.75715 1.0000
N4 N 1 0.61480 0.10932 0.74285 1.0000
N5 N 1 0.93934 0.60390 0.28627 1.0000
N6 N 1 0.39610 0.06066 0.21373 1.0000
N7 N 1 0.06066 0.39610 0.71373 1.0000
N8 N 1 0.60390 0.93934 0.78627 1.0000
N9 N 1 0.07045 0.68188 0.31189 1.0000
N10 N 1 0.31812 0.92955 0.18811 1.0000
N11 N 1 0.92955 0.31812 0.68811 1.0000
N12 N 1 0.68188 0.07045 0.81189 1.0000
N13 N 1 0.00265 0.49586 0.17496 1.0000
N14 N 1 0.50414 0.99735 0.32504 1.0000
N15 N 1 0.99735 0.50414 0.82504 1.0000
N16 N 1 0.49586 0.00265 0.67496 1.0000
N17 N 1 0.89674 0.48889 0.20168 1.0000
N18 N 1 0.51111 0.10326 0.29832 1.0000
N19 N 1 0.10326 0.51111 0.79832 1.0000
N20 N 1 0.48889 0.89674 0.70168 1.0000
N21 N 1 0.81087 0.76724 0.49356 1.0000
N22 N 1 0.23276 0.18913 0.00644 1.0000
N23 N 1 0.18913 0.23276 0.50644 1.0000
N24 N 1 0.76724 0.81087 0.99356 1.0000
N25 N 1 0.74787 0.81795 0.66833 1.0000
N26 N 1 0.18205 0.25213 0.83167 1.0000
N27 N 1 0.25213 0.18205 0.33167 1.0000
N28 N 1 0.81795 0.74787 0.16833 1.0000
N29 N 1 0.84060 0.89038 0.48837 1.0000
N30 N 1 0.10962 0.15940 0.01163 1.0000
N31 N 1 0.15940 0.10962 0.51163 1.0000
N32 N 1 0.89038 0.84060 0.98837 1.0000
N33 N 1 0.75343 0.72230 0.60507 1.0000
N34 N 1 0.27770 0.24657 0.89493 1.0000
N35 N 1 0.24657 0.27770 0.39493 1.0000
N36 N 1 0.72230 0.75343 0.10507 1.0000
N37 N 1 0.80174 0.92216 0.59704 1.0000
N38 N 1 0.07784 0.19826 0.90296 1.0000
N39 N 1 0.19826 0.07784 0.40296 1.0000
N40 N 1 0.92216 0.80174 0.09704 1.0000
N41 N 1 0.35293 0.63509 0.42035 1.0000
N42 N 1 0.36491 0.64707 0.07965 1.0000
N43 N 1 0.64707 0.36491 0.57965 1.0000
N44 N 1 0.63509 0.35293 0.92035 1.0000
N45 N 1 0.31652 0.52465 0.58690 1.0000
N46 N 1 0.47535 0.68348 0.91310 1.0000
N47 N 1 0.68348 0.47535 0.41310 1.0000
N48 N 1 0.52465 0.31652 0.08690 1.0000
N49 N 1 0.25657 0.54472 0.48248 1.0000
N50 N 1 0.45528 0.74343 0.01752 1.0000
N51 N 1 0.74343 0.45528 0.51752 1.0000
N52 N 1 0.54472 0.25657 0.98248 1.0000
N53 N 1 0.47183 0.67049 0.48567 1.0000
N54 N 1 0.32951 0.52817 0.01433 1.0000
N55 N 1 0.52817 0.32951 0.51433 1.0000
N56 N 1 0.67049 0.47183 0.98567 1.0000
N57 N 1 0.44902 0.60170 0.58937 1.0000
N58 N 1 0.39830 0.55098 0.91063 1.0000
N59 N 1 0.55098 0.39830 0.41063 1.0000
N60 N 1 0.60170 0.44902 0.08937 1.0000
Zn1 Zn 1 0.30541 0.69459 0.25000 1.0000
Zn2 Zn 1 0.69459 0.30541 0.75000 1.0000
Zn3 Zn 1 0.80728 0.64739 0.32961 1.0000
Zn4 Zn 1 0.35261 0.19272 0.17039 1.0000
Zn5 Zn 1 0.19272 0.35261 0.67039 1.0000
N16 N 4 0.05426 0.21036 0.85252 1.0000
N17 N 4 0.96791 0.09389 0.40382 1.0000
N18 N 4 0.85499 0.06633 0.34789 1.0000
N19 N 4 0.92125 0.04812 0.89921 1.0000
N20 N 4 0.02270 0.09023 0.90591 1.0000
N21 N 4 0.21054 0.16394 0.98782 1.0000
N22 N 4 0.31371 0.17794 0.09015 1.0000
N23 N 4 0.14942 0.12752 0.72455 1.0000
N24 N 4 0.13663 0.12972 0.57603 1.0000
N25 N 4 0.57863 0.11009 0.21170 1.0000
N26 N 4 0.65841 0.14620 0.13556 1.0000
N27 N 4 0.59244 0.17050 0.11808 1.0000
N28 N 4 0.47052 0.13997 0.40013 1.0000
N29 N 4 0.36868 0.09778 0.41874 1.0000
N30 N 4 0.35353 0.11361 0.33111 1.0000
N31 N 4 0.45531 0.29025 0.14780 1.0000
N32 N 4 0.48072 0.32682 0.28601 1.0000
N33 N 4 0.46967 0.28087 0.30154 1.0000
N34 N 4 0.37733 0.47318 0.18833 1.0000
N35 N 4 0.41017 0.50291 0.06163 1.0000
N36 N 4 0.35789 0.53202 0.08398 1.0000
N37 N 4 0.75150 0.02686 0.04087 1.0000
N38 N 4 0.80078 0.96872 0.13835 1.0000
N39 N 4 0.81111 0.95698 0.05397 1.0000
N40 N 4 0.64703 0.45246 0.22818 1.0000
N41 N 4 0.68012 0.38660 0.16157 1.0000
N42 N 4 0.74461 0.40544 0.21220 1.0000
N43 N 4 0.85103 0.93374 0.70356 1.0000
N44 N 4 0.83921 0.86047 0.75670 1.0000
N45 N 4 0.81758 0.89068 0.81598 1.0000
N46 N 4 0.09393 0.25143 0.86349 1.0000
N47 N 4 0.98010 0.21799 0.86238 1.0000
N48 N 4 0.97349 0.26422 0.87936 1.0000
N49 N 4 0.90786 0.09729 0.33018 1.0000
N50 N 4 0.95245 0.06101 0.46643 1.0000
N51 N 4 0.88222 0.04379 0.43171 1.0000
N52 N 4 0.96195 0.07140 0.84578 1.0000
N53 N 4 0.95658 0.05259 0.99175 1.0000
N54 N 4 0.01981 0.07875 0.99600 1.0000
N55 N 4 0.28369 0.14919 0.01798 1.0000
N56 N 4 0.19527 0.20137 0.04104 1.0000
N57 N 4 0.25937 0.21013 0.10471 1.0000
N58 N 4 0.09854 0.13747 0.64450 1.0000
N59 N 4 0.21075 0.11512 0.61350 1.0000
N60 N 4 0.21868 0.11379 0.70590 1.0000
Cd1 Cd 4 0.43086 0.17901 0.18858 1.0000
Cd2 Cd 4 0.49312 0.40037 0.11443 1.0000
Cd3 Cd 4 0.73756 0.05238 0.25514 1.0000
Cd4 Cd 4 0.80928 0.00630 0.84988 1.0000
Cd5 Cd 4 0.07800 0.36035 0.92281 1.0000
Cd6 Cd 4 0.11657 0.13656 0.86599 1.0000
#End

data_14-zni2_Cd
_audit_creation_method ToposPro
_Chemical_Name_Systematic GITTAF
_cell_length_a 24.3736
_cell_length_b 24.3736
_cell_length_c 18.48373
_cell_angle_alpha 48.75152
_cell_angle_beta 48.75152
_cell_angle_gamma 90
_cell_volume 3967.94
_cell_formula_units_Z 16
_symmetry_space_group_name_H-M 'P 1'
_symmetry_Int_Tables_number 1
loop_
_symmetry_equiv_pos_site_id
_symmetry_equiv_pos_as_xyz
1 x,y,z
loop_
_atom_site_label
_atom_site_type_symbol
_atom_site_symmetry_multiplicity
_atom_site_fract_x
_atom_site_fract_y
_atom_site_fract_z
_atom_site_occupancy
N1 N 1 0.15955 0.75000 0.50000 1.0000
N2 N 1 0.25000 0.84045 0.00000 1.0000
N3 N 1 0.84045 0.25000 0.50000 1.0000
N4 N 1 0.34045 0.25000 0.50000 1.0000
N5 N 1 0.75000 0.15955 0.00000 1.0000
N6 N 1 0.25000 0.34045 0.00000 1.0000
N7 N 1 0.65955 0.75000 0.50000 1.0000
N8 N 1 0.75000 0.65955 0.00000 1.0000
N9 N 1 0.52301 0.08787 0.98472 1.0000
N10 N 1 0.91213 0.47699 0.51528 1.0000
N11 N 1 0.99226 0.58787 0.98472 1.0000
N12 N 1 0.49226 0.42740 0.98472 1.0000
N13 N 1 0.97699 0.07260 0.01528 1.0000
N14 N 1 0.08787 0.52301 0.48472 1.0000
N15 N 1 0.58787 0.99226 0.48472 1.0000
N16 N 1 0.07260 0.97699 0.51528 1.0000
N17 N 1 0.42740 0.49226 0.48472 1.0000
N18 N 1 0.00774 0.41213 0.01528 1.0000
N19 N 1 0.50774 0.57260 0.01528 1.0000
N20 N 1 0.02301 0.92740 0.98472 1.0000
N21 N 1 0.47699 0.91213 0.01528 1.0000
N22 N 1 0.41213 0.00774 0.51528 1.0000
N23 N 1 0.92740 0.02301 0.48472 1.0000

Zn6 Zn 1 0.64739 0.80728 0.82961 1.0000
#End

data_13-ict_Zn
_audit_creation_method ToposPro
_Chemical_Name_Systematic GUPBOJ01
_cell_length_a 15.99498
_cell_length_b 26.65099
_cell_length_c 13.36449
_cell_angle_alpha 90
_cell_angle_beta 104.7179
_cell_angle_gamma 90
_cell_volume 5510.115
_cell_formula_units_Z 24
_symmetry_space_group_name_H-M 'P 21/c'
_symmetry_Int_Tables_number 14
loop_
_symmetry_equiv_pos_site_id
_symmetry_equiv_pos_as_xyz
1 x,y,z
2 -x,1/2+y,1/2-z
3 -x,-y,-z
4 x,1/2-y,1/2+z
loop_
_atom_site_label
_atom_site_type_symbol
_atom_site_symmetry_multiplicity
_atom_site_fract_x
_atom_site_fract_y
_atom_site_fract_z
_atom_site_occupancy
N1 N 4 0.64476 0.10728 0.18337 1.0000
N2 N 4 0.53801 0.15367 0.16646 1.0000
N3 N 4 0.41481 0.14881 0.31832 1.0000
N4 N 4 0.43618 0.12071 0.46716 1.0000
N5 N 4 0.45279 0.25614 0.21515 1.0000
N6 N 4 0.46920 0.33185 0.19004 1.0000
N7 N 4 0.41592 0.45599 0.13613 1.0000
N8 N 4 0.34240 0.50843 0.18971 1.0000
N9 N 4 0.76380 0.01577 0.11746 1.0000
N10 N 4 0.79387 0.00282 0.97766 1.0000
N11 N 4 0.72470 0.44171 0.24874 1.0000
N12 N 4 0.60809 0.41074 0.16858 1.0000
N13 N 4 0.83609 0.94974 0.78201 1.0000
N14 N 4 0.87621 0.89681 0.69081 1.0000
N15 N 4 0.03637 0.29759 0.86863 1.0000
N16 N 4 0.04939 0.22244 0.83628 1.0000
N17 N 4 0.95611 0.09412 0.39687 1.0000
N18 N 4 0.83321 0.06628 0.33300 1.0000
N19 N 4 0.91966 0.06017 0.88188 1.0000
N20 N 4 0.01927 0.11012 0.88503 1.0000
N21 N 4 0.20138 0.16877 0.97561 1.0000
N22 N 4 0.31386 0.17903 0.09135 1.0000
N23 N 4 0.13896 0.13552 0.71924 1.0000
N24 N 4 0.12648 0.12762 0.56166 1.0000
N25 N 4 0.56587 0.10851 0.19400 1.0000
N26 N 4 0.66560 0.15144 0.14965 1.0000
N27 N 4 0.59932 0.18030 0.13923 1.0000
N28 N 4 0.47438 0.14341 0.40466 1.0000
N29 N 4 0.35347 0.11213 0.41997 1.0000
N30 N 4 0.34028 0.12953 0.32747 1.0000
N31 N 4 0.43996 0.29016 0.14275 1.0000
N32 N 4 0.50008 0.32363 0.29103 1.0000
N33 N 4 0.48976 0.27656 0.30667 1.0000
N34 N 4 0.37972 0.46496 0.21125 1.0000
N35 N 4 0.40131 0.49376 0.06846 1.0000
N36 N 4 0.35566 0.52637 0.10185 1.0000
N37 N 4 0.74209 0.02812 0.01983 1.0000
N38 N 4 0.82910 0.98319 0.13576 1.0000
N39 N 4 0.84772 0.97520 0.04892 1.0000
N40 N 4 0.64212 0.45112 0.21754 1.0000
N41 N 4 0.66920 0.37658 0.16969 1.0000
N42 N 4 0.74164 0.39577 0.21939 1.0000
N43 N 4 0.86209 0.94465 0.69775 1.0000
N44 N 4 0.85941 0.87241 0.77054 1.0000
N45 N 4 0.83429 0.90521 0.82710 1.0000
N46 N 4 0.09242 0.26431 0.85596 1.0000
N47 N 4 0.96708 0.22972 0.83692 1.0000
N48 N 4 0.95886 0.27646 0.85688 1.0000
N49 N 4 0.89352 0.09433 0.31259 1.0000
N50 N 4 0.93488 0.06595 0.46900 1.0000
N51 N 4 0.85847 0.04857 0.42933 1.0000
N52 N 4 0.96760 0.07851 0.82526 1.0000
N53 N 4 0.94152 0.08049 0.97629 1.0000
N54 N 4 0.00338 0.11158 0.97800 1.0000
N55 N 4 0.28200 0.15471 0.00536 1.0000
N56 N 4 0.18356 0.20145 0.04292 1.0000
N57 N 4 0.25320 0.20783 0.11460 1.0000
N58 N 4 0.08915 0.14690 0.62878 1.0000
N59 N 4 0.19927 0.10433 0.61032 1.0000
N60 N 4 0.20702 0.10927 0.70826 1.0000
Zn1 Zn 4 0.42975 0.18269 0.18996 1.0000
Zn2 Zn 4 0.48193 0.39569 0.11630 1.0000
Zn3 Zn 4 0.72287 0.04859 0.22962 1.0000
Zn4 Zn 4 0.81598 0.01591 0.83834 1.0000
Zn5 Zn 4 0.06643 0.36805 0.91158 1.0000
Zn6 Zn 4 0.10632 0.15477 0.84785 1.0000
#End

data_14-nog_Zn
_audit_creation_method ToposPro

N24 N 1 0.57260 0.50774 0.51528 1.0000
N25 N 1 0.50165 0.25165 0.75000 1.0000
N26 N 1 0.74835 0.49835 0.75000 1.0000
N27 N 1 0.24835 0.75165 0.75000 1.0000
N28 N 1 0.99835 0.00165 0.25000 1.0000
N29 N 1 0.25165 0.50165 0.25000 1.0000
N30 N 1 0.75165 0.24835 0.25000 1.0000
N31 N 1 0.00165 0.99835 0.75000 1.0000
N32 N 1 0.49835 0.74835 0.25000 1.0000
N33 N 1 0.40096 0.13997 0.84240 1.0000
N34 N 1 0.86003 0.59904 0.65760 1.0000
N35 N 1 0.25664 0.63997 0.84240 1.0000
N36 N 1 0.75664 0.51763 0.84240 1.0000
N37 N 1 0.09904 0.98237 0.15760 1.0000
N38 N 1 0.13997 0.40096 0.34240 1.0000
N39 N 1 0.63997 0.25664 0.34240 1.0000
N40 N 1 0.98237 0.09904 0.65760 1.0000
N41 N 1 0.51763 0.75664 0.34240 1.0000
N42 N 1 0.74336 0.36003 0.15760 1.0000
N43 N 1 0.24336 0.48237 0.15760 1.0000
N44 N 1 0.90096 0.01763 0.84240 1.0000
N45 N 1 0.59904 0.86003 0.15760 1.0000
N46 N 1 0.36003 0.74336 0.65760 1.0000
N47 N 1 0.01763 0.90096 0.34240 1.0000
N48 N 1 0.48237 0.24336 0.65760 1.0000
N49 N 1 0.39871 0.00224 0.25307 1.0000
N50 N 1 0.99776 0.60129 0.24693 1.0000
N51 N 1 0.84822 0.50224 0.25307 1.0000
N52 N 1 0.34822 0.24468 0.25307 1.0000
N53 N 1 0.10129 0.25532 0.74693 1.0000
N54 N 1 0.00224 0.39871 0.75307 1.0000
N55 N 1 0.50224 0.84822 0.75307 1.0000
N56 N 1 0.25532 0.10129 0.24693 1.0000
N57 N 1 0.24468 0.34822 0.75307 1.0000
N58 N 1 0.15178 0.49776 0.74693 1.0000
N59 N 1 0.65178 0.75532 0.74693 1.0000
N60 N 1 0.89871 0.74468 0.25307 1.0000
N61 N 1 0.60129 0.99776 0.74693 1.0000
N62 N 1 0.49776 0.15178 0.24693 1.0000
N63 N 1 0.74468 0.89871 0.75307 1.0000
N64 N 1 0.75532 0.65178 0.24693 1.0000
N65 N 1 0.56614 0.09084 0.00167 1.0000
N66 N 1 0.90916 0.43386 0.49833 1.0000
N67 N 1 0.93218 0.59084 0.00167 1.0000
N68 N 1 0.43218 0.40749 0.00167 1.0000
N69 N 1 0.93386 0.09251 0.99833 1.0000
N70 N 1 0.09084 0.56614 0.50167 1.0000
N71 N 1 0.59084 0.93218 0.50167 1.0000
N72 N 1 0.09251 0.93386 0.49833 1.0000
N73 N 1 0.40749 0.43218 0.50167 1.0000
N74 N 1 0.06782 0.40916 0.99833 1.0000
N75 N 1 0.56782 0.59251 0.99833 1.0000
N76 N 1 0.06614 0.90749 0.00167 1.0000
N77 N 1 0.43386 0.90916 0.99833 1.0000
N78 N 1 0.40916 0.06782 0.49833 1.0000
N79 N 1 0.90749 0.06614 0.50167 1.0000
N80 N 1 0.59251 0.56782 0.49833 1.0000
N81 N 1 0.17642 0.83213 0.40100 1.0000
N82 N 1 0.16787 0.82358 0.09900 1.0000
N83 N 1 0.92258 0.33213 0.40100 1.0000
N84 N 1 0.42258 0.26686 0.40100 1.0000
N85 N 1 0.32358 0.23314 0.59900 1.0000
N86 N 1 0.83213 0.17642 0.90100 1.0000
N87 N 1 0.33213 0.92258 0.90100 1.0000
N88 N 1 0.23314 0.32358 0.09900 1.0000
N89 N 1 0.26686 0.42258 0.90100 1.0000
N90 N 1 0.07742 0.66787 0.59900 1.0000
N91 N 1 0.57742 0.73314 0.59900 1.0000
N92 N 1 0.67642 0.76686 0.40100 1.0000
N93 N 1 0.82358 0.16787 0.59900 1.0000
N94 N 1 0.66787 0.07742 0.09900 1.0000
N95 N 1 0.76686 0.67642 0.90100 1.0000
N96 N 1 0.73314 0.57742 0.09900 1.0000
N97 N 1 0.42002 0.03325 0.13981 1.0000
N98 N 1 0.96675 0.57998 0.36019 1.0000
N99 N 1 0.94017 0.53325 0.13981 1.0000
N100 N 1 0.44017 0.32694 0.13981 1.0000
N101 N 1 0.07998 0.17306 0.86019 1.0000
N102 N 1 0.03325 0.42002 0.63981 1.0000
N103 N 1 0.53325 0.94017 0.63981 1.0000
N104 N 1 0.17306 0.07998 0.36019 1.0000
N105 N 1 0.32694 0.44017 0.63981 1.0000
N106 N 1 0.05983 0.46675 0.86019 1.0000
N107 N 1 0.55983 0.67306 0.86019 1.0000
N108 N 1 0.92002 0.82694 0.13981 1.0000
N109 N 1 0.57998 0.96675 0.86019 1.0000
N110 N 1 0.46675 0.05983 0.36019 1.0000
N111 N 1 0.82694 0.92002 0.63981 1.0000
N112 N 1 0.67306 0.55983 0.36019 1.0000
N113 N 1 0.41330 0.14561 0.89833 1.0000
N114 N 1 0.85439 0.58670 0.60167 1.0000
N115 N 1 0.18837 0.64561 0.89833 1.0000
N116 N 1 0.68837 0.45605 0.89833 1.0000
N117 N 1 0.08670 0.04395 0.10167 1.0000
N118 N 1 0.14561 0.41330 0.39833 1.0000
N119 N 1 0.64561 0.18837 0.39833 1.0000
N120 N 1 0.04395 0.08670 0.60167 1.0000
N121 N 1 0.45605 0.68837 0.39833 1.0000
N122 N 1 0.81163 0.35439 0.10167 1.0000
N123 N 1 0.31163 0.54395 0.10167 1.0000
N124 N 1 0.91330 0.95605 0.89833 1.0000

_Chemical_Name_Systematic HIFWAV
_cell_length_a 23.87734
_cell_length_b 9.46632
_cell_length_c 23.99521
_cell_angle_alpha 90
_cell_angle_beta 91.32262
_cell_angle_gamma 90
_cell_volume 5422.204
_cell_formula_units_Z 20
_symmetry_space_group_name_H-M 'P 21/n'
_symmetry_Int_Tables_number 14
loop_
_symmetry_equiv_pos_site_id
_symmetry_equiv_pos_as_xyz
1 x,y,z
2 1/2-x,1/2+y,1/2-z
3 -x,-y,-z
4 1/2+x,1/2-y,1/2+z
loop_
_atom_site_label
_atom_site_type_symbol
_atom_site_symmetry_multiplicity
_atom_site_fract_x
_atom_site_fract_y
_atom_site_fract_z
_atom_site_occupancy
N1 N 4 0.78299 0.84962 0.72123 1.0000
N2 N 4 0.77087 0.66954 0.76866 1.0000
N3 N 4 0.83906 0.13018 0.66423 1.0000
N4 N 4 0.91823 0.21423 0.67798 1.0000
N5 N 4 0.96087 0.39222 0.78065 1.0000
N6 N 4 0.95126 0.57055 0.82965 1.0000
N7 N 4 0.00771 0.07603 0.76158 1.0000
N8 N 4 0.00081 0.89477 0.80935 1.0000
N9 N 4 0.60236 0.36539 0.61599 1.0000
N10 N 4 0.64531 0.52444 0.57557 1.0000
N11 N 4 0.51639 0.75362 0.42973 1.0000
N12 N 4 0.58395 0.72660 0.48452 1.0000
N13 N 4 0.68754 0.84856 0.55427 1.0000
N14 N 4 0.72477 0.98071 0.61299 1.0000
N15 N 4 0.70845 0.58846 0.46250 1.0000
N16 N 4 0.75123 0.54389 0.39052 1.0000
N17 N 4 0.73973 0.69293 0.26821 1.0000
N18 N 4 0.71611 0.86946 0.22241 1.0000
N19 N 4 0.13783 0.37653 0.67273 1.0000
N20 N 4 0.05536 0.31817 0.68436 1.0000
N21 N 4 0.74722 0.78801 0.75343 1.0000
N22 N 4 0.82853 0.76964 0.71640 1.0000
N23 N 4 0.82101 0.65777 0.74596 1.0000
N24 N 4 0.88335 0.12029 0.69659 1.0000
N25 N 4 0.84625 0.22999 0.62583 1.0000
N26 N 4 0.89553 0.28232 0.63441 1.0000
N27 N 4 0.98133 0.51771 0.78999 1.0000
N28 N 4 0.91828 0.36735 0.81434 1.0000
N29 N 4 0.91236 0.47813 0.84480 1.0000
N30 N 4 0.97559 0.01341 0.79704 1.0000
N31 N 4 0.04839 0.88408 0.78172 1.0000
N32 N 4 0.05270 0.99679 0.75196 1.0000
N33 N 4 0.59854 0.45338 0.57436 1.0000
N34 N 4 0.67802 0.48076 0.61781 1.0000
N35 N 4 0.65127 0.38208 0.64304 1.0000
N36 N 4 0.56628 0.69771 0.43401 1.0000
N37 N 4 0.50324 0.81645 0.47736 1.0000
N38 N 4 0.54522 0.79974 0.51144 1.0000
N39 N 4 0.71519 0.84820 0.60163 1.0000
N40 N 4 0.67993 0.98063 0.53632 1.0000
N41 N 4 0.70314 0.06285 0.57289 1.0000
N42 N 4 0.71792 0.63631 0.41264 1.0000
N43 N 4 0.73576 0.46719 0.47120 1.0000
N44 N 4 0.76237 0.43961 0.42643 1.0000
N45 N 4 0.75748 0.81627 0.25202 1.0000
N46 N 4 0.67305 0.77965 0.22034 1.0000
N47 N 4 0.68773 0.66993 0.24879 1.0000
N48 N 4 0.10027 0.36323 0.71084 1.0000
N49 N 4 0.11644 0.33968 0.62295 1.0000
N50 N 4 0.06517 0.30325 0.63023 1.0000
Zn1 Zn 4 0.76778 0.03277 0.68162 1.0000
Zn2 Zn 4 0.98715 0.25648 0.72229 1.0000
Zn3 Zn 4 0.96601 0.75935 0.86215 1.0000
Zn4 Zn 4 0.65849 0.67389 0.51840 1.0000
Zn5 Zn 4 0.78374 0.55715 0.31498 1.0000
#End

data_15-crb_Zn
_audit_creation_method ToposPro
_Chemical_Name_Systematic GITTEJ
_cell_length_a 20.19657
_cell_length_b 9.963641
_cell_length_c 20.22194
_cell_angle_alpha 90
_cell_angle_beta 92.33869
_cell_angle_gamma 90
_cell_volume 4065.9
_cell_formula_units_Z 16
_symmetry_space_group_name_H-M 'P 2/n'
_symmetry_Int_Tables_number 13
loop_
_symmetry_equiv_pos_site_id
_symmetry_equiv_pos_as_xyz
1 x,y,z
2 1/2-x,y,1/2-z

N125 N 1 0.58670 0.85439 0.10167 1.0000
N126 N 1 0.35439 0.81163 0.60167 1.0000
N127 N 1 0.95605 0.91330 0.39833 1.0000
N128 N 1 0.54395 0.31163 0.60167 1.0000
N129 N 1 0.48928 0.03806 0.16705 1.0000
N130 N 1 0.96194 0.51072 0.33295 1.0000
N131 N 1 0.84367 0.53806 0.16705 1.0000
N132 N 1 0.34367 0.29489 0.16705 1.0000
N133 N 1 0.01072 0.20511 0.83295 1.0000
N134 N 1 0.03806 0.48928 0.66705 1.0000
N135 N 1 0.53806 0.84367 0.66705 1.0000
N136 N 1 0.20511 0.01072 0.33295 1.0000
N137 N 1 0.29489 0.34367 0.66705 1.0000
N138 N 1 0.15633 0.46194 0.83295 1.0000
N139 N 1 0.65633 0.70511 0.83295 1.0000
N140 N 1 0.98928 0.79489 0.16705 1.0000
N141 N 1 0.51072 0.96194 0.83295 1.0000
N142 N 1 0.46194 0.15633 0.33295 1.0000
N143 N 1 0.79489 0.98928 0.66705 1.0000
N144 N 1 0.70511 0.65633 0.33295 1.0000
N145 N 1 0.10539 0.80111 0.43845 1.0000
N146 N 1 0.19889 0.89461 0.06155 1.0000
N147 N 1 0.95616 0.30111 0.43845 1.0000
N148 N 1 0.45616 0.26044 0.43845 1.0000
N149 N 1 0.39461 0.23956 0.56155 1.0000
N150 N 1 0.80111 0.10539 0.93845 1.0000
N151 N 1 0.30111 0.95616 0.93845 1.0000
N152 N 1 0.23956 0.39461 0.06155 1.0000
N153 N 1 0.26044 0.45616 0.93845 1.0000
N154 N 1 0.04384 0.69889 0.56155 1.0000
N155 N 1 0.54384 0.73956 0.56155 1.0000
N156 N 1 0.60539 0.76044 0.43845 1.0000
N157 N 1 0.89461 0.19889 0.56155 1.0000
N158 N 1 0.69889 0.04384 0.06155 1.0000
N159 N 1 0.76044 0.60539 0.93845 1.0000
N160 N 1 0.73956 0.54384 0.06155 1.0000
Cd1 Cd 1 0.30178 0.00006 0.19749 1.0000
Cd2 Cd 1 0.99994 0.69822 0.30251 1.0000
Cd3 Cd 1 0.00073 0.50006 0.19749 1.0000
Cd4 Cd 1 0.50073 0.30245 0.19749 1.0000
Cd5 Cd 1 0.19822 0.19755 0.80251 1.0000
Cd6 Cd 1 0.00006 0.30178 0.69749 1.0000
Cd7 Cd 1 0.50006 0.00073 0.69749 1.0000
Cd8 Cd 1 0.19755 0.19822 0.30251 1.0000
Cd9 Cd 1 0.30245 0.50073 0.69749 1.0000
Cd10 Cd 1 0.99927 0.49994 0.80251 1.0000
Cd11 Cd 1 0.49927 0.69755 0.80251 1.0000
Cd12 Cd 1 0.80178 0.80245 0.19749 1.0000
Cd13 Cd 1 0.69822 0.99994 0.80251 1.0000
Cd14 Cd 1 0.49994 0.99927 0.30251 1.0000
Cd15 Cd 1 0.80245 0.80178 0.69749 1.0000
Cd16 Cd 1 0.69755 0.49927 0.30251 1.0000
#End

data_15-nog_Cd
_audit_creation_method ToposPro
_Chemical_Name_Systematic HIFWAV
_cell_length_a 25.2742
_cell_length_b 10.27451
_cell_length_c 25.41452
_cell_angle_alpha 90
_cell_angle_beta 91.91953
_cell_angle_gamma 90
_cell_volume 6595.94
_cell_formula_units_Z 20
_symmetry_space_group_name_H-M 'P 21/n'
_symmetry_Int_Tables_number 14
loop_
_symmetry_equiv_pos_site_id
_symmetry_equiv_pos_as_xyz
1 x,y,z
2 1/2-x,1/2+y,1/2-z
3 -x,-y,-z
4 1/2+x,1/2-y,1/2+z
loop_
_atom_site_label
_atom_site_type_symbol
_atom_site_symmetry_multiplicity
_atom_site_fract_x
_atom_site_fract_y
_atom_site_fract_z
_atom_site_occupancy
N1 N 4 0.78666 0.85361 0.72714 1.0000
N2 N 4 0.77632 0.68256 0.76902 1.0000
N3 N 4 0.83930 0.13570 0.66401 1.0000
N4 N 4 0.91465 0.21157 0.67707 1.0000
N5 N 4 0.95735 0.39149 0.78244 1.0000
N6 N 4 0.94624 0.55679 0.82772 1.0000
N7 N 4 0.00649 0.06385 0.76499 1.0000
N8 N 4 0.99713 0.89366 0.80776 1.0000
N9 N 4 0.60863 0.36967 0.61701 1.0000
N10 N 4 0.64603 0.51989 0.57811 1.0000
N11 N 4 0.51437 0.76034 0.43184 1.0000
N12 N 4 0.57869 0.73066 0.48337 1.0000
N13 N 4 0.68402 0.85340 0.55775 1.0000
N14 N 4 0.71846 0.97553 0.61407 1.0000
N15 N 4 0.70709 0.58145 0.45896 1.0000
N16 N 4 0.74715 0.53865 0.39119 1.0000
N17 N 4 0.73613 0.69048 0.26377 1.0000
N18 N 4 0.71401 0.85786 0.22309 1.0000
N19 N 4 0.13594 0.37384 0.67280 1.0000

3 -x,-y,-z
4 1/2+x,-y,1/2+z
loop_
_atom_site_label
_atom_site_type_symbol
_atom_site_symmetry_multiplicity
_atom_site_fract_x
_atom_site_fract_y
_atom_site_fract_z
_atom_site_occupancy
N1 N 4 0.50464 0.29801 0.36002 1.0000
N2 N 4 0.43114 0.32051 0.43397 1.0000
N3 N 4 0.39542 0.20419 0.23625 1.0000
N4 N 4 0.38153 0.18467 0.17438 1.0000
N5 N 4 0.39179 0.98925 0.22044 1.0000
N6 N 4 0.55138 0.94230 0.40833 1.0000
N7 N 4 0.49075 0.79730 0.35395 1.0000
N8 N 4 0.58445 0.83184 0.40713 1.0000
N9 N 4 0.28266 0.02415 0.38423 1.0000
N10 N 4 0.34319 0.94874 0.46686 1.0000
N11 N 4 0.44762 0.14944 0.00329 1.0000
N12 N 4 0.34647 0.21990 0.99308 1.0000
N13 N 4 0.28221 0.93188 0.48105 1.0000
N14 N 4 0.37197 0.74846 0.99069 1.0000
N15 N 4 0.40063 0.69851 0.09210 1.0000
N16 N 4 0.40219 0.58937 0.05824 1.0000
N17 N 4 0.44845 0.26181 0.97178 1.0000
N18 N 4 0.71822 0.55415 0.37861 1.0000
N19 N 4 0.53348 0.45499 0.16173 1.0000
N20 N 4 0.51771 0.46850 0.22237 1.0000
N21 N 4 0.61801 0.54236 0.21529 1.0000
N22 N 4 0.66654 0.42983 0.44767 1.0000
N23 N 4 0.72873 0.42193 0.46340 1.0000
N24 N 4 0.46272 0.43268 0.43091 1.0000
N25 N 4 0.45724 0.23777 0.39014 1.0000
N26 N 4 0.40176 0.08356 0.26443 1.0000
N27 N 4 0.49375 0.92049 0.37553 1.0000
N28 N 4 0.34321 0.00564 0.40727 1.0000
N29 N 4 0.38474 0.12393 0.01614 1.0000
N30 N 4 0.38203 0.79640 0.05034 1.0000
N31 N 4 0.37940 0.05214 0.16486 1.0000
N32 N 4 0.24515 0.97848 0.43005 1.0000
N33 N 4 0.38603 0.30476 0.96563 1.0000
N34 N 4 0.38459 0.62059 0.99583 1.0000
N35 N 4 0.76049 0.49854 0.42072 1.0000
N36 N 4 0.59534 0.50043 0.15751 1.0000
N37 N 4 0.54689 0.74283 0.37358 1.0000
N38 N 4 0.56992 0.52219 0.25523 1.0000
N39 N 4 0.66022 0.51136 0.39547 1.0000
N40 N 4 0.50793 0.41831 0.38531 1.0000
Zn1 Zn 4 0.42315 0.05787 0.36046 1.0000
Zn2 Zn 4 0.35391 0.97935 0.07609 1.0000
Zn3 Zn 4 0.35929 0.48219 0.92775 1.0000
Zn4 Zn 4 0.57179 0.55502 0.35332 1.0000
#End

data_16-crb2_Zn
_audit_creation_method ToposPro
_Chemical_Name_Systematic VEJYEP
_cell_length_a 9.819283
_cell_length_b 14.58515
_cell_length_c 14.62994
_cell_angle_alpha 90
_cell_angle_beta 98.67256
_cell_angle_gamma 90
_cell_volume 2071.282
_cell_formula_units_Z 8
_symmetry_space_group_name_H-M 'P 21/n'
_symmetry_Int_Tables_number 14
loop_
_symmetry_equiv_pos_site_id
_symmetry_equiv_pos_as_xyz
1 x,y,z
2 1/2-x,1/2+y,1/2-z
3 -x,-y,-z
4 1/2+x,1/2-y,1/2+z
loop_
_atom_site_label
_atom_site_type_symbol
_atom_site_symmetry_multiplicity
_atom_site_fract_x
_atom_site_fract_y
_atom_site_fract_z
_atom_site_occupancy
N1 N 4 0.51711 0.90858 0.84575 1.0000
N2 N 4 0.55470 0.89508 0.70418 1.0000
N3 N 4 0.58084 0.81692 0.74368 1.0000
N4 N 4 0.61708 0.18491 0.51932 1.0000
N5 N 4 0.69677 0.13427 0.65604 1.0000
N6 N 4 0.05610 0.03796 0.62014 1.0000
N7 N 4 0.55785 0.28414 0.91697 1.0000
N8 N 4 0.17604 0.00666 0.65400 1.0000
N9 N 4 0.18451 0.15431 0.66673 1.0000
N10 N 4 0.51406 0.15424 0.55529 1.0000
N11 N 4 0.41744 0.16900 0.90972 1.0000
N12 N 4 0.56898 0.23983 0.84243 1.0000
N13 N 4 0.55767 0.82550 0.83080 1.0000
N14 N 4 0.51558 0.95134 0.76729 1.0000
N15 N 4 0.72948 0.17240 0.58166 1.0000
N16 N 4 0.06193 0.12883 0.62817 1.0000
N17 N 4 0.46439 0.24033 0.95813 1.0000
N20 N 4 0.05891 0.31354 0.68427 1.0000
N21 N 4 0.75174 0.78739 0.75266 1.0000
N22 N 4 0.83256 0.79019 0.72761 1.0000
N23 N 4 0.82612 0.68373 0.75376 1.0000
N24 N 4 0.88139 0.12634 0.69483 1.0000
N25 N 4 0.84632 0.22644 0.62747 1.0000
N26 N 4 0.89327 0.27374 0.63563 1.0000
N27 N 4 0.97748 0.50550 0.79344 1.0000
N28 N 4 0.91390 0.37230 0.80967 1.0000
N29 N 4 0.90702 0.47511 0.83786 1.0000
N30 N 4 0.97518 0.00634 0.79755 1.0000
N31 N 4 0.04173 0.88146 0.78180 1.0000
N32 N 4 0.04755 0.98732 0.75512 1.0000
N33 N 4 0.60329 0.44857 0.57696 1.0000
N34 N 4 0.67761 0.48566 0.61865 1.0000
N35 N 4 0.65427 0.39224 0.64293 1.0000
N36 N 4 0.56104 0.70579 0.43558 1.0000
N37 N 4 0.50323 0.81830 0.47697 1.0000
N38 N 4 0.54327 0.79993 0.50903 1.0000
N39 N 4 0.71448 0.85363 0.60022 1.0000
N40 N 4 0.66920 0.97427 0.54547 1.0000
N41 N 4 0.69070 0.05030 0.58055 1.0000
N42 N 4 0.72116 0.63110 0.41425 1.0000
N43 N 4 0.72420 0.45919 0.46349 1.0000
N44 N 4 0.74915 0.43262 0.42132 1.0000
N45 N 4 0.75374 0.80356 0.24880 1.0000
N46 N 4 0.67209 0.77916 0.22221 1.0000
N47 N 4 0.68587 0.67498 0.24756 1.0000
N48 N 4 0.10164 0.35522 0.70922 1.0000
N49 N 4 0.11471 0.34384 0.62568 1.0000
N50 N 4 0.06677 0.30618 0.63282 1.0000
Cd1 Cd 4 0.76532 0.03553 0.68448 1.0000
Cd2 Cd 4 0.98776 0.25043 0.72400 1.0000
Cd3 Cd 4 0.96023 0.75071 0.86139 1.0000
Cd4 Cd 4 0.65705 0.67357 0.51811 1.0000
Cd5 Cd 4 0.78277 0.55255 0.31307 1.0000
#End

data_16-crb_Cd
_audit_creation_method ToposPro
_Chemical_Name_Systematic GITTEJ
_cell_length_a 21.19359
_cell_length_b 10.85576
_cell_length_c 21.22989
_cell_angle_alpha 90
_cell_angle_beta 93.32481
_cell_angle_gamma 90
_cell_volume 4876.194
_cell_formula_units_Z 16
_symmetry_space_group_name_H-M 'P 2/n'
_symmetry_Int_Tables_number 13
loop_
_symmetry_equiv_pos_site_id
_symmetry_equiv_pos_as_xyz
1 x,y,z
2 1/2-x,y,1/2-z
3 -x,-y,-z
4 1/2+x,-y,1/2+z
loop_
_atom_site_label
_atom_site_type_symbol
_atom_site_symmetry_multiplicity
_atom_site_fract_x
_atom_site_fract_y
_atom_site_fract_z
_atom_site_occupancy
N1 N 4 0.50217 0.30559 0.36471 1.0000
N2 N 4 0.43324 0.32257 0.43539 1.0000
N3 N 4 0.39381 0.19513 0.23558 1.0000
N4 N 4 0.38120 0.17547 0.17622 1.0000
N5 N 4 0.39463 0.99773 0.22116 1.0000
N6 N 4 0.55288 0.93089 0.41231 1.0000
N7 N 4 0.49294 0.80483 0.35722 1.0000
N8 N 4 0.58268 0.82742 0.41001 1.0000
N9 N 4 0.28226 0.02089 0.39118 1.0000
N10 N 4 0.34157 0.94641 0.46843 1.0000
N11 N 4 0.45023 0.15049 0.00277 1.0000
N12 N 4 0.35407 0.21721 0.99304 1.0000
N13 N 4 0.28354 0.92800 0.48138 1.0000
N14 N 4 0.37605 0.73815 0.99477 1.0000
N15 N 4 0.40524 0.69943 0.09214 1.0000
N16 N 4 0.40715 0.59705 0.06144 1.0000
N17 N 4 0.45136 0.25614 0.97481 1.0000
N18 N 4 0.71860 0.55025 0.38416 1.0000
N19 N 4 0.53315 0.45185 0.16413 1.0000
N20 N 4 0.51876 0.46333 0.22207 1.0000
N21 N 4 0.61141 0.54392 0.21453 1.0000
N22 N 4 0.67084 0.43314 0.44881 1.0000
N23 N 4 0.73061 0.42608 0.46371 1.0000
N24 N 4 0.46160 0.42753 0.43197 1.0000
N25 N 4 0.45848 0.24784 0.39391 1.0000
N26 N 4 0.40206 0.08552 0.26299 1.0000
N27 N 4 0.49775 0.91651 0.37977 1.0000
N28 N 4 0.34050 0.00362 0.41297 1.0000
N29 N 4 0.39030 0.12690 0.01377 1.0000
N30 N 4 0.38618 0.78598 0.05084 1.0000
N31 N 4 0.38181 0.05390 0.16764 1.0000
N32 N 4 0.24729 0.97396 0.43374 1.0000
N33 N 4 0.39205 0.29669 0.96890 1.0000
N34 N 4 0.38928 0.62149 0.00161 1.0000
N35 N 4 0.75978 0.49821 0.42376 1.0000

N18 N 4 0.25483 0.07868 0.68258 1.0000
N19 N 4 0.48243 0.16891 0.83830 1.0000
N20 N 4 0.56378 0.12321 0.63951 1.0000
Zn1 Zn 4 0.45497 0.07859 0.73566 1.0000
Zn2 Zn 4 0.58202 0.72054 0.92050 1.0000
#End

data_17-4,4L37_Zn
_audit_creation_method ToposPro
_Chemical_Name_Systematic CUIMDZ02
_cell_length_a 19.0884
_cell_length_b 18.21283
_cell_length_c 9.426107
_cell_angle_alpha 90
_cell_angle_beta 90
_cell_angle_gamma 90
_cell_volume 3277.022
_cell_formula_units_Z 20
_symmetry_space_group_name_H-M 'C c c a'
_symmetry_Int_Tables_number 68
loop_
_symmetry_equiv_pos_site_id
_symmetry_equiv_pos_as_xyz
1 x,y,z
2 1/2-x,-y,z
3 1/2+x,-y,1/2-z
4 -x,y,1/2-z
5 -x,-y,-z
6 1/2+x,y,-z
7 1/2-x,y,1/2+z
8 x,-y,1/2+z
9 1/2+x,1/2+y,z
10 -x,1/2-y,z
11 x,1/2-y,1/2-z
12 1/2-x,1/2+y,1/2-z
13 1/2-x,1/2-y,-z
14 x,1/2+y,-z
15 -x,1/2+y,1/2+z
16 1/2+x,1/2-y,1/2+z
loop_
_atom_site_label
_atom_site_type_symbol
_atom_site_symmetry_multiplicity
_atom_site_fract_x
_atom_site_fract_y
_atom_site_fract_z
_atom_site_occupancy
N1 N 16 0.92068 0.32218 0.98851 1.0000
N2 N 16 0.97388 0.32414 0.90254 1.0000
N3 N 16 0.93600 0.36767 0.09108 1.0000
N4 N 16 0.02233 0.37036 0.95165 1.0000
N5 N 16 0.99839 0.39751 0.06858 1.0000
N6 N 16 0.86443 0.49637 0.95926 1.0000
N7 N 16 0.84144 0.49419 0.08937 1.0000
N8 N 16 0.84399 0.55940 0.90945 1.0000
N9 N 16 0.80719 0.55581 0.11992 1.0000
N10 N 16 0.80864 0.59633 0.00800 1.0000
N11 N 16 0.80779 0.30609 0.22999 1.0000
N12 N 8 0.84903 0.25000 0.25000 1.0000
N13 N 16 0.74124 0.28489 0.23802 1.0000
Zn1 Zn 4 0.00000 0.25000 0.75000 1.0000
Zn2 Zn 16 0.85534 0.40467 0.21180 1.0000
#End

data_18-cag_Zn
_audit_creation_method ToposPro
_Chemical_Name_Systematic IMIDZB11
_cell_length_a 15.32422
_cell_length_b 15.34115
_cell_length_c 17.56315
_cell_angle_alpha 90
_cell_angle_beta 90
_cell_angle_gamma 90
_cell_volume 4128.941
_cell_formula_units_Z 16
_symmetry_space_group_name_H-M 'P b c a'
_symmetry_Int_Tables_number 61
loop_
_symmetry_equiv_pos_site_id
_symmetry_equiv_pos_as_xyz
1 x,y,z
2 1/2-x,-y,1/2+z
3 1/2+x,1/2-y,-z
4 -x,1/2+y,1/2-z
5 -x,-y,-z
6 1/2+x,y,1/2-z
7 1/2-x,1/2+y,z
8 x,1/2-y,1/2+z
loop_
_atom_site_label
_atom_site_type_symbol
_atom_site_symmetry_multiplicity
_atom_site_fract_x
_atom_site_fract_y
_atom_site_fract_z
_atom_site_occupancy
N1 N 8 0.17190 0.32529 0.11228 1.0000
N2 N 8 0.06517 0.25447 0.15009 1.0000
N3 N 8 0.14999 0.25245 0.14557 1.0000
N4 N 8 0.03468 0.32813 0.11979 1.0000
N5 N 8 0.10104 0.37216 0.09629 1.0000

N36 N 4 0.59020 0.50151 0.15974 1.0000
N37 N 4 0.54560 0.75019 0.37607 1.0000
N38 N 4 0.56705 0.51997 0.25287 1.0000
N39 N 4 0.66370 0.50961 0.39990 1.0000
N40 N 4 0.50389 0.41650 0.38845 1.0000
Cd1 Cd 4 0.42510 0.06191 0.36558 1.0000
Cd2 Cd 4 0.35794 0.97385 0.07338 1.0000
Cd3 Cd 4 0.36394 0.47951 0.92995 1.0000
Cd4 Cd 4 0.56989 0.55756 0.35600 1.0000
#End

data_17-crb2_Cd
_audit_creation_method ToposPro
_Chemical_Name_Systematic VEJYEP
_cell_length_a 10.71266
_cell_length_b 15.54381
_cell_length_c 15.39734
_cell_angle_alpha 90
_cell_angle_beta 99.80994
_cell_angle_gamma 90
_cell_volume 2526.408
_cell_formula_units_Z 8
_symmetry_space_group_name_H-M 'P 21/n'
_symmetry_Int_Tables_number 14
loop_
_symmetry_equiv_pos_site_id
_symmetry_equiv_pos_as_xyz
1 x,y,z
2 1/2-x,1/2+y,1/2-z
3 -x,-y,-z
4 1/2+x,1/2-y,1/2+z
loop_
_atom_site_label
_atom_site_type_symbol
_atom_site_symmetry_multiplicity
_atom_site_fract_x
_atom_site_fract_y
_atom_site_fract_z
_atom_site_occupancy
N1 N 4 0.51872 0.90439 0.84102 1.0000
N2 N 4 0.55362 0.89279 0.70703 1.0000
N3 N 4 0.58133 0.81981 0.74537 1.0000
N4 N 4 0.61913 0.18380 0.52268 1.0000
N5 N 4 0.69065 0.13552 0.65364 1.0000
N6 N 4 0.06303 0.03910 0.62225 1.0000
N7 N 4 0.55227 0.28122 0.91474 1.0000
N8 N 4 0.17517 0.01036 0.65269 1.0000
N9 N 4 0.18191 0.14897 0.66391 1.0000
N10 N 4 0.52350 0.15548 0.55507 1.0000
N11 N 4 0.42373 0.17308 0.90770 1.0000
N12 N 4 0.56264 0.23836 0.84456 1.0000
N13 N 4 0.55981 0.82727 0.82773 1.0000
N14 N 4 0.51527 0.94460 0.76615 1.0000
N15 N 4 0.72177 0.17126 0.58359 1.0000
N16 N 4 0.06775 0.12431 0.62937 1.0000
N17 N 4 0.46656 0.24085 0.95324 1.0000
N18 N 4 0.24795 0.07829 0.67821 1.0000
N19 N 4 0.48344 0.17184 0.84060 1.0000
N20 N 4 0.56811 0.12588 0.63559 1.0000
Cd1 Cd 4 0.45315 0.07707 0.73190 1.0000
Cd2 Cd 4 0.58539 0.71662 0.92110 1.0000
#End

data_18-dia_Cd
_audit_creation_method ToposPro
_Chemical_Name_Systematic OFERUN01
_cell_length_a 17.73953
_cell_length_b 8.268542
_cell_length_c 15.52659
_cell_angle_alpha 90
_cell_angle_beta 106.3143
_cell_angle_gamma 90
_cell_volume 2185.74
_cell_formula_units_Z 8
_symmetry_space_group_name_H-M 'P 21/c'
_symmetry_Int_Tables_number 14
loop_
_symmetry_equiv_pos_site_id
_symmetry_equiv_pos_as_xyz
1 x,y,z
2 -x,1/2+y,1/2-z
3 -x,-y,-z
4 x,1/2-y,1/2+z
loop_
_atom_site_label
_atom_site_type_symbol
_atom_site_symmetry_multiplicity
_atom_site_fract_x
_atom_site_fract_y
_atom_site_fract_z
_atom_site_occupancy
N1 N 4 0.26903 0.85563 0.20194 1.0000
N2 N 4 0.18526 0.71419 0.23621 1.0000
N3 N 4 0.30769 0.04830 0.03144 1.0000
N4 N 4 0.22242 0.06421 0.90899 1.0000
N5 N 4 0.44024 0.11863 0.26414 1.0000
N6 N 4 0.53188 0.27130 0.32784 1.0000
N7 N 4 0.06120 0.31331 0.20912 1.0000
N8 N 4 0.96900 0.14907 0.18202 1.0000
N9 N 4 0.25387 0.70765 0.22082 1.0000
N10 N 4 0.15829 0.86532 0.22720 1.0000

N6 N 8 0.33750 0.45637 0.15470 1.0000
N7 N 8 0.41751 0.55619 0.19278 1.0000
N8 N 8 0.40456 0.50377 0.13538 1.0000
N9 N 8 0.35869 0.54146 0.24724 1.0000
N10 N 8 0.30913 0.47925 0.22363 1.0000
N11 N 8 0.36387 0.25442 0.13595 1.0000
N12 N 8 0.43703 0.18214 0.21252 1.0000
N13 N 8 0.41163 0.26138 0.19701 1.0000
N14 N 8 0.40506 0.12650 0.16144 1.0000
N15 N 8 0.35965 0.17147 0.11377 1.0000
N16 N 8 0.32836 0.37041 0.98429 1.0000
N17 N 8 0.40094 0.36892 0.88473 1.0000
N18 N 8 0.40604 0.35359 0.95758 1.0000
N19 N 8 0.32061 0.39497 0.86632 1.0000
N20 N 8 0.27553 0.39597 0.92823 1.0000
Zn1 Zn 8 0.29607 0.35493 0.09334 1.0000
Zn2 Zn 8 0.99629 0.15358 0.19059 1.0000
#End

data_19-kat1_Zn
_audit_creation_method TopósPro
_Chemical_Name_Systematic OFERUN08
_cell_length_a 15.68606
_cell_length_b 15.68606
_cell_length_c 15.68969
_cell_angle_alpha 90
_cell_angle_beta 90
_cell_angle_gamma 90
_cell_volume 3860.487
_cell_formula_units_Z 16
_symmetry_space_group_name_H-M 'P -4 2 c'
_symmetry_Int_Tables_number 112
loop_
_symmetry_equiv_pos_site_id
_symmetry_equiv_pos_as_xyz
1 x,y,z
2 -x,-y,z
3 x,-y,1/2-z
4 -x,y,1/2-z
5 y,x,1/2+z
6 -y,-x,1/2+z
7 -y,x,-z
8 y,-x,-z
loop_
_atom_site_label
_atom_site_type_symbol
_atom_site_symmetry_multiplicity
_atom_site_fract_x
_atom_site_fract_y
_atom_site_fract_z
_atom_site_occupancy
N1 N 8 0.66665 0.42162 0.51279 1.0000
N2 N 8 0.72619 0.42774 0.38849 1.0000
N3 N 8 0.68998 0.35437 0.39462 1.0000
N4 N 8 0.65350 0.35083 0.47125 1.0000
N5 N 8 0.71167 0.46893 0.46148 1.0000
N6 N 8 0.53407 0.13273 0.37565 1.0000
N7 N 8 0.66810 0.12049 0.40589 1.0000
N8 N 8 0.65141 0.06601 0.34678 1.0000
N9 N 8 0.56885 0.07378 0.32821 1.0000
N10 N 8 0.59567 0.16146 0.42342 1.0000
N11 N 8 0.63023 0.11765 0.63684 1.0000
N12 N 8 0.68234 0.23907 0.67657 1.0000
N13 N 8 0.70717 0.18303 0.73098 1.0000
N14 N 8 0.67467 0.10825 0.70651 1.0000
N15 N 8 0.63507 0.19864 0.61871 1.0000
N16 N 8 0.87026 0.00055 0.63559 1.0000
N17 N 8 0.78037 0.90492 0.59312 1.0000
N18 N 8 0.84307 0.90496 0.54008 1.0000
N19 N 8 0.89831 0.96393 0.56660 1.0000
N20 N 8 0.79756 0.96374 0.65192 1.0000
Zn1 Zn 2 0.00000 0.00000 0.50000 1.0000
Zn2 Zn 2 0.50000 0.00000 0.25000 1.0000
Zn3 Zn 4 0.72440 0.00000 0.75000 1.0000
Zn4 Zn 8 0.58761 0.25112 0.51328 1.0000
#End

data_20-neb_Zn
_audit_creation_method TopósPro
_Chemical_Name_Systematic EQOBUH
_cell_length_a 14.95549
_cell_length_b 10.21584
_cell_length_c 12.65008
_cell_angle_alpha 80.93518
_cell_angle_beta 42.41936
_cell_angle_gamma 56.64545
_cell_volume 977.4947
_cell_formula_units_Z 4
_symmetry_space_group_name_H-M 'P 1'
_symmetry_Int_Tables_number 1
loop_
_symmetry_equiv_pos_site_id
_symmetry_equiv_pos_as_xyz
1 x,y,z
loop_
_atom_site_label
_atom_site_type_symbol
_atom_site_symmetry_multiplicity
_atom_site_fract_x
_atom_site_fract_y
_atom_site_fract_z
N11 N 4 0.21033 0.95324 0.20569 1.0000
N12 N 4 0.25964 0.96110 0.96978 1.0000
N13 N 4 0.24707 0.21392 0.93288 1.0000
N14 N 4 0.30009 0.20403 0.00918 1.0000
N15 N 4 0.50069 0.19588 0.25228 1.0000
N16 N 4 0.49101 0.24115 0.38589 1.0000
N17 N 4 0.43403 0.14568 0.34629 1.0000
N18 N 4 0.01748 0.22781 0.24713 1.0000
N19 N 4 0.98236 0.18545 0.10417 1.0000
N20 N 4 0.03974 0.28789 0.12097 1.0000
Cd1 Cd 4 0.37189 0.94614 0.16233 1.0000
Cd2 Cd 4 0.12904 0.51117 0.28860 1.0000
#End

data_19-neb_Cd
_audit_creation_method TopósPro
_Chemical_Name_Systematic EQOBUH
_cell_length_a 16.0055
_cell_length_b 10.9883
_cell_length_c 13.4744
_cell_angle_alpha 81.04
_cell_angle_beta 42.7
_cell_angle_gamma 56.26
_cell_volume 1195.026
_cell_formula_units_Z 4
_symmetry_space_group_name_H-M 'P 1'
_symmetry_Int_Tables_number 1
loop_
_symmetry_equiv_pos_site_id
_symmetry_equiv_pos_as_xyz
1 x,y,z
loop_
_atom_site_label
_atom_site_type_symbol
_atom_site_symmetry_multiplicity
_atom_site_fract_x
_atom_site_fract_y
_atom_site_fract_z
_atom_site_occupancy
N1 N 1 0.57551 0.85479 0.43695 1.0000
N2 N 1 0.39521 0.67449 0.11725 1.0000
N3 N 1 0.13275 0.43695 0.85479 1.0000
N4 N 1 0.81305 0.11725 0.67449 1.0000
N5 N 1 0.19625 0.99886 0.62215 1.0000
N6 N 1 0.25114 0.05375 0.06726 1.0000
N7 N 1 0.18274 0.62215 0.99886 1.0000
N8 N 1 0.62785 0.06726 0.05375 1.0000
N9 N 1 0.98778 0.03915 0.75873 1.0000
N10 N 1 0.21085 0.26222 0.03566 1.0000
N11 N 1 0.21434 0.75873 0.03915 1.0000
N12 N 1 0.49127 0.03566 0.26222 1.0000
N13 N 1 0.82191 0.67424 0.27934 1.0000
N14 N 1 0.57576 0.42809 0.02548 1.0000
N15 N 1 0.22452 0.27934 0.67424 1.0000
N16 N 1 0.97066 0.02548 0.42809 1.0000
N17 N 1 0.23082 0.85688 0.61969 1.0000
N18 N 1 0.39312 0.01918 0.95739 1.0000
N19 N 1 0.29261 0.61969 0.85688 1.0000
N20 N 1 0.63031 0.95739 0.01918 1.0000
N21 N 1 0.10110 0.88191 0.70455 1.0000
N22 N 1 0.36809 0.14890 0.93757 1.0000
N23 N 1 0.31243 0.70455 0.88191 1.0000
N24 N 1 0.54545 0.93757 0.14890 1.0000
N25 N 1 0.04599 0.11216 0.70794 1.0000
N26 N 1 0.13784 0.20401 0.11609 1.0000
N27 N 1 0.13391 0.70794 0.11216 1.0000
N28 N 1 0.54206 0.11609 0.20401 1.0000
N29 N 1 0.58443 0.81874 0.52928 1.0000
N30 N 1 0.43126 0.66557 0.18245 1.0000
N31 N 1 0.06755 0.52928 0.81874 1.0000
N32 N 1 0.72072 0.18245 0.66557 1.0000
N33 N 1 0.73791 0.70620 0.43111 1.0000
N34 N 1 0.54380 0.51209 0.12523 1.0000
N35 N 1 0.12477 0.43111 0.70620 1.0000
N36 N 1 0.81889 0.12523 0.51209 1.0000
N37 N 1 0.72205 0.76587 0.28228 1.0000
N38 N 1 0.48413 0.52795 0.02020 1.0000
N39 N 1 0.22980 0.28228 0.76587 1.0000
N40 N 1 0.96772 0.02020 0.52795 1.0000
Cd1 Cd 1 0.36258 0.04300 0.50438 1.0000
Cd2 Cd 1 0.20700 0.88742 0.15995 1.0000
Cd3 Cd 1 0.09005 0.50438 0.04300 1.0000
Cd4 Cd 1 0.74562 0.15995 0.88742 1.0000
#End

data_20-hcb_Cd
_audit_creation_method TopósPro
_Chemical_Name_Systematic CAYSEB
_cell_length_a 7.573424
_cell_length_b 10.66568
_cell_length_c 14.194
_cell_angle_alpha 90
_cell_angle_beta 116.0631
_cell_angle_gamma 90
_cell_volume 1029.94
_cell_formula_units_Z 4
_symmetry_space_group_name_H-M 'P 1'
_symmetry_Int_Tables_number 1
loop_
_symmetry_equiv_pos_site_id
_symmetry_equiv_pos_as_xyz

_atom_site_occupancy
N1 N 1 0.56775 0.86109 0.43964 1.0000
N2 N 1 0.38891 0.68225 0.11848 1.0000
N3 N 1 0.13152 0.43964 0.86109 1.0000
N4 N 1 0.81036 0.11848 0.68225 1.0000
N5 N 1 0.20351 0.00005 0.61635 1.0000
N6 N 1 0.24995 0.04649 0.06992 1.0000
N7 N 1 0.18008 0.61635 0.00005 1.0000
N8 N 1 0.63365 0.06992 0.04649 1.0000
N9 N 1 0.97858 0.04558 0.76422 1.0000
N10 N 1 0.20442 0.27142 0.03838 1.0000
N11 N 1 0.21162 0.76422 0.04558 1.0000
N12 N 1 0.48578 0.03838 0.27142 1.0000
N13 N 1 0.83000 0.66690 0.27162 1.0000
N14 N 1 0.58310 0.42000 0.01852 1.0000
N15 N 1 0.23148 0.27162 0.66690 1.0000
N16 N 1 0.97838 0.01852 0.42000 1.0000
N17 N 1 0.23813 0.84854 0.61382 1.0000
N18 N 1 0.40146 0.01187 0.95049 1.0000
N19 N 1 0.29951 0.61382 0.84854 1.0000
N20 N 1 0.63618 0.95049 0.01187 1.0000
N21 N 1 0.09836 0.87678 0.70555 1.0000
N22 N 1 0.37322 0.15164 0.93069 1.0000
N23 N 1 0.31931 0.70555 0.87678 1.0000
N24 N 1 0.54445 0.93069 0.15164 1.0000
N25 N 1 0.04307 0.12229 0.70911 1.0000
N26 N 1 0.12771 0.20693 0.12447 1.0000
N27 N 1 0.12553 0.70911 0.12229 1.0000
N28 N 1 0.54089 0.12447 0.20693 1.0000
N29 N 1 0.57727 0.82389 0.53830 1.0000
N30 N 1 0.42611 0.67273 0.18946 1.0000
N31 N 1 0.06054 0.53830 0.82389 1.0000
N32 N 1 0.71170 0.18946 0.67273 1.0000
N33 N 1 0.74031 0.70306 0.43396 1.0000
N34 N 1 0.54694 0.50969 0.12733 1.0000
N35 N 1 0.12267 0.43396 0.70306 1.0000
N36 N 1 0.81604 0.12733 0.50969 1.0000
N37 N 1 0.72380 0.76431 0.27474 1.0000
N38 N 1 0.48569 0.52620 0.01285 1.0000
N39 N 1 0.23715 0.27474 0.76431 1.0000
N40 N 1 0.97526 0.01285 0.52620 1.0000
Zn1 Zn 1 0.36395 0.04450 0.50392 1.0000
Zn2 Zn 1 0.20550 0.88605 0.16237 1.0000
Zn3 Zn 1 0.08763 0.50392 0.04450 1.0000
Zn4 Zn 1 0.74608 0.16237 0.88605 1.0000
#End

data_21-dia_Zn
_audit_creation_method ToposPro
_Chemical_Name_Systematic OFERUN01
_cell_length_a 16.38752
_cell_length_b 7.81097
_cell_length_c 14.98104
_cell_angle_alpha 90
_cell_angle_beta 108.495
_cell_angle_gamma 90
_cell_volume 1818.567
_cell_formula_units_Z 8
_symmetry_space_group_name_H-M 'P 21/c'
_symmetry_Int_Tables_number 14
loop_
_symmetry_equiv_pos_site_id
_symmetry_equiv_pos_as_xyz
1 x,y,z
2 -x,1/2+y,1/2-z
3 -x,-y,-z
4 x,1/2-y,1/2+z
loop_
_atom_site_label
_atom_site_type_symbol
_atom_site_symmetry_multiplicity
_atom_site_fract_x
_atom_site_fract_y
_atom_site_fract_z
_atom_site_occupancy
N1 N 4 0.27304 0.85523 0.20095 1.0000
N2 N 4 0.18356 0.70689 0.23720 1.0000
N3 N 4 0.31040 0.04360 0.03709 1.0000
N4 N 4 0.21935 0.05860 0.90470 1.0000
N5 N 4 0.43611 0.11385 0.26004 1.0000
N6 N 4 0.53645 0.27588 0.32925 1.0000
N7 N 4 0.06562 0.32217 0.21197 1.0000
N8 N 4 0.96647 0.14487 0.18354 1.0000
N9 N 4 0.25696 0.69884 0.22061 1.0000
N10 N 4 0.15467 0.86771 0.22821 1.0000
N11 N 4 0.21010 0.95961 0.20543 1.0000
N12 N 4 0.25917 0.95043 0.97038 1.0000
N13 N 4 0.24573 0.21769 0.93054 1.0000
N14 N 4 0.30225 0.20849 0.01292 1.0000
N15 N 4 0.50132 0.19697 0.24956 1.0000
N16 N 4 0.49309 0.24192 0.38885 1.0000
N17 N 4 0.43079 0.14063 0.34588 1.0000
N18 N 4 0.01962 0.23152 0.25144 1.0000
N19 N 4 0.97949 0.18065 0.10214 1.0000
N20 N 4 0.04111 0.29118 0.11978 1.0000
Zn1 Zn 4 0.37093 0.94544 0.16278 1.0000
Zn2 Zn 4 0.12904 0.51694 0.28777 1.0000
#End

data_22-lon_Zn
_audit_creation_method ToposPro
1 x,y,z
loop_
_atom_site_label
_atom_site_type_symbol
_atom_site_symmetry_multiplicity
_atom_site_fract_x
_atom_site_fract_y
_atom_site_fract_z
_atom_site_occupancy
N1 N 1 0.47112 0.92958 0.24792 1.0000
N2 N 1 0.52888 0.07042 0.75208 1.0000
N3 N 1 0.47112 0.57042 0.74792 1.0000
N4 N 1 0.52888 0.42958 0.25208 1.0000
N5 N 1 0.25844 0.06591 0.97808 1.0000
N6 N 1 0.74156 0.93409 0.02192 1.0000
N7 N 1 0.25844 0.43409 0.47808 1.0000
N8 N 1 0.74156 0.56591 0.52192 1.0000
N9 N 1 0.48546 0.80891 0.23842 1.0000
N10 N 1 0.51454 0.19109 0.76158 1.0000
N11 N 1 0.48546 0.69109 0.73842 1.0000
N12 N 1 0.51454 0.30891 0.26158 1.0000
N13 N 1 0.42565 0.84949 0.37100 1.0000
N14 N 1 0.57435 0.15051 0.62900 1.0000
N15 N 1 0.42565 0.65051 0.87100 1.0000
N16 N 1 0.57435 0.34949 0.12900 1.0000
N17 N 1 0.43423 0.95524 0.32930 1.0000
N18 N 1 0.56577 0.04476 0.67070 1.0000
N19 N 1 0.43423 0.54476 0.82930 1.0000
N20 N 1 0.56577 0.45524 0.17070 1.0000
N21 N 1 0.09046 0.12002 0.95679 1.0000
N22 N 1 0.90954 0.87998 0.04321 1.0000
N23 N 1 0.09046 0.37998 0.45679 1.0000
N24 N 1 0.90954 0.62002 0.54321 1.0000
N25 N 1 0.25571 0.02748 0.88890 1.0000
N26 N 1 0.74429 0.97252 0.11110 1.0000
N27 N 1 0.25571 0.47252 0.38890 1.0000
N28 N 1 0.74429 0.52748 0.61110 1.0000
N29 N 1 0.98432 0.11501 0.85458 1.0000
N30 N 1 0.01568 0.88499 0.14542 1.0000
N31 N 1 0.98432 0.38499 0.35458 1.0000
N32 N 1 0.01568 0.61501 0.64542 1.0000
N33 N 1 0.08623 0.05789 0.81252 1.0000
N34 N 1 0.91377 0.94211 0.18748 1.0000
N35 N 1 0.08623 0.44211 0.31252 1.0000
N36 N 1 0.91377 0.55789 0.68748 1.0000
N37 N 1 0.45693 0.75983 0.31468 1.0000
N38 N 1 0.54307 0.24017 0.68532 1.0000
N39 N 1 0.45693 0.74017 0.81468 1.0000
N40 N 1 0.54307 0.25983 0.18532 1.0000
Cd1 Cd 1 0.50600 0.06207 0.14000 1.0000
Cd2 Cd 1 0.49400 0.93793 0.86000 1.0000
Cd3 Cd 1 0.50600 0.43793 0.64000 1.0000
Cd4 Cd 1 0.49400 0.56207 0.36000 1.0000
#End

data_21-kat1_Cd
_audit_creation_method ToposPro
_Chemical_Name_Systematic OFERUN08
_cell_length_a 16.74606
_cell_length_b 16.74606
_cell_length_c 16.86488
_cell_angle_alpha 90
_cell_angle_beta 90
_cell_angle_gamma 90
_cell_volume 4729.426
_cell_formula_units_Z 16
_symmetry_space_group_name_H-M 'P -4 2 c'
_symmetry_Int_Tables_number 112
loop_
_symmetry_equiv_pos_site_id
_symmetry_equiv_pos_as_xyz
1 x,y,z
2 -x,-y,z
3 x,-y,1/2-z
4 -x,y,1/2-z
5 y,x,1/2+z
6 -y,-x,1/2+z
7 -y,x,-z
8 y,-x,-z
loop_
_atom_site_label
_atom_site_type_symbol
_atom_site_symmetry_multiplicity
_atom_site_fract_x
_atom_site_fract_y
_atom_site_fract_z
_atom_site_occupancy
N1 N 8 0.66824 0.42021 0.50749 1.0000
N2 N 8 0.72426 0.42667 0.39206 1.0000
N3 N 8 0.69025 0.35772 0.39733 1.0000
N4 N 8 0.65592 0.35402 0.46843 1.0000
N5 N 8 0.71056 0.46488 0.46002 1.0000
N6 N 8 0.53876 0.13099 0.37602 1.0000
N7 N 8 0.66438 0.11915 0.40347 1.0000
N8 N 8 0.64823 0.06826 0.34823 1.0000
N9 N 8 0.57093 0.07584 0.33148 1.0000
N10 N 8 0.59686 0.15759 0.42028 1.0000
N11 N 8 0.63344 0.12118 0.63948 1.0000
N12 N 8 0.68007 0.23559 0.67691 1.0000
N13 N 8 0.70380 0.18321 0.72779 1.0000
N14 N 8 0.67471 0.11290 0.70467 1.0000

_Chemical_Name_Systematic SIVGEL
_cell_length_a 15.63506
_cell_length_b 9.666062
_cell_length_c 9.666062
_cell_angle_alpha 58.4057
_cell_angle_beta 90
_cell_angle_gamma 90
_cell_volume 1244.302
_cell_formula_units_Z 4
_symmetry_space_group_name_H-M 'P 1'
_symmetry_Int_Tables_number 1
loop_
_symmetry_equiv_pos_site_id
_symmetry_equiv_pos_as_xyz
1 x,y,z
loop_
_atom_site_label
_atom_site_type_symbol
_atom_site_symmetry_multiplicity
_atom_site_fract_x
_atom_site_fract_y
_atom_site_fract_z
_atom_site_occupancy
N1 N 1 0.94650 0.59265 0.92598 1.0000
N2 N 1 0.05350 0.07402 0.40735 1.0000
N3 N 1 0.55350 0.59265 0.92598 1.0000
N4 N 1 0.44650 0.07402 0.40735 1.0000
N5 N 1 0.18372 0.77002 0.55167 1.0000
N6 N 1 0.81628 0.44833 0.22998 1.0000
N7 N 1 0.31628 0.77002 0.55167 1.0000
N8 N 1 0.68372 0.44833 0.22998 1.0000
N9 N 1 0.02182 0.79644 0.31948 1.0000
N10 N 1 0.97818 0.68052 0.20356 1.0000
N11 N 1 0.47818 0.79644 0.31948 1.0000
N12 N 1 0.52182 0.68052 0.20356 1.0000
N13 N 1 0.20882 0.63323 0.68509 1.0000
N14 N 1 0.79118 0.31491 0.36677 1.0000
N15 N 1 0.29118 0.63323 0.68509 1.0000
N16 N 1 0.70882 0.31491 0.36677 1.0000
N17 N 1 0.99074 0.70646 0.68916 1.0000
N18 N 1 0.00926 0.31084 0.29354 1.0000
N19 N 1 0.50926 0.70646 0.68916 1.0000
N20 N 1 0.49074 0.31084 0.29354 1.0000
N21 N 1 0.91722 0.63371 0.69446 1.0000
N22 N 1 0.08278 0.30554 0.36629 1.0000
N23 N 1 0.58278 0.63371 0.69446 1.0000
N24 N 1 0.41722 0.30554 0.36629 1.0000
N25 N 1 0.25000 0.85488 0.46870 1.0000
N26 N 1 0.75000 0.53130 0.14512 1.0000
N27 N 1 0.00906 0.68132 0.83213 1.0000
N28 N 1 0.99094 0.16787 0.31868 1.0000
N29 N 1 0.49094 0.68132 0.83213 1.0000
N30 N 1 0.50906 0.16787 0.31868 1.0000
N31 N 1 0.88976 0.56280 0.84174 1.0000
N32 N 1 0.11024 0.15826 0.43720 1.0000
N33 N 1 0.61024 0.56280 0.84174 1.0000
N34 N 1 0.38976 0.15826 0.43720 1.0000
N35 N 1 0.01361 0.90971 0.16233 1.0000
N36 N 1 0.98639 0.83767 0.09029 1.0000
N37 N 1 0.48639 0.90971 0.16233 1.0000
N38 N 1 0.51361 0.83767 0.09029 1.0000
N39 N 1 0.00000 0.65460 0.34540 1.0000
N40 N 1 0.50000 0.65460 0.34540 1.0000
Zn1 Zn 1 0.06210 0.83764 0.49052 1.0000
Zn2 Zn 1 0.93790 0.50948 0.16236 1.0000
Zn3 Zn 1 0.43790 0.83764 0.49052 1.0000
Zn4 Zn 1 0.56210 0.50948 0.16236 1.0000
#End

data_23-sod_Zn
_audit_creation_method ToposPro
_Chemical_Name_Systematic OFERUN03
_cell_length_a 14.33642
_cell_length_b 14.33642
_cell_length_c 14.33642
_cell_angle_alpha 109.4712
_cell_angle_beta 109.4712
_cell_angle_gamma 109.4712
_cell_volume 2268.297
_cell_formula_units_Z 6
_symmetry_space_group_name_H-M 'P 1'
_symmetry_Int_Tables_number 1
loop_
_symmetry_equiv_pos_site_id
_symmetry_equiv_pos_as_xyz
1 x,y,z
loop_
_atom_site_label
_atom_site_type_symbol
_atom_site_symmetry_multiplicity
_atom_site_fract_x
_atom_site_fract_y
_atom_site_fract_z
_atom_site_occupancy
N1 N 1 0.61305 0.24315 0.61305 1.0000
N2 N 1 0.36990 0.36990 0.75685 1.0000
N3 N 1 0.00000 0.63010 0.38695 1.0000
N4 N 1 0.00000 0.38695 0.63010 1.0000
N5 N 1 0.61305 0.61305 0.24315 1.0000
N6 N 1 0.24315 0.61305 0.61305 1.0000
N7 N 1 0.63010 0.00000 0.38695 1.0000
N15 N 8 0.63684 0.19715 0.62276 1.0000
N16 N 8 0.86797 0.99671 0.63216 1.0000
N17 N 8 0.78312 0.90745 0.59349 1.0000
N18 N 8 0.84152 0.90736 0.54341 1.0000
N19 N 8 0.89355 0.96233 0.56758 1.0000
N20 N 8 0.79979 0.96243 0.64802 1.0000
Cd1 Cd 2 0.00000 0.00000 0.50000 1.0000
Cd2 Cd 2 0.50000 0.00000 0.25000 1.0000
Cd3 Cd 4 0.72418 0.00000 0.75000 1.0000
Cd4 Cd 8 0.58803 0.25012 0.51314 1.0000
#End

data_22-cag_Cd
_audit_creation_method ToposPro
_Chemical_Name_Systematic IMIDZB11
_cell_length_a 16.41621
_cell_length_b 16.34026
_cell_length_c 18.83387
_cell_angle_alpha 90
_cell_angle_beta 90
_cell_angle_gamma 90
_cell_volume 5052.093
_cell_formula_units_Z 16
_symmetry_space_group_name_H-M 'P b c a'
_symmetry_Int_Tables_number 61
loop_
_symmetry_equiv_pos_site_id
_symmetry_equiv_pos_as_xyz
1 x,y,z
2 1/2-x,-y,1/2+z
3 1/2+x,1/2-y,-z
4 -x,1/2+y,1/2-z
5 -x,-y,-z
6 1/2+x,y,1/2-z
7 1/2-x,1/2+y,z
8 x,1/2-y,1/2+z
loop_
_atom_site_label
_atom_site_type_symbol
_atom_site_symmetry_multiplicity
_atom_site_fract_x
_atom_site_fract_y
_atom_site_fract_z
_atom_site_occupancy
N1 N 8 0.16702 0.32404 0.11285 1.0000
N2 N 8 0.06766 0.25738 0.14880 1.0000
N3 N 8 0.14690 0.25561 0.14415 1.0000
N4 N 8 0.03888 0.32623 0.12050 1.0000
N5 N 8 0.10077 0.36778 0.09816 1.0000
N6 N 8 0.33956 0.45865 0.15736 1.0000
N7 N 8 0.41307 0.55342 0.19290 1.0000
N8 N 8 0.40171 0.50388 0.13941 1.0000
N9 N 8 0.35831 0.53911 0.24352 1.0000
N10 N 8 0.31264 0.47997 0.22146 1.0000
N11 N 8 0.36522 0.25030 0.13674 1.0000
N12 N 8 0.43263 0.18281 0.20934 1.0000
N13 N 8 0.40905 0.25716 0.19419 1.0000
N14 N 8 0.40340 0.13028 0.16171 1.0000
N15 N 8 0.36148 0.17236 0.11652 1.0000
N16 N 8 0.32914 0.37125 0.97958 1.0000
N17 N 8 0.39715 0.36986 0.88685 1.0000
N18 N 8 0.40191 0.35588 0.95489 1.0000
N19 N 8 0.32211 0.39350 0.86946 1.0000
N20 N 8 0.27975 0.39443 0.92723 1.0000
Cd1 Cd 8 0.29518 0.35552 0.09166 1.0000
Cd2 Cd 8 0.99596 0.15249 0.19095 1.0000
#End

data_23-lon_Cd
_audit_creation_method ToposPro
_Chemical_Name_Systematic SIVGEL
_cell_length_a 16.78992
_cell_length_b 10.35043
_cell_length_c 10.35043
_cell_angle_alpha 57.86929
_cell_angle_beta 90
_cell_angle_gamma 90
_cell_volume 1523.229
_cell_formula_units_Z 4
_symmetry_space_group_name_H-M 'P 1'
_symmetry_Int_Tables_number 1
loop_
_symmetry_equiv_pos_site_id
_symmetry_equiv_pos_as_xyz
1 x,y,z
loop_
_atom_site_label
_atom_site_type_symbol
_atom_site_symmetry_multiplicity
_atom_site_fract_x
_atom_site_fract_y
_atom_site_fract_z
_atom_site_occupancy
N1 N 1 0.94629 0.59502 0.91773 1.0000
N2 N 1 0.05371 0.08227 0.40498 1.0000
N3 N 1 0.55371 0.59502 0.91773 1.0000
N4 N 1 0.44629 0.08227 0.40498 1.0000
N5 N 1 0.18823 0.76655 0.55425 1.0000
N6 N 1 0.81177 0.44575 0.23345 1.0000
N7 N 1 0.31177 0.76655 0.55425 1.0000
N8 N 1 0.68823 0.44575 0.23345 1.0000

N8 N 1 0.75685 0.36990 0.36990 1.0000
N9 N 1 0.38695 0.63010 0.00000 1.0000
N10 N 1 0.36990 0.75685 0.36990 1.0000
N11 N 1 0.63010 0.38695 0.00000 1.0000
N12 N 1 0.38695 0.00000 0.63010 1.0000
N13 N 1 0.79113 0.31853 0.73611 1.0000
N14 N 1 0.41757 0.47260 0.68147 1.0000
N15 N 1 0.05503 0.58243 0.26389 1.0000
N16 N 1 0.94497 0.20887 0.52740 1.0000
N17 N 1 0.73611 0.31853 0.79113 1.0000
N18 N 1 0.79113 0.73611 0.31853 1.0000
N19 N 1 0.31853 0.73611 0.79113 1.0000
N20 N 1 0.94497 0.52740 0.20887 1.0000
N21 N 1 0.52740 0.94497 0.20887 1.0000
N22 N 1 0.68147 0.41757 0.47260 1.0000
N23 N 1 0.20887 0.52740 0.94497 1.0000
N24 N 1 0.47260 0.41757 0.68147 1.0000
N25 N 1 0.05503 0.26389 0.58243 1.0000
N26 N 1 0.58243 0.05503 0.26389 1.0000
N27 N 1 0.31853 0.79113 0.73611 1.0000
N28 N 1 0.47260 0.68147 0.41757 1.0000
N29 N 1 0.73611 0.79113 0.31853 1.0000
N30 N 1 0.58243 0.26389 0.05503 1.0000
N31 N 1 0.41757 0.68147 0.47260 1.0000
N32 N 1 0.26389 0.58243 0.05503 1.0000
N33 N 1 0.68147 0.47260 0.41757 1.0000
N34 N 1 0.26389 0.05503 0.58243 1.0000
N35 N 1 0.20887 0.94497 0.52740 1.0000
N36 N 1 0.52740 0.20887 0.94497 1.0000
N37 N 1 0.71499 0.27209 0.62650 1.0000
N38 N 1 0.35441 0.44290 0.72791 1.0000
N39 N 1 0.08849 0.64559 0.37350 1.0000
N40 N 1 0.91151 0.28501 0.55710 1.0000
N41 N 1 0.62650 0.27209 0.71499 1.0000
N42 N 1 0.71499 0.62650 0.27209 1.0000
N43 N 1 0.27209 0.62650 0.71499 1.0000
N44 N 1 0.91151 0.55710 0.28501 1.0000
N45 N 1 0.55710 0.91151 0.28501 1.0000
N46 N 1 0.72791 0.35441 0.44290 1.0000
N47 N 1 0.28501 0.55710 0.91151 1.0000
N48 N 1 0.44290 0.35441 0.72791 1.0000
N49 N 1 0.08849 0.37350 0.64559 1.0000
N50 N 1 0.64559 0.08849 0.37350 1.0000
N51 N 1 0.27209 0.71499 0.62650 1.0000
N52 N 1 0.44290 0.72791 0.35441 1.0000
N53 N 1 0.62650 0.71499 0.27209 1.0000
N54 N 1 0.64559 0.37350 0.08849 1.0000
N55 N 1 0.35441 0.72791 0.44290 1.0000
N56 N 1 0.37350 0.64559 0.08849 1.0000
N57 N 1 0.72791 0.44290 0.35441 1.0000
N58 N 1 0.37350 0.08849 0.64559 1.0000
N59 N 1 0.28501 0.91151 0.55710 1.0000
N60 N 1 0.55710 0.28501 0.91151 1.0000
Zn1 Zn 1 0.75000 0.25000 0.50000 1.0000
Zn2 Zn 1 0.25000 0.50000 0.75000 1.0000
Zn3 Zn 1 0.25000 0.75000 0.50000 1.0000
Zn4 Zn 1 0.50000 0.25000 0.75000 1.0000
Zn5 Zn 1 0.75000 0.50000 0.25000 1.0000
Zn6 Zn 1 0.50000 0.75000 0.25000 1.0000
#End

data_24-hcb_Zn
_audit_creation_method ToposPro
_Chemical_Name_Systematic CAYSEB
_cell_length_a 7.388536
_cell_length_b 9.818039
_cell_length_c 13.3632
_cell_angle_alpha 90
_cell_angle_beta 116.1294
_cell_angle_gamma 90
_cell_volume 870.3106
_cell_formula_units_Z 4
_symmetry_space_group_name_H-M 'P 21/c'
_symmetry_Int_Tables_number 14
loop_
_symmetry_equiv_pos_site_id
_symmetry_equiv_pos_as_xyz
1 x,y,z
2 -x,1/2+y,1/2-z
3 -x,-y,-z
4 x,1/2-y,1/2+z
loop_
_atom_site_label
_atom_site_type_symbol
_atom_site_symmetry_multiplicity
_atom_site_fract_x
_atom_site_fract_y
_atom_site_fract_z
_atom_site_occupancy
N1 N 4 0.47116 0.93360 0.24267 1.0000
N2 N 4 0.27223 0.06384 0.98428 1.0000
N3 N 4 0.48530 0.80218 0.23437 1.0000
N4 N 4 0.42676 0.85073 0.37533 1.0000
N5 N 4 0.43494 0.96409 0.32935 1.0000
N6 N 4 0.10034 0.12193 0.96296 1.0000
N7 N 4 0.26708 0.02278 0.88870 1.0000
N8 N 4 0.98940 0.11673 0.85460 1.0000
N9 N 4 0.09204 0.05564 0.80854 1.0000
N10 N 4 0.45752 0.75131 0.31636 1.0000
Zn1 Zn 4 0.50756 0.05787 0.13698 1.0000
#End

N9 N 1 0.02071 0.79546 0.31240 1.0000
N10 N 1 0.97929 0.68760 0.20454 1.0000
N11 N 1 0.47929 0.79546 0.31240 1.0000
N12 N 1 0.52071 0.68760 0.20454 1.0000
N13 N 1 0.21155 0.63836 0.68024 1.0000
N14 N 1 0.78845 0.31976 0.36164 1.0000
N15 N 1 0.28845 0.63836 0.68024 1.0000
N16 N 1 0.71155 0.31976 0.36164 1.0000
N17 N 1 0.98755 0.70171 0.69513 1.0000
N18 N 1 0.01245 0.30487 0.29829 1.0000
N19 N 1 0.51245 0.70171 0.69513 1.0000
N20 N 1 0.48755 0.30487 0.29829 1.0000
N21 N 1 0.91909 0.63392 0.70077 1.0000
N22 N 1 0.08091 0.29923 0.36608 1.0000
N23 N 1 0.58091 0.63392 0.70077 1.0000
N24 N 1 0.41909 0.29923 0.36608 1.0000
N25 N 1 0.25000 0.84634 0.47580 1.0000
N26 N 1 0.75000 0.52420 0.15366 1.0000
N27 N 1 0.00467 0.67805 0.82903 1.0000
N28 N 1 0.99533 0.17097 0.32195 1.0000
N29 N 1 0.49533 0.67805 0.82903 1.0000
N30 N 1 0.50467 0.17097 0.32195 1.0000
N31 N 1 0.89345 0.56737 0.83940 1.0000
N32 N 1 0.10655 0.16060 0.43263 1.0000
N33 N 1 0.60655 0.56737 0.83940 1.0000
N34 N 1 0.39345 0.16060 0.43263 1.0000
N35 N 1 0.01291 0.90224 0.16491 1.0000
N36 N 1 0.98709 0.83509 0.09776 1.0000
N37 N 1 0.48709 0.90224 0.16491 1.0000
N38 N 1 0.51291 0.83509 0.09776 1.0000
N39 N 1 0.00000 0.66241 0.33759 1.0000
N40 N 1 0.50000 0.66241 0.33759 1.0000
Cd1 Cd 1 0.06272 0.83657 0.48995 1.0000
Cd2 Cd 1 0.93728 0.51005 0.16343 1.0000
Cd3 Cd 1 0.43728 0.83657 0.48995 1.0000
Cd4 Cd 1 0.56272 0.51005 0.16343 1.0000
#End

data_24-dia2_Cd
_audit_creation_method ToposPro
_Chemical_Name_Systematic KEYSEO
_cell_length_a 10.41428
_cell_length_b 10.41428
_cell_length_c 10.78697
_cell_angle_alpha 90
_cell_angle_beta 90
_cell_angle_gamma 90
_cell_volume 1169.924
_cell_formula_units_Z 4
_symmetry_space_group_name_H-M 'P 43 21 2'
_symmetry_Int_Tables_number 96
loop_
_symmetry_equiv_pos_site_id
_symmetry_equiv_pos_as_xyz
1 x,y,z
2 -x,-y,1/2+z
3 1/2+x,1/2-y,1/4-z
4 1/2-x,1/2+y,3/4-z
5 -y,-x,1/2-z
6 y,x,-z
7 1/2+y,1/2-x,1/4+z
8 1/2-y,1/2+x,3/4+z
loop_
_atom_site_label
_atom_site_type_symbol
_atom_site_symmetry_multiplicity
_atom_site_fract_x
_atom_site_fract_y
_atom_site_fract_z
_atom_site_occupancy
N1 N 8 0.26612 0.77912 0.39819 1.0000
N2 N 8 0.20208 0.74809 0.50062 1.0000
N3 N 8 0.19499 0.94853 0.47258 1.0000
N4 N 8 0.15788 0.85355 0.54709 1.0000
N5 N 8 0.26196 0.90297 0.38026 1.0000
Cd1 Cd 4 0.34645 0.65355 0.25000 1.0000
#End

data_25-bik_Cd
_audit_creation_method ToposPro
_Chemical_Name_Systematic YOMBOS
_cell_length_a 14.96482
_cell_length_b 10.27635
_cell_length_c 17.41996
_cell_angle_alpha 90
_cell_angle_beta 117.0328
_cell_angle_gamma 90
_cell_volume 2386.226
_cell_formula_units_Z 6
_symmetry_space_group_name_H-M 'P 21'
_symmetry_Int_Tables_number 4
loop_
_symmetry_equiv_pos_site_id
_symmetry_equiv_pos_as_xyz
1 x,y,z
2 -x,1/2+y,-z
loop_
_atom_site_label
_atom_site_type_symbol
_atom_site_symmetry_multiplicity
_atom_site_fract_x

data_25-can_Zn
_audit_creation_method TopostPro
_Chemical_Name_Systematic PAJRUQ
_cell_length_a 9.600691
_cell_length_b 23.04108
_cell_length_c 41.19614
_cell_angle_alpha 90
_cell_angle_beta 90
_cell_angle_gamma 90
_cell_volume 9113.009
_cell_formula_units_Z 24
_symmetry_space_group_name_H-M 'P n m a'
_symmetry_Int_Tables_number 62
loop_
_symmetry_equiv_pos_site_id
_symmetry_equiv_pos_as_xyz
1 x,y,z
2 1/2-x,-y,1/2+z
3 1/2+x,1/2-y,1/2-z
4 -x,1/2+y,-z
5 -x,-y,-z
6 1/2+x,y,1/2-z
7 1/2-x,1/2+y,1/2+z
8 x,1/2-y,z
loop_
_atom_site_label
_atom_site_type_symbol
_atom_site_symmetry_multiplicity
_atom_site_fract_x
_atom_site_fract_y
_atom_site_fract_z
_atom_site_occupancy
N1 N 8 0.75162 0.43973 0.94821 1.0000
N2 N 8 0.71743 0.39630 0.99158 1.0000
N3 N 8 0.75322 0.47641 0.87126 1.0000
N4 N 8 0.75241 0.43212 0.82757 1.0000
N5 N 8 0.66499 0.56697 0.92782 1.0000
N6 N 8 0.48773 0.60923 0.94414 1.0000
N7 N 8 0.83201 0.42879 0.06366 1.0000
N8 N 8 0.00914 0.47103 0.07997 1.0000
N9 N 8 0.66008 0.39267 0.75543 1.0000
N10 N 8 0.48463 0.39306 0.72613 1.0000
N11 N 8 0.75275 0.29491 0.04772 1.0000
N12 N 8 0.77719 0.29500 0.80612 1.0000
N13 N 8 0.76653 0.44459 0.97957 1.0000
N14 N 8 0.67224 0.36184 0.96779 1.0000
N15 N 8 0.69347 0.38883 0.94085 1.0000
N16 N 8 0.81149 0.43318 0.85603 1.0000
N17 N 8 0.65808 0.47449 0.82517 1.0000
N18 N 8 0.65856 0.50202 0.85233 1.0000
N19 N 8 0.54701 0.55840 0.94278 1.0000
N20 N 8 0.56847 0.64907 0.93011 1.0000
N21 N 8 0.67871 0.62276 0.91999 1.0000
N22 N 8 0.96272 0.43774 0.05675 1.0000
N23 N 8 0.90772 0.48256 0.10112 1.0000
N24 N 8 0.79760 0.45629 0.09099 1.0000
N25 N 8 0.53852 0.36832 0.75170 1.0000
N26 N 8 0.57228 0.43250 0.71415 1.0000
N27 N 8 0.68135 0.43223 0.73237 1.0000
N28 N 4 0.69410 0.25000 0.03421 1.0000
N29 N 8 0.84688 0.27794 0.06958 1.0000
N30 N 4 0.80590 0.25000 0.78810 1.0000
N31 N 8 0.73080 0.27796 0.83511 1.0000
Zn1 Zn 8 0.79432 0.50256 0.91598 1.0000
Zn2 Zn 8 0.70456 0.37730 0.03869 1.0000
Zn3 Zn 8 0.79431 0.37746 0.79154 1.0000
#End

data_26-dft_Zn
_audit_creation_method TopostPro
_Chemical_Name_Systematic VEJYOZ
_cell_length_a 18.74843
_cell_length_b 18.74843
_cell_length_c 16.08804
_cell_angle_alpha 90
_cell_angle_beta 90
_cell_angle_gamma 90
_cell_volume 5655.006
_cell_formula_units_Z 16
_symmetry_space_group_name_H-M 'P 42/m n m'
_symmetry_Int_Tables_number 136
loop_
_symmetry_equiv_pos_site_id
_symmetry_equiv_pos_as_xyz
1 x,y,z
2 -x,-y,z
3 1/2+x,1/2-y,1/2-z
4 1/2-x,1/2+y,1/2-z
5 -y,-x,-z
6 y,x,-z
7 1/2+y,1/2-x,1/2+z
8 1/2-y,1/2+x,1/2+z
9 -x,-y,-z
10 x,y,-z
11 1/2-x,1/2+y,1/2+z
12 1/2+x,1/2-y,1/2+z
13 y,x,z
14 -y,-x,z
15 1/2-y,1/2+x,1/2-z
16 1/2+y,1/2-x,1/2-z
_atom_site_fract_y
_atom_site_fract_z
_atom_site_occupancy
N1 N 2 0.71842 0.11362 0.32525 1.0000
N2 N 2 0.62763 0.13764 0.26825 1.0000
N3 N 2 0.36072 0.38445 0.18893 1.0000
N4 N 2 0.27942 0.34844 0.19142 1.0000
N5 N 2 0.88533 0.28766 0.00059 1.0000
N6 N 2 0.39150 0.18750 0.91392 1.0000
N7 N 2 0.12410 0.09303 0.36119 1.0000
N8 N 2 0.15654 0.08143 0.30436 1.0000
N9 N 2 0.39149 0.08382 0.87201 1.0000
N10 N 2 0.84540 0.39161 0.95663 1.0000
N11 N 2 0.98916 0.10973 0.57671 1.0000
N12 N 2 0.01207 0.00487 0.62258 1.0000
N13 N 2 0.08601 0.12970 0.23081 1.0000
N14 N 2 0.00996 0.17139 0.24142 1.0000
N15 N 2 0.03386 0.14832 0.32210 1.0000
N16 N 2 0.98429 0.07842 0.50076 1.0000
N17 N 2 0.00379 0.95506 0.49889 1.0000
N18 N 2 0.02095 0.91011 0.57434 1.0000
N19 N 2 0.77862 0.18838 0.30655 1.0000
N20 N 2 0.72596 0.25886 0.23844 1.0000
N21 N 2 0.63275 0.22708 0.21506 1.0000
N22 N 2 0.46375 0.17330 0.99424 1.0000
N23 N 2 0.50869 0.06151 0.00272 1.0000
N24 N 2 0.46374 0.00661 0.92695 1.0000
N25 N 2 0.37280 0.30645 0.13334 1.0000
N26 N 2 0.29978 0.22203 0.10126 1.0000
N27 N 2 0.24237 0.24841 0.13746 1.0000
N28 N 2 0.98387 0.29604 0.02763 1.0000
N29 N 2 0.00567 0.40452 0.00085 1.0000
N30 N 2 0.91979 0.46325 0.95700 1.0000
Cd1 Cd 2 0.94246 0.20453 0.38740 1.0000
Cd2 Cd 2 0.50133 0.31023 0.10343 1.0000
Cd3 Cd 2 0.09893 0.15170 0.11002 1.0000
#End

data_26-dia3_Cd
_audit_creation_method TopostPro
_Chemical_Name_Systematic SIVGOV
_cell_length_a 10.19018
_cell_length_b 10.19018
_cell_length_c 10.25066
_cell_angle_alpha 119.831
_cell_angle_beta 119.831
_cell_angle_gamma 88.58805
_cell_volume 765.1194
_cell_formula_units_Z 2
_symmetry_space_group_name_H-M 'P 1'
_symmetry_Int_Tables_number 1
loop_
_symmetry_equiv_pos_site_id
_symmetry_equiv_pos_as_xyz
1 x,y,z
loop_
_atom_site_label
_atom_site_type_symbol
_atom_site_symmetry_multiplicity
_atom_site_fract_x
_atom_site_fract_y
_atom_site_fract_z
_atom_site_occupancy
N1 N 1 0.59574 0.09966 0.00912 1.0000
N2 N 1 0.09966 0.59574 0.50912 1.0000
N3 N 1 0.68625 0.34445 0.18230 1.0000
N4 N 1 0.34445 0.68625 0.68230 1.0000
N5 N 1 0.89224 0.77855 0.88920 1.0000
N6 N 1 0.77855 0.89224 0.38920 1.0000
N7 N 1 0.82860 0.68927 0.62388 1.0000
N8 N 1 0.68927 0.82860 0.12388 1.0000
N9 N 1 0.94455 0.72368 0.78632 1.0000
N10 N 1 0.72368 0.94455 0.28632 1.0000
N11 N 1 0.48097 0.15466 0.02481 1.0000
N12 N 1 0.15466 0.48097 0.52481 1.0000
N13 N 1 0.72323 0.21660 0.10674 1.0000
N14 N 1 0.21660 0.72323 0.60674 1.0000
N15 N 1 0.70449 0.72225 0.62478 1.0000
N16 N 1 0.72225 0.70449 0.12478 1.0000
N17 N 1 0.74446 0.77750 0.78914 1.0000
N18 N 1 0.77750 0.74446 0.28914 1.0000
N19 N 1 0.53715 0.30694 0.13235 1.0000
N20 N 1 0.30694 0.53715 0.63235 1.0000
Cd1 Cd 1 0.84784 0.58845 0.38684 1.0000
Cd2 Cd 1 0.58845 0.84784 0.88684 1.0000
#End

data_27-dia4_Cd
_audit_creation_method TopostPro
_Chemical_Name_Systematic HOKMUR
_cell_length_a 20.58474
_cell_length_b 13.9243
_cell_length_c 10.6909
_cell_angle_alpha 90
_cell_angle_beta 90.83234
_cell_angle_gamma 90
_cell_volume 3063.99
_cell_formula_units_Z 8
_symmetry_space_group_name_H-M 'P c'
_symmetry_Int_Tables_number 7
loop_

loop_
_atom_site_label
_atom_site_type_symbol
_atom_site_symmetry_multiplicity
_atom_site_fract_x
_atom_site_fract_y
_atom_site_fract_z
_atom_site_occupancy
N1 N 8 0.71187 0.28813 0.16807 1.0000
N2 N 16 0.64007 0.31135 0.26988 1.0000
N3 N 16 0.63444 0.03221 0.24478 1.0000
N4 N 16 0.69984 0.11017 0.30821 1.0000
N5 N 16 0.68128 0.10975 0.04007 1.0000
N6 N 8 0.59495 0.17815 0.00000 1.0000
N7 N 16 0.71726 0.04835 0.33670 1.0000
N8 N 16 0.65455 0.26733 0.20710 1.0000
N9 N 16 0.64883 0.09989 0.25171 1.0000
N10 N 16 0.62796 0.15195 0.06448 1.0000
N11 N 16 0.67685 0.00049 0.29745 1.0000
Zn1 Zn 16 0.60388 0.17639 0.18277 1.0000
#End

data_27-dia2_Zn
_audit_creation_method TopoSPro
_Chemical_Name_Systematic HOKMUR
_cell_length_a 19.04994
_cell_length_b 13.1654
_cell_length_c 9.962445
_cell_angle_alpha 90
_cell_angle_beta 91.90868
_cell_angle_gamma 90
_cell_volume 2497.196
_cell_formula_units_Z 8
_symmetry_space_group_name_H-M 'P c'
_symmetry_Int_Tables_number 7
loop_
_symmetry_equiv_pos_site_id
_symmetry_equiv_pos_as_xyz
1 x,y,z
2 x,-y,1/2+z
loop_
_atom_site_label
_atom_site_type_symbol
_atom_site_symmetry_multiplicity
_atom_site_fract_x
_atom_site_fract_y
_atom_site_fract_z
_atom_site_occupancy
N1 N 2 0.46404 0.69617 0.45132 1.0000
N2 N 2 0.41065 0.63683 0.45983 1.0000
N3 N 2 0.45375 0.77396 0.53399 1.0000
N4 N 2 0.36797 0.67846 0.54784 1.0000
N5 N 2 0.71895 0.80776 0.45446 1.0000
N6 N 2 0.66422 0.86509 0.45603 1.0000
N7 N 2 0.70617 0.72755 0.53161 1.0000
N8 N 2 0.61804 0.81990 0.53414 1.0000
N9 N 2 0.97695 0.68977 0.48933 1.0000
N10 N 2 0.92287 0.63119 0.48041 1.0000
N11 N 2 0.87144 0.67697 0.54486 1.0000
N12 N 2 0.95840 0.77128 0.55926 1.0000
N13 N 2 0.21850 0.80820 0.48511 1.0000
N14 N 2 0.12017 0.82542 0.57368 1.0000
N15 N 2 0.16535 0.86819 0.49137 1.0000
N16 N 2 0.20577 0.72868 0.56361 1.0000
N17 N 2 0.76132 0.59695 0.97192 1.0000
N18 N 2 0.76212 0.64608 0.86031 1.0000
N19 N 2 0.76806 0.57764 0.76239 1.0000
N20 N 2 0.76675 0.49853 0.94210 1.0000
N21 N 2 0.01548 0.00066 0.97868 1.0000
N22 N 2 0.26994 0.49851 0.96564 1.0000
N23 N 2 0.27527 0.57645 0.78577 1.0000
N24 N 2 0.54580 0.90825 0.97098 1.0000
N25 N 2 0.54776 0.85966 0.85912 1.0000
N26 N 2 0.52383 0.92253 0.76286 1.0000
N27 N 2 0.52054 0.00059 0.94303 1.0000
N28 N 2 0.39438 0.76326 0.59397 1.0000
N29 N 2 0.64375 0.73481 0.58113 1.0000
N30 N 2 0.14499 0.73911 0.61870 1.0000
N31 N 2 0.77096 0.48629 0.81262 1.0000
N32 N 2 0.89323 0.76365 0.59387 1.0000
N33 N 2 0.50701 0.00982 0.81433 1.0000
N34 N 2 0.01625 0.92278 0.79810 1.0000
N35 N 2 0.26046 0.48794 0.83608 1.0000
N36 N 2 0.00957 0.85380 0.89512 1.0000
N37 N 2 0.01996 0.01375 0.84944 1.0000
N38 N 2 0.29075 0.59282 0.99527 1.0000
N39 N 2 0.00909 0.90221 0.00732 1.0000
N40 N 2 0.29391 0.64138 0.88355 1.0000
Zn1 Zn 2 0.77610 0.61686 0.56964 1.0000
Zn2 Zn 2 0.02434 0.88386 0.60654 1.0000
Zn3 Zn 2 0.52394 0.88316 0.56968 1.0000
Zn4 Zn 2 0.27574 0.61691 0.59387 1.0000
#End

data_28-bik_Zn
_audit_creation_method TopoSPro
_Chemical_Name_Systematic YOMBOS
_cell_length_a 13.95479
_cell_length_b 9.581575
_cell_length_c 16.29477
_cell_angle_alpha 90
_symmetry_equiv_pos_site_id
_symmetry_equiv_pos_as_xyz
1 x,y,z
2 x,-y,1/2+z
loop_
_atom_site_label
_atom_site_type_symbol
_atom_site_symmetry_multiplicity
_atom_site_fract_x
_atom_site_fract_y
_atom_site_fract_z
_atom_site_occupancy
N1 N 2 0.46143 0.69576 0.45503 1.0000
N2 N 2 0.41092 0.64110 0.46144 1.0000
N3 N 2 0.45241 0.76819 0.53414 1.0000
N4 N 2 0.37130 0.68028 0.54456 1.0000
N5 N 2 0.71591 0.80828 0.45810 1.0000
N6 N 2 0.66469 0.86177 0.45883 1.0000
N7 N 2 0.70374 0.73249 0.52986 1.0000
N8 N 2 0.62143 0.81841 0.53106 1.0000
N9 N 2 0.97258 0.68852 0.49196 1.0000
N10 N 2 0.92158 0.63481 0.48285 1.0000
N11 N 2 0.87430 0.67881 0.54432 1.0000
N12 N 2 0.95622 0.76514 0.55904 1.0000
N13 N 2 0.21509 0.80933 0.48858 1.0000
N14 N 2 0.12289 0.82370 0.57021 1.0000
N15 N 2 0.16480 0.86452 0.49282 1.0000
N16 N 2 0.20368 0.73493 0.56329 1.0000
N17 N 2 0.76218 0.59539 0.96536 1.0000
N18 N 2 0.76212 0.64215 0.86114 1.0000
N19 N 2 0.76761 0.57775 0.76968 1.0000
N20 N 2 0.76773 0.50262 0.93717 1.0000
N21 N 2 0.01570 0.99641 0.97317 1.0000
N22 N 2 0.27148 0.50334 0.96170 1.0000
N23 N 2 0.27421 0.57737 0.79355 1.0000
N24 N 2 0.54536 0.90898 0.96399 1.0000
N25 N 2 0.54598 0.86247 0.85966 1.0000
N26 N 2 0.52388 0.92201 0.77112 1.0000
N27 N 2 0.52275 0.99659 0.93899 1.0000
N28 N 2 0.39672 0.75895 0.58983 1.0000
N29 N 2 0.64532 0.73838 0.57537 1.0000
N30 N 2 0.14665 0.74350 0.61417 1.0000
N31 N 2 0.77115 0.49129 0.81617 1.0000
N32 N 2 0.89548 0.75948 0.59174 1.0000
N33 N 2 0.50955 0.00517 0.81965 1.0000
N34 N 2 0.01564 0.92170 0.80533 1.0000
N35 N 2 0.26143 0.49348 0.84150 1.0000
N36 N 2 0.00919 0.85715 0.89641 1.0000
N37 N 2 0.01978 0.00795 0.85232 1.0000
N38 N 2 0.29061 0.59255 0.98785 1.0000
N39 N 2 0.00923 0.90368 0.00083 1.0000
N40 N 2 0.29221 0.63869 0.88332 1.0000
Cd1 Cd 2 0.77527 0.61798 0.57046 1.0000
Cd2 Cd 2 0.02456 0.88275 0.60649 1.0000
Cd3 Cd 2 0.52470 0.88182 0.57152 1.0000
Cd4 Cd 2 0.27506 0.61834 0.59426 1.0000
#End

data_28-sod_Cd
_audit_creation_method TopoSPro
_Chemical_Name_Systematic OFERUN03
_cell_length_a 15.34759
_cell_length_b 15.34759
_cell_length_c 15.34759
_cell_angle_alpha 109.4712
_cell_angle_beta 109.4712
_cell_angle_gamma 109.4712
_cell_volume 2782.906
_cell_formula_units_Z 6
_symmetry_space_group_name_H-M 'P 1'
_symmetry_Int_Tables_number 1
loop_
_symmetry_equiv_pos_site_id
_symmetry_equiv_pos_as_xyz
1 x,y,z
loop_
_atom_site_label
_atom_site_type_symbol
_atom_site_symmetry_multiplicity
_atom_site_fract_x
_atom_site_fract_y
_atom_site_fract_z
_atom_site_occupancy
N1 N 1 0.61918 0.24614 0.61918 1.0000
N2 N 1 0.37303 0.37303 0.75386 1.0000
N3 N 1 0.00000 0.62697 0.38082 1.0000
N4 N 1 0.00000 0.38082 0.62697 1.0000
N5 N 1 0.61918 0.61918 0.24614 1.0000
N6 N 1 0.24614 0.61918 0.61918 1.0000
N7 N 1 0.62697 0.00000 0.38082 1.0000
N8 N 1 0.75386 0.37303 0.37303 1.0000
N9 N 1 0.38082 0.62697 0.00000 1.0000
N10 N 1 0.37303 0.75386 0.37303 1.0000
N11 N 1 0.62697 0.38082 0.00000 1.0000
N12 N 1 0.38082 0.00000 0.62697 1.0000
N13 N 1 0.78549 0.31661 0.73397 1.0000
N14 N 1 0.41737 0.46888 0.68339 1.0000
N15 N 1 0.05152 0.58263 0.26603 1.0000
N16 N 1 0.94848 0.21451 0.53112 1.0000
N17 N 1 0.73397 0.31661 0.78549 1.0000
N18 N 1 0.78549 0.73397 0.31661 1.0000

_cell_angle_beta 116.7461
_cell_angle_gamma 90
_cell_volume 1945.649
_cell_formula_units_Z 6
_symmetry_space_group_name_H-M 'P 21'
_symmetry_Int_Tables_number 4
loop_
_symmetry_equiv_pos_site_id
_symmetry_equiv_pos_as_xyz
1 x,y,z
2 -x,1/2+y,-z
loop_
_atom_site_label
_atom_site_type_symbol
_atom_site_symmetry_multiplicity
_atom_site_fract_x
_atom_site_fract_y
_atom_site_fract_z
_atom_site_occupancy
N1 N 2 0.71928 0.10865 0.32874 1.0000
N2 N 2 0.62264 0.13473 0.26817 1.0000
N3 N 2 0.36380 0.39083 0.19155 1.0000
N4 N 2 0.27704 0.35188 0.19443 1.0000
N5 N 2 0.88228 0.28257 0.00177 1.0000
N6 N 2 0.38832 0.19326 0.91269 1.0000
N7 N 2 0.12700 0.09095 0.36574 1.0000
N8 N 2 0.16236 0.07975 0.30538 1.0000
N9 N 2 0.38746 0.08236 0.86766 1.0000
N10 N 2 0.83988 0.39331 0.95446 1.0000
N11 N 2 0.98844 0.11646 0.57762 1.0000
N12 N 2 0.01360 0.00440 0.62652 1.0000
N13 N 2 0.08677 0.13108 0.22678 1.0000
N14 N 2 0.00474 0.17416 0.23797 1.0000
N15 N 2 0.02987 0.14904 0.32386 1.0000
N16 N 2 0.98265 0.08283 0.49609 1.0000
N17 N 2 0.00391 0.95083 0.49400 1.0000
N18 N 2 0.02301 0.90272 0.57465 1.0000
N19 N 2 0.78422 0.18841 0.30861 1.0000
N20 N 2 0.72851 0.26390 0.23601 1.0000
N21 N 2 0.62877 0.23046 0.21129 1.0000
N22 N 2 0.46639 0.17764 0.99799 1.0000
N23 N 2 0.51395 0.05762 0.00625 1.0000
N24 N 2 0.46494 0.99901 0.92556 1.0000
N25 N 2 0.37750 0.30670 0.13250 1.0000
N26 N 2 0.29987 0.21566 0.09870 1.0000
N27 N 2 0.23809 0.24381 0.13724 1.0000
N28 N 2 0.98797 0.29205 0.03128 1.0000
N29 N 2 0.01159 0.40819 0.00261 1.0000
N30 N 2 0.91981 0.47055 0.95515 1.0000
Zn1 Zn 2 0.94192 0.20467 0.38687 1.0000
Zn2 Zn 2 0.50217 0.30870 0.10384 1.0000
Zn3 Zn 2 0.09886 0.15255 0.11018 1.0000
#End

data_29-mer_Zn
_audit_creation_method ToposPro
_Chemical_Name_Systematic VEJZIU
_cell_length_a 26.47993
_cell_length_b 26.47993
_cell_length_c 20.99502
_cell_angle_alpha 50.90382
_cell_angle_beta 50.90382
_cell_angle_gamma 90
_cell_volume 6659.332
_cell_formula_units_Z 16
_symmetry_space_group_name_H-M 'P 1'
_symmetry_Int_Tables_number 1
loop_
_symmetry_equiv_pos_site_id
_symmetry_equiv_pos_as_xyz
1 x,y,z
loop_
_atom_site_label
_atom_site_type_symbol
_atom_site_symmetry_multiplicity
_atom_site_fract_x
_atom_site_fract_y
_atom_site_fract_z
_atom_site_occupancy
N1 N 1 0.13863 0.87571 0.45844 1.0000
N2 N 1 0.33415 0.59707 0.54156 1.0000
N3 N 1 0.59707 0.12429 0.54156 1.0000
N4 N 1 0.66585 0.13863 0.45844 1.0000
N5 N 1 0.59707 0.33415 0.54156 1.0000
N6 N 1 0.86137 0.33415 0.54156 1.0000
N7 N 1 0.87571 0.13863 0.45844 1.0000
N8 N 1 0.12429 0.86137 0.54156 1.0000
N9 N 1 0.13863 0.66585 0.45844 1.0000
N10 N 1 0.87571 0.40293 0.45844 1.0000
N11 N 1 0.40293 0.87571 0.45844 1.0000
N12 N 1 0.40293 0.66585 0.45844 1.0000
N13 N 1 0.66585 0.40293 0.45844 1.0000
N14 N 1 0.86137 0.12429 0.54156 1.0000
N15 N 1 0.12429 0.59707 0.54156 1.0000
N16 N 1 0.33415 0.86137 0.54156 1.0000
N17 N 1 0.07631 0.92369 0.50000 1.0000
N18 N 1 0.42369 0.57631 0.50000 1.0000
N19 N 1 0.57631 0.07631 0.50000 1.0000
N20 N 1 0.57631 0.42369 0.50000 1.0000
N21 N 1 0.92369 0.42369 0.50000 1.0000
N22 N 1 0.92369 0.07631 0.50000 1.0000
N19 N 1 0.31661 0.73397 0.78549 1.0000
N20 N 1 0.94848 0.53112 0.21451 1.0000
N21 N 1 0.53112 0.94848 0.21451 1.0000
N22 N 1 0.68339 0.41737 0.46888 1.0000
N23 N 1 0.21451 0.53112 0.94848 1.0000
N24 N 1 0.46888 0.41737 0.68339 1.0000
N25 N 1 0.05152 0.26603 0.58263 1.0000
N26 N 1 0.58263 0.05152 0.26603 1.0000
N27 N 1 0.31661 0.78549 0.73397 1.0000
N28 N 1 0.46888 0.68339 0.41737 1.0000
N29 N 1 0.73397 0.78549 0.31661 1.0000
N30 N 1 0.58263 0.26603 0.05152 1.0000
N31 N 1 0.41737 0.68339 0.46888 1.0000
N32 N 1 0.26603 0.58263 0.05152 1.0000
N33 N 1 0.68339 0.46888 0.41737 1.0000
N34 N 1 0.26603 0.05152 0.58263 1.0000
N35 N 1 0.21451 0.94848 0.53112 1.0000
N36 N 1 0.53112 0.21451 0.94848 1.0000
N37 N 1 0.71445 0.27316 0.63169 1.0000
N38 N 1 0.35853 0.44129 0.72684 1.0000
N39 N 1 0.08276 0.64147 0.36831 1.0000
N40 N 1 0.91724 0.28555 0.55871 1.0000
N41 N 1 0.63169 0.27316 0.71445 1.0000
N42 N 1 0.71445 0.63169 0.27316 1.0000
N43 N 1 0.27316 0.63169 0.71445 1.0000
N44 N 1 0.91724 0.55871 0.28555 1.0000
N45 N 1 0.55871 0.91724 0.28555 1.0000
N46 N 1 0.72684 0.35853 0.44129 1.0000
N47 N 1 0.28555 0.55871 0.91724 1.0000
N48 N 1 0.44129 0.35853 0.72684 1.0000
N49 N 1 0.08276 0.36831 0.64147 1.0000
N50 N 1 0.64147 0.08276 0.36831 1.0000
N51 N 1 0.27316 0.71445 0.63169 1.0000
N52 N 1 0.44129 0.72684 0.35853 1.0000
N53 N 1 0.63169 0.71445 0.27316 1.0000
N54 N 1 0.64147 0.36831 0.08276 1.0000
N55 N 1 0.35853 0.72684 0.44129 1.0000
N56 N 1 0.36831 0.64147 0.08276 1.0000
N57 N 1 0.72684 0.44129 0.35853 1.0000
N58 N 1 0.36831 0.08276 0.64147 1.0000
N59 N 1 0.28555 0.91724 0.55871 1.0000
N60 N 1 0.55871 0.28555 0.91724 1.0000
Cd1 Cd 1 0.75000 0.25000 0.50000 1.0000
Cd2 Cd 1 0.25000 0.50000 0.75000 1.0000
Cd3 Cd 1 0.25000 0.75000 0.50000 1.0000
Cd4 Cd 1 0.50000 0.25000 0.75000 1.0000
Cd5 Cd 1 0.75000 0.50000 0.25000 1.0000
Cd6 Cd 1 0.50000 0.75000 0.25000 1.0000
#End

data_29-can_Cd
_audit_creation_method ToposPro
_Chemical_Name_Systematic PAJRUQ
_cell_length_a 10.34
_cell_length_b 24.6733
_cell_length_c 43.9206
_cell_angle_alpha 90
_cell_angle_beta 90
_cell_angle_gamma 90
_cell_volume 11205.11
_cell_formula_units_Z 24
_symmetry_space_group_name_H-M 'P n m a'
_symmetry_Int_Tables_number 62
loop_
_symmetry_equiv_pos_site_id
_symmetry_equiv_pos_as_xyz
1 x,y,z
2 1/2-x,-y,1/2+z
3 1/2+x,1/2-y,1/2-z
4 -x,1/2+y,-z
5 -x,-y,-z
6 1/2+x,y,1/2-z
7 1/2-x,1/2+y,1/2+z
8 x,1/2-y,z
loop_
_atom_site_label
_atom_site_type_symbol
_atom_site_symmetry_multiplicity
_atom_site_fract_x
_atom_site_fract_y
_atom_site_fract_z
_atom_site_occupancy
N1 N 8 0.74943 0.43778 0.94925 1.0000
N2 N 8 0.71638 0.39744 0.99000 1.0000
N3 N 8 0.75113 0.47588 0.86916 1.0000
N4 N 8 0.75002 0.43388 0.82832 1.0000
N5 N 8 0.66058 0.56911 0.92828 1.0000
N6 N 8 0.49490 0.60827 0.94334 1.0000
N7 N 8 0.83591 0.43060 0.06487 1.0000
N8 N 8 0.00161 0.46991 0.07979 1.0000
N9 N 8 0.65556 0.39351 0.75402 1.0000
N10 N 8 0.49188 0.39345 0.72672 1.0000
N11 N 8 0.75578 0.29202 0.04805 1.0000
N12 N 8 0.77636 0.29208 0.80641 1.0000
N13 N 8 0.76221 0.44251 0.97871 1.0000
N14 N 8 0.67537 0.36517 0.96770 1.0000
N15 N 8 0.69589 0.39028 0.94236 1.0000
N16 N 8 0.80588 0.43553 0.85492 1.0000
N17 N 8 0.66144 0.47291 0.82607 1.0000
N18 N 8 0.66207 0.49902 0.85151 1.0000
N19 N 8 0.55079 0.56088 0.94221 1.0000

N23 N 1 0.07631 0.57631 0.50000 1.0000
N24 N 1 0.42369 0.92369 0.50000 1.0000
N25 N 1 0.04446 0.04446 0.28691 1.0000
N26 N 1 0.33137 0.33137 0.71309 1.0000
N27 N 1 0.33137 0.95554 0.71309 1.0000
N28 N 1 0.66863 0.04446 0.28691 1.0000
N29 N 1 0.95554 0.33137 0.71309 1.0000
N30 N 1 0.95554 0.95554 0.71309 1.0000
N31 N 1 0.04446 0.66863 0.28691 1.0000
N32 N 1 0.66863 0.66863 0.28691 1.0000
N33 N 1 0.94281 0.90841 0.49795 1.0000
N34 N 1 0.40636 0.44076 0.50205 1.0000
N35 N 1 0.44076 0.09159 0.50205 1.0000
N36 N 1 0.59364 0.94281 0.49795 1.0000
N37 N 1 0.44076 0.40636 0.50205 1.0000
N38 N 1 0.05719 0.40636 0.50205 1.0000
N39 N 1 0.90841 0.94281 0.49795 1.0000
N40 N 1 0.09159 0.05719 0.50205 1.0000
N41 N 1 0.94281 0.59364 0.49795 1.0000
N42 N 1 0.90841 0.55924 0.49795 1.0000
N43 N 1 0.55924 0.90841 0.49795 1.0000
N44 N 1 0.55924 0.59364 0.49795 1.0000
N45 N 1 0.59364 0.55924 0.49795 1.0000
N46 N 1 0.05719 0.09159 0.50205 1.0000
N47 N 1 0.09159 0.44076 0.50205 1.0000
N48 N 1 0.40636 0.05719 0.50205 1.0000
N49 N 1 0.02604 0.85105 0.34650 1.0000
N50 N 1 0.19755 0.37254 0.65350 1.0000
N51 N 1 0.37254 0.14895 0.65350 1.0000
N52 N 1 0.80245 0.02604 0.34650 1.0000
N53 N 1 0.37254 0.19755 0.65350 1.0000
N54 N 1 0.97396 0.19755 0.65350 1.0000
N55 N 1 0.85105 0.02604 0.34650 1.0000
N56 N 1 0.14895 0.97396 0.65350 1.0000
N57 N 1 0.02604 0.80245 0.34650 1.0000
N58 N 1 0.85105 0.62746 0.34650 1.0000
N59 N 1 0.62746 0.85105 0.34650 1.0000
N60 N 1 0.62746 0.80245 0.34650 1.0000
N61 N 1 0.80245 0.62746 0.34650 1.0000
N62 N 1 0.97396 0.14895 0.65350 1.0000
N63 N 1 0.14895 0.37254 0.65350 1.0000
N64 N 1 0.19755 0.97396 0.65350 1.0000
N65 N 1 0.12067 0.84431 0.31139 1.0000
N66 N 1 0.15569 0.43205 0.68861 1.0000
N67 N 1 0.43205 0.15569 0.68861 1.0000
N68 N 1 0.84431 0.12067 0.31139 1.0000
N69 N 1 0.87933 0.15569 0.68861 1.0000
N70 N 1 0.15569 0.87933 0.68861 1.0000
N71 N 1 0.84431 0.56795 0.31139 1.0000
N72 N 1 0.56795 0.84431 0.31139 1.0000
N73 N 1 0.26565 0.10344 0.00000 1.0000
N74 N 1 0.10344 0.26565 0.00000 1.0000
N75 N 1 0.26565 0.89656 0.00000 1.0000
N76 N 1 0.89656 0.26565 0.00000 1.0000
N77 N 1 0.73435 0.10344 0.00000 1.0000
N78 N 1 0.89656 0.73435 0.00000 1.0000
N79 N 1 0.10344 0.73435 0.00000 1.0000
N80 N 1 0.73435 0.89656 0.00000 1.0000
N81 N 1 0.10891 0.91404 0.43326 1.0000
N82 N 1 0.34730 0.54217 0.56674 1.0000
N83 N 1 0.54217 0.08596 0.56674 1.0000
N84 N 1 0.65270 0.10891 0.43326 1.0000
N85 N 1 0.54217 0.34730 0.56674 1.0000
N86 N 1 0.89109 0.34730 0.56674 1.0000
N87 N 1 0.91404 0.10891 0.43326 1.0000
N88 N 1 0.08596 0.89109 0.56674 1.0000
N89 N 1 0.10891 0.65270 0.43326 1.0000
N90 N 1 0.91404 0.45783 0.43326 1.0000
N91 N 1 0.45783 0.91404 0.43326 1.0000
N92 N 1 0.45783 0.65270 0.43326 1.0000
N93 N 1 0.65270 0.45783 0.43326 1.0000
N94 N 1 0.89109 0.08596 0.56674 1.0000
N95 N 1 0.08596 0.54217 0.56674 1.0000
N96 N 1 0.34730 0.89109 0.56674 1.0000
N97 N 1 0.02654 0.97121 0.36782 1.0000
N98 N 1 0.33903 0.39436 0.63218 1.0000
N99 N 1 0.39436 0.02879 0.63218 1.0000
N100 N 1 0.66097 0.02654 0.36782 1.0000
N101 N 1 0.39436 0.33903 0.63218 1.0000
N102 N 1 0.97346 0.33903 0.63218 1.0000
N103 N 1 0.97121 0.02654 0.36782 1.0000
N104 N 1 0.02879 0.97346 0.63218 1.0000
N105 N 1 0.02654 0.66097 0.36782 1.0000
N106 N 1 0.97121 0.60564 0.36782 1.0000
N107 N 1 0.60564 0.97121 0.36782 1.0000
N108 N 1 0.60564 0.66097 0.36782 1.0000
N109 N 1 0.66097 0.60564 0.36782 1.0000
N110 N 1 0.97346 0.02879 0.63218 1.0000
N111 N 1 0.02879 0.39436 0.63218 1.0000
N112 N 1 0.33903 0.97346 0.63218 1.0000
N113 N 1 0.08437 0.87663 0.32497 1.0000
N114 N 1 0.20160 0.40933 0.67503 1.0000
N115 N 1 0.40933 0.12337 0.67503 1.0000
N116 N 1 0.79840 0.08437 0.32497 1.0000
N117 N 1 0.40933 0.20160 0.67503 1.0000
N118 N 1 0.91563 0.20160 0.67503 1.0000
N119 N 1 0.87663 0.08437 0.32497 1.0000
N120 N 1 0.12337 0.91563 0.67503 1.0000
N121 N 1 0.08437 0.79840 0.32497 1.0000
N122 N 1 0.87663 0.59067 0.32497 1.0000
N123 N 1 0.59067 0.87663 0.32497 1.0000
N20 N 8 0.56940 0.64554 0.93018 1.0000
N21 N 8 0.67250 0.62116 0.92081 1.0000
N22 N 8 0.95769 0.43816 0.05836 1.0000
N23 N 8 0.90769 0.48185 0.09943 1.0000
N24 N 8 0.80460 0.45736 0.09016 1.0000
N25 N 8 0.54239 0.37082 0.75083 1.0000
N26 N 8 0.57305 0.42993 0.71514 1.0000
N27 N 8 0.67492 0.42995 0.73213 1.0000
N28 N 4 0.70132 0.25000 0.03536 1.0000
N29 N 8 0.84320 0.27616 0.06849 1.0000
N30 N 4 0.80325 0.25000 0.78952 1.0000
N31 N 8 0.73308 0.27618 0.83352 1.0000
Cd1 Cd 8 0.79547 0.50283 0.91589 1.0000
Cd2 Cd 8 0.70244 0.37723 0.03906 1.0000
Cd3 Cd 8 0.79560 0.37729 0.79103 1.0000
#End

data_30-mer_Cd
_audit_creation_method TososPro
_Chemical_Name_Systematic VEJZIU
_cell_length_a 28.32135
_cell_length_b 28.32135
_cell_length_c 22.46242
_cell_angle_alpha 50.91915
_cell_angle_beta 50.91915
_cell_angle_gamma 90
_cell_volume 8160.571
_cell_formula_units_Z 16
_symmetry_space_group_name_H-M 'P 1'
_symmetry_Int_Tables_number 1
loop_
_symmetry_equiv_pos_site_id
_symmetry_equiv_pos_as_xyz
1 x,y,z
loop_
_atom_site_label
_atom_site_type_symbol
_atom_site_symmetry_multiplicity
_atom_site_fract_x
_atom_site_fract_y
_atom_site_fract_z
_atom_site_occupancy
N1 N 1 0.13681 0.87667 0.46104 1.0000
N2 N 1 0.33771 0.59785 0.53896 1.0000
N3 N 1 0.59785 0.12333 0.53896 1.0000
N4 N 1 0.66229 0.13681 0.46104 1.0000
N5 N 1 0.59785 0.33771 0.53896 1.0000
N6 N 1 0.86319 0.33771 0.53896 1.0000
N7 N 1 0.87667 0.13681 0.46104 1.0000
N8 N 1 0.12333 0.86319 0.53896 1.0000
N9 N 1 0.13681 0.66229 0.46104 1.0000
N10 N 1 0.87667 0.40215 0.46104 1.0000
N11 N 1 0.40215 0.87667 0.46104 1.0000
N12 N 1 0.40215 0.66229 0.46104 1.0000
N13 N 1 0.66229 0.40215 0.46104 1.0000
N14 N 1 0.86319 0.12333 0.53896 1.0000
N15 N 1 0.12333 0.59785 0.53896 1.0000
N16 N 1 0.33771 0.86319 0.53896 1.0000
N17 N 1 0.07853 0.92147 0.50000 1.0000
N18 N 1 0.42147 0.57853 0.50000 1.0000
N19 N 1 0.57853 0.07853 0.50000 1.0000
N20 N 1 0.57853 0.42147 0.50000 1.0000
N21 N 1 0.92147 0.42147 0.50000 1.0000
N22 N 1 0.92147 0.07853 0.50000 1.0000
N23 N 1 0.07853 0.57853 0.50000 1.0000
N24 N 1 0.42147 0.92147 0.50000 1.0000
N25 N 1 0.03967 0.03967 0.29536 1.0000
N26 N 1 0.33503 0.33503 0.70464 1.0000
N27 N 1 0.33503 0.96033 0.70464 1.0000
N28 N 1 0.66497 0.03967 0.29536 1.0000
N29 N 1 0.96033 0.33503 0.70464 1.0000
N30 N 1 0.96033 0.96033 0.70464 1.0000
N31 N 1 0.03967 0.66497 0.29536 1.0000
N32 N 1 0.66497 0.66497 0.29536 1.0000
N33 N 1 0.94492 0.91265 0.49232 1.0000
N34 N 1 0.40497 0.43723 0.50768 1.0000
N35 N 1 0.43723 0.08735 0.50768 1.0000
N36 N 1 0.59503 0.94492 0.49232 1.0000
N37 N 1 0.43723 0.40497 0.50768 1.0000
N38 N 1 0.05508 0.40497 0.50768 1.0000
N39 N 1 0.91265 0.94492 0.49232 1.0000
N40 N 1 0.08735 0.05508 0.50768 1.0000
N41 N 1 0.94492 0.59503 0.49232 1.0000
N42 N 1 0.91265 0.56277 0.49232 1.0000
N43 N 1 0.56277 0.91265 0.49232 1.0000
N44 N 1 0.56277 0.59503 0.49232 1.0000
N45 N 1 0.59503 0.56277 0.49232 1.0000
N46 N 1 0.05508 0.08735 0.50768 1.0000
N47 N 1 0.08735 0.43723 0.50768 1.0000
N48 N 1 0.40497 0.05508 0.50768 1.0000
N49 N 1 0.02850 0.85000 0.34558 1.0000
N50 N 1 0.19558 0.37408 0.65442 1.0000
N51 N 1 0.37408 0.15000 0.65442 1.0000
N52 N 1 0.80442 0.02850 0.34558 1.0000
N53 N 1 0.37408 0.19558 0.65442 1.0000
N54 N 1 0.97150 0.19558 0.65442 1.0000
N55 N 1 0.85000 0.02850 0.34558 1.0000
N56 N 1 0.15000 0.97150 0.65442 1.0000
N57 N 1 0.02850 0.80442 0.34558 1.0000
N58 N 1 0.85000 0.62592 0.34558 1.0000
N59 N 1 0.62592 0.85000 0.34558 1.0000

N124 N 1 0.59067 0.79840 0.32497 1.0000
N125 N 1 0.79840 0.59067 0.32497 1.0000
N126 N 1 0.91563 0.12337 0.67503 1.0000
N127 N 1 0.12337 0.40933 0.67503 1.0000
N128 N 1 0.20160 0.91563 0.67503 1.0000
N129 N 1 0.23061 0.07154 0.10911 1.0000
N130 N 1 0.18065 0.33972 0.89089 1.0000
N131 N 1 0.33972 0.92846 0.89089 1.0000
N132 N 1 0.81935 0.23061 0.10911 1.0000
N133 N 1 0.33972 0.18065 0.89089 1.0000
N134 N 1 0.76939 0.18065 0.89089 1.0000
N135 N 1 0.07154 0.23061 0.10911 1.0000
N136 N 1 0.92846 0.76939 0.89089 1.0000
N137 N 1 0.23061 0.81935 0.10911 1.0000
N138 N 1 0.07154 0.66028 0.10911 1.0000
N139 N 1 0.66028 0.07154 0.10911 1.0000
N140 N 1 0.66028 0.81935 0.10911 1.0000
N141 N 1 0.81935 0.66028 0.10911 1.0000
N142 N 1 0.76939 0.92846 0.89089 1.0000
N143 N 1 0.92846 0.33972 0.89089 1.0000
N144 N 1 0.18065 0.76939 0.89089 1.0000
N145 N 1 0.28263 0.12866 0.06781 1.0000
N146 N 1 0.19647 0.35045 0.93219 1.0000
N147 N 1 0.35045 0.87134 0.93219 1.0000
N148 N 1 0.80353 0.28263 0.06781 1.0000
N149 N 1 0.35045 0.19647 0.93219 1.0000
N150 N 1 0.71737 0.19647 0.93219 1.0000
N151 N 1 0.12866 0.28263 0.06781 1.0000
N152 N 1 0.87134 0.71737 0.93219 1.0000
N153 N 1 0.28263 0.80353 0.06781 1.0000
N154 N 1 0.12866 0.64955 0.06781 1.0000
N155 N 1 0.64955 0.12866 0.06781 1.0000
N156 N 1 0.64955 0.80353 0.06781 1.0000
N157 N 1 0.80353 0.64955 0.06781 1.0000
N158 N 1 0.71737 0.87134 0.93219 1.0000
N159 N 1 0.87134 0.35045 0.93219 1.0000
N160 N 1 0.19647 0.71737 0.93219 1.0000
Zn1 Zn 1 0.11317 0.95623 0.30960 1.0000
Zn2 Zn 1 0.26583 0.42277 0.69040 1.0000
Zn3 Zn 1 0.42277 0.04377 0.69040 1.0000
Zn4 Zn 1 0.73417 0.11317 0.30960 1.0000
Zn5 Zn 1 0.42277 0.26583 0.69040 1.0000
Zn6 Zn 1 0.88683 0.26583 0.69040 1.0000
Zn7 Zn 1 0.95623 0.11317 0.30960 1.0000
Zn8 Zn 1 0.04377 0.88683 0.69040 1.0000
Zn9 Zn 1 0.11317 0.73417 0.30960 1.0000
Zn10 Zn 1 0.95623 0.57723 0.30960 1.0000
Zn11 Zn 1 0.57723 0.95623 0.30960 1.0000
Zn12 Zn 1 0.57723 0.73417 0.30960 1.0000
Zn13 Zn 1 0.73417 0.57723 0.30960 1.0000
Zn14 Zn 1 0.88683 0.04377 0.69040 1.0000
Zn15 Zn 1 0.04377 0.42277 0.69040 1.0000
Zn16 Zn 1 0.26583 0.88683 0.69040 1.0000
#End

data_30-dia3_Zn
_audit_creation_method ToposPro
_Chemical_Name_Systematic SIVGOV
_cell_length_a 9.503263
_cell_length_b 9.503263
_cell_length_c 9.543764
_cell_angle_alpha 119.3534
_cell_angle_beta 119.3534
_cell_angle_gamma 89.53699
_cell_volume 623.4685
_cell_formula_units_Z 2
_symmetry_space_group_name_H-M 'P 1'
_symmetry_Int_Tables_number 1
loop_
_symmetry_equiv_pos_site_id
_symmetry_equiv_pos_as_xyz
1 x,y,z
loop_
_atom_site_label
_atom_site_type_symbol
_atom_site_symmetry_multiplicity
_atom_site_fract_x
_atom_site_fract_y
_atom_site_fract_z
_atom_site_occupancy
N1 N 1 0.59601 0.09088 0.00302 1.0000
N2 N 1 0.09088 0.59601 0.50302 1.0000
N3 N 1 0.69423 0.35241 0.19014 1.0000
N4 N 1 0.35241 0.69423 0.69014 1.0000
N5 N 1 0.89556 0.78170 0.89876 1.0000
N6 N 1 0.78170 0.89556 0.39876 1.0000
N7 N 1 0.82799 0.68507 0.61513 1.0000
N8 N 1 0.68507 0.82799 0.11513 1.0000
N9 N 1 0.95140 0.72213 0.78897 1.0000
N10 N 1 0.72213 0.95140 0.28897 1.0000
N11 N 1 0.47277 0.14840 0.01937 1.0000
N12 N 1 0.14840 0.47277 0.51937 1.0000
N13 N 1 0.73324 0.21668 0.10868 1.0000
N14 N 1 0.21668 0.73324 0.60868 1.0000
N15 N 1 0.69593 0.72124 0.61616 1.0000
N16 N 1 0.72124 0.69593 0.11616 1.0000
N17 N 1 0.73819 0.78098 0.79174 1.0000
N18 N 1 0.78098 0.73819 0.29174 1.0000
N19 N 1 0.53369 0.31084 0.13536 1.0000
N20 N 1 0.31084 0.53369 0.63536 1.0000
Zn1 Zn 1 0.84878 0.58939 0.38816 1.0000
N60 N 1 0.62592 0.80442 0.34558 1.0000
N61 N 1 0.80442 0.62592 0.34558 1.0000
N62 N 1 0.97150 0.15000 0.65442 1.0000
N63 N 1 0.15000 0.37408 0.65442 1.0000
N64 N 1 0.19558 0.97150 0.65442 1.0000
N65 N 1 0.11688 0.84360 0.31280 1.0000
N66 N 1 0.15640 0.42968 0.68720 1.0000
N67 N 1 0.42968 0.15640 0.68720 1.0000
N68 N 1 0.84360 0.11688 0.31280 1.0000
N69 N 1 0.88312 0.15640 0.68720 1.0000
N70 N 1 0.15640 0.88312 0.68720 1.0000
N71 N 1 0.84360 0.57032 0.31280 1.0000
N72 N 1 0.57032 0.84360 0.31280 1.0000
N73 N 1 0.26771 0.10574 0.00000 1.0000
N74 N 1 0.10574 0.26771 0.00000 1.0000
N75 N 1 0.26771 0.89426 0.00000 1.0000
N76 N 1 0.89426 0.26771 0.00000 1.0000
N77 N 1 0.73229 0.10574 0.00000 1.0000
N78 N 1 0.89426 0.73229 0.00000 1.0000
N79 N 1 0.10574 0.73229 0.00000 1.0000
N80 N 1 0.73229 0.89426 0.00000 1.0000
N81 N 1 0.10908 0.91249 0.43751 1.0000
N82 N 1 0.34999 0.54659 0.56249 1.0000
N83 N 1 0.54659 0.08751 0.56249 1.0000
N84 N 1 0.65001 0.10908 0.43751 1.0000
N85 N 1 0.54659 0.34999 0.56249 1.0000
N86 N 1 0.89092 0.34999 0.56249 1.0000
N87 N 1 0.91249 0.10908 0.43751 1.0000
N88 N 1 0.08751 0.89092 0.56249 1.0000
N89 N 1 0.10908 0.65001 0.43751 1.0000
N90 N 1 0.91249 0.45341 0.43751 1.0000
N91 N 1 0.45341 0.91249 0.43751 1.0000
N92 N 1 0.45341 0.65001 0.43751 1.0000
N93 N 1 0.65001 0.45341 0.43751 1.0000
N94 N 1 0.89092 0.08751 0.56249 1.0000
N95 N 1 0.08751 0.54659 0.56249 1.0000
N96 N 1 0.34999 0.89092 0.56249 1.0000
N97 N 1 0.02298 0.97117 0.37100 1.0000
N98 N 1 0.34218 0.39399 0.62900 1.0000
N99 N 1 0.39399 0.02883 0.62900 1.0000
N100 N 1 0.65782 0.02298 0.37100 1.0000
N101 N 1 0.39399 0.34218 0.62900 1.0000
N102 N 1 0.97702 0.34218 0.62900 1.0000
N103 N 1 0.97117 0.02298 0.37100 1.0000
N104 N 1 0.02883 0.97702 0.62900 1.0000
N105 N 1 0.02298 0.65782 0.37100 1.0000
N106 N 1 0.97117 0.60601 0.37100 1.0000
N107 N 1 0.60601 0.97117 0.37100 1.0000
N108 N 1 0.60601 0.65782 0.37100 1.0000
N109 N 1 0.65782 0.60601 0.37100 1.0000
N110 N 1 0.97702 0.02883 0.62900 1.0000
N111 N 1 0.02883 0.39399 0.62900 1.0000
N112 N 1 0.34218 0.97702 0.62900 1.0000
N113 N 1 0.08293 0.87388 0.32545 1.0000
N114 N 1 0.19934 0.40839 0.67455 1.0000
N115 N 1 0.40839 0.12612 0.67455 1.0000
N116 N 1 0.80066 0.08293 0.32545 1.0000
N117 N 1 0.40839 0.19934 0.67455 1.0000
N118 N 1 0.91707 0.19934 0.67455 1.0000
N119 N 1 0.87388 0.08293 0.32545 1.0000
N120 N 1 0.12612 0.91707 0.67455 1.0000
N121 N 1 0.08293 0.80066 0.32545 1.0000
N122 N 1 0.87388 0.59161 0.32545 1.0000
N123 N 1 0.59161 0.87388 0.32545 1.0000
N124 N 1 0.59161 0.80066 0.32545 1.0000
N125 N 1 0.80066 0.59161 0.32545 1.0000
N126 N 1 0.91707 0.12612 0.67455 1.0000
N127 N 1 0.12612 0.40839 0.67455 1.0000
N128 N 1 0.19934 0.91707 0.67455 1.0000
N129 N 1 0.23496 0.07594 0.10201 1.0000
N130 N 1 0.17795 0.33697 0.89799 1.0000
N131 N 1 0.33697 0.92406 0.89799 1.0000
N132 N 1 0.82205 0.23496 0.10201 1.0000
N133 N 1 0.33697 0.17795 0.89799 1.0000
N134 N 1 0.76504 0.17795 0.89799 1.0000
N135 N 1 0.07594 0.23496 0.10201 1.0000
N136 N 1 0.92406 0.76504 0.89799 1.0000
N137 N 1 0.23496 0.82205 0.10201 1.0000
N138 N 1 0.07594 0.66303 0.10201 1.0000
N139 N 1 0.66303 0.07594 0.10201 1.0000
N140 N 1 0.66303 0.82205 0.10201 1.0000
N141 N 1 0.82205 0.66303 0.10201 1.0000
N142 N 1 0.76504 0.92406 0.89799 1.0000
N143 N 1 0.92406 0.33697 0.89799 1.0000
N144 N 1 0.17795 0.76504 0.89799 1.0000
N145 N 1 0.28350 0.12925 0.06350 1.0000
N146 N 1 0.19275 0.34700 0.93650 1.0000
N147 N 1 0.34700 0.87075 0.93650 1.0000
N148 N 1 0.80725 0.28350 0.06350 1.0000
N149 N 1 0.34700 0.19275 0.93650 1.0000
N150 N 1 0.71650 0.19275 0.93650 1.0000
N151 N 1 0.12925 0.28350 0.06350 1.0000
N152 N 1 0.87075 0.71650 0.93650 1.0000
N153 N 1 0.28350 0.80725 0.06350 1.0000
N154 N 1 0.12925 0.65300 0.06350 1.0000
N155 N 1 0.65300 0.12925 0.06350 1.0000
N156 N 1 0.65300 0.80725 0.06350 1.0000
N157 N 1 0.80725 0.65300 0.06350 1.0000
N158 N 1 0.71650 0.87075 0.93650 1.0000
N159 N 1 0.87075 0.34700 0.93650 1.0000
N160 N 1 0.19275 0.71650 0.93650 1.0000

Zn2 Zn 1 0.58939 0.84878 0.88816 1.0000
#End

data_31-mer2_Zn
_audit_creation_method ToposPro
_Chemical_Name_Systematic GUPCAW
_cell_length_a 26.67214
_cell_length_b 26.67214
_cell_length_c 21.06423
_cell_angle_alpha 50.71972
_cell_angle_beta 50.71972
_cell_angle_gamma 90
_cell_volume 6673.564
_cell_formula_units_Z 16
_symmetry_space_group_name_H-M 'P 1'
_symmetry_Int_Tables_number 1
loop_
_symmetry_equiv_pos_site_id
_symmetry_equiv_pos_as_xyz
1 x,y,z
loop_
_atom_site_label
_atom_site_type_symbol
_atom_site_symmetry_multiplicity
_atom_site_fract_x
_atom_site_fract_y
_atom_site_fract_z
_atom_site_occupancy
N1 N 1 0.09853 0.64762 0.44435 1.0000
N2 N 1 0.54288 0.09197 0.55565 1.0000
N3 N 1 0.90147 0.35238 0.55565 1.0000
N4 N 1 0.35238 0.54288 0.55565 1.0000
N5 N 1 0.09197 0.90147 0.55565 1.0000
N6 N 1 0.64762 0.45712 0.44435 1.0000
N7 N 1 0.90803 0.09853 0.44435 1.0000
N8 N 1 0.45712 0.90803 0.44435 1.0000
N9 N 1 0.07957 0.53905 0.57228 1.0000
N10 N 1 0.65185 0.11133 0.42772 1.0000
N11 N 1 0.92043 0.46095 0.42772 1.0000
N12 N 1 0.46095 0.65185 0.42772 1.0000
N13 N 1 0.11133 0.92043 0.42772 1.0000
N14 N 1 0.53905 0.34815 0.57228 1.0000
N15 N 1 0.88867 0.07957 0.57228 1.0000
N16 N 1 0.34815 0.88867 0.57228 1.0000
N17 N 1 0.93026 0.33873 0.88949 1.0000
N18 N 1 0.81975 0.22822 0.11051 1.0000
N19 N 1 0.06974 0.66127 0.11051 1.0000
N20 N 1 0.66127 0.81975 0.11051 1.0000
N21 N 1 0.22822 0.06974 0.11051 1.0000
N22 N 1 0.33873 0.18025 0.88949 1.0000
N23 N 1 0.77178 0.93026 0.88949 1.0000
N24 N 1 0.18025 0.77178 0.88949 1.0000
N25 N 1 0.12480 0.41049 0.67237 1.0000
N26 N 1 0.79718 0.08287 0.32763 1.0000
N27 N 1 0.87520 0.58951 0.32763 1.0000
N28 N 1 0.58951 0.79718 0.32763 1.0000
N29 N 1 0.08287 0.87520 0.32763 1.0000
N30 N 1 0.41049 0.20282 0.67237 1.0000
N31 N 1 0.91713 0.12480 0.67237 1.0000
N32 N 1 0.20282 0.91713 0.67237 1.0000
N33 N 1 0.20098 0.40822 0.67526 1.0000
N34 N 1 0.87624 0.08348 0.32474 1.0000
N35 N 1 0.79902 0.59178 0.32474 1.0000
N36 N 1 0.59178 0.87624 0.32474 1.0000
N37 N 1 0.08348 0.79902 0.32474 1.0000
N38 N 1 0.40822 0.12376 0.67526 1.0000
N39 N 1 0.91652 0.20098 0.67526 1.0000
N40 N 1 0.12376 0.91652 0.67526 1.0000
N41 N 1 0.22967 0.81983 0.11059 1.0000
N42 N 1 0.34025 0.93042 0.88941 1.0000
N43 N 1 0.77033 0.18017 0.88941 1.0000
N44 N 1 0.18017 0.34025 0.88941 1.0000
N45 N 1 0.93042 0.77033 0.88941 1.0000
N46 N 1 0.81983 0.65975 0.11059 1.0000
N47 N 1 0.06958 0.22967 0.11059 1.0000
N48 N 1 0.65975 0.06958 0.11059 1.0000
N49 N 1 0.97754 0.33622 0.63190 1.0000
N50 N 1 0.60944 0.96812 0.36810 1.0000
N51 N 1 0.02246 0.66378 0.36810 1.0000
N52 N 1 0.66378 0.60944 0.36810 1.0000
N53 N 1 0.96812 0.02246 0.36810 1.0000
N54 N 1 0.33622 0.39056 0.63190 1.0000
N55 N 1 0.03188 0.97754 0.63190 1.0000
N56 N 1 0.39056 0.03188 0.63190 1.0000
N57 N 1 0.03328 0.39271 0.62949 1.0000
N58 N 1 0.66277 0.02220 0.37051 1.0000
N59 N 1 0.96672 0.60729 0.37051 1.0000
N60 N 1 0.60729 0.66277 0.37051 1.0000
N61 N 1 0.02220 0.96672 0.37051 1.0000
N62 N 1 0.39271 0.33723 0.62949 1.0000
N63 N 1 0.97780 0.03328 0.62949 1.0000
N64 N 1 0.33723 0.97780 0.62949 1.0000
N65 N 1 0.08126 0.57892 0.49007 1.0000
N66 N 1 0.57132 0.06899 0.50993 1.0000
N67 N 1 0.91874 0.42108 0.50993 1.0000
N68 N 1 0.42108 0.57132 0.50993 1.0000
N69 N 1 0.06899 0.91874 0.50993 1.0000
N70 N 1 0.57892 0.42868 0.49007 1.0000
N71 N 1 0.93101 0.08126 0.49007 1.0000
N72 N 1 0.42868 0.93101 0.49007 1.0000
N73 N 1 0.89552 0.26201 0.00000 1.0000
Cd1 Cd 1 0.11345 0.95628 0.30942 1.0000
Cd2 Cd 1 0.26570 0.42287 0.69058 1.0000
Cd3 Cd 1 0.42287 0.04372 0.69058 1.0000
Cd4 Cd 1 0.73430 0.11345 0.30942 1.0000
Cd5 Cd 1 0.42287 0.26570 0.69058 1.0000
Cd6 Cd 1 0.88655 0.26570 0.69058 1.0000
Cd7 Cd 1 0.95628 0.11345 0.30942 1.0000
Cd8 Cd 1 0.04372 0.88655 0.69058 1.0000
Cd9 Cd 1 0.11345 0.73430 0.30942 1.0000
Cd10 Cd 1 0.95628 0.57713 0.30942 1.0000
Cd11 Cd 1 0.57713 0.95628 0.30942 1.0000
Cd12 Cd 1 0.57713 0.73430 0.30942 1.0000
Cd13 Cd 1 0.73430 0.57713 0.30942 1.0000
Cd14 Cd 1 0.88655 0.04372 0.69058 1.0000
Cd15 Cd 1 0.04372 0.42287 0.69058 1.0000
Cd16 Cd 1 0.26570 0.88655 0.69058 1.0000
#End

data_31-dft_Cd
_audit_creation_method ToposPro
_Chemical_Name_Systematic VEJYOZ
_cell_length_a 20.05629
_cell_length_b 20.05629
_cell_length_c 17.2208
_cell_angle_alpha 90
_cell_angle_beta 90
_cell_angle_gamma 90
_cell_volume 6927.151
_cell_formula_units_Z 16
_symmetry_space_group_name_H-M 'P 42/m n m'
_symmetry_Int_Tables_number 136
loop_
_symmetry_equiv_pos_site_id
_symmetry_equiv_pos_as_xyz
1 x,y,z
2 -x,-y,z
3 1/2+x,1/2-y,1/2-z
4 1/2-x,1/2+y,1/2-z
5 -y,-x,-z
6 y,x,-z
7 1/2+y,1/2-x,1/2+z
8 1/2-y,1/2+x,1/2+z
9 -x,-y,-z
10 x,y,-z
11 1/2-x,1/2+y,1/2+z
12 1/2+x,1/2-y,1/2+z
13 y,x,z
14 -y,-x,z
15 1/2-y,1/2+x,1/2-z
16 1/2+y,1/2-x,1/2-z
loop_
_atom_site_label
_atom_site_type_symbol
_atom_site_symmetry_multiplicity
_atom_site_fract_x
_atom_site_fract_y
_atom_site_fract_z
_atom_site_occupancy
N1 N 8 0.70974 0.29026 0.17247 1.0000
N2 N 16 0.64255 0.31193 0.26744 1.0000
N3 N 16 0.63736 0.03403 0.24780 1.0000
N4 N 16 0.69854 0.10683 0.30698 1.0000
N5 N 16 0.67824 0.11160 0.03751 1.0000
N6 N 8 0.59783 0.17588 0.00000 1.0000
N7 N 16 0.71476 0.04888 0.33366 1.0000
N8 N 16 0.65611 0.27081 0.20891 1.0000
N9 N 16 0.65093 0.09738 0.25424 1.0000
N10 N 16 0.62864 0.15122 0.06027 1.0000
N11 N 16 0.67696 0.00426 0.29705 1.0000
Cd1 Cd 16 0.60410 0.17677 0.18249 1.0000
#End

data_32-mer2_Cd
_audit_creation_method ToposPro
_Chemical_Name_Systematic GUPCAW
_cell_length_a 28.53581
_cell_length_b 28.53581
_cell_length_c 22.53795
_cell_angle_alpha 50.72365
_cell_angle_beta 50.72365
_cell_angle_gamma 90
_cell_volume 8175.957
_cell_formula_units_Z 16
_symmetry_space_group_name_H-M 'P 1'
_symmetry_Int_Tables_number 1
loop_
_symmetry_equiv_pos_site_id
_symmetry_equiv_pos_as_xyz
1 x,y,z
loop_
_atom_site_label
_atom_site_type_symbol
_atom_site_symmetry_multiplicity
_atom_site_fract_x
_atom_site_fract_y
_atom_site_fract_z
_atom_site_occupancy
N1 N 1 0.09838 0.64477 0.44901 1.0000
N2 N 1 0.54738 0.09378 0.55099 1.0000
N3 N 1 0.90162 0.35523 0.55099 1.0000
N4 N 1 0.35523 0.54738 0.55099 1.0000

N74 N 1 0.10448 0.73799 0.00000 1.0000	N5 N 1 0.09378 0.90162 0.55099 1.0000
N75 N 1 0.73799 0.89552 0.00000 1.0000	N6 N 1 0.64477 0.45262 0.44901 1.0000
N76 N 1 0.26201 0.10448 0.00000 1.0000	N7 N 1 0.90622 0.09838 0.44901 1.0000
N77 N 1 0.87639 0.35234 0.93128 1.0000	N8 N 1 0.45262 0.90622 0.44901 1.0000
N78 N 1 0.80767 0.28362 0.06872 1.0000	N9 N 1 0.08053 0.54318 0.56887 1.0000
N79 N 1 0.12361 0.64766 0.06872 1.0000	N10 N 1 0.64940 0.11205 0.43113 1.0000
N80 N 1 0.64766 0.80767 0.06872 1.0000	N11 N 1 0.91947 0.45682 0.43113 1.0000
N81 N 1 0.28362 0.12361 0.06872 1.0000	N12 N 1 0.45682 0.64940 0.43113 1.0000
N82 N 1 0.35234 0.19233 0.93128 1.0000	N13 N 1 0.11205 0.91947 0.43113 1.0000
N83 N 1 0.71638 0.87639 0.93128 1.0000	N14 N 1 0.54318 0.35060 0.56887 1.0000
N84 N 1 0.19233 0.71638 0.93128 1.0000	N15 N 1 0.88795 0.08053 0.56887 1.0000
N85 N 1 0.15961 0.43541 0.68094 1.0000	N16 N 1 0.35060 0.88795 0.56887 1.0000
N86 N 1 0.84054 0.11635 0.31906 1.0000	N17 N 1 0.92590 0.33602 0.89665 1.0000
N87 N 1 0.84039 0.56459 0.31906 1.0000	N18 N 1 0.82256 0.23267 0.10335 1.0000
N88 N 1 0.56459 0.84054 0.31906 1.0000	N19 N 1 0.07410 0.66398 0.10335 1.0000
N89 N 1 0.11635 0.84039 0.31906 1.0000	N20 N 1 0.66398 0.82256 0.10335 1.0000
N90 N 1 0.43541 0.15946 0.68094 1.0000	N21 N 1 0.23267 0.07410 0.10335 1.0000
N91 N 1 0.88365 0.15961 0.68094 1.0000	N22 N 1 0.33602 0.17744 0.89665 1.0000
N92 N 1 0.15946 0.88365 0.68094 1.0000	N23 N 1 0.76733 0.92590 0.89665 1.0000
N93 N 1 0.26053 0.89196 0.00000 1.0000	N24 N 1 0.17744 0.76733 0.89665 1.0000
N94 N 1 0.73947 0.10804 0.00000 1.0000	N25 N 1 0.12741 0.40939 0.67214 1.0000
N95 N 1 0.10804 0.26053 0.00000 1.0000	N26 N 1 0.79955 0.08152 0.32786 1.0000
N96 N 1 0.89196 0.73947 0.00000 1.0000	N27 N 1 0.87259 0.59061 0.32786 1.0000
N97 N 1 0.28992 0.81370 0.06874 1.0000	N28 N 1 0.59061 0.79955 0.32786 1.0000
N98 N 1 0.35867 0.88244 0.93126 1.0000	N29 N 1 0.08152 0.87259 0.32786 1.0000
N99 N 1 0.71008 0.18630 0.93126 1.0000	N30 N 1 0.40939 0.20045 0.67214 1.0000
N100 N 1 0.18630 0.35867 0.93126 1.0000	N31 N 1 0.91848 0.12741 0.67214 1.0000
N101 N 1 0.88244 0.71008 0.93126 1.0000	N32 N 1 0.20045 0.91848 0.67214 1.0000
N102 N 1 0.81370 0.64133 0.06874 1.0000	N33 N 1 0.19864 0.40721 0.67500 1.0000
N103 N 1 0.11756 0.28992 0.06874 1.0000	N34 N 1 0.87364 0.08221 0.32500 1.0000
N104 N 1 0.64133 0.11756 0.06874 1.0000	N35 N 1 0.80136 0.59279 0.32500 1.0000
N105 N 1 0.95842 0.33470 0.70773 1.0000	N36 N 1 0.59279 0.87364 0.32500 1.0000
N106 N 1 0.66615 0.04243 0.29227 1.0000	N37 N 1 0.08221 0.80136 0.32500 1.0000
N107 N 1 0.04158 0.66530 0.29227 1.0000	N38 N 1 0.40721 0.12636 0.67500 1.0000
N108 N 1 0.66530 0.66615 0.29227 1.0000	N39 N 1 0.91779 0.19864 0.67500 1.0000
N109 N 1 0.04243 0.04158 0.29227 1.0000	N40 N 1 0.12636 0.91779 0.67500 1.0000
N110 N 1 0.33470 0.33385 0.70773 1.0000	N41 N 1 0.23423 0.82281 0.10339 1.0000
N111 N 1 0.95757 0.95842 0.70773 1.0000	N42 N 1 0.33762 0.92620 0.89661 1.0000
N112 N 1 0.33385 0.95757 0.70773 1.0000	N43 N 1 0.76577 0.17719 0.89661 1.0000
N113 N 1 0.06360 0.39481 0.50753 1.0000	N44 N 1 0.17719 0.33762 0.89661 1.0000
N114 N 1 0.57114 0.90234 0.49247 1.0000	N45 N 1 0.92620 0.76577 0.89661 1.0000
N115 N 1 0.93640 0.60519 0.49247 1.0000	N46 N 1 0.82281 0.66238 0.10339 1.0000
N116 N 1 0.60519 0.57114 0.49247 1.0000	N47 N 1 0.07380 0.23423 0.10339 1.0000
N117 N 1 0.90234 0.93640 0.49247 1.0000	N48 N 1 0.66238 0.07380 0.10339 1.0000
N118 N 1 0.39481 0.42886 0.50753 1.0000	N49 N 1 0.98107 0.33913 0.62894 1.0000
N119 N 1 0.09766 0.06360 0.50753 1.0000	N50 N 1 0.61001 0.96807 0.37106 1.0000
N120 N 1 0.42886 0.09766 0.50753 1.0000	N51 N 1 0.01893 0.66087 0.37106 1.0000
N121 N 1 0.09826 0.42992 0.50607 1.0000	N52 N 1 0.66087 0.61001 0.37106 1.0000
N122 N 1 0.60433 0.93599 0.49393 1.0000	N53 N 1 0.96807 0.01893 0.37106 1.0000
N123 N 1 0.90174 0.57008 0.49393 1.0000	N54 N 1 0.33913 0.38999 0.62894 1.0000
N124 N 1 0.57008 0.60433 0.49393 1.0000	N55 N 1 0.03193 0.98107 0.62894 1.0000
N125 N 1 0.93599 0.90174 0.49393 1.0000	N56 N 1 0.38999 0.03193 0.62894 1.0000
N126 N 1 0.42992 0.39567 0.50607 1.0000	N57 N 1 0.03328 0.39204 0.62666 1.0000
N127 N 1 0.06401 0.09826 0.50607 1.0000	N58 N 1 0.65994 0.01870 0.37334 1.0000
N128 N 1 0.39567 0.06401 0.50607 1.0000	N59 N 1 0.96672 0.60796 0.37334 1.0000
N129 N 1 0.85585 0.63223 0.33783 1.0000	N60 N 1 0.60796 0.65994 0.37334 1.0000
N130 N 1 0.19368 0.97006 0.66217 1.0000	N61 N 1 0.01870 0.96672 0.37334 1.0000
N131 N 1 0.14415 0.36777 0.66217 1.0000	N62 N 1 0.39204 0.34006 0.62666 1.0000
N132 N 1 0.36777 0.19368 0.66217 1.0000	N63 N 1 0.98130 0.03328 0.62666 1.0000
N133 N 1 0.97006 0.14415 0.66217 1.0000	N64 N 1 0.34006 0.98130 0.62666 1.0000
N134 N 1 0.63223 0.80632 0.33783 1.0000	N65 N 1 0.08217 0.58046 0.49190 1.0000
N135 N 1 0.02994 0.85585 0.33783 1.0000	N66 N 1 0.57407 0.07236 0.50810 1.0000
N136 N 1 0.80632 0.02994 0.33783 1.0000	N67 N 1 0.91783 0.41954 0.50810 1.0000
N137 N 1 0.80850 0.63359 0.33612 1.0000	N68 N 1 0.41954 0.57407 0.50810 1.0000
N138 N 1 0.14462 0.96971 0.66388 1.0000	N69 N 1 0.07236 0.91783 0.50810 1.0000
N139 N 1 0.19150 0.36641 0.66388 1.0000	N70 N 1 0.58046 0.42593 0.49190 1.0000
N140 N 1 0.36641 0.14462 0.66388 1.0000	N71 N 1 0.92764 0.08217 0.49190 1.0000
N141 N 1 0.96971 0.19150 0.66388 1.0000	N72 N 1 0.42593 0.92764 0.49190 1.0000
N142 N 1 0.63359 0.85538 0.33612 1.0000	N73 N 1 0.89346 0.26430 0.00000 1.0000
N143 N 1 0.03029 0.80850 0.33612 1.0000	N74 N 1 0.10654 0.73570 0.00000 1.0000
N144 N 1 0.85538 0.03029 0.33612 1.0000	N75 N 1 0.73570 0.89346 0.00000 1.0000
N145 N 1 0.89266 0.34979 0.50192 1.0000	N76 N 1 0.26430 0.10654 0.00000 1.0000
N146 N 1 0.39458 0.85171 0.49808 1.0000	N77 N 1 0.87555 0.34868 0.93564 1.0000
N147 N 1 0.10734 0.65021 0.49808 1.0000	N78 N 1 0.81119 0.28432 0.06436 1.0000
N148 N 1 0.65021 0.39458 0.49808 1.0000	N79 N 1 0.12445 0.65132 0.06436 1.0000
N149 N 1 0.85171 0.10734 0.49808 1.0000	N80 N 1 0.65132 0.81119 0.06436 1.0000
N150 N 1 0.34979 0.60542 0.50192 1.0000	N81 N 1 0.28432 0.12445 0.06436 1.0000
N151 N 1 0.14829 0.89266 0.50192 1.0000	N82 N 1 0.34868 0.18881 0.93564 1.0000
N152 N 1 0.60542 0.14829 0.50192 1.0000	N83 N 1 0.71568 0.87555 0.93564 1.0000
N153 N 1 0.90459 0.41736 0.42221 1.0000	N84 N 1 0.18881 0.71568 0.93564 1.0000
N154 N 1 0.32680 0.83958 0.57779 1.0000	N85 N 1 0.16003 0.43274 0.68007 1.0000
N155 N 1 0.09541 0.58264 0.57779 1.0000	N86 N 1 0.84010 0.11282 0.31993 1.0000
N156 N 1 0.58264 0.32680 0.57779 1.0000	N87 N 1 0.83997 0.56726 0.31993 1.0000
N157 N 1 0.83958 0.09541 0.57779 1.0000	N88 N 1 0.56726 0.84010 0.31993 1.0000
N158 N 1 0.41736 0.67320 0.42221 1.0000	N89 N 1 0.11282 0.83997 0.31993 1.0000
N159 N 1 0.16042 0.90459 0.42221 1.0000	N90 N 1 0.43274 0.15990 0.68007 1.0000
N160 N 1 0.67320 0.16042 0.42221 1.0000	N91 N 1 0.88718 0.16003 0.68007 1.0000
Zn1 Zn 1 0.04663 0.42385 0.68554 1.0000	N92 N 1 0.15990 0.88718 0.68007 1.0000
Zn2 Zn 1 0.73216 0.10938 0.31446 1.0000	N93 N 1 0.26310 0.89030 0.00000 1.0000
Zn3 Zn 1 0.95337 0.57615 0.31446 1.0000	N94 N 1 0.73690 0.10970 0.00000 1.0000
Zn4 Zn 1 0.57615 0.73216 0.31446 1.0000	N95 N 1 0.10970 0.26310 0.00000 1.0000
Zn5 Zn 1 0.10938 0.95337 0.31446 1.0000	N96 N 1 0.89030 0.73690 0.00000 1.0000
Zn6 Zn 1 0.42385 0.26784 0.68554 1.0000	N97 N 1 0.29043 0.81699 0.06437 1.0000
Zn7 Zn 1 0.89062 0.04663 0.68554 1.0000	N98 N 1 0.35479 0.88135 0.93563 1.0000
Zn8 Zn 1 0.26784 0.89062 0.68554 1.0000	N99 N 1 0.70957 0.18301 0.93563 1.0000
Zn9 Zn 1 0.10915 0.73317 0.31259 1.0000	N100 N 1 0.18301 0.35479 0.93563 1.0000
Zn10 Zn 1 0.42175 0.04576 0.68741 1.0000	N101 N 1 0.88135 0.70957 0.93563 1.0000
Zn11 Zn 1 0.89085 0.26683 0.68741 1.0000	N102 N 1 0.81699 0.64521 0.06437 1.0000
Zn12 Zn 1 0.26683 0.42175 0.68741 1.0000	N103 N 1 0.11865 0.29043 0.06437 1.0000
Zn13 Zn 1 0.04576 0.89085 0.68741 1.0000	N104 N 1 0.64521 0.11865 0.06437 1.0000
Zn14 Zn 1 0.73317 0.57825 0.31259 1.0000	N105 N 1 0.96327 0.33773 0.69984 1.0000

Zn15 Zn 1 0.95424 0.10915 0.31259 1.0000
Zn16 Zn 1 0.57825 0.95424 0.31259 1.0000
#End

data_32-dia4_Zn
_audit_creation_method TopsPro
_Chemical_Name_Systematic KEYSEO
_cell_length_a 9.665467
_cell_length_b 9.665467
_cell_length_c 10.35533
_cell_angle_alpha 90
_cell_angle_beta 90
_cell_angle_gamma 90
_cell_volume 967.408
_cell_formula_units_Z 4
_symmetry_space_group_name_H-M 'P 43 21 2'
_symmetry_Int_Tables_number 96
loop_
_symmetry_equiv_pos_site_id
_symmetry_equiv_pos_as_xyz
1 x,y,z
2 -x,-y,1/2+z
3 1/2+x,1/2-y,1/4-z
4 1/2-x,1/2+y,3/4-z
5 -y,-x,1/2-z
6 y,x,-z
7 1/2+y,1/2-x,1/4+z
8 1/2-y,1/2+x,3/4+z
loop_
_atom_site_label
_atom_site_type_symbol
_atom_site_symmetry_multiplicity
_atom_site_fract_x
_atom_site_fract_y
_atom_site_fract_z
_atom_site_occupancy
N1 N 8 0.26892 0.77416 0.39374 1.0000
N2 N 8 0.20166 0.74079 0.50163 1.0000
N3 N 8 0.19436 0.95675 0.47253 1.0000
N4 N 8 0.15540 0.85429 0.55079 1.0000
N5 N 8 0.26456 0.90755 0.37542 1.0000
Zn1 Zn 4 0.34393 0.65607 0.25000 1.0000
#End

data_33-gis_Zn
_audit_creation_method TopsPro
_Chemical_Name_Systematic EQOCOC01
_cell_length_a 18.04816
_cell_length_b 18.04816
_cell_length_c 16.20996
_cell_angle_alpha 56.17213
_cell_angle_beta 56.17213
_cell_angle_gamma 90
_cell_volume 3255.646
_cell_formula_units_Z 8
_symmetry_space_group_name_H-M 'P 1'
_symmetry_Int_Tables_number 1
loop_
_symmetry_equiv_pos_site_id
_symmetry_equiv_pos_as_xyz
1 x,y,z
loop_
_atom_site_label
_atom_site_type_symbol
_atom_site_symmetry_multiplicity
_atom_site_fract_x
_atom_site_fract_y
_atom_site_fract_z
_atom_site_occupancy
N1 N 1 0.34514 0.50000 0.00000 1.0000
N2 N 1 0.34514 0.00000 0.00000 1.0000
N3 N 1 0.00000 0.84514 0.50000 1.0000
N4 N 1 0.50000 0.15486 0.50000 1.0000
N5 N 1 0.65486 0.00000 0.00000 1.0000
N6 N 1 0.50000 0.84514 0.50000 1.0000
N7 N 1 0.00000 0.15486 0.50000 1.0000
N8 N 1 0.65486 0.50000 0.00000 1.0000
N9 N 1 0.22881 0.12528 0.24944 1.0000
N10 N 1 0.37472 0.97825 0.25056 1.0000
N11 N 1 0.62528 0.02175 0.74944 1.0000
N12 N 1 0.47825 0.87472 0.75056 1.0000
N13 N 1 0.52175 0.12528 0.24944 1.0000
N14 N 1 0.37472 0.27119 0.25056 1.0000
N15 N 1 0.77119 0.87472 0.75056 1.0000
N16 N 1 0.62528 0.72881 0.74944 1.0000
N17 N 1 0.14126 0.18484 0.20158 1.0000
N18 N 1 0.14126 0.11358 0.20158 1.0000
N19 N 1 0.31516 0.84284 0.29842 1.0000
N20 N 1 0.61358 0.15716 0.70158 1.0000
N21 N 1 0.34284 0.81516 0.79842 1.0000
N22 N 1 0.65716 0.11358 0.20158 1.0000
N23 N 1 0.38642 0.84284 0.29842 1.0000
N24 N 1 0.31516 0.35874 0.29842 1.0000
N25 N 1 0.38642 0.35874 0.29842 1.0000
N26 N 1 0.34284 0.88642 0.79842 1.0000
N27 N 1 0.85874 0.88642 0.79842 1.0000
N28 N 1 0.85874 0.81516 0.79842 1.0000
N29 N 1 0.68484 0.15716 0.70158 1.0000
N30 N 1 0.65716 0.18484 0.20158 1.0000
N31 N 1 0.61358 0.64126 0.70158 1.0000
N32 N 1 0.68484 0.64126 0.70158 1.0000
N106 N 1 0.66311 0.03757 0.30016 1.0000
N107 N 1 0.03673 0.66227 0.30016 1.0000
N108 N 1 0.66227 0.66311 0.30016 1.0000
N109 N 1 0.03757 0.03673 0.30016 1.0000
N110 N 1 0.33773 0.33689 0.69984 1.0000
N111 N 1 0.96243 0.96327 0.69984 1.0000
N112 N 1 0.33689 0.96243 0.69984 1.0000
N113 N 1 0.06140 0.39390 0.51280 1.0000
N114 N 1 0.57420 0.90670 0.48720 1.0000
N115 N 1 0.93860 0.60610 0.48720 1.0000
N116 N 1 0.60610 0.57420 0.48720 1.0000
N117 N 1 0.90670 0.93860 0.48720 1.0000
N118 N 1 0.39390 0.42580 0.51280 1.0000
N119 N 1 0.09330 0.06140 0.51280 1.0000
N120 N 1 0.42580 0.09330 0.51280 1.0000
N121 N 1 0.09390 0.42683 0.51140 1.0000
N122 N 1 0.60530 0.93823 0.48860 1.0000
N123 N 1 0.90610 0.57317 0.48860 1.0000
N124 N 1 0.57317 0.60530 0.48860 1.0000
N125 N 1 0.93823 0.90610 0.48860 1.0000
N126 N 1 0.42683 0.39470 0.51140 1.0000
N127 N 1 0.06177 0.09390 0.51140 1.0000
N128 N 1 0.39470 0.06177 0.51140 1.0000
N129 N 1 0.85448 0.63046 0.33744 1.0000
N130 N 1 0.19192 0.96789 0.66256 1.0000
N131 N 1 0.14552 0.36954 0.66256 1.0000
N132 N 1 0.36954 0.19192 0.66256 1.0000
N133 N 1 0.96789 0.14552 0.66256 1.0000
N134 N 1 0.63046 0.80808 0.33744 1.0000
N135 N 1 0.03211 0.85448 0.33744 1.0000
N136 N 1 0.80808 0.03211 0.33744 1.0000
N137 N 1 0.81015 0.63180 0.33568 1.0000
N138 N 1 0.14583 0.96748 0.66432 1.0000
N139 N 1 0.18985 0.36820 0.66432 1.0000
N140 N 1 0.36820 0.14583 0.66432 1.0000
N141 N 1 0.96748 0.18985 0.66432 1.0000
N142 N 1 0.63180 0.85417 0.33568 1.0000
N143 N 1 0.03252 0.81015 0.33568 1.0000
N144 N 1 0.85417 0.03252 0.33568 1.0000
N145 N 1 0.89341 0.35275 0.50087 1.0000
N146 N 1 0.39428 0.85362 0.49913 1.0000
N147 N 1 0.10659 0.64725 0.49913 1.0000
N148 N 1 0.64725 0.39428 0.49913 1.0000
N149 N 1 0.85362 0.10659 0.49913 1.0000
N150 N 1 0.35275 0.60572 0.50087 1.0000
N151 N 1 0.14638 0.89341 0.50087 1.0000
N152 N 1 0.60572 0.14638 0.50087 1.0000
N153 N 1 0.90458 0.41604 0.42616 1.0000
N154 N 1 0.33074 0.84221 0.57384 1.0000
N155 N 1 0.09542 0.58396 0.57384 1.0000
N156 N 1 0.58396 0.33074 0.57384 1.0000
N157 N 1 0.84221 0.09542 0.57384 1.0000
N158 N 1 0.41604 0.66926 0.42616 1.0000
N159 N 1 0.15779 0.90458 0.42616 1.0000
N160 N 1 0.66926 0.15779 0.42616 1.0000
Cd1 Cd 1 0.04651 0.42395 0.68572 1.0000
Cd2 Cd 1 0.73223 0.10967 0.31428 1.0000
Cd3 Cd 1 0.95349 0.57605 0.31428 1.0000
Cd4 Cd 1 0.57605 0.73223 0.31428 1.0000
Cd5 Cd 1 0.10967 0.95349 0.31428 1.0000
Cd6 Cd 1 0.42395 0.26777 0.68572 1.0000
Cd7 Cd 1 0.89033 0.04651 0.68572 1.0000
Cd8 Cd 1 0.26777 0.89033 0.68572 1.0000
Cd9 Cd 1 0.10954 0.73332 0.31231 1.0000
Cd10 Cd 1 0.42184 0.04562 0.68769 1.0000
Cd11 Cd 1 0.89046 0.26668 0.68769 1.0000
Cd12 Cd 1 0.26668 0.42184 0.68769 1.0000
Cd13 Cd 1 0.04562 0.89046 0.68769 1.0000
Cd14 Cd 1 0.73332 0.57816 0.31231 1.0000
Cd15 Cd 1 0.95438 0.10954 0.31231 1.0000
Cd16 Cd 1 0.57816 0.95438 0.31231 1.0000
#End

data_33-sra_Cd
_audit_creation_method TopsPro
_Chemical_Name_Systematic BISPAU
_cell_length_a 10.43327
_cell_length_b 13.51776
_cell_length_c 21.60229
_cell_angle_alpha 90
_cell_angle_beta 96.57137
_cell_angle_gamma 90
_cell_volume 3026.651
_cell_formula_units_Z 8
_symmetry_space_group_name_H-M 'P 21/c'
_symmetry_Int_Tables_number 14
loop_
_symmetry_equiv_pos_site_id
_symmetry_equiv_pos_as_xyz
1 x,y,z
2 -x,1/2+y,1/2-z
3 -x,-y,-z
4 x,1/2-y,1/2+z
loop_
_atom_site_label
_atom_site_type_symbol
_atom_site_symmetry_multiplicity
_atom_site_fract_x
_atom_site_fract_y
_atom_site_fract_z
_atom_site_occupancy

N33 N 1 0.25716 0.42569 0.08812 1.0000
N34 N 1 0.25716 0.98619 0.08812 1.0000
N35 N 1 0.07431 0.84528 0.41188 1.0000
N36 N 1 0.48619 0.15472 0.58812 1.0000
N37 N 1 0.34528 0.57431 0.91188 1.0000
N38 N 1 0.65472 0.98619 0.08812 1.0000
N39 N 1 0.51381 0.84528 0.41188 1.0000
N40 N 1 0.07431 0.24284 0.41188 1.0000
N41 N 1 0.51381 0.24284 0.41188 1.0000
N42 N 1 0.34528 0.01381 0.91188 1.0000
N43 N 1 0.74284 0.01381 0.91188 1.0000
N44 N 1 0.74284 0.57431 0.91188 1.0000
N45 N 1 0.92569 0.15472 0.58812 1.0000
N46 N 1 0.65472 0.42569 0.08812 1.0000
N47 N 1 0.48619 0.75716 0.58812 1.0000
N48 N 1 0.92569 0.75716 0.58812 1.0000
N49 N 1 0.19533 0.19192 0.23097 1.0000
N50 N 1 0.19533 0.07710 0.23097 1.0000
N51 N 1 0.30808 0.92630 0.26903 1.0000
N52 N 1 0.57710 0.07370 0.73097 1.0000
N53 N 1 0.42630 0.80808 0.76903 1.0000
N54 N 1 0.57370 0.07710 0.23097 1.0000
N55 N 1 0.42290 0.92630 0.26903 1.0000
N56 N 1 0.30808 0.30467 0.26903 1.0000
N57 N 1 0.42290 0.30467 0.26903 1.0000
N58 N 1 0.42630 0.92290 0.76903 1.0000
N59 N 1 0.80467 0.92290 0.76903 1.0000
N60 N 1 0.80467 0.80808 0.76903 1.0000
N61 N 1 0.69192 0.07370 0.73097 1.0000
N62 N 1 0.57370 0.19192 0.23097 1.0000
N63 N 1 0.57710 0.69533 0.73097 1.0000
N64 N 1 0.69192 0.69533 0.73097 1.0000
N65 N 1 0.25799 0.54618 0.94525 1.0000
N66 N 1 0.25799 0.00857 0.94525 1.0000
N67 N 1 0.95382 0.70324 0.55475 1.0000
N68 N 1 0.50857 0.29676 0.44525 1.0000
N69 N 1 0.20324 0.45382 0.05475 1.0000
N70 N 1 0.79676 0.00857 0.94525 1.0000
N71 N 1 0.49143 0.70324 0.55475 1.0000
N72 N 1 0.95382 0.24201 0.55475 1.0000
N73 N 1 0.49143 0.24201 0.55475 1.0000
N74 N 1 0.20324 0.99143 0.05475 1.0000
N75 N 1 0.74201 0.99143 0.05475 1.0000
N76 N 1 0.74201 0.45382 0.05475 1.0000
N77 N 1 0.04618 0.29676 0.44525 1.0000
N78 N 1 0.79676 0.54618 0.94525 1.0000
N79 N 1 0.50857 0.75799 0.44525 1.0000
N80 N 1 0.04618 0.75799 0.44525 1.0000
Zn1 Zn 1 0.21024 0.28976 0.25000 1.0000
Zn2 Zn 1 0.21024 0.96024 0.25000 1.0000
Zn3 Zn 1 0.46024 0.03976 0.75000 1.0000
Zn4 Zn 1 0.46024 0.71024 0.75000 1.0000
Zn5 Zn 1 0.53976 0.96024 0.25000 1.0000
Zn6 Zn 1 0.53976 0.28976 0.25000 1.0000
Zn7 Zn 1 0.78976 0.03976 0.75000 1.0000
Zn8 Zn 1 0.78976 0.71024 0.75000 1.0000
#End

data_34-crb3_Zn
_audit_creation_method TopoSPro
_Chemical_Name_Systematic VEJYIT
_cell_length_a 9.426752
_cell_length_b 24.11033
_cell_length_c 23.4076
_cell_angle_alpha 90
_cell_angle_beta 90
_cell_angle_gamma 90
_cell_volume 5320.127
_cell_formula_units_Z 16
_symmetry_space_group_name_H-M 'P b c a'
_symmetry_Int_Tables_number 61
loop_
_symmetry_equiv_pos_site_id
_symmetry_equiv_pos_as_xyz
1 x,y,z
2 1/2-x,-y,1/2+z
3 1/2+x,1/2-y,-z
4 -x,1/2+y,1/2-z
5 -x,-y,-z
6 1/2+x,y,1/2-z
7 1/2-x,1/2+y,z
8 x,1/2-y,1/2+z
loop_
_atom_site_label
_atom_site_type_symbol
_atom_site_symmetry_multiplicity
_atom_site_fract_x
_atom_site_fract_y
_atom_site_fract_z
_atom_site_occupancy
N1 N 8 0.12404 0.23539 0.48190 1.0000
N2 N 8 0.29953 0.28718 0.47927 1.0000
N3 N 8 0.00096 0.08267 0.41973 1.0000
N4 N 8 0.74075 0.01269 0.25317 1.0000
N5 N 8 0.10054 0.27269 0.44072 1.0000
N6 N 8 0.87135 0.99544 0.25717 1.0000
N7 N 8 0.90713 0.96652 0.21077 1.0000
N8 N 8 0.24710 0.24421 0.50575 1.0000
N9 N 8 0.69585 0.99441 0.20393 1.0000
N10 N 8 0.79809 0.96592 0.17768 1.0000
N11 N 8 0.98804 0.07936 0.57917 1.0000
N1 N 4 0.51094 0.48721 0.34414 1.0000
N2 N 4 0.56430 0.56223 0.37527 1.0000
N3 N 4 0.68134 0.56725 0.35965 1.0000
N4 N 4 0.70034 0.49591 0.31923 1.0000
N5 N 4 0.59432 0.44612 0.30953 1.0000
N6 N 4 0.18727 0.55590 0.38175 1.0000
N7 N 4 0.06437 0.56603 0.36356 1.0000
N8 N 4 0.02253 0.62902 0.40243 1.0000
N9 N 4 0.11882 0.65762 0.44442 1.0000
N10 N 4 0.22122 0.61191 0.43164 1.0000
N11 N 4 0.28909 0.33345 0.42414 1.0000
N12 N 4 0.23509 0.36595 0.47181 1.0000
N13 N 4 0.23911 0.29198 0.51079 1.0000
N14 N 4 0.29541 0.21423 0.48757 1.0000
N15 N 4 0.32645 0.24004 0.43359 1.0000
N16 N 4 0.22941 0.38787 0.25314 1.0000
N17 N 4 0.25403 0.30392 0.22707 1.0000
N18 N 4 0.18874 0.30662 0.17181 1.0000
N19 N 4 0.12408 0.39147 0.16374 1.0000
N20 N 4 0.14946 0.44208 0.21430 1.0000
Cd1 Cd 4 0.30999 0.43867 0.34659 1.0000
Cd2 Cd 4 0.82153 0.68330 0.39571 1.0000
#End

data_34-gis_Cd
_audit_creation_method TopoSPro
_Chemical_Name_Systematic EQOCOC01
_cell_length_a 19.28352
_cell_length_b 19.28352
_cell_length_c 17.36323
_cell_angle_alpha 56.26878
_cell_angle_beta 56.26878
_cell_angle_gamma 90
_cell_volume 3997.298
_cell_formula_units_Z 8
_symmetry_space_group_name_H-M 'P 1'
_symmetry_Int_Tables_number 1
loop_
_symmetry_equiv_pos_site_id
_symmetry_equiv_pos_as_xyz
1 x,y,z
loop_
_atom_site_label
_atom_site_type_symbol
_atom_site_symmetry_multiplicity
_atom_site_fract_x
_atom_site_fract_y
_atom_site_fract_z
_atom_site_occupancy
N1 N 1 0.34068 0.50000 0.00000 1.0000
N2 N 1 0.34068 0.00000 0.00000 1.0000
N3 N 1 0.00000 0.84068 0.50000 1.0000
N4 N 1 0.50000 0.15932 0.50000 1.0000
N5 N 1 0.65932 0.00000 0.00000 1.0000
N6 N 1 0.50000 0.84068 0.50000 1.0000
N7 N 1 0.00000 0.15932 0.50000 1.0000
N8 N 1 0.65932 0.50000 0.00000 1.0000
N9 N 1 0.22501 0.12610 0.24781 1.0000
N10 N 1 0.37390 0.97282 0.25219 1.0000
N11 N 1 0.62610 0.02718 0.74781 1.0000
N12 N 1 0.47282 0.87390 0.75219 1.0000
N13 N 1 0.52718 0.12610 0.24781 1.0000
N14 N 1 0.37390 0.27499 0.25219 1.0000
N15 N 1 0.77499 0.87390 0.75219 1.0000
N16 N 1 0.62610 0.72501 0.74781 1.0000
N17 N 1 0.14339 0.18210 0.20268 1.0000
N18 N 1 0.14339 0.11522 0.20268 1.0000
N19 N 1 0.31790 0.84607 0.29732 1.0000
N20 N 1 0.61522 0.15393 0.70268 1.0000
N21 N 1 0.34607 0.81790 0.79732 1.0000
N22 N 1 0.65393 0.11522 0.20268 1.0000
N23 N 1 0.38478 0.84607 0.29732 1.0000
N24 N 1 0.31790 0.35661 0.29732 1.0000
N25 N 1 0.38478 0.35661 0.29732 1.0000
N26 N 1 0.34607 0.88478 0.79732 1.0000
N27 N 1 0.85661 0.88478 0.79732 1.0000
N28 N 1 0.85661 0.81790 0.79732 1.0000
N29 N 1 0.68210 0.15393 0.70268 1.0000
N30 N 1 0.65393 0.18210 0.20268 1.0000
N31 N 1 0.61522 0.64339 0.70268 1.0000
N32 N 1 0.68210 0.64339 0.70268 1.0000
N33 N 1 0.25829 0.43083 0.08252 1.0000
N34 N 1 0.25829 0.98665 0.08252 1.0000
N35 N 1 0.06917 0.84081 0.41748 1.0000
N36 N 1 0.48665 0.15919 0.58252 1.0000
N37 N 1 0.34081 0.56917 0.91748 1.0000
N38 N 1 0.65919 0.98665 0.08252 1.0000
N39 N 1 0.51335 0.84081 0.41748 1.0000
N40 N 1 0.06917 0.24171 0.41748 1.0000
N41 N 1 0.51335 0.24171 0.41748 1.0000
N42 N 1 0.34081 0.01335 0.91748 1.0000
N43 N 1 0.74171 0.01335 0.91748 1.0000
N44 N 1 0.74171 0.56917 0.91748 1.0000
N45 N 1 0.93083 0.15919 0.58252 1.0000
N46 N 1 0.65919 0.43083 0.08252 1.0000
N47 N 1 0.48665 0.75829 0.58252 1.0000
N48 N 1 0.93083 0.75829 0.58252 1.0000
N49 N 1 0.19373 0.18858 0.23041 1.0000
N50 N 1 0.19373 0.08101 0.23041 1.0000
N51 N 1 0.31142 0.92413 0.26959 1.0000
N52 N 1 0.58101 0.07587 0.73041 1.0000

N12 N 8 0.20947 0.30484 0.43917 1.0000
N13 N 8 0.95489 0.13263 0.43020 1.0000
N14 N 8 0.88819 0.15306 0.38455 1.0000
N15 N 8 0.13528 0.10423 0.64539 1.0000
N16 N 8 0.96225 0.07222 0.36728 1.0000
N17 N 8 0.89275 0.11552 0.34546 1.0000
N18 N 8 0.13230 0.14346 0.60817 1.0000
N19 N 8 0.04163 0.12798 0.56739 1.0000
N20 N 8 0.04625 0.06484 0.62736 1.0000
Zn1 Zn 8 0.98421 0.17568 0.50234 1.0000
Zn2 Zn 8 0.99790 0.00198 0.32527 1.0000
#End

data_35-cha_Zn
_audit_creation_method ToposPro
_Chemical_Name_Systematic TOHDEB
_cell_length_a 17.18327
_cell_length_b 17.18327
_cell_length_c 17.18327
_cell_angle_alpha 104.8147
_cell_angle_beta 104.8147
_cell_angle_gamma 104.8147
_cell_volume 4453.318
_cell_formula_units_Z 12
_symmetry_space_group_name_H-M 'R -3 r'
_symmetry_Int_Tables_number 148
loop_
_symmetry_equiv_pos_site_id
_symmetry_equiv_pos_as_xyz
1 x,y,z
2 z,x,y
3 y,z,x
4 -x,-y,-z
5 -z,-x,-y
6 -y,-z,-x
loop_
_atom_site_label
_atom_site_type_symbol
_atom_site_symmetry_multiplicity
_atom_site_fract_x
_atom_site_fract_y
_atom_site_fract_z
_atom_site_occupancy
N1 N 6 0.10351 0.39546 0.02730 1.0000
N2 N 6 0.94960 0.14889 0.75236 1.0000
N3 N 6 0.02786 0.39593 0.10392 1.0000
N4 N 6 0.32127 0.24490 0.96493 1.0000
N5 N 6 0.05127 0.24774 0.85048 1.0000
N6 N 6 0.24493 0.32090 0.96490 1.0000
N7 N 6 0.03225 0.35111 0.03241 1.0000
N8 N 6 0.25864 0.25842 0.91508 1.0000
N9 N 6 0.96918 0.12109 0.81802 1.0000
N10 N 6 0.00023 0.22731 0.77212 1.0000
N11 N 6 0.09580 0.46778 0.14267 1.0000
N12 N 6 0.14285 0.46749 0.09500 1.0000
N13 N 6 0.29847 0.34562 0.04512 1.0000
N14 N 6 0.34587 0.29842 0.04515 1.0000
N15 N 6 0.03237 0.18241 0.87891 1.0000
N16 N 6 0.54793 0.11847 0.84569 1.0000
N17 N 6 0.44763 0.14882 0.87606 1.0000
N18 N 6 0.50225 0.11025 0.89499 1.0000
N19 N 6 0.45921 0.18062 0.81515 1.0000
N20 N 6 0.52171 0.16180 0.79632 1.0000
Zn1 Zn 6 0.35792 0.14416 0.92972 1.0000
Zn2 Zn 6 0.14447 0.35806 0.92961 1.0000
#End

data_36-sra_Zn
_audit_creation_method ToposPro
_Chemical_Name_Systematic BISPAU
_cell_length_a 9.685135
_cell_length_b 12.8143
_cell_length_c 20.07514
_cell_angle_alpha 90
_cell_angle_beta 96.33424
_cell_angle_gamma 90
_cell_volume 2476.279
_cell_formula_units_Z 8
_symmetry_space_group_name_H-M 'P 21/c'
_symmetry_Int_Tables_number 14
loop_
_symmetry_equiv_pos_site_id
_symmetry_equiv_pos_as_xyz
1 x,y,z
2 -x,1/2+y,1/2-z
3 -x,-y,-z
4 x,1/2-y,1/2+z
loop_
_atom_site_label
_atom_site_type_symbol
_atom_site_symmetry_multiplicity
_atom_site_fract_x
_atom_site_fract_y
_atom_site_fract_z
_atom_site_occupancy
N1 N 4 0.50352 0.48606 0.34488 1.0000
N2 N 4 0.55956 0.56697 0.37675 1.0000
N3 N 4 0.68568 0.57197 0.35986 1.0000
N4 N 4 0.70773 0.49472 0.31791 1.0000
N5 N 4 0.59449 0.44126 0.30855 1.0000
N6 N 4 0.19140 0.55304 0.37915 1.0000

N53 N 1 0.42413 0.81142 0.76959 1.0000
N54 N 1 0.57587 0.08101 0.23041 1.0000
N55 N 1 0.41899 0.92413 0.26959 1.0000
N56 N 1 0.31142 0.30627 0.26959 1.0000
N57 N 1 0.41899 0.30627 0.26959 1.0000
N58 N 1 0.42413 0.91899 0.76959 1.0000
N59 N 1 0.80627 0.91899 0.76959 1.0000
N60 N 1 0.80627 0.81142 0.76959 1.0000
N61 N 1 0.68858 0.07587 0.73041 1.0000
N62 N 1 0.57587 0.18858 0.23041 1.0000
N63 N 1 0.58101 0.69373 0.73041 1.0000
N64 N 1 0.68858 0.69373 0.73041 1.0000
N65 N 1 0.25926 0.54304 0.94866 1.0000
N66 N 1 0.25926 0.00831 0.94866 1.0000
N67 N 1 0.95696 0.70792 0.55134 1.0000
N68 N 1 0.50831 0.29208 0.44866 1.0000
N69 N 1 0.20792 0.45696 0.05134 1.0000
N70 N 1 0.79208 0.00831 0.94866 1.0000
N71 N 1 0.49169 0.70792 0.55134 1.0000
N72 N 1 0.95696 0.24074 0.55134 1.0000
N73 N 1 0.49169 0.24074 0.55134 1.0000
N74 N 1 0.20792 0.99169 0.05134 1.0000
N75 N 1 0.74074 0.99169 0.05134 1.0000
N76 N 1 0.74074 0.45696 0.05134 1.0000
N77 N 1 0.04304 0.29208 0.44866 1.0000
N78 N 1 0.79208 0.54304 0.94866 1.0000
N79 N 1 0.50831 0.75926 0.44866 1.0000
N80 N 1 0.04304 0.75926 0.44866 1.0000
Cd1 Cd 1 0.20978 0.29022 0.25000 1.0000
Cd2 Cd 1 0.20978 0.95978 0.25000 1.0000
Cd3 Cd 1 0.45978 0.04022 0.75000 1.0000
Cd4 Cd 1 0.45978 0.70978 0.75000 1.0000
Cd5 Cd 1 0.54022 0.95978 0.25000 1.0000
Cd6 Cd 1 0.54022 0.29022 0.25000 1.0000
Cd7 Cd 1 0.79022 0.04022 0.75000 1.0000
Cd8 Cd 1 0.79022 0.70978 0.75000 1.0000
#End

data_35-cha_Cd
_audit_creation_method ToposPro
_Chemical_Name_Systematic TOHDEB
_cell_length_a 18.39027
_cell_length_b 18.39027
_cell_length_c 18.39027
_cell_angle_alpha 104.7132
_cell_angle_beta 104.7132
_cell_angle_gamma 104.7132
_cell_volume 5470.853
_cell_formula_units_Z 12
_symmetry_space_group_name_H-M 'R -3 r'
_symmetry_Int_Tables_number 148
loop_
_symmetry_equiv_pos_site_id
_symmetry_equiv_pos_as_xyz
1 x,y,z
2 z,x,y
3 y,z,x
4 -x,-y,-z
5 -z,-x,-y
6 -y,-z,-x
loop_
_atom_site_label
_atom_site_type_symbol
_atom_site_symmetry_multiplicity
_atom_site_fract_x
_atom_site_fract_y
_atom_site_fract_z
_atom_site_occupancy
N1 N 6 0.10225 0.39754 0.03078 1.0000
N2 N 6 0.95363 0.15123 0.75715 1.0000
N3 N 6 0.03084 0.39746 0.10189 1.0000
N4 N 6 0.31896 0.24735 0.96676 1.0000
N5 N 6 0.04804 0.24331 0.84891 1.0000
N6 N 6 0.24791 0.31875 0.96686 1.0000
N7 N 6 0.03605 0.35555 0.03576 1.0000
N8 N 6 0.25891 0.25859 0.92098 1.0000
N9 N 6 0.97161 0.12511 0.81828 1.0000
N10 N 6 0.00076 0.22447 0.77566 1.0000
N11 N 6 0.09324 0.46500 0.13754 1.0000
N12 N 6 0.13771 0.46506 0.09325 1.0000
N13 N 6 0.30059 0.34443 0.04055 1.0000
N14 N 6 0.34481 0.29999 0.04050 1.0000
N15 N 6 0.03038 0.18237 0.87534 1.0000
N16 N 6 0.54604 0.12147 0.84809 1.0000
N17 N 6 0.45038 0.14753 0.87444 1.0000
N18 N 6 0.50264 0.11299 0.89353 1.0000
N19 N 6 0.46122 0.17707 0.81751 1.0000
N20 N 6 0.52087 0.16091 0.80115 1.0000
Cd1 Cd 6 0.35643 0.14318 0.92917 1.0000
Cd2 Cd 6 0.14422 0.35705 0.92936 1.0000
#End

data_36-crb3_Cd
_audit_creation_method ToposPro
_Chemical_Name_Systematic VEJYIT
_cell_length_a 10.10026
_cell_length_b 25.82835
_cell_length_c 24.94365
_cell_angle_alpha 90
_cell_angle_beta 90
_cell_angle_gamma 90

N7 N 4 0.05892 0.56307 0.36052 1.0000
N8 N 4 0.01457 0.62966 0.40247 1.0000
N9 N 4 0.11916 0.66059 0.44693 1.0000
N10 N 4 0.22901 0.61274 0.43253 1.0000
N11 N 4 0.29047 0.33587 0.42157 1.0000
N12 N 4 0.23381 0.36842 0.47375 1.0000
N13 N 4 0.23807 0.28919 0.51453 1.0000
N14 N 4 0.29708 0.20755 0.48788 1.0000
N15 N 4 0.32955 0.23665 0.43002 1.0000
N16 N 4 0.23183 0.38836 0.25687 1.0000
N17 N 4 0.25947 0.29960 0.22942 1.0000
N18 N 4 0.18935 0.30118 0.17010 1.0000
N19 N 4 0.11860 0.39036 0.16091 1.0000
N20 N 4 0.14514 0.44457 0.21478 1.0000
Zn1 Zn 4 0.30788 0.43870 0.34714 1.0000
Zn2 Zn 4 0.81985 0.68310 0.39530 1.0000
#End

data_37-ana_Zn
_audit_creation_method ToposPro
_Chemical_Name_Systematic EHETER02
_cell_length_a 22.31438
_cell_length_b 22.31438
_cell_length_c 22.31438
_cell_angle_alpha 109.4712
_cell_angle_beta 109.4712
_cell_angle_gamma 109.4712
_cell_volume 8553.274
_cell_formula_units_Z 24
_symmetry_space_group_name_H-M 'P 1'
_symmetry_Int_Tables_number 1
loop_
_symmetry_equiv_pos_site_id
_symmetry_equiv_pos_as_xyz
1 x,y,z
loop_
_atom_site_label
_atom_site_type_symbol
_atom_site_symmetry_multiplicity
_atom_site_fract_x
_atom_site_fract_y
_atom_site_fract_z
_atom_site_occupancy
N1 N 1 0.80846 0.07175 0.00579 1.0000
N2 N 1 0.69733 0.26330 0.69154 1.0000
N3 N 1 0.56597 0.80267 0.49421 1.0000
N4 N 1 0.26330 0.69154 0.69733 1.0000
N5 N 1 0.73670 0.92825 0.43403 1.0000
N6 N 1 0.57175 0.30846 0.50579 1.0000
N7 N 1 0.80846 0.23670 0.80267 1.0000
N8 N 1 0.76330 0.06597 0.57175 1.0000
N9 N 1 0.49421 0.42825 0.69154 1.0000
N10 N 1 0.56597 0.26330 0.07175 1.0000
N11 N 1 0.42825 0.23670 0.93403 1.0000
N12 N 1 0.57175 0.76330 0.06597 1.0000
N13 N 1 0.06597 0.57175 0.76330 1.0000
N14 N 1 0.99421 0.19154 0.92825 1.0000
N15 N 1 0.93403 0.00579 0.69733 1.0000
N16 N 1 0.19154 0.92825 0.99421 1.0000
N17 N 1 0.26330 0.07175 0.56597 1.0000
N18 N 1 0.00579 0.80846 0.07175 1.0000
N19 N 1 0.19154 0.76330 0.19733 1.0000
N20 N 1 0.07175 0.56597 0.26330 1.0000
N21 N 1 0.50579 0.57175 0.30846 1.0000
N22 N 1 0.49421 0.56597 0.80267 1.0000
N23 N 1 0.69733 0.93403 0.00579 1.0000
N24 N 1 0.99421 0.30267 0.06597 1.0000
N25 N 1 0.30267 0.73670 0.30846 1.0000
N26 N 1 0.19733 0.50579 0.43403 1.0000
N27 N 1 0.23670 0.93403 0.42825 1.0000
N28 N 1 0.73670 0.30846 0.30267 1.0000
N29 N 1 0.69154 0.49421 0.42825 1.0000
N30 N 1 0.30846 0.30267 0.73670 1.0000
N31 N 1 0.80267 0.49421 0.56597 1.0000
N32 N 1 0.80267 0.80846 0.23670 1.0000
N33 N 1 0.30267 0.06597 0.99421 1.0000
N34 N 1 0.43403 0.19733 0.50579 1.0000
N35 N 1 0.92825 0.99421 0.19154 1.0000
N36 N 1 0.76330 0.19733 0.19154 1.0000
N37 N 1 0.06597 0.99421 0.30267 1.0000
N38 N 1 0.00579 0.69733 0.93403 1.0000
N39 N 1 0.19733 0.19154 0.76330 1.0000
N40 N 1 0.42825 0.69154 0.49421 1.0000
N41 N 1 0.43403 0.73670 0.92825 1.0000
N42 N 1 0.07175 0.00579 0.80846 1.0000
N43 N 1 0.92825 0.43403 0.73670 1.0000
N44 N 1 0.69154 0.69733 0.26330 1.0000
N45 N 1 0.93403 0.42825 0.23670 1.0000
N46 N 1 0.23670 0.80267 0.80846 1.0000
N47 N 1 0.50579 0.43403 0.19733 1.0000
N48 N 1 0.30846 0.50579 0.57175 1.0000
N49 N 1 0.86168 0.13394 0.96931 1.0000
N50 N 1 0.60763 0.27226 0.63832 1.0000
N51 N 1 0.66463 0.89237 0.53069 1.0000
N52 N 1 0.27226 0.63832 0.60763 1.0000
N53 N 1 0.72774 0.86606 0.33537 1.0000
N54 N 1 0.63394 0.36168 0.46931 1.0000
N55 N 1 0.86168 0.22774 0.89237 1.0000
N56 N 1 0.77226 0.16463 0.63394 1.0000
N57 N 1 0.53069 0.36606 0.63832 1.0000
N58 N 1 0.66463 0.27226 0.13394 1.0000
_cell_volume 6507.125
_cell_formula_units_Z 16
_symmetry_space_group_name_H-M 'P b c a'
_symmetry_Int_Tables_number 61
loop_
_symmetry_equiv_pos_site_id
_symmetry_equiv_pos_as_xyz
1 x,y,z
2 1/2-x,-y,1/2+z
3 1/2+x,1/2-y,-z
4 -x,1/2+y,1/2-z
5 -x,-y,-z
6 1/2+x,y,1/2-z
7 1/2-x,1/2+y,z
8 x,1/2-y,1/2+z
loop_
_atom_site_label
_atom_site_type_symbol
_atom_site_symmetry_multiplicity
_atom_site_fract_x
_atom_site_fract_y
_atom_site_fract_z
_atom_site_occupancy
N1 N 8 0.12848 0.23773 0.48096 1.0000
N2 N 8 0.29261 0.28592 0.47826 1.0000
N3 N 8 0.99675 0.08498 0.41754 1.0000
N4 N 8 0.74530 0.01098 0.25078 1.0000
N5 N 8 0.10619 0.27261 0.44259 1.0000
N6 N 8 0.86733 0.99480 0.25446 1.0000
N7 N 8 0.90039 0.96776 0.21106 1.0000
N8 N 8 0.24370 0.24579 0.50315 1.0000
N9 N 8 0.70318 0.99388 0.20457 1.0000
N10 N 8 0.79825 0.96719 0.17997 1.0000
N11 N 8 0.99376 0.08140 0.58093 1.0000
N12 N 8 0.20827 0.30261 0.44096 1.0000
N13 N 8 0.95362 0.13165 0.42735 1.0000
N14 N 8 0.89093 0.15051 0.38461 1.0000
N15 N 8 0.13146 0.10417 0.64310 1.0000
N16 N 8 0.96016 0.07506 0.36837 1.0000
N17 N 8 0.89494 0.11529 0.34790 1.0000
N18 N 8 0.12857 0.14107 0.60827 1.0000
N19 N 8 0.04381 0.12687 0.57006 1.0000
N20 N 8 0.04842 0.06754 0.62605 1.0000
Cd1 Cd 8 0.98403 0.17656 0.50242 1.0000
Cd2 Cd 8 0.99762 0.00197 0.32530 1.0000
#End

data_37-ana_Cd
_audit_creation_method TopsPro
_Chemical_Name_Systematic EHETER02
_cell_length_a 23.86465
_cell_length_b 23.86465
_cell_length_c 23.86465
_cell_angle_alpha 109.4712
_cell_angle_beta 109.4712
_cell_angle_gamma 109.4712
_cell_volume 10462.69
_cell_formula_units_Z 24
_symmetry_space_group_name_H-M 'P 1'
_symmetry_Int_Tables_number 1
loop_
_symmetry_equiv_pos_site_id
_symmetry_equiv_pos_as_xyz
1 x,y,z
loop_
_atom_site_label
_atom_site_type_symbol
_atom_site_symmetry_multiplicity
_atom_site_fract_x
_atom_site_fract_y
_atom_site_fract_z
_atom_site_occupancy
N1 N 1 0.80883 0.07272 0.00300 1.0000
N2 N 1 0.69417 0.26389 0.69117 1.0000
N3 N 1 0.56972 0.80583 0.49700 1.0000
N4 N 1 0.26389 0.69117 0.69417 1.0000
N5 N 1 0.73611 0.92728 0.43028 1.0000
N6 N 1 0.57272 0.30883 0.50300 1.0000
N7 N 1 0.80883 0.23611 0.80583 1.0000
N8 N 1 0.76389 0.06972 0.57272 1.0000
N9 N 1 0.49700 0.42728 0.69117 1.0000
N10 N 1 0.56972 0.26389 0.07272 1.0000
N11 N 1 0.42728 0.23611 0.93028 1.0000
N12 N 1 0.57272 0.76389 0.06972 1.0000
N13 N 1 0.06972 0.57272 0.76389 1.0000
N14 N 1 0.99700 0.19117 0.92728 1.0000
N15 N 1 0.93028 0.00300 0.69417 1.0000
N16 N 1 0.19117 0.92728 0.99700 1.0000
N17 N 1 0.26389 0.07272 0.56972 1.0000
N18 N 1 0.00300 0.80883 0.07272 1.0000
N19 N 1 0.19117 0.76389 0.19417 1.0000
N20 N 1 0.07272 0.56972 0.26389 1.0000
N21 N 1 0.50300 0.57272 0.30883 1.0000
N22 N 1 0.49700 0.56972 0.80583 1.0000
N23 N 1 0.69417 0.93028 0.00300 1.0000
N24 N 1 0.99700 0.30583 0.06972 1.0000
N25 N 1 0.30583 0.73611 0.30883 1.0000
N26 N 1 0.19417 0.50300 0.43028 1.0000
N27 N 1 0.23611 0.93028 0.42728 1.0000
N28 N 1 0.73611 0.30883 0.30583 1.0000
N29 N 1 0.69117 0.49700 0.42728 1.0000

N59 N 1 0.36606 0.22774 0.83537 1.0000	N30 N 1 0.30883 0.30583 0.73611 1.0000
N60 N 1 0.63394 0.77226 0.16463 1.0000	N31 N 1 0.80583 0.49700 0.56972 1.0000
N61 N 1 0.16463 0.63394 0.77226 1.0000	N32 N 1 0.80583 0.80883 0.23611 1.0000
N62 N 1 0.03069 0.13832 0.86606 1.0000	N33 N 1 0.30583 0.06972 0.99700 1.0000
N63 N 1 0.83537 0.96931 0.60763 1.0000	N34 N 1 0.43028 0.19417 0.50300 1.0000
N64 N 1 0.13832 0.86606 0.03069 1.0000	N35 N 1 0.92728 0.99700 0.19117 1.0000
N65 N 1 0.27226 0.13394 0.66463 1.0000	N36 N 1 0.76389 0.19417 0.19117 1.0000
N66 N 1 0.96931 0.86168 0.13394 1.0000	N37 N 1 0.06972 0.99700 0.30583 1.0000
N67 N 1 0.13832 0.77226 0.10763 1.0000	N38 N 1 0.00300 0.69417 0.93028 1.0000
N68 N 1 0.13394 0.66463 0.27226 1.0000	N39 N 1 0.19417 0.19117 0.76389 1.0000
N69 N 1 0.46931 0.63394 0.36168 1.0000	N40 N 1 0.42728 0.69117 0.49700 1.0000
N70 N 1 0.53069 0.66463 0.89237 1.0000	N41 N 1 0.43028 0.73611 0.92728 1.0000
N71 N 1 0.60763 0.83537 0.96931 1.0000	N42 N 1 0.07272 0.00300 0.80883 1.0000
N72 N 1 0.03069 0.39237 0.16463 1.0000	N43 N 1 0.92728 0.43028 0.73611 1.0000
N73 N 1 0.39237 0.72774 0.36168 1.0000	N44 N 1 0.69117 0.69417 0.26389 1.0000
N74 N 1 0.10763 0.46931 0.33537 1.0000	N45 N 1 0.93028 0.42728 0.23611 1.0000
N75 N 1 0.22774 0.83537 0.36606 1.0000	N46 N 1 0.23611 0.80583 0.80883 1.0000
N76 N 1 0.72774 0.36168 0.39237 1.0000	N47 N 1 0.50300 0.43028 0.19417 1.0000
N77 N 1 0.63832 0.53069 0.36606 1.0000	N48 N 1 0.30883 0.50300 0.57272 1.0000
N78 N 1 0.36168 0.39237 0.72774 1.0000	N49 N 1 0.85871 0.13089 0.96887 1.0000
N79 N 1 0.89237 0.53069 0.66463 1.0000	N50 N 1 0.61016 0.27218 0.64129 1.0000
N80 N 1 0.89237 0.86168 0.22774 1.0000	N51 N 1 0.66202 0.88984 0.53113 1.0000
N81 N 1 0.39237 0.16463 0.03069 1.0000	N52 N 1 0.27218 0.64129 0.61016 1.0000
N82 N 1 0.33537 0.10763 0.46931 1.0000	N53 N 1 0.72782 0.86911 0.33798 1.0000
N83 N 1 0.86606 0.03069 0.13832 1.0000	N54 N 1 0.63089 0.35871 0.46887 1.0000
N84 N 1 0.77226 0.10763 0.13832 1.0000	N55 N 1 0.85871 0.22782 0.88984 1.0000
N85 N 1 0.16463 0.03069 0.39237 1.0000	N56 N 1 0.77218 0.16202 0.63089 1.0000
N86 N 1 0.96931 0.60763 0.83537 1.0000	N57 N 1 0.53113 0.36911 0.64129 1.0000
N87 N 1 0.10763 0.13832 0.77226 1.0000	N58 N 1 0.66202 0.27218 0.13089 1.0000
N88 N 1 0.36606 0.63832 0.53069 1.0000	N59 N 1 0.36911 0.22782 0.83798 1.0000
N89 N 1 0.33537 0.72774 0.86606 1.0000	N60 N 1 0.63089 0.77218 0.16202 1.0000
N90 N 1 0.13394 0.96931 0.86168 1.0000	N61 N 1 0.16202 0.63089 0.77218 1.0000
N91 N 1 0.86606 0.33537 0.72774 1.0000	N62 N 1 0.03113 0.14129 0.86911 1.0000
N92 N 1 0.63832 0.60763 0.27226 1.0000	N63 N 1 0.83798 0.96887 0.61016 1.0000
N93 N 1 0.83537 0.36606 0.22774 1.0000	N64 N 1 0.14129 0.86911 0.03113 1.0000
N94 N 1 0.22774 0.89237 0.86168 1.0000	N65 N 1 0.27218 0.13089 0.66202 1.0000
N95 N 1 0.46931 0.33537 0.10763 1.0000	N66 N 1 0.96887 0.85871 0.13089 1.0000
N96 N 1 0.36168 0.46931 0.63394 1.0000	N67 N 1 0.14129 0.77218 0.11016 1.0000
N97 N 1 0.79599 0.08052 0.91171 1.0000	N68 N 1 0.13089 0.66202 0.27218 1.0000
N98 N 1 0.61572 0.28453 0.70401 1.0000	N69 N 1 0.46887 0.63089 0.35871 1.0000
N99 N 1 0.66881 0.88428 0.58829 1.0000	N70 N 1 0.53113 0.66202 0.88984 1.0000
N100 N 1 0.28453 0.70401 0.61572 1.0000	N71 N 1 0.61016 0.83798 0.96887 1.0000
N101 N 1 0.71547 0.91948 0.33119 1.0000	N72 N 1 0.03113 0.38984 0.16202 1.0000
N102 N 1 0.58052 0.29599 0.41171 1.0000	N73 N 1 0.38984 0.72782 0.35871 1.0000
N103 N 1 0.79599 0.21547 0.88428 1.0000	N74 N 1 0.11016 0.46887 0.33798 1.0000
N104 N 1 0.78453 0.16881 0.58052 1.0000	N75 N 1 0.22782 0.83798 0.36911 1.0000
N105 N 1 0.58829 0.41948 0.70401 1.0000	N76 N 1 0.72782 0.35871 0.38984 1.0000
N106 N 1 0.66881 0.28453 0.08052 1.0000	N77 N 1 0.64129 0.53113 0.36911 1.0000
N107 N 1 0.41948 0.21547 0.83119 1.0000	N78 N 1 0.35871 0.38984 0.72782 1.0000
N108 N 1 0.58052 0.78453 0.16881 1.0000	N79 N 1 0.88984 0.53113 0.66202 1.0000
N109 N 1 0.16881 0.58052 0.78453 1.0000	N80 N 1 0.88984 0.85871 0.22782 1.0000
N110 N 1 0.08829 0.20401 0.91948 1.0000	N81 N 1 0.38984 0.16202 0.03113 1.0000
N111 N 1 0.83119 0.91171 0.61572 1.0000	N82 N 1 0.33798 0.11016 0.46887 1.0000
N112 N 1 0.20401 0.91948 0.08829 1.0000	N83 N 1 0.86911 0.03113 0.14129 1.0000
N113 N 1 0.28453 0.08052 0.66881 1.0000	N84 N 1 0.77218 0.11016 0.14129 1.0000
N114 N 1 0.91171 0.79599 0.08052 1.0000	N85 N 1 0.16202 0.03113 0.38984 1.0000
N115 N 1 0.20401 0.78453 0.11572 1.0000	N86 N 1 0.96887 0.61016 0.83798 1.0000
N116 N 1 0.08052 0.66881 0.28453 1.0000	N87 N 1 0.11016 0.14129 0.77218 1.0000
N117 N 1 0.41171 0.58052 0.29599 1.0000	N88 N 1 0.36911 0.64129 0.53113 1.0000
N118 N 1 0.58829 0.66881 0.88428 1.0000	N89 N 1 0.33798 0.72782 0.86911 1.0000
N119 N 1 0.61572 0.83119 0.91171 1.0000	N90 N 1 0.13089 0.96887 0.85871 1.0000
N120 N 1 0.08829 0.38428 0.16881 1.0000	N91 N 1 0.86911 0.33798 0.72782 1.0000
N121 N 1 0.38428 0.71547 0.29599 1.0000	N92 N 1 0.64129 0.61016 0.27218 1.0000
N122 N 1 0.11572 0.41171 0.33119 1.0000	N93 N 1 0.83798 0.36911 0.22782 1.0000
N123 N 1 0.21547 0.83119 0.41948 1.0000	N94 N 1 0.22782 0.88984 0.85871 1.0000
N124 N 1 0.71547 0.29599 0.38428 1.0000	N95 N 1 0.46887 0.33798 0.11016 1.0000
N125 N 1 0.70401 0.58829 0.41948 1.0000	N96 N 1 0.35871 0.46887 0.63089 1.0000
N126 N 1 0.29599 0.38428 0.71547 1.0000	N97 N 1 0.79736 0.08108 0.91509 1.0000
N127 N 1 0.88428 0.58829 0.66881 1.0000	N98 N 1 0.61773 0.28373 0.70264 1.0000
N128 N 1 0.88428 0.79599 0.21547 1.0000	N99 N 1 0.66599 0.88227 0.58491 1.0000
N129 N 1 0.38428 0.16881 0.08829 1.0000	N100 N 1 0.28373 0.70264 0.61773 1.0000
N130 N 1 0.33119 0.11572 0.41171 1.0000	N101 N 1 0.71627 0.91892 0.33401 1.0000
N131 N 1 0.91948 0.08829 0.20401 1.0000	N102 N 1 0.58108 0.29736 0.41509 1.0000
N132 N 1 0.78453 0.11572 0.20401 1.0000	N103 N 1 0.79736 0.21627 0.88227 1.0000
N133 N 1 0.16881 0.08829 0.38428 1.0000	N104 N 1 0.78373 0.16599 0.58108 1.0000
N134 N 1 0.91171 0.61572 0.83119 1.0000	N105 N 1 0.58491 0.41892 0.70264 1.0000
N135 N 1 0.11572 0.20401 0.78453 1.0000	N106 N 1 0.66599 0.28373 0.08108 1.0000
N136 N 1 0.41948 0.70401 0.58829 1.0000	N107 N 1 0.41892 0.21627 0.83401 1.0000
N137 N 1 0.33119 0.71547 0.91948 1.0000	N108 N 1 0.58108 0.78373 0.16599 1.0000
N138 N 1 0.08052 0.91171 0.79599 1.0000	N109 N 1 0.16599 0.58108 0.78373 1.0000
N139 N 1 0.91948 0.33119 0.71547 1.0000	N110 N 1 0.08491 0.20264 0.91892 1.0000
N140 N 1 0.70401 0.61572 0.28453 1.0000	N111 N 1 0.83401 0.91509 0.61773 1.0000
N141 N 1 0.83119 0.41948 0.21547 1.0000	N112 N 1 0.20264 0.91892 0.08491 1.0000
N142 N 1 0.21547 0.88428 0.79599 1.0000	N113 N 1 0.28373 0.08108 0.66599 1.0000
N143 N 1 0.41171 0.33119 0.11572 1.0000	N114 N 1 0.91509 0.79736 0.08108 1.0000
N144 N 1 0.29599 0.41171 0.58052 1.0000	N115 N 1 0.20264 0.78373 0.11773 1.0000
N145 N 1 0.86965 0.12880 0.02768 1.0000	N116 N 1 0.08108 0.66599 0.28373 1.0000
N146 N 1 0.65803 0.25915 0.63035 1.0000	N117 N 1 0.41509 0.58108 0.29736 1.0000
N147 N 1 0.60112 0.84197 0.47232 1.0000	N118 N 1 0.58491 0.66599 0.88227 1.0000
N148 N 1 0.25915 0.63035 0.65803 1.0000	N119 N 1 0.61773 0.83401 0.91509 1.0000
N149 N 1 0.74085 0.87120 0.39888 1.0000	N120 N 1 0.08491 0.38227 0.16599 1.0000
N150 N 1 0.62880 0.36965 0.52768 1.0000	N121 N 1 0.38227 0.71627 0.29736 1.0000
N151 N 1 0.86965 0.24085 0.84197 1.0000	N122 N 1 0.11773 0.41509 0.33401 1.0000
N152 N 1 0.75915 0.10112 0.62880 1.0000	N123 N 1 0.21627 0.83401 0.41892 1.0000
N153 N 1 0.47232 0.37120 0.63035 1.0000	N124 N 1 0.71627 0.29736 0.38227 1.0000
N154 N 1 0.60112 0.25915 0.12880 1.0000	N125 N 1 0.70264 0.58491 0.41892 1.0000
N155 N 1 0.37120 0.24085 0.89888 1.0000	N126 N 1 0.29736 0.38227 0.71627 1.0000
N156 N 1 0.62880 0.75915 0.10112 1.0000	N127 N 1 0.88227 0.58491 0.66599 1.0000
N157 N 1 0.10112 0.62880 0.75915 1.0000	N128 N 1 0.88227 0.79736 0.21627 1.0000
N158 N 1 0.97232 0.13035 0.87120 1.0000	N129 N 1 0.38227 0.16599 0.08491 1.0000
N159 N 1 0.89888 0.02768 0.65803 1.0000	N130 N 1 0.33401 0.11773 0.41509 1.0000

N160 N 1 0.13035 0.87120 0.97232 1.0000
N161 N 1 0.25915 0.12880 0.60112 1.0000
N162 N 1 0.02768 0.86965 0.12880 1.0000
N163 N 1 0.13035 0.75915 0.15803 1.0000
N164 N 1 0.12880 0.60112 0.25915 1.0000
N165 N 1 0.52768 0.62880 0.36965 1.0000
N166 N 1 0.47232 0.60112 0.84197 1.0000
N167 N 1 0.65803 0.89888 0.02768 1.0000
N168 N 1 0.97232 0.34197 0.10112 1.0000
N169 N 1 0.34197 0.74085 0.36965 1.0000
N170 N 1 0.15803 0.52768 0.39888 1.0000
N171 N 1 0.24085 0.89888 0.37120 1.0000
N172 N 1 0.74085 0.36965 0.34197 1.0000
N173 N 1 0.63035 0.47232 0.37120 1.0000
N174 N 1 0.36965 0.34197 0.74085 1.0000
N175 N 1 0.84197 0.47232 0.60112 1.0000
N176 N 1 0.84197 0.86965 0.24085 1.0000
N177 N 1 0.34197 0.10112 0.97232 1.0000
N178 N 1 0.39888 0.15803 0.52768 1.0000
N179 N 1 0.87120 0.97232 0.13035 1.0000
N180 N 1 0.75915 0.15803 0.13035 1.0000
N181 N 1 0.10112 0.97232 0.34197 1.0000
N182 N 1 0.02768 0.65803 0.89888 1.0000
N183 N 1 0.15803 0.13035 0.75915 1.0000
N184 N 1 0.37120 0.63035 0.47232 1.0000
N185 N 1 0.39888 0.74085 0.87120 1.0000
N186 N 1 0.12880 0.02768 0.86965 1.0000
N187 N 1 0.87120 0.39888 0.74085 1.0000
N188 N 1 0.63035 0.65803 0.25915 1.0000
N189 N 1 0.89888 0.37120 0.24085 1.0000
N190 N 1 0.24085 0.84197 0.86965 1.0000
N191 N 1 0.52768 0.39888 0.15803 1.0000
N192 N 1 0.36965 0.52768 0.62880 1.0000
N193 N 1 0.76288 0.04198 0.93432 1.0000
N194 N 1 0.67144 0.27910 0.73712 1.0000
N195 N 1 0.60766 0.82856 0.56568 1.0000
N196 N 1 0.27910 0.73712 0.67144 1.0000
N197 N 1 0.72090 0.95802 0.39234 1.0000
N198 N 1 0.54198 0.26288 0.43432 1.0000
N199 N 1 0.76288 0.22090 0.82856 1.0000
N200 N 1 0.77910 0.10766 0.54198 1.0000
N201 N 1 0.56568 0.45802 0.73712 1.0000
N202 N 1 0.60766 0.27910 0.04198 1.0000
N203 N 1 0.45802 0.22090 0.89234 1.0000
N204 N 1 0.54198 0.77910 0.10766 1.0000
N205 N 1 0.10766 0.54198 0.77910 1.0000
N206 N 1 0.06568 0.23712 0.95802 1.0000
N207 N 1 0.89234 0.93432 0.67144 1.0000
N208 N 1 0.23712 0.95802 0.06568 1.0000
N209 N 1 0.27910 0.04198 0.60766 1.0000
N210 N 1 0.93432 0.76288 0.04198 1.0000
N211 N 1 0.23712 0.77910 0.17144 1.0000
N212 N 1 0.04198 0.60766 0.27910 1.0000
N213 N 1 0.43432 0.54198 0.26288 1.0000
N214 N 1 0.56568 0.60766 0.82856 1.0000
N215 N 1 0.67144 0.89234 0.93432 1.0000
N216 N 1 0.06568 0.32856 0.10766 1.0000
N217 N 1 0.32856 0.72090 0.26288 1.0000
N218 N 1 0.17144 0.43432 0.39234 1.0000
N219 N 1 0.22090 0.89234 0.45802 1.0000
N220 N 1 0.72090 0.26288 0.32856 1.0000
N221 N 1 0.73712 0.56568 0.45802 1.0000
N222 N 1 0.26288 0.32856 0.72090 1.0000
N223 N 1 0.82856 0.56568 0.60766 1.0000
N224 N 1 0.82856 0.76288 0.22090 1.0000
N225 N 1 0.32856 0.10766 0.06568 1.0000
N226 N 1 0.39234 0.17144 0.43432 1.0000
N227 N 1 0.95802 0.06568 0.23712 1.0000
N228 N 1 0.77910 0.17144 0.23712 1.0000
N229 N 1 0.10766 0.06568 0.32856 1.0000
N230 N 1 0.93432 0.67144 0.89234 1.0000
N231 N 1 0.17144 0.23712 0.77910 1.0000
N232 N 1 0.45802 0.73712 0.56568 1.0000
N233 N 1 0.39234 0.72090 0.95802 1.0000
N234 N 1 0.04198 0.93432 0.76288 1.0000
N235 N 1 0.95802 0.39234 0.72090 1.0000
N236 N 1 0.73712 0.67144 0.27910 1.0000
N237 N 1 0.89234 0.45802 0.22090 1.0000
N238 N 1 0.22090 0.82856 0.76288 1.0000
N239 N 1 0.43432 0.39234 0.17144 1.0000
N240 N 1 0.26288 0.43432 0.54198 1.0000
Zn1 Zn 1 0.93050 0.21525 0.96525 1.0000
Zn2 Zn 1 0.53475 0.28475 0.56950 1.0000
Zn3 Zn 1 0.75000 0.96525 0.53475 1.0000
Zn4 Zn 1 0.28475 0.56950 0.53475 1.0000
Zn5 Zn 1 0.71525 0.78475 0.25000 1.0000
Zn6 Zn 1 0.71525 0.43050 0.46525 1.0000
Zn7 Zn 1 0.78475 0.25000 0.71525 1.0000
Zn8 Zn 1 0.75000 0.28475 0.21525 1.0000
Zn9 Zn 1 0.28475 0.21525 0.75000 1.0000
Zn10 Zn 1 0.25000 0.71525 0.78475 1.0000
Zn11 Zn 1 0.03475 0.06950 0.78475 1.0000
Zn12 Zn 1 0.06950 0.78475 0.03475 1.0000
Zn13 Zn 1 0.96525 0.93050 0.21525 1.0000
Zn14 Zn 1 0.21525 0.75000 0.28475 1.0000
Zn15 Zn 1 0.46525 0.71525 0.43050 1.0000
Zn16 Zn 1 0.53475 0.75000 0.96525 1.0000
Zn17 Zn 1 0.03475 0.46525 0.25000 1.0000
Zn18 Zn 1 0.56950 0.53475 0.28475 1.0000
Zn19 Zn 1 0.43050 0.46525 0.71525 1.0000
Zn20 Zn 1 0.96525 0.53475 0.75000 1.0000

N131 N 1 0.91892 0.08491 0.20264 1.0000
N132 N 1 0.78373 0.11773 0.20264 1.0000
N133 N 1 0.16599 0.08491 0.38227 1.0000
N134 N 1 0.91509 0.61773 0.83401 1.0000
N135 N 1 0.11773 0.20264 0.78373 1.0000
N136 N 1 0.41892 0.70264 0.58491 1.0000
N137 N 1 0.33401 0.71627 0.91892 1.0000
N138 N 1 0.08108 0.91509 0.79736 1.0000
N139 N 1 0.91892 0.33401 0.71627 1.0000
N140 N 1 0.70264 0.61773 0.28373 1.0000
N141 N 1 0.83401 0.41892 0.21627 1.0000
N142 N 1 0.21627 0.88227 0.79736 1.0000
N143 N 1 0.41509 0.33401 0.11773 1.0000
N144 N 1 0.29736 0.41509 0.58108 1.0000
N145 N 1 0.86612 0.12604 0.02348 1.0000
N146 N 1 0.65736 0.25992 0.63388 1.0000
N147 N 1 0.60256 0.84264 0.47652 1.0000
N148 N 1 0.25992 0.63388 0.65736 1.0000
N149 N 1 0.74008 0.87396 0.39744 1.0000
N150 N 1 0.62604 0.36612 0.52348 1.0000
N151 N 1 0.86612 0.24008 0.84264 1.0000
N152 N 1 0.75992 0.10256 0.62604 1.0000
N153 N 1 0.47652 0.37396 0.63388 1.0000
N154 N 1 0.60256 0.25992 0.12604 1.0000
N155 N 1 0.37396 0.24008 0.89744 1.0000
N156 N 1 0.62604 0.75992 0.10256 1.0000
N157 N 1 0.10256 0.62604 0.75992 1.0000
N158 N 1 0.97652 0.13388 0.87396 1.0000
N159 N 1 0.89744 0.02348 0.65736 1.0000
N160 N 1 0.13388 0.87396 0.97652 1.0000
N161 N 1 0.25992 0.12604 0.60256 1.0000
N162 N 1 0.02348 0.86612 0.12604 1.0000
N163 N 1 0.13388 0.75992 0.15736 1.0000
N164 N 1 0.12604 0.60256 0.25992 1.0000
N165 N 1 0.52348 0.62604 0.36612 1.0000
N166 N 1 0.47652 0.60256 0.84264 1.0000
N167 N 1 0.65736 0.89744 0.02348 1.0000
N168 N 1 0.97652 0.34264 0.10256 1.0000
N169 N 1 0.34264 0.74008 0.36612 1.0000
N170 N 1 0.15736 0.52348 0.39744 1.0000
N171 N 1 0.24008 0.89744 0.37396 1.0000
N172 N 1 0.74008 0.36612 0.34264 1.0000
N173 N 1 0.63388 0.47652 0.37396 1.0000
N174 N 1 0.36612 0.34264 0.74008 1.0000
N175 N 1 0.84264 0.47652 0.60256 1.0000
N176 N 1 0.84264 0.86612 0.24008 1.0000
N177 N 1 0.34264 0.10256 0.97652 1.0000
N178 N 1 0.39744 0.15736 0.52348 1.0000
N179 N 1 0.87396 0.97652 0.13388 1.0000
N180 N 1 0.75992 0.15736 0.13388 1.0000
N181 N 1 0.10256 0.97652 0.34264 1.0000
N182 N 1 0.02348 0.65736 0.89744 1.0000
N183 N 1 0.15736 0.13388 0.75992 1.0000
N184 N 1 0.37396 0.63388 0.47652 1.0000
N185 N 1 0.39744 0.74008 0.87396 1.0000
N186 N 1 0.12604 0.02348 0.86612 1.0000
N187 N 1 0.87396 0.39744 0.74008 1.0000
N188 N 1 0.63388 0.65736 0.25992 1.0000
N189 N 1 0.89744 0.37396 0.24008 1.0000
N190 N 1 0.24008 0.84264 0.86612 1.0000
N191 N 1 0.52348 0.39744 0.15736 1.0000
N192 N 1 0.36612 0.52348 0.62604 1.0000
N193 N 1 0.76630 0.04490 0.93630 1.0000
N194 N 1 0.67000 0.27859 0.73370 1.0000
N195 N 1 0.60860 0.83000 0.56370 1.0000
N196 N 1 0.27859 0.73370 0.67000 1.0000
N197 N 1 0.72141 0.95510 0.39140 1.0000
N198 N 1 0.54490 0.26630 0.43630 1.0000
N199 N 1 0.76630 0.22141 0.83000 1.0000
N200 N 1 0.77859 0.10860 0.54490 1.0000
N201 N 1 0.56370 0.45510 0.73370 1.0000
N202 N 1 0.60860 0.27859 0.04490 1.0000
N203 N 1 0.45510 0.22141 0.89140 1.0000
N204 N 1 0.54490 0.77859 0.10860 1.0000
N205 N 1 0.10860 0.54490 0.77859 1.0000
N206 N 1 0.06370 0.23370 0.95510 1.0000
N207 N 1 0.89140 0.93630 0.67000 1.0000
N208 N 1 0.23370 0.95510 0.06370 1.0000
N209 N 1 0.27859 0.04490 0.60860 1.0000
N210 N 1 0.93630 0.76630 0.04490 1.0000
N211 N 1 0.23370 0.77859 0.17000 1.0000
N212 N 1 0.04490 0.60860 0.27859 1.0000
N213 N 1 0.43630 0.54490 0.26630 1.0000
N214 N 1 0.56370 0.60860 0.83000 1.0000
N215 N 1 0.67000 0.89140 0.93630 1.0000
N216 N 1 0.06370 0.33000 0.10860 1.0000
N217 N 1 0.33000 0.72141 0.26630 1.0000
N218 N 1 0.17000 0.43630 0.39140 1.0000
N219 N 1 0.22141 0.89140 0.45510 1.0000
N220 N 1 0.72141 0.26630 0.33000 1.0000
N221 N 1 0.73370 0.56370 0.45510 1.0000
N222 N 1 0.26630 0.33000 0.72141 1.0000
N223 N 1 0.83000 0.56370 0.60860 1.0000
N224 N 1 0.83000 0.76630 0.22141 1.0000
N225 N 1 0.33000 0.10860 0.06370 1.0000
N226 N 1 0.39140 0.17000 0.43630 1.0000
N227 N 1 0.95510 0.06370 0.23370 1.0000
N228 N 1 0.77859 0.17000 0.23370 1.0000
N229 N 1 0.10860 0.06370 0.33000 1.0000
N230 N 1 0.93630 0.67000 0.89140 1.0000
N231 N 1 0.17000 0.23370 0.77859 1.0000

Zn21 Zn 1 0.46525 0.25000 0.03475 1.0000
Zn22 Zn 1 0.25000 0.03475 0.46525 1.0000
Zn23 Zn 1 0.78475 0.03475 0.06950 1.0000
Zn24 Zn 1 0.21525 0.96525 0.93050 1.0000
#End

data_38-qtz_Zn
_audit_creation_method ToposPro
_Chemical_Name_Systematic CAGLIF
_cell_length_a 8.913351
_cell_length_b 8.913351
_cell_length_c 11.63692
_cell_angle_alpha 90
_cell_angle_beta 90
_cell_angle_gamma 120
_cell_volume 800.6649
_cell_formula_units_Z 3
_symmetry_space_group_name_H-M 'P 64 2 2'
_symmetry_Int_Tables_number 181
loop_
_symmetry_equiv_pos_site_id
_symmetry_equiv_pos_as_xyz
1 x,y,z
2 -x,-y,z
3 -y,x-y,1/3+z
4 -x+y,-x,2/3+z
5 x-y,x,2/3+z
6 y,-x+y,1/3+z
7 x-y,-y,-z
8 -x,-x+y,2/3-z
9 y,x,1/3-z
10 -y,-x,1/3-z
11 x,x-y,2/3-z
12 -x+y,y,-z
loop_
_atom_site_label
_atom_site_type_symbol
_atom_site_symmetry_multiplicity
_atom_site_fract_x
_atom_site_fract_y
_atom_site_fract_z
_atom_site_occupancy
N1 N 12 0.87041 0.29854 0.22784 1.0000
N2 N 6 0.73459 0.26541 0.16667 1.0000
N3 N 12 0.92120 0.18405 0.20447 1.0000
Zn1 Zn 3 0.00000 0.50000 0.33333 1.0000
#End

data_39-rho_Zn
_audit_creation_method ToposPro
_Chemical_Name_Systematic MECWOH
_cell_length_a 24.69465
_cell_length_b 24.69465
_cell_length_c 24.69465
_cell_angle_alpha 109.4712
_cell_angle_beta 109.4712
_cell_angle_gamma 109.4712
_cell_volume 11592.75
_cell_formula_units_Z 24
_symmetry_space_group_name_H-M 'P 1'
_symmetry_Int_Tables_number 1
loop_
_symmetry_equiv_pos_site_id
_symmetry_equiv_pos_as_xyz
1 x,y,z
loop_
_atom_site_label
_atom_site_type_symbol
_atom_site_symmetry_multiplicity
_atom_site_fract_x
_atom_site_fract_y
_atom_site_fract_z
_atom_site_occupancy
N1 N 1 0.42055 0.27018 0.61806 1.0000
N2 N 1 0.84963 0.27018 0.65212 1.0000
N3 N 1 0.42055 0.61806 0.27018 1.0000
N4 N 1 0.34788 0.72982 0.15037 1.0000
N5 N 1 0.61806 0.27018 0.42055 1.0000
N6 N 1 0.84963 0.19751 0.57945 1.0000
N7 N 1 0.57945 0.84963 0.19751 1.0000
N8 N 1 0.80249 0.38194 0.65212 1.0000
N9 N 1 0.72982 0.34788 0.15037 1.0000
N10 N 1 0.72982 0.57945 0.38194 1.0000
N11 N 1 0.61806 0.42055 0.27018 1.0000
N12 N 1 0.61806 0.19751 0.34788 1.0000
N13 N 1 0.15037 0.42055 0.80249 1.0000
N14 N 1 0.27018 0.42055 0.61806 1.0000
N15 N 1 0.80249 0.42055 0.15037 1.0000
N16 N 1 0.27018 0.61806 0.42055 1.0000
N17 N 1 0.42055 0.15037 0.80249 1.0000
N18 N 1 0.15037 0.72982 0.34788 1.0000
N19 N 1 0.72982 0.38194 0.57945 1.0000
N20 N 1 0.57945 0.38194 0.72982 1.0000
N21 N 1 0.65212 0.38194 0.80249 1.0000
N22 N 1 0.34788 0.19751 0.61806 1.0000
N23 N 1 0.57945 0.19751 0.84963 1.0000
N24 N 1 0.57945 0.72982 0.38194 1.0000
N25 N 1 0.65212 0.84963 0.27018 1.0000
N26 N 1 0.38194 0.65212 0.80249 1.0000
N27 N 1 0.15037 0.34788 0.72982 1.0000
N28 N 1 0.27018 0.84963 0.65212 1.0000
N232 N 1 0.45510 0.73370 0.56370 1.0000
N233 N 1 0.39140 0.72141 0.95510 1.0000
N234 N 1 0.04490 0.93630 0.76630 1.0000
N235 N 1 0.95510 0.39140 0.72141 1.0000
N236 N 1 0.73370 0.67000 0.27859 1.0000
N237 N 1 0.89140 0.45510 0.22141 1.0000
N238 N 1 0.22141 0.83000 0.76630 1.0000
N239 N 1 0.43630 0.39140 0.17000 1.0000
N240 N 1 0.26630 0.43630 0.54490 1.0000
Cd1 Cd 1 0.93081 0.21540 0.96540 1.0000
Cd2 Cd 1 0.53460 0.28460 0.56919 1.0000
Cd3 Cd 1 0.75000 0.96540 0.53460 1.0000
Cd4 Cd 1 0.28460 0.56919 0.53460 1.0000
Cd5 Cd 1 0.71540 0.78460 0.25000 1.0000
Cd6 Cd 1 0.71540 0.43081 0.46540 1.0000
Cd7 Cd 1 0.78460 0.25000 0.71540 1.0000
Cd8 Cd 1 0.75000 0.28460 0.21540 1.0000
Cd9 Cd 1 0.28460 0.21540 0.75000 1.0000
Cd10 Cd 1 0.25000 0.71540 0.78460 1.0000
Cd11 Cd 1 0.03460 0.06919 0.78460 1.0000
Cd12 Cd 1 0.06919 0.78460 0.03460 1.0000
Cd13 Cd 1 0.96540 0.93081 0.21540 1.0000
Cd14 Cd 1 0.21540 0.75000 0.28460 1.0000
Cd15 Cd 1 0.46540 0.71540 0.43081 1.0000
Cd16 Cd 1 0.53460 0.75000 0.96540 1.0000
Cd17 Cd 1 0.03460 0.46540 0.25000 1.0000
Cd18 Cd 1 0.56919 0.53460 0.28460 1.0000
Cd19 Cd 1 0.43081 0.46540 0.71540 1.0000
Cd20 Cd 1 0.96540 0.53460 0.75000 1.0000
Cd21 Cd 1 0.46540 0.25000 0.03460 1.0000
Cd22 Cd 1 0.25000 0.03460 0.46540 1.0000
Cd23 Cd 1 0.78460 0.03460 0.06919 1.0000
Cd24 Cd 1 0.21540 0.96540 0.93081 1.0000
#End

data_38-qtz_Cd
_audit_creation_method ToposPro
_Chemical_Name_Systematic CAGLIF
_cell_length_a 9.427706
_cell_length_b 9.427706
_cell_length_c 12.63616
_cell_angle_alpha 90
_cell_angle_beta 90
_cell_angle_gamma 120
_cell_volume 972.6529
_cell_formula_units_Z 3
_symmetry_space_group_name_H-M 'P 64 2 2'
_symmetry_Int_Tables_number 181
loop_
_symmetry_equiv_pos_site_id
_symmetry_equiv_pos_as_xyz
1 x,y,z
2 -x,-y,z
3 -y,x-y,1/3+z
4 -x+y,-x,2/3+z
5 x-y,x,2/3+z
6 y,-x+y,1/3+z
7 x-y,-y,-z
8 -x,-x+y,2/3-z
9 y,x,1/3-z
10 -y,-x,1/3-z
11 x,x-y,2/3-z
12 -x+y,y,-z
loop_
_atom_site_label
_atom_site_type_symbol
_atom_site_symmetry_multiplicity
_atom_site_fract_x
_atom_site_fract_y
_atom_site_fract_z
_atom_site_occupancy
N1 N 12 0.86594 0.29014 0.22453 1.0000
N2 N 6 0.73932 0.26068 0.16667 1.0000
N3 N 12 0.91454 0.18279 0.20255 1.0000
Cd1 Cd 3 0.00000 0.50000 0.33333 1.0000
#End

data_39-rho_Cd
_audit_creation_method ToposPro
_Chemical_Name_Systematic MECWOH
_cell_length_a 26.42624
_cell_length_b 26.42624
_cell_length_c 26.42624
_cell_angle_alpha 109.4712
_cell_angle_beta 109.4712
_cell_angle_gamma 109.4712
_cell_volume 14206.4
_cell_formula_units_Z 24
_symmetry_space_group_name_H-M 'P 1'
_symmetry_Int_Tables_number 1
loop_
_symmetry_equiv_pos_site_id
_symmetry_equiv_pos_as_xyz
1 x,y,z
loop_
_atom_site_label
_atom_site_type_symbol
_atom_site_symmetry_multiplicity
_atom_site_fract_x
_atom_site_fract_y
_atom_site_fract_z

```
N29 N 1 0.65212 0.27018 0.84963 1.0000
N30 N 1 0.34788 0.61806 0.19751 1.0000
N31 N 1 0.19751 0.34788 0.61806 1.0000
N32 N 1 0.80249 0.65212 0.38194 1.0000
N33 N 1 0.15037 0.80249 0.42055 1.0000
N34 N 1 0.38194 0.80249 0.65212 1.0000
N35 N 1 0.19751 0.84963 0.57945 1.0000
N36 N 1 0.27018 0.65212 0.84963 1.0000
N37 N 1 0.61806 0.34788 0.19751 1.0000
N38 N 1 0.19751 0.57945 0.84963 1.0000
N39 N 1 0.84963 0.65212 0.27018 1.0000
N40 N 1 0.19751 0.61806 0.34788 1.0000
N41 N 1 0.42055 0.80249 0.15037 1.0000
N42 N 1 0.72982 0.15037 0.34788 1.0000
N43 N 1 0.38194 0.57945 0.72982 1.0000
N44 N 1 0.84963 0.57945 0.19751 1.0000
N45 N 1 0.34788 0.15037 0.72982 1.0000
N46 N 1 0.38194 0.72982 0.57945 1.0000
N47 N 1 0.80249 0.15037 0.42055 1.0000
N48 N 1 0.65212 0.80249 0.38194 1.0000
N49 N 1 0.52561 0.32952 0.57703 1.0000
N50 N 1 0.80392 0.32952 0.75249 1.0000
N51 N 1 0.52561 0.57703 0.32952 1.0000
N52 N 1 0.24751 0.67048 0.19608 1.0000
N53 N 1 0.57703 0.32952 0.52561 1.0000
N54 N 1 0.80392 0.05143 0.47439 1.0000
N55 N 1 0.47439 0.80392 0.05143 1.0000
N56 N 1 0.94857 0.42297 0.75249 1.0000
N57 N 1 0.67048 0.24751 0.19608 1.0000
N58 N 1 0.67048 0.47439 0.42297 1.0000
N59 N 1 0.57703 0.52561 0.32952 1.0000
N60 N 1 0.57703 0.05143 0.24751 1.0000
N61 N 1 0.19608 0.52561 0.94857 1.0000
N62 N 1 0.32952 0.52561 0.57703 1.0000
N63 N 1 0.94857 0.52561 0.19608 1.0000
N64 N 1 0.32952 0.57703 0.52561 1.0000
N65 N 1 0.52561 0.19608 0.94857 1.0000
N66 N 1 0.19608 0.67048 0.24751 1.0000
N67 N 1 0.67048 0.42297 0.47439 1.0000
N68 N 1 0.47439 0.42297 0.67048 1.0000
N69 N 1 0.75249 0.42297 0.94857 1.0000
N70 N 1 0.24751 0.05143 0.57703 1.0000
N71 N 1 0.47439 0.05143 0.80392 1.0000
N72 N 1 0.47439 0.67048 0.42297 1.0000
N73 N 1 0.75249 0.80392 0.32952 1.0000
N74 N 1 0.42297 0.75249 0.94857 1.0000
N75 N 1 0.19608 0.24751 0.67048 1.0000
N76 N 1 0.32952 0.80392 0.75249 1.0000
N77 N 1 0.75249 0.32952 0.80392 1.0000
N78 N 1 0.24751 0.57703 0.05143 1.0000
N79 N 1 0.05143 0.24751 0.57703 1.0000
N80 N 1 0.94857 0.75249 0.42297 1.0000
N81 N 1 0.19608 0.94857 0.52561 1.0000
N82 N 1 0.42297 0.94857 0.75249 1.0000
N83 N 1 0.05143 0.80392 0.47439 1.0000
N84 N 1 0.32952 0.75249 0.80392 1.0000
N85 N 1 0.57703 0.24751 0.05143 1.0000
N86 N 1 0.05143 0.47439 0.80392 1.0000
N87 N 1 0.80392 0.75249 0.32952 1.0000
N88 N 1 0.05143 0.57703 0.24751 1.0000
N89 N 1 0.52561 0.94857 0.19608 1.0000
N90 N 1 0.67048 0.19608 0.24751 1.0000
N91 N 1 0.42297 0.47439 0.67048 1.0000
N92 N 1 0.80392 0.47439 0.05143 1.0000
N93 N 1 0.24751 0.19608 0.67048 1.0000
N94 N 1 0.42297 0.67048 0.47439 1.0000
N95 N 1 0.94857 0.19608 0.52561 1.0000
N96 N 1 0.75249 0.94857 0.42297 1.0000
N97 N 1 0.37147 0.22905 0.55537 1.0000
N98 N 1 0.85758 0.22905 0.67368 1.0000
N99 N 1 0.37147 0.55537 0.22905 1.0000
N100 N 1 0.32632 0.77095 0.14242 1.0000
N101 N 1 0.55537 0.22905 0.37147 1.0000
N102 N 1 0.85758 0.18390 0.62853 1.0000
N103 N 1 0.62853 0.85758 0.18390 1.0000
N104 N 1 0.81610 0.44463 0.67368 1.0000
N105 N 1 0.77095 0.32632 0.14242 1.0000
N106 N 1 0.77095 0.62853 0.44463 1.0000
N107 N 1 0.55537 0.37147 0.22905 1.0000
N108 N 1 0.55537 0.18390 0.32632 1.0000
N109 N 1 0.14242 0.37147 0.81610 1.0000
N110 N 1 0.22905 0.37147 0.55537 1.0000
N111 N 1 0.81610 0.37147 0.14242 1.0000
N112 N 1 0.22905 0.55537 0.37147 1.0000
N113 N 1 0.37147 0.14242 0.81610 1.0000
N114 N 1 0.14242 0.77095 0.32632 1.0000
N115 N 1 0.77095 0.44463 0.62853 1.0000
N116 N 1 0.62853 0.44463 0.77095 1.0000
N117 N 1 0.67368 0.44463 0.81610 1.0000
N118 N 1 0.32632 0.18390 0.55537 1.0000
N119 N 1 0.62853 0.18390 0.85758 1.0000
N120 N 1 0.62853 0.77095 0.44463 1.0000
N121 N 1 0.67368 0.85758 0.22905 1.0000
N122 N 1 0.44463 0.67368 0.81610 1.0000
N123 N 1 0.14242 0.32632 0.77095 1.0000
N124 N 1 0.22905 0.85758 0.67368 1.0000
N125 N 1 0.67368 0.22905 0.85758 1.0000
N126 N 1 0.32632 0.55537 0.18390 1.0000
N127 N 1 0.18390 0.32632 0.55537 1.0000
N128 N 1 0.81610 0.67368 0.44463 1.0000
N129 N 1 0.14242 0.81610 0.37147 1.0000
_atom_site_occupancy
N1 N 1 0.41730 0.26719 0.61650 1.0000
N2 N 1 0.84989 0.26719 0.65069 1.0000
N3 N 1 0.41730 0.61650 0.26719 1.0000
N4 N 1 0.34931 0.73281 0.15011 1.0000
N5 N 1 0.61650 0.26719 0.41730 1.0000
N6 N 1 0.84989 0.19920 0.58270 1.0000
N7 N 1 0.58270 0.84989 0.19920 1.0000
N8 N 1 0.80080 0.38350 0.65069 1.0000
N9 N 1 0.73281 0.34931 0.15011 1.0000
N10 N 1 0.73281 0.58270 0.38350 1.0000
N11 N 1 0.61650 0.41730 0.26719 1.0000
N12 N 1 0.61650 0.19920 0.34931 1.0000
N13 N 1 0.15011 0.41730 0.80080 1.0000
N14 N 1 0.26719 0.41730 0.61650 1.0000
N15 N 1 0.80080 0.41730 0.15011 1.0000
N16 N 1 0.26719 0.61650 0.41730 1.0000
N17 N 1 0.41730 0.15011 0.80080 1.0000
N18 N 1 0.15011 0.73281 0.34931 1.0000
N19 N 1 0.73281 0.38350 0.58270 1.0000
N20 N 1 0.58270 0.38350 0.73281 1.0000
N21 N 1 0.65069 0.38350 0.80080 1.0000
N22 N 1 0.34931 0.19920 0.61650 1.0000
N23 N 1 0.58270 0.19920 0.84989 1.0000
N24 N 1 0.58270 0.73281 0.38350 1.0000
N25 N 1 0.65069 0.84989 0.26719 1.0000
N26 N 1 0.38350 0.65069 0.80080 1.0000
N27 N 1 0.15011 0.34931 0.73281 1.0000
N28 N 1 0.26719 0.84989 0.65069 1.0000
N29 N 1 0.65069 0.26719 0.84989 1.0000
N30 N 1 0.34931 0.61650 0.19920 1.0000
N31 N 1 0.19920 0.34931 0.61650 1.0000
N32 N 1 0.80080 0.65069 0.38350 1.0000
N33 N 1 0.15011 0.80080 0.41730 1.0000
N34 N 1 0.38350 0.80080 0.65069 1.0000
N35 N 1 0.19920 0.84989 0.58270 1.0000
N36 N 1 0.26719 0.65069 0.84989 1.0000
N37 N 1 0.61650 0.34931 0.19920 1.0000
N38 N 1 0.19920 0.58270 0.84989 1.0000
N39 N 1 0.84989 0.65069 0.26719 1.0000
N40 N 1 0.19920 0.61650 0.34931 1.0000
N41 N 1 0.41730 0.80080 0.15011 1.0000
N42 N 1 0.73281 0.15011 0.34931 1.0000
N43 N 1 0.38350 0.58270 0.73281 1.0000
N44 N 1 0.84989 0.58270 0.19920 1.0000
N45 N 1 0.34931 0.15011 0.73281 1.0000
N46 N 1 0.38350 0.73281 0.58270 1.0000
N47 N 1 0.80080 0.15011 0.41730 1.0000
N48 N 1 0.65069 0.80080 0.38350 1.0000
N49 N 1 0.52651 0.32805 0.57460 1.0000
N50 N 1 0.80155 0.32805 0.75345 1.0000
N51 N 1 0.52651 0.57460 0.32805 1.0000
N52 N 1 0.24655 0.67195 0.19845 1.0000
N53 N 1 0.57460 0.32805 0.52651 1.0000
N54 N 1 0.80155 0.04809 0.47349 1.0000
N55 N 1 0.47349 0.80155 0.04809 1.0000
N56 N 1 0.95191 0.42540 0.75345 1.0000
N57 N 1 0.67195 0.24655 0.19845 1.0000
N58 N 1 0.67195 0.47349 0.42540 1.0000
N59 N 1 0.57460 0.52651 0.32805 1.0000
N60 N 1 0.57460 0.04809 0.24655 1.0000
N61 N 1 0.19845 0.52651 0.95191 1.0000
N62 N 1 0.32805 0.52651 0.57460 1.0000
N63 N 1 0.95191 0.52651 0.19845 1.0000
N64 N 1 0.32805 0.57460 0.52651 1.0000
N65 N 1 0.52651 0.19845 0.95191 1.0000
N66 N 1 0.19845 0.67195 0.24655 1.0000
N67 N 1 0.67195 0.42540 0.47349 1.0000
N68 N 1 0.47349 0.42540 0.67195 1.0000
N69 N 1 0.75345 0.42540 0.95191 1.0000
N70 N 1 0.24655 0.04809 0.57460 1.0000
N71 N 1 0.47349 0.04809 0.80155 1.0000
N72 N 1 0.47349 0.67195 0.42540 1.0000
N73 N 1 0.75345 0.80155 0.32805 1.0000
N74 N 1 0.42540 0.75345 0.95191 1.0000
N75 N 1 0.19845 0.24655 0.67195 1.0000
N76 N 1 0.32805 0.80155 0.75345 1.0000
N77 N 1 0.75345 0.32805 0.80155 1.0000
N78 N 1 0.24655 0.57460 0.04809 1.0000
N79 N 1 0.04809 0.24655 0.57460 1.0000
N80 N 1 0.95191 0.75345 0.42540 1.0000
N81 N 1 0.19845 0.95191 0.52651 1.0000
N82 N 1 0.42540 0.95191 0.75345 1.0000
N83 N 1 0.04809 0.80155 0.47349 1.0000
N84 N 1 0.32805 0.75345 0.80155 1.0000
N85 N 1 0.57460 0.24655 0.04809 1.0000
N86 N 1 0.04809 0.47349 0.80155 1.0000
N87 N 1 0.80155 0.75345 0.32805 1.0000
N88 N 1 0.04809 0.57460 0.24655 1.0000
N89 N 1 0.52651 0.95191 0.19845 1.0000
N90 N 1 0.67195 0.19845 0.24655 1.0000
N91 N 1 0.42540 0.47349 0.67195 1.0000
N92 N 1 0.80155 0.47349 0.04809 1.0000
N93 N 1 0.24655 0.19845 0.67195 1.0000
N94 N 1 0.42540 0.67195 0.47349 1.0000
N95 N 1 0.95191 0.19845 0.52651 1.0000
N96 N 1 0.75345 0.95191 0.42540 1.0000
N97 N 1 0.37147 0.22881 0.55799 1.0000
N98 N 1 0.85734 0.22881 0.67082 1.0000
N99 N 1 0.37147 0.55799 0.22881 1.0000
N100 N 1 0.32918 0.77119 0.14266 1.0000
```

N130 N 1 0.44463 0.81610 0.67368 1.0000
N131 N 1 0.18390 0.85758 0.62853 1.0000
N132 N 1 0.22905 0.67368 0.85758 1.0000
N133 N 1 0.55537 0.32632 0.18390 1.0000
N134 N 1 0.18390 0.62853 0.85758 1.0000
N135 N 1 0.85758 0.67368 0.22905 1.0000
N136 N 1 0.18390 0.55537 0.32632 1.0000
N137 N 1 0.37147 0.81610 0.14242 1.0000
N138 N 1 0.77095 0.14242 0.32632 1.0000
N139 N 1 0.44463 0.62853 0.77095 1.0000
N140 N 1 0.85758 0.62853 0.18390 1.0000
N141 N 1 0.32632 0.14242 0.77095 1.0000
N142 N 1 0.44463 0.77095 0.62853 1.0000
N143 N 1 0.81610 0.14242 0.37147 1.0000
N144 N 1 0.67368 0.81610 0.44463 1.0000
N145 N 1 0.40611 0.25101 0.65712 1.0000
N146 N 1 0.84491 0.25101 0.59389 1.0000
N147 N 1 0.40611 0.65712 0.25101 1.0000
N148 N 1 0.40611 0.74899 0.15509 1.0000
N149 N 1 0.65712 0.25101 0.40611 1.0000
N150 N 1 0.59389 0.84491 0.25101 1.0000
N151 N 1 0.74899 0.34288 0.59389 1.0000
N152 N 1 0.74899 0.40611 0.15509 1.0000
N153 N 1 0.74899 0.59389 0.34288 1.0000
N154 N 1 0.65712 0.40611 0.25101 1.0000
N155 N 1 0.15509 0.40611 0.74899 1.0000
N156 N 1 0.25101 0.40611 0.65712 1.0000
N157 N 1 0.25101 0.65712 0.40611 1.0000
N158 N 1 0.40611 0.15509 0.74899 1.0000
N159 N 1 0.15509 0.74899 0.40611 1.0000
N160 N 1 0.59389 0.34288 0.74899 1.0000
N161 N 1 0.59389 0.25101 0.84491 1.0000
N162 N 1 0.59389 0.74899 0.34288 1.0000
N163 N 1 0.34288 0.59389 0.74899 1.0000
N164 N 1 0.25101 0.84491 0.59389 1.0000
N165 N 1 0.34288 0.74899 0.59389 1.0000
N166 N 1 0.25101 0.59389 0.84491 1.0000
N167 N 1 0.84491 0.59389 0.25101 1.0000
N168 N 1 0.74899 0.15509 0.40611 1.0000
N169 N 1 0.50899 0.26663 0.54087 1.0000
N170 N 1 0.75764 0.26663 0.72576 1.0000
N171 N 1 0.50899 0.54087 0.26663 1.0000
N172 N 1 0.27424 0.73337 0.24236 1.0000
N173 N 1 0.54087 0.26663 0.50899 1.0000
N174 N 1 0.75764 0.03188 0.49101 1.0000
N175 N 1 0.49101 0.75764 0.03188 1.0000
N176 N 1 0.96812 0.45913 0.72576 1.0000
N177 N 1 0.73337 0.27424 0.24236 1.0000
N178 N 1 0.73337 0.49101 0.45913 1.0000
N179 N 1 0.54087 0.50899 0.26663 1.0000
N180 N 1 0.54087 0.03188 0.27424 1.0000
N181 N 1 0.24236 0.50899 0.96812 1.0000
N182 N 1 0.26663 0.50899 0.54087 1.0000
N183 N 1 0.96812 0.50899 0.24236 1.0000
N184 N 1 0.26663 0.54087 0.50899 1.0000
N185 N 1 0.50899 0.24236 0.96812 1.0000
N186 N 1 0.24236 0.73337 0.27424 1.0000
N187 N 1 0.73337 0.45913 0.49101 1.0000
N188 N 1 0.49101 0.45913 0.73337 1.0000
N189 N 1 0.72576 0.45913 0.96812 1.0000
N190 N 1 0.27424 0.03188 0.54087 1.0000
N191 N 1 0.49101 0.03188 0.75764 1.0000
N192 N 1 0.49101 0.73337 0.45913 1.0000
N193 N 1 0.72576 0.75764 0.26663 1.0000
N194 N 1 0.45913 0.72576 0.96812 1.0000
N195 N 1 0.24236 0.27424 0.73337 1.0000
N196 N 1 0.26663 0.75764 0.72576 1.0000
N197 N 1 0.72576 0.26663 0.75764 1.0000
N198 N 1 0.27424 0.54087 0.03188 1.0000
N199 N 1 0.03188 0.27424 0.54087 1.0000
N200 N 1 0.96812 0.72576 0.45913 1.0000
N201 N 1 0.24236 0.96812 0.50899 1.0000
N202 N 1 0.45913 0.96812 0.72576 1.0000
N203 N 1 0.03188 0.75764 0.49101 1.0000
N204 N 1 0.26663 0.72576 0.75764 1.0000
N205 N 1 0.54087 0.27424 0.03188 1.0000
N206 N 1 0.03188 0.49101 0.75764 1.0000
N207 N 1 0.75764 0.72576 0.26663 1.0000
N208 N 1 0.03188 0.54087 0.27424 1.0000
N209 N 1 0.50899 0.96812 0.24236 1.0000
N210 N 1 0.73337 0.24236 0.27424 1.0000
N211 N 1 0.45913 0.49101 0.73337 1.0000
N212 N 1 0.75764 0.49101 0.03188 1.0000
N213 N 1 0.27424 0.24236 0.73337 1.0000
N214 N 1 0.45913 0.73337 0.49101 1.0000
N215 N 1 0.96812 0.24236 0.50899 1.0000
N216 N 1 0.72576 0.96812 0.45913 1.0000
N217 N 1 0.56794 0.36863 0.56794 1.0000
N218 N 1 0.80069 0.36863 0.80069 1.0000
N219 N 1 0.56794 0.56794 0.36863 1.0000
N220 N 1 0.19931 0.63137 0.19931 1.0000
N221 N 1 0.80069 0.00000 0.43206 1.0000
N222 N 1 0.43206 0.80069 0.00000 1.0000
N223 N 1 0.00000 0.43206 0.80069 1.0000
N224 N 1 0.63137 0.19931 0.19931 1.0000
N225 N 1 0.63137 0.43206 0.43206 1.0000
N226 N 1 0.56794 0.00000 0.19931 1.0000
N227 N 1 0.19931 0.56794 0.00000 1.0000
N228 N 1 0.36863 0.56794 0.56794 1.0000
N229 N 1 0.00000 0.56794 0.19931 1.0000
N230 N 1 0.56794 0.19931 0.00000 1.0000

N101 N 1 0.55799 0.22881 0.37147 1.0000
N102 N 1 0.85734 0.18652 0.62853 1.0000
N103 N 1 0.62853 0.85734 0.18652 1.0000
N104 N 1 0.81348 0.44201 0.67082 1.0000
N105 N 1 0.77119 0.32918 0.14266 1.0000
N106 N 1 0.77119 0.62853 0.44201 1.0000
N107 N 1 0.55799 0.37147 0.22881 1.0000
N108 N 1 0.55799 0.18652 0.32918 1.0000
N109 N 1 0.14266 0.37147 0.81348 1.0000
N110 N 1 0.22881 0.37147 0.55799 1.0000
N111 N 1 0.81348 0.37147 0.14266 1.0000
N112 N 1 0.22881 0.55799 0.37147 1.0000
N113 N 1 0.37147 0.14266 0.81348 1.0000
N114 N 1 0.14266 0.77119 0.32918 1.0000
N115 N 1 0.77119 0.44201 0.62853 1.0000
N116 N 1 0.62853 0.44201 0.77119 1.0000
N117 N 1 0.67082 0.44201 0.81348 1.0000
N118 N 1 0.32918 0.18652 0.55799 1.0000
N119 N 1 0.62853 0.18652 0.85734 1.0000
N120 N 1 0.62853 0.77119 0.44201 1.0000
N121 N 1 0.67082 0.85734 0.22881 1.0000
N122 N 1 0.44201 0.67082 0.81348 1.0000
N123 N 1 0.14266 0.32918 0.77119 1.0000
N124 N 1 0.22881 0.85734 0.67082 1.0000
N125 N 1 0.67082 0.22881 0.85734 1.0000
N126 N 1 0.32918 0.55799 0.18652 1.0000
N127 N 1 0.18652 0.32918 0.55799 1.0000
N128 N 1 0.81348 0.67082 0.44201 1.0000
N129 N 1 0.14266 0.81348 0.37147 1.0000
N130 N 1 0.44201 0.81348 0.67082 1.0000
N131 N 1 0.18652 0.85734 0.62853 1.0000
N132 N 1 0.22881 0.67082 0.85734 1.0000
N133 N 1 0.55799 0.32918 0.18652 1.0000
N134 N 1 0.18652 0.62853 0.85734 1.0000
N135 N 1 0.85734 0.67082 0.22881 1.0000
N136 N 1 0.18652 0.55799 0.32918 1.0000
N137 N 1 0.37147 0.81348 0.14266 1.0000
N138 N 1 0.77119 0.14266 0.32918 1.0000
N139 N 1 0.44201 0.62853 0.77119 1.0000
N140 N 1 0.85734 0.62853 0.18652 1.0000
N141 N 1 0.32918 0.14266 0.77119 1.0000
N142 N 1 0.44201 0.77119 0.62853 1.0000
N143 N 1 0.81348 0.14266 0.37147 1.0000
N144 N 1 0.67082 0.81348 0.44201 1.0000
N145 N 1 0.40379 0.24917 0.65297 1.0000
N146 N 1 0.84538 0.24917 0.59621 1.0000
N147 N 1 0.40379 0.65297 0.24917 1.0000
N148 N 1 0.40379 0.75083 0.15462 1.0000
N149 N 1 0.65297 0.24917 0.40379 1.0000
N150 N 1 0.59621 0.84538 0.24917 1.0000
N151 N 1 0.75083 0.34703 0.59621 1.0000
N152 N 1 0.75083 0.40379 0.15462 1.0000
N153 N 1 0.75083 0.59621 0.34703 1.0000
N154 N 1 0.65297 0.40379 0.24917 1.0000
N155 N 1 0.15462 0.40379 0.75083 1.0000
N156 N 1 0.24917 0.40379 0.65297 1.0000
N157 N 1 0.24917 0.65297 0.40379 1.0000
N158 N 1 0.40379 0.15462 0.75083 1.0000
N159 N 1 0.15462 0.75083 0.40379 1.0000
N160 N 1 0.59621 0.34703 0.75083 1.0000
N161 N 1 0.59621 0.24917 0.84538 1.0000
N162 N 1 0.59621 0.75083 0.34703 1.0000
N163 N 1 0.34703 0.59621 0.75083 1.0000
N164 N 1 0.24917 0.84538 0.59621 1.0000
N165 N 1 0.34703 0.75083 0.59621 1.0000
N166 N 1 0.24917 0.59621 0.84538 1.0000
N167 N 1 0.84538 0.59621 0.24917 1.0000
N168 N 1 0.75083 0.15462 0.40379 1.0000
N169 N 1 0.51104 0.26938 0.54091 1.0000
N170 N 1 0.75834 0.26938 0.72847 1.0000
N171 N 1 0.51104 0.54091 0.26938 1.0000
N172 N 1 0.27153 0.73062 0.24166 1.0000
N173 N 1 0.54091 0.26938 0.51104 1.0000
N174 N 1 0.75834 0.02987 0.48896 1.0000
N175 N 1 0.48896 0.75834 0.02987 1.0000
N176 N 1 0.97013 0.45909 0.72847 1.0000
N177 N 1 0.73062 0.27153 0.24166 1.0000
N178 N 1 0.73062 0.48896 0.45909 1.0000
N179 N 1 0.54091 0.51104 0.26938 1.0000
N180 N 1 0.54091 0.02987 0.27153 1.0000
N181 N 1 0.24166 0.51104 0.97013 1.0000
N182 N 1 0.26938 0.51104 0.54091 1.0000
N183 N 1 0.97013 0.51104 0.24166 1.0000
N184 N 1 0.26938 0.54091 0.51104 1.0000
N185 N 1 0.51104 0.24166 0.97013 1.0000
N186 N 1 0.24166 0.73062 0.27153 1.0000
N187 N 1 0.73062 0.45909 0.48896 1.0000
N188 N 1 0.48896 0.45909 0.73062 1.0000
N189 N 1 0.72847 0.45909 0.97013 1.0000
N190 N 1 0.27153 0.02987 0.54091 1.0000
N191 N 1 0.48896 0.02987 0.75834 1.0000
N192 N 1 0.48896 0.73062 0.45909 1.0000
N193 N 1 0.72847 0.75834 0.26938 1.0000
N194 N 1 0.45909 0.72847 0.97013 1.0000
N195 N 1 0.24166 0.27153 0.73062 1.0000
N196 N 1 0.26938 0.75834 0.72847 1.0000
N197 N 1 0.72847 0.26938 0.75834 1.0000
N198 N 1 0.27153 0.54091 0.02987 1.0000
N199 N 1 0.02987 0.27153 0.54091 1.0000
N200 N 1 0.97013 0.72847 0.45909 1.0000
N201 N 1 0.24166 0.97013 0.51104 1.0000

N231 N 1 0.43206 0.43206 0.63137 1.0000
N232 N 1 0.80069 0.43206 0.00000 1.0000
N233 N 1 0.19931 0.00000 0.56794 1.0000
N234 N 1 0.43206 0.00000 0.80069 1.0000
N235 N 1 0.43206 0.63137 0.43206 1.0000
N236 N 1 0.80069 0.80069 0.36863 1.0000
N237 N 1 0.19931 0.19931 0.63137 1.0000
N238 N 1 0.36863 0.80069 0.80069 1.0000
N239 N 1 0.00000 0.19931 0.56794 1.0000
N240 N 1 0.00000 0.80069 0.43206 1.0000
Zn1 Zn 1 0.50000 0.35349 0.64651 1.0000
Zn2 Zn 1 0.85349 0.35349 0.70698 1.0000
Zn3 Zn 1 0.50000 0.64651 0.35349 1.0000
Zn4 Zn 1 0.29302 0.64651 0.14651 1.0000
Zn5 Zn 1 0.64651 0.35349 0.50000 1.0000
Zn6 Zn 1 0.85349 0.14651 0.50000 1.0000
Zn7 Zn 1 0.50000 0.85349 0.14651 1.0000
Zn8 Zn 1 0.64651 0.29302 0.14651 1.0000
Zn9 Zn 1 0.64651 0.50000 0.35349 1.0000
Zn10 Zn 1 0.64651 0.14651 0.29302 1.0000
Zn11 Zn 1 0.14651 0.50000 0.85349 1.0000
Zn12 Zn 1 0.35349 0.50000 0.64651 1.0000
Zn13 Zn 1 0.85349 0.50000 0.14651 1.0000
Zn14 Zn 1 0.35349 0.64651 0.50000 1.0000
Zn15 Zn 1 0.50000 0.14651 0.85349 1.0000
Zn16 Zn 1 0.14651 0.64651 0.29302 1.0000
Zn17 Zn 1 0.70698 0.35349 0.85349 1.0000
Zn18 Zn 1 0.29302 0.14651 0.64651 1.0000
Zn19 Zn 1 0.70698 0.85349 0.35349 1.0000
Zn20 Zn 1 0.35349 0.70698 0.85349 1.0000
Zn21 Zn 1 0.14651 0.29302 0.64651 1.0000
Zn22 Zn 1 0.35349 0.85349 0.70698 1.0000
Zn23 Zn 1 0.85349 0.70698 0.35349 1.0000
Zn24 Zn 1 0.14651 0.85349 0.50000 1.0000
#End

N202 N 1 0.45909 0.97013 0.72847 1.0000
N203 N 1 0.02987 0.75834 0.48896 1.0000
N204 N 1 0.26938 0.72847 0.75834 1.0000
N205 N 1 0.54091 0.27153 0.02987 1.0000
N206 N 1 0.02987 0.48896 0.75834 1.0000
N207 N 1 0.75834 0.72847 0.26938 1.0000
N208 N 1 0.02987 0.54091 0.27153 1.0000
N209 N 1 0.51104 0.97013 0.24166 1.0000
N210 N 1 0.73062 0.24166 0.27153 1.0000
N211 N 1 0.45909 0.48896 0.73062 1.0000
N212 N 1 0.75834 0.48896 0.02987 1.0000
N213 N 1 0.27153 0.24166 0.73062 1.0000
N214 N 1 0.45909 0.73062 0.48896 1.0000
N215 N 1 0.97013 0.24166 0.51104 1.0000
N216 N 1 0.72847 0.97013 0.45909 1.0000
N217 N 1 0.56604 0.36459 0.56604 1.0000
N218 N 1 0.79856 0.36459 0.79856 1.0000
N219 N 1 0.56604 0.56604 0.36459 1.0000
N220 N 1 0.20144 0.63541 0.20144 1.0000
N221 N 1 0.79856 0.00000 0.43396 1.0000
N222 N 1 0.43396 0.79856 0.00000 1.0000
N223 N 1 0.00000 0.43396 0.79856 1.0000
N224 N 1 0.63541 0.20144 0.20144 1.0000
N225 N 1 0.63541 0.43396 0.43396 1.0000
N226 N 1 0.56604 0.00000 0.20144 1.0000
N227 N 1 0.20144 0.56604 0.00000 1.0000
N228 N 1 0.36459 0.56604 0.56604 1.0000
N229 N 1 0.00000 0.56604 0.20144 1.0000
N230 N 1 0.56604 0.20144 0.00000 1.0000
N231 N 1 0.43396 0.43396 0.63541 1.0000
N232 N 1 0.79856 0.43396 0.00000 1.0000
N233 N 1 0.20144 0.00000 0.56604 1.0000
N234 N 1 0.43396 0.00000 0.79856 1.0000
N235 N 1 0.43396 0.63541 0.43396 1.0000
N236 N 1 0.79856 0.79856 0.36459 1.0000
N237 N 1 0.20144 0.20144 0.63541 1.0000
N238 N 1 0.36459 0.79856 0.79856 1.0000
N239 N 1 0.00000 0.20144 0.56604 1.0000
N240 N 1 0.00000 0.79856 0.43396 1.0000
Cd1 Cd 1 0.50000 0.35352 0.64648 1.0000
Cd2 Cd 1 0.85352 0.35352 0.70705 1.0000
Cd3 Cd 1 0.50000 0.64648 0.35352 1.0000
Cd4 Cd 1 0.29295 0.64648 0.14648 1.0000
Cd5 Cd 1 0.64648 0.35352 0.50000 1.0000
Cd6 Cd 1 0.85352 0.14648 0.50000 1.0000
Cd7 Cd 1 0.50000 0.85352 0.14648 1.0000
Cd8 Cd 1 0.64648 0.29295 0.14648 1.0000
Cd9 Cd 1 0.64648 0.50000 0.35352 1.0000
Cd10 Cd 1 0.64648 0.14648 0.29295 1.0000
Cd11 Cd 1 0.14648 0.50000 0.85352 1.0000
Cd12 Cd 1 0.35352 0.50000 0.64648 1.0000
Cd13 Cd 1 0.85352 0.50000 0.14648 1.0000
Cd14 Cd 1 0.35352 0.64648 0.50000 1.0000
Cd15 Cd 1 0.50000 0.14648 0.85352 1.0000
Cd16 Cd 1 0.14648 0.64648 0.29295 1.0000
Cd17 Cd 1 0.70705 0.35352 0.85352 1.0000
Cd18 Cd 1 0.29295 0.14648 0.64648 1.0000
Cd19 Cd 1 0.70705 0.85352 0.35352 1.0000
Cd20 Cd 1 0.35352 0.70705 0.85352 1.0000
Cd21 Cd 1 0.14648 0.29295 0.64648 1.0000
Cd22 Cd 1 0.35352 0.85352 0.70705 1.0000
Cd23 Cd 1 0.85352 0.70705 0.35352 1.0000
Cd24 Cd 1 0.14648 0.85352 0.50000 1.0000
#End
